# Total synthesis of aristolactam alkaloids *via* synergistic C–H bond activation and dehydro-Diels–Alder reactions†
†Electronic supplementary information (ESI) available: Detailed experimental procedures and spectroscopic data. CCDC 1526825. For ESI and crystallographic data in CIF or other electronic format see DOI: 10.1039/c7sc00161d


**DOI:** 10.1039/c7sc00161d

**Published:** 2017-03-23

**Authors:** Mallu Chenna Reddy, Masilamani Jeganmohan

**Affiliations:** a Department of Chemistry , Indian Institute of Science Education and Research , Pune 411021 , India . Email: mjeganmohan@iiserpune.ac.in; b Department of Chemistry , Indian Institute of Technology Madras , Chennai 600036 , Tamil Nadu , India . Email: mjeganmohan@iitm.ac.in

## Abstract

A concise total synthesis of aristolactam alkaloids by a synergistic combination of C–H bond activation and dehydro-Diels–Alder reactions is described. To achieve the synthesis two new synthetic methodologies, namely the oxidative cyclization of benzamides with vinyl sulfone leading to 3-methyleneisoindolin-1-ones *via* a ruthenium-catalyzed C–H bond activation, and a dehydro-Diels–Alder reaction followed by the fluoride ion mediated desulfonylation of 3-methyleneisoindolin-1-ones with benzynes, were developed. The method presented allows the opportunity for the construction of all the rings of aristolactams from easily available starting materials.

## Abstract 2

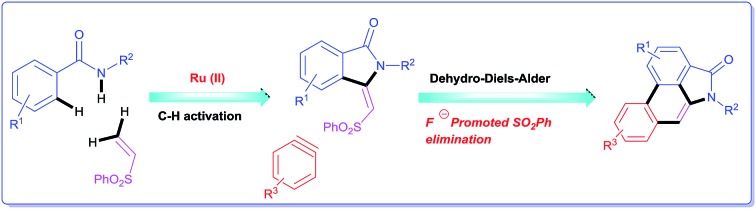
A concise total synthesis of aristolactam alkaloids by a synergistic combination of C–H bond activation and dehydro-Diels–Alder reactions is described.

## Introduction

Aristolactams are naturally occurring phenanthrene lactam alkaloids. These alkaloids are isolated from *Aristolochiaceae*, *Annonaceae*, *Piper Piperaceae*, and *Saururaceae* plant species.^1^–^3^ Aristolactams are frequently used as folk medicines in several countries.2d–f Meanwhile, these molecules show an interesting array of biological properties such as anti-inflammatory, anti-platelet, anti-mycobacterial, neuroprotective and anti-cancer activities.^2^,^3^ Due to their unique structural features and potential biological activities, a considerable amount of effort has been devoted to synthesizing these molecules by several research groups.^4^ After surveying all these elegant contributions, we understood that a general and easily approachable method for synthesizing these alkaloids with a minimum number of steps from easily affordable starting materials is needed. Meanwhile, the new method should be general for the preparation of numerous aristolactam derivatives in order to explore the utility of these molecules in various areas. Particularly, the utility of these alkaloids in various biological applications has been extensively increased in the past two decades.

Herein, we wish to report an efficient two step synthesis of aristolactam alkaloids from easily available and affordable starting materials such as aromatic acids, alkyl amines and alkenes. To execute the synthesis two new synthetic methodologies, namely the preparation of 3-methyleneisoindolin-1-ones *via* a ruthenium-catalyzed oxidative cyclization of aromatic amides with vinyl sulfone, and a dehydro-Diels–Alder reaction followed by SO_2_Ph cleavage of 3-methyleneisoindolin-1-ones with benzynes, were developed. The present method is compatible for the preparation of various aristolactam derivatives including sensitive I, Br, Cl, F and CF_3_ functional groups. The combination of C–H bond activation and dehydro-Diels–Alder reactions allows a short and efficient synthesis of several aristolactam alkaloids in good yields.
1

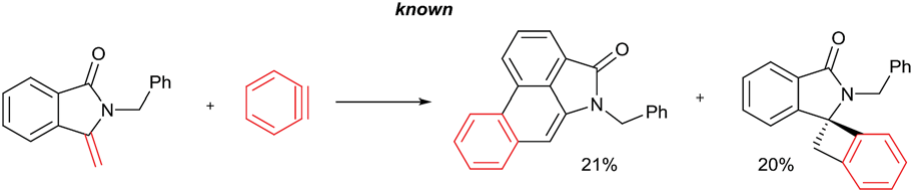



2

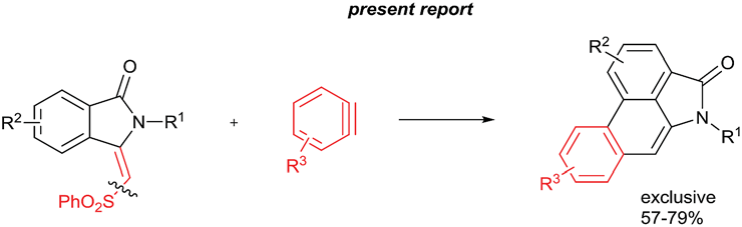




**Scheme 1:**
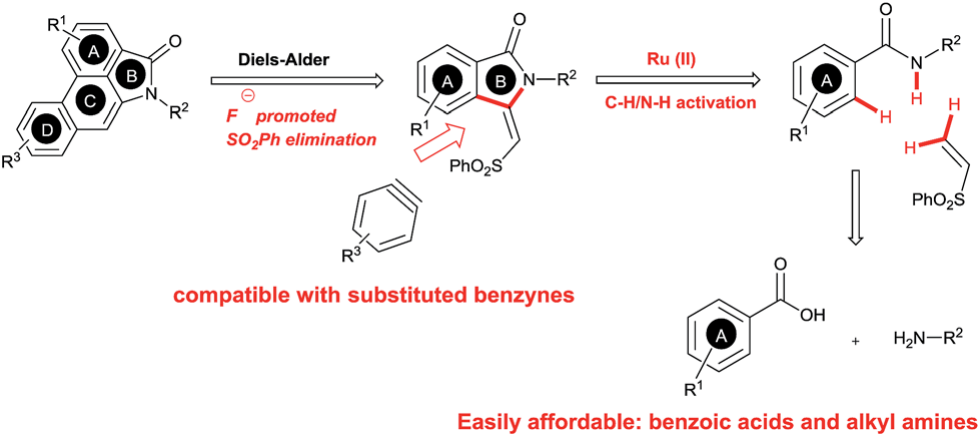
Retrosynthetic analysis.

The goal of this work is to construct aristolactam cyclic rings A–D in a simple manner from easily affordable starting materials (Scheme 1). Rings A and B having 3-methyleneisoindolone can be constructed *via* a metal-catalyzed C–H/N–H annulation of substituted benzamides with alkenes in one pot.^5^–^7^ Substituted benzamides can be easily prepared from benzoic acids and amines. Rings C and D can be constructed in one pot *via* the dehydro-Diels–Alder reaction of 3-methyleneisoindolin-1-ones with benzynes.^8^,^9^ However, this type of cycloaddition reaction is not very effective, because it provides competing side products along with the expected product (eqn (1)).4*i* To overcome this problem, we engineered a molecule that has a cleavable SO_2_Ph group at the β-carbon of alkene of 3-methyleneisoindolin-1-one. After the cycloaddition reaction, the sulfonyl group can be easily cleaved by a fluoride source in the same step (eqn (2)). Thus, the cycloaddition reaction can be done in a highly selective manner.

## Results and discussion

**Scheme 2:**
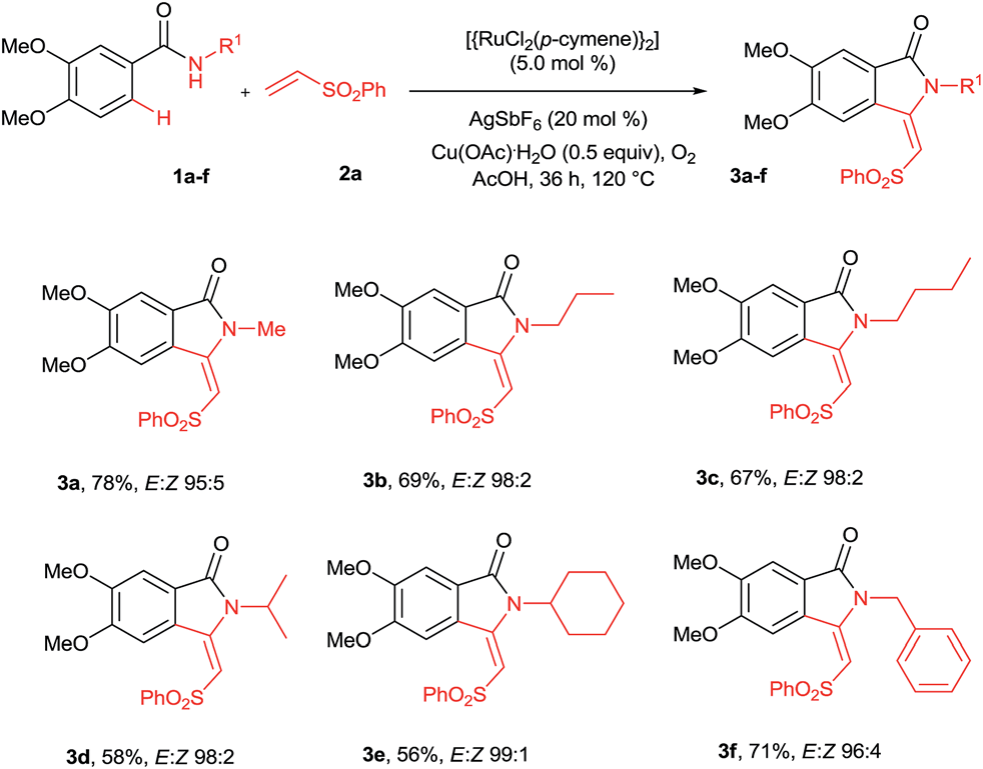
Cyclization of *N*-substituted benzamides.

Our continuous interest in ruthenium-catalyzed C–H bond activation reaction prompted us to explore the possibility of developing a new synthetic route for the synthesis of key intermediates 3-methyleneisoindolin-1-ones *via* the ruthenium-catalyzed oxidative cyclization of benzamides with vinyl phenyl sulfone.6c,d,7l The oxidative cyclization of *N*-methyl benzamide **1a** with phenyl vinyl sulfone (**2a**) in the presence of [{RuCl_2_(*p*-cymene)}_2_] (5 mol%), AgSbF_6_ (20 mol%) and Cu(OAc)_2_·H_2_O (0.5 equiv.) under oxygen at 120 °C for 36 h provided 3-methyleneisoindolin-1-one **3a** in 78% yield in an 95 : 5 *E*/*Z* ratio (Scheme 2).

Initially, the cyclization reaction was examined with various solvents such as 1,2-dichloroethane, THF, 1,4-dioxane, DMF, toluene, CF_3_COOH and CH_3_COOH (Table 1). Among them, acetic acid was effective yielding product **3a** in 78% yield (entry 7). Other solvents such as toluene and THF were less effective, affording product **3a** in 20% and 15% yields, respectively (entries 2 and 5). The remaining solvents were not effective. The cyclization reaction was further examined with additives AgSbF_6_, AgBF_4_, AgOTf and KPF_6_. Among them, AgSbF_6_ was effective, providing product **3a** in 78% yield (entry 7). The remaining additives were less effective for the cyclization reaction (entries 8–10). The cyclization reaction did not proceed without AgSbF_6_ (entry 11). AgSbF_6_ is used to generate a cationic ruthenium species for activating weak amide group assisted C–H bonds.5c,d Cu(OAc)_2_·H_2_O has been widely used as an oxidant for weak chelating group assisted C–H bond activation.5*c* Usually, a 2.0 equiv. amount of copper source is needed for this type of reaction. However, in the present reaction, a 0.5 equiv. amount of copper source was used and the remaining amount of the copper source was regenerated under oxygen. The cyclization reaction was examined with various substrates such as methyl, propyl, butyl, isopropyl, cyclohexyl and benzyl substituted benzamides **1b–f** (Scheme 2). These reactions worked very well, providing the expected cyclization products **3b–f** in 69%, 67%, 58%, 56% and 71% yields, respectively, in 96 : 4 to 99 : 1 *E*/*Z* ratios.

**Table 1:** Optimization studies^*a*^

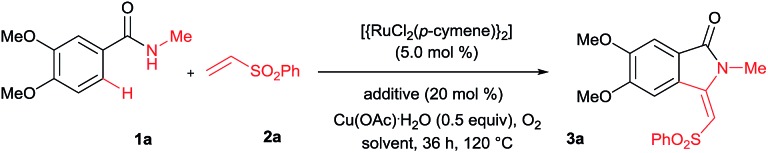			
Entry	Solvent	Additive	Yield of **3a**^*b*^ (%)
1	ClCH_2_CH_2_Cl	AgSbF_6_	—
2	THF	AgSbF_6_	15
3	1,4-Dioxane	AgSbF_6_	—
4	DMF	AgSbF_6_	—
5	Toluene	AgSbF_6_	20
6	CF_3_COOH	AgSbF_6_	—
7	CH_3_COOH	AgSbF_6_	78
8	CH_3_COOH	AgBF_4_	42
9	CH_3_COOH	AgOTf	46
10	CH_3_COOH	KPF_6_	15
11	CH_3_COOH	—	NR

^*a*^All reactions were carried out under the following conditions: **1a** (75 mg), **2a** (1.5 equiv.), [{RuCl_2_(*p*-cymene)}_2_] (5 mol%), additive (20 mol%) and Cu(OAc)_2_·H_2_O (50 mol%) in solvent at 120 °C for 36 h under an oxygen atmosphere.

^*b*^Isolated yield.

**Scheme 3:**
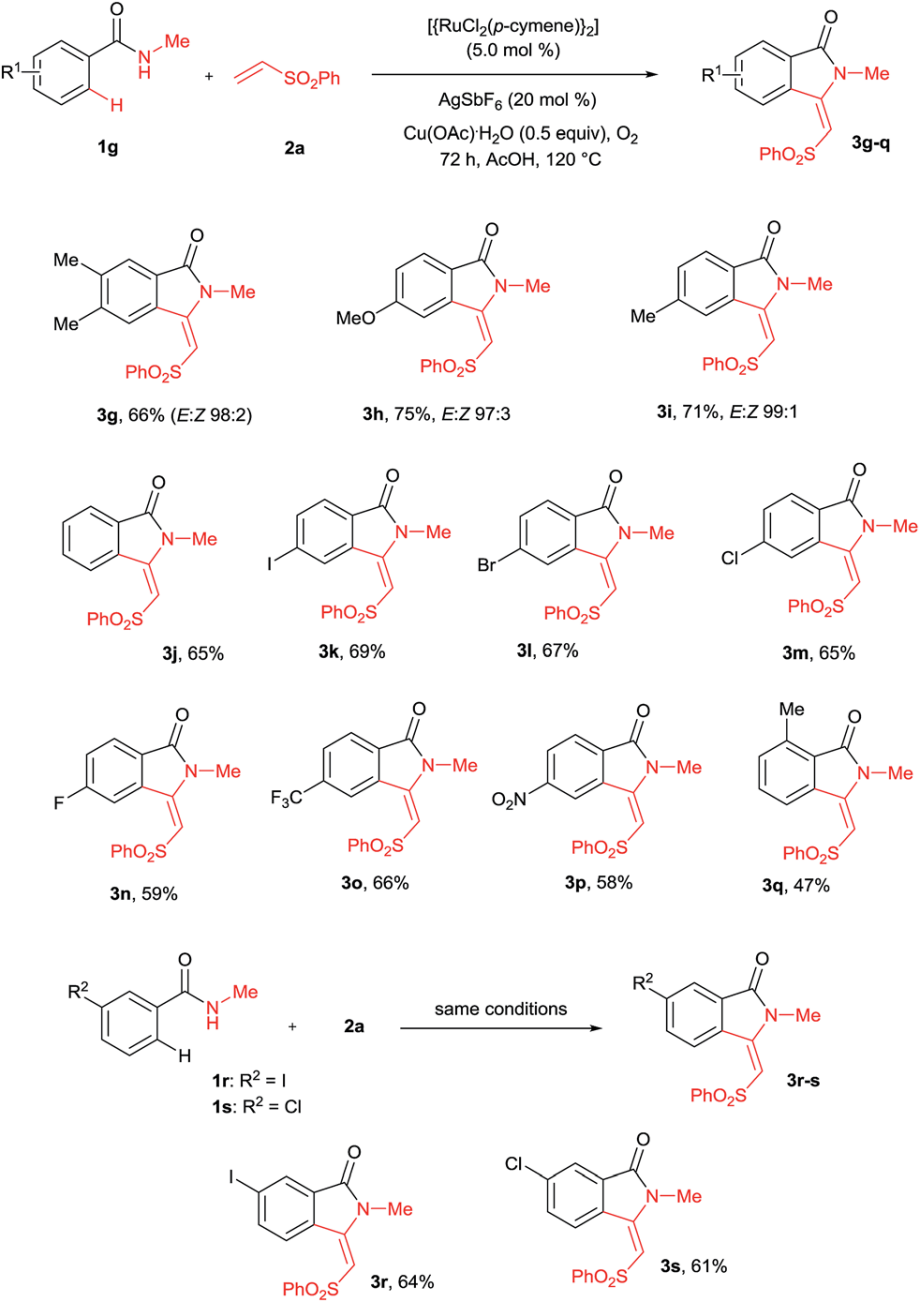
Scope of substituted benzamides.

A variety of substituted benzamides **1g–s** were compatible for the cyclization reaction (Scheme 3). Electron-releasing (OMe and Me) and halogen (I, Br, Cl and F) substituted benzamides **1g–n** efficiently reacted with **2a** affording isoindolin-1-ones **3g–n** in good yields. The less reactive electron withdrawing (CF_3_ and NO_2_) substituted benzamides **1o–p** also efficiently reacted with **2a** providing products **3o** and **3p** in good yields. Similarly, *ortho* and *meta* substituted benzamides **1q–s** also efficiently participated in the reaction, giving products **3q–s** in 47%, 64% and 61% yields, respectively. Particularly, in the *meta* substituted benzamides **1r–s**, C–H bond activation takes place at a less hindered C_6_–H.
3

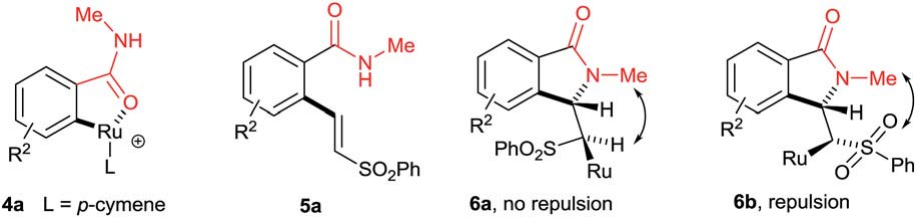




The cyclization reaction proceeds *via* a cationic ruthenium(ii) catalyzed *ortho* alkenylation of benzamide **1a** with alkene **2a***via* intermediate **4a**, providing *ortho* alkenylated benzamide **5a**.7*i* Intramolecular addition of the amide N–H bond of **5a** into an alkene moiety affords product **3** (eqn (1), for the detailed mechanism see the ESI†). It is important to note that a minor amount of *Z* stereoisomer was observed in the cyclization of electron-rich OMe and Me substituted benzamides. Intermediate **6b** accounts for the formation of the *Z* stereoisomer. Presently, the exact reason for the observation of a minor amount of the *Z* stereoisomer is unclear. However, in halogen and electron-withdrawing substituted benzamides, the *E* stereoisomer was observed exclusively. The observation of high *E* stereoselectivity for product **3** is mainly due to the formation of intermediate **6a** in which the sulfonyl moiety of the alkene and the cyclic tertiary C–N–Me bond are anti to each other (eqn (3)). Syn coplanarity is avoided due to the steric hindrance of the methyl and SO_2_Ph groups of intermediate **6b**.7*i*

**Scheme 4:**
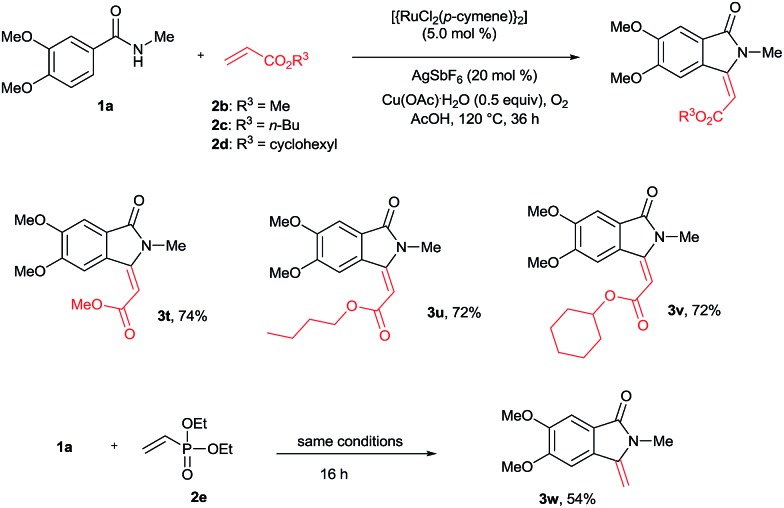
Scope of alkenes.

The cyclization reaction was further examined with various alkenes (Scheme 4). Methyl, *n*-butyl and cyclohexyl acrylates **2b–d** efficiently reacted with **1a** yielding cyclization products **3t–v** in good yields. In these reactions, only *E* stereoselectivity was observed. Diethyl vinylphosphonate (**2e**) was also efficiently involved in the reaction, giving product **3w** in 54% yield with a free exo double bond. In the product **3w**, phosphonate (P=O(OEt)_2_) was cleaved under the present reaction conditions. The cyclization reaction was not compatible with acrylonitrile, methyl vinyl ketone and styrene.

**Scheme 5:**
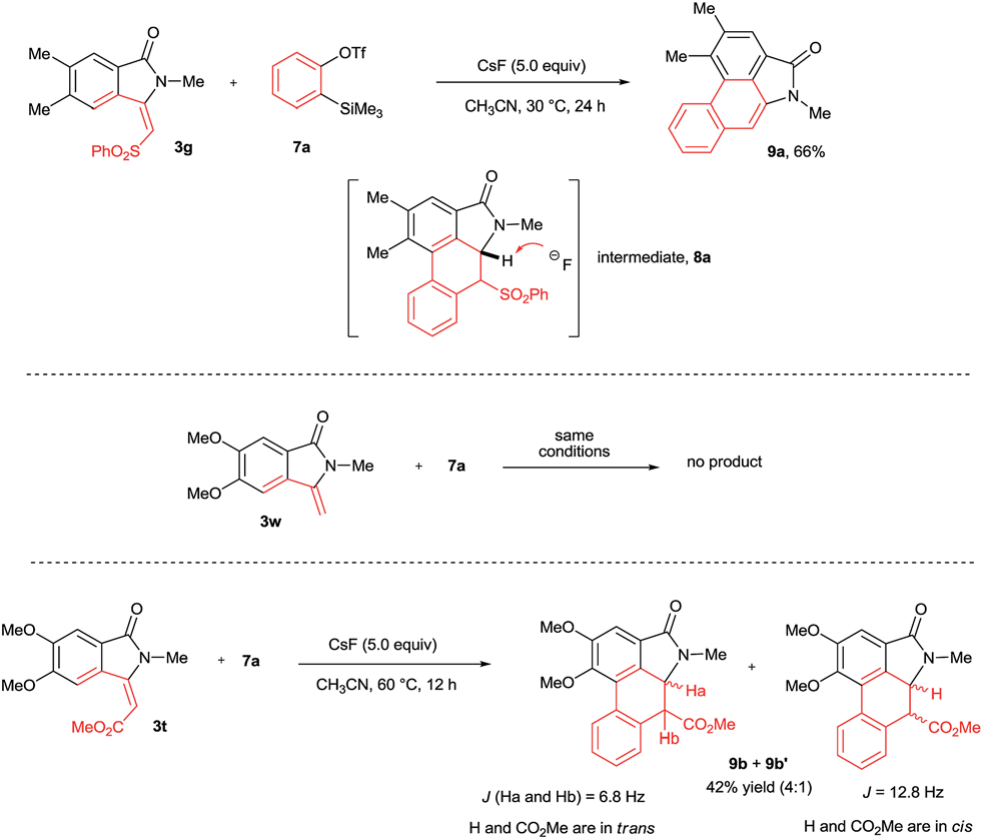
Dehydro-Diels–Alder reaction with benzynes.

To explore the possibility of the preparation of aristolactam derivatives, the dehydro-Diels–Alder reaction of **3** with benzyne was examined (Scheme 5). The cycloaddition of **3g** with benzyne precursor **7a** in the presence of CsF in CH_3_CN at 30 °C for 24 h gave aristolactam derivative **9a** in 66% yield. It is believed that after cycloaddition reaction, intermediate **8a** is formed in which SO_2_Ph is cleaved by a fluoride ion. The formation of intermediate **8a** was confirmed by MALDI-TOF experiment (for the detailed mechanism, see the ESI†).^8^ However, in the cycloaddition reaction of **3w** with **7a**, no product was observed. In the cycloaddition of **3t** in which an ester substituent is present at the β-carbon of the alkene with **7a**, a mixture of heterocyclic molecules **9b** and **9b′** was observed in a 42% combined yield in a 4 : 1 diastereoselective ratio. In the reaction, the CO_2_Me group did not eliminate like SO_2_Ph. This result clearly reveals that the SO_2_Ph group is crucial in order to obtain aristolactams in greater yield with high selectivity.

**Scheme 6:**
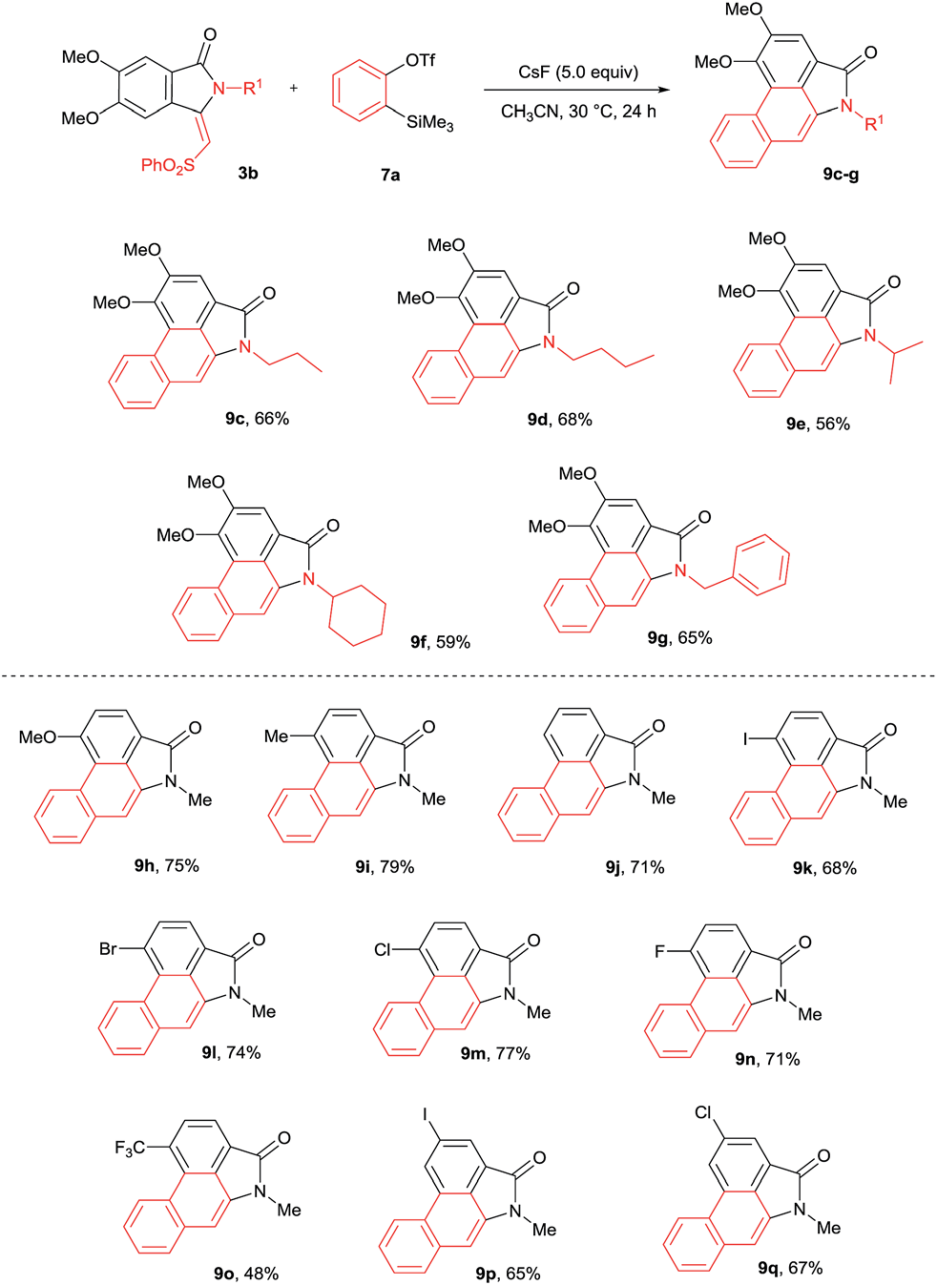
Scope of substituted isoindolin-1-ones.

The cycloaddition reaction was examined with various *N*-substituted indolin-1-one derivatives **3b–f** (Scheme 6). *N*-propyl, butyl, iso-propyl, cyclohexyl and benzyl substituted isoindolin-1-ones **3b–f** underwent cycloaddition with **7a** providing aristolactam derivatives **9c–g** in good yields. Meanwhile, OMe, Me, I, Br, Cl, F and CF_3_ substituted isoindolin-1-ones **3h–s** also efficiently participated in the reaction yielding products **9h–q** in good yields.

**Scheme 7:**
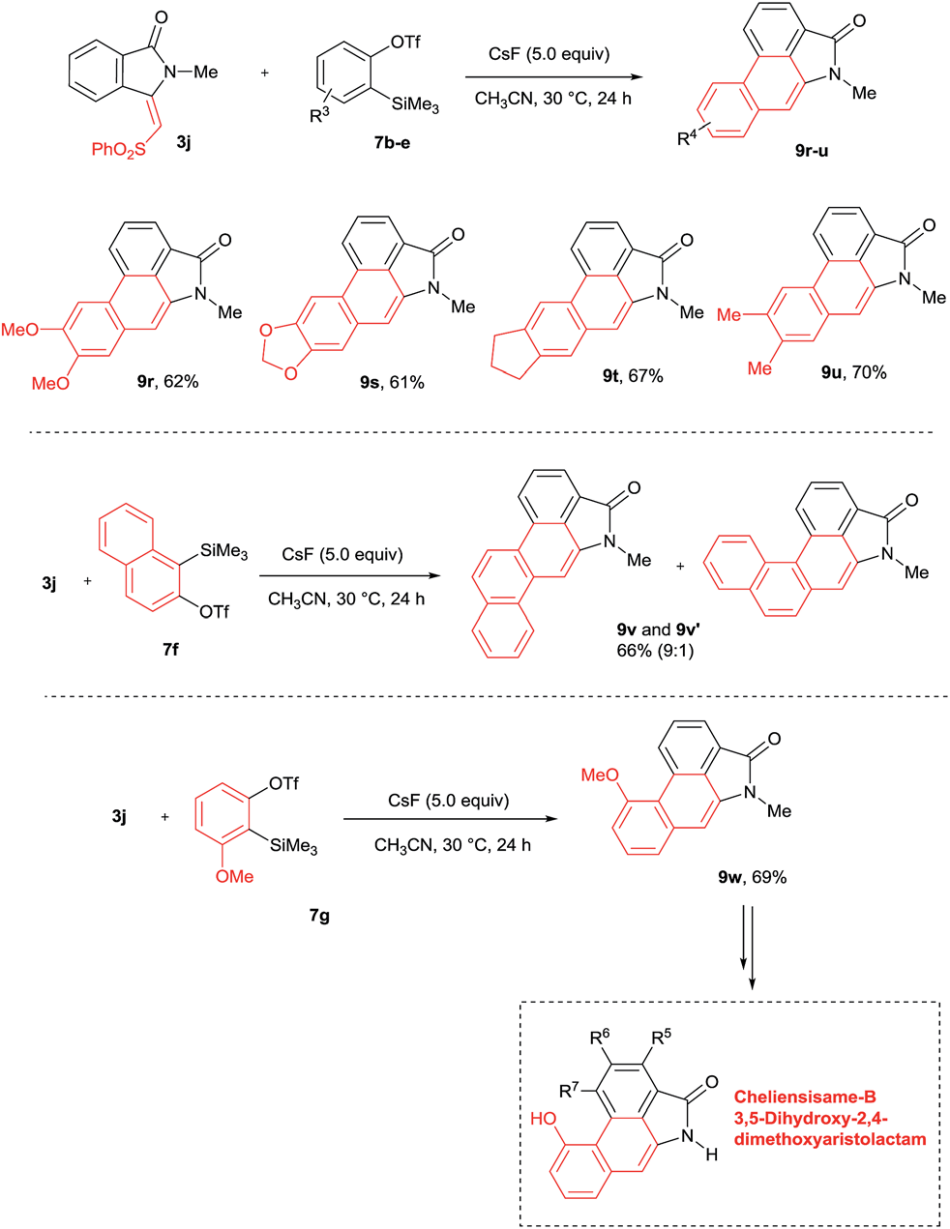
Scope of substituted benzynes.

The scope of the cycloaddition reaction was further examined with substituted benzynes **7b–g** (Scheme 7). Symmetrical benzynes such as 3,4-dimethoxy benzyne, 3,4-dimethyl benzyne, indene derivative and 1,3-benzodioxale reacted with **3j**, providing cyclization products **9r–u** in good yields. When unsymmetrical benzyne **7f** was used, regioisomeric products **9v** and **9v′** were observed in 66% yield in a 9 : 1 ratio. Interestingly, the unsymmetrical benzyne precursor **7g** provided aristolactam **9w** in 69% yield in a highly regioselective manner. The structure of compound **9w** was supported by single crystal X-ray diffraction analysis. It is important to note that by using benzyne precursor **7g**, several natural products can be prepared by changing the substituent on the benzamides.

**Scheme 8:**
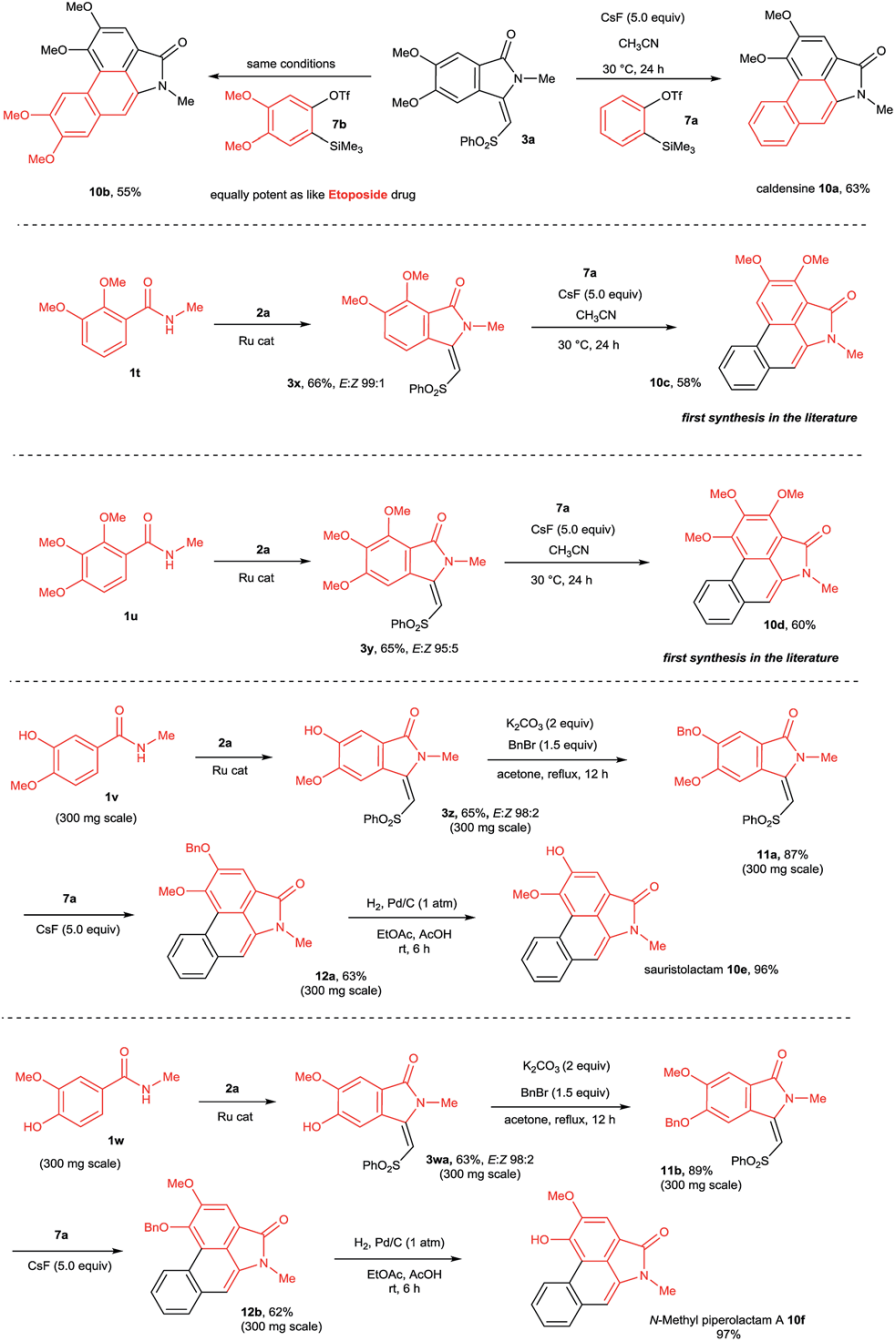
Synthesis of *N*-methyl aristolactam alkaloids.

This result prompted us to explore the possibility of preparing *N*-methyl aristolactam alkaloids (Scheme 8). Treatment of compound **3a** with benzyne precursors **7a** or **7b** in the presence of CsF in CH_3_CN at 30 °C for 24 h gave caldensine **10a** and **10b** in 63% and 55% yields, respectively. Caldensine exhibited an IC_50_ value of 25 mM against chloroquine-sensitive and also showed antiplasmodial activity.3*a* Compound **10b** is equally potent towards multidrug-resistant cell lines compared with the commercially available drug etoposide.2*a* In a similar fashion, other alkaloids such as 2,3-dimethoxy-*N*-methyl-aristolactam **10c** and 2,3,4-trimethoxy-*N*-methyl-aristolactam **10d** were prepared in good yields. It is important to note that the alkaloids **10c–d** were prepared for the first time in the literature. A highly useful sauristolactam (**10e**) and *N*-methyl piperolactam A (**10f**) were prepared in three steps. The reaction of 3-hydroxy-4-methoxy (**1v**) and 3-methoxy-4-hydroxy (**1w**) benzamides with **2a** provided products **3z** and **3wa** in good yields. Later, a free hydroxy group of **3z** and **3wa** was protected with benzyl bromide followed by a cycloaddition reaction with **7a** affording products **12a–b**. Later, the benzyl group was deprotected by a palladium-catalyzed hydrogenation reaction, yielding alkaloids **10e–f** in excellent yields. Sauristolactam (**10e**) and *N*-methyl piperolactam A (**10f**) have shown cytotoxic activity against several cancer cell lines1c,2a and neuroprotective activity.3*b*

**Scheme 9:**
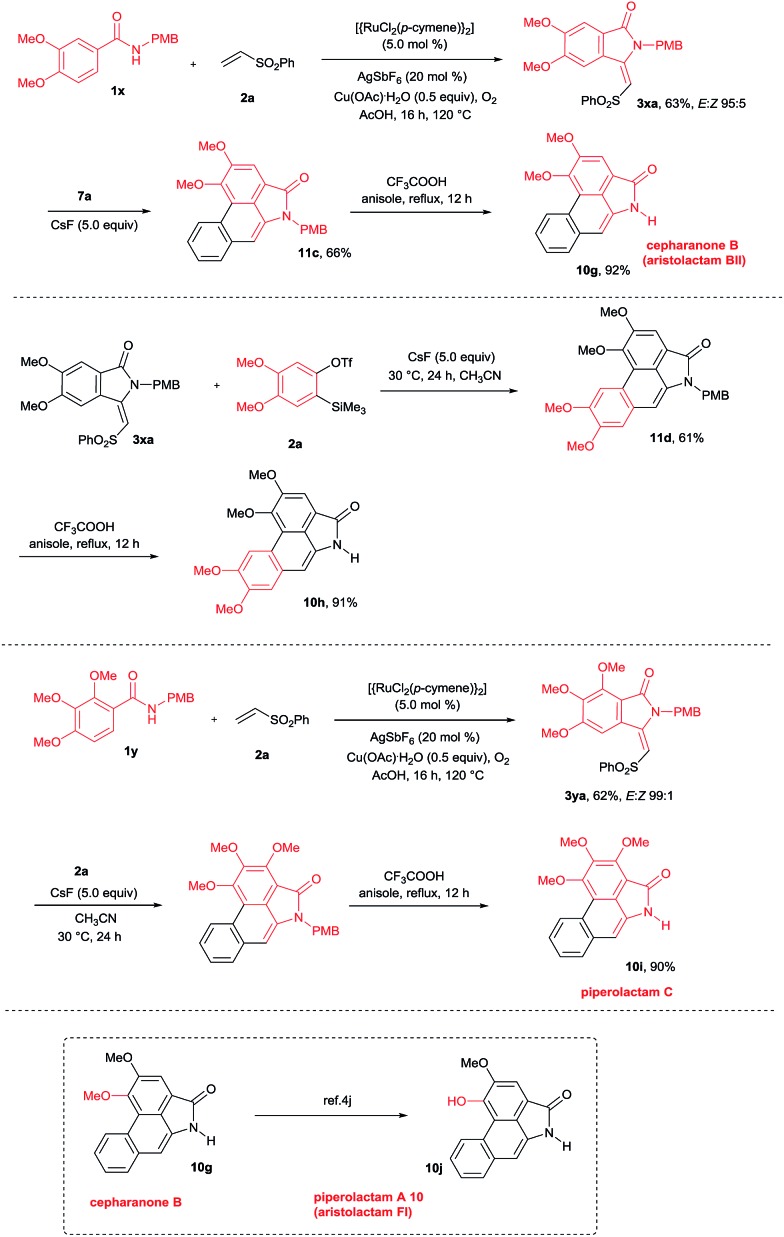
Synthesis of *N*-H aristolactam alkaloids.

By employing the present protocol, *N*-H aristolactams were also prepared by using *N*-PMB substituted benzamides (Scheme 9). The reaction of **1x** with **2a** at 120 °C for 16 h under similar reaction conditions provided product **3xa** in 63% yield. Later, **3xa** was treated with benzyne precursors **7a** or **7b** in the presence of CsF in CH_3_CN at 30 °C for 24 h followed by PMB cleavage yielding cepharanone B (**10g**) and norcepharanone (**10h**) in good yields. In a similar fashion, piperolactam C alkaloid (**10i**) was prepared by the cyclization of **1y** with **2a** in the presence of a ruthenium catalyst followed by cycloaddition with **7a** and subsequent PMB cleavage. Meanwhile, by using cepharanone B (**10g**), aristolactam FI (**10j**) can be prepared easily using a known procedure.4*j* Cepharanone B (**10g**) showed antimalarial activity with IC_50_ values of 7.51–11.01 μg mL^–1^ (ref. 3c) and also exhibited significant cytotoxic activity against human CNS carcinoma cells.3*d* Piperolactam C showed cytotoxicity against P-388 cells with an IC_50_ value of 78 μM.3*e* It is important to note that the *E*/*Z* ratio of indolin-1-one does not affect the yield of the benzyne cycloaddition reaction.

## Conclusions

In conclusion, we have demonstrated an efficient route to synthesize aristolactam alkaloids in good yields using a synergistic combination of C–H bond activation, dehydro-Diels–Alder and desulfonylation reactions. To prepare the target molecules two new synthetic methodologies namely, a ruthenium-catalyzed oxidative cyclization and dehydro-Diels–Alder reaction, were developed. A library of aristolactam derivatives that have substituents on all rings was prepared from easily available starting materials.

## Supplementary Material

Supplementary information

Crystal structure data
