# Supplementary material for: Total synthesis of aristolactam alkaloids via synergistic C–H bond activation and dehydro-Diels–Alder reactions
Source: Chem Sci. 2017 Mar 23;8(5):4130–5. doi: 10.1039/c7sc00161d (PMC6100235; doi:10.1039/c7sc00161d)

Electronic Supplementary Material (ESI) for Chemical Science.
This journal is © The Royal Society of Chemistry 2017

Total Synthesis of Aristolactam Alkaloids via Synergistic C-H Bond Activation and Dehydro-Diels-Alder Reactions

*Mallu Chenna Reddy and Masilamani Jeganmohan**

Department of Chemistry, Indian Institute of Science Education and Research, Pune 411008, India

Email: mjeganmohan@iiserpune.ac.in

Electronic Supplementary Information (ESI)

Table of Contents

Pages S2 – S5 Experimental Section

Page S6 – S9 Mechanistic Discussion

Pages S10 – S49 Spectral Data of all Compounds including NOESY Studies

Page S50 Reference

Pages S51 – S119 Copies of ^{1}H and ^{13}C NMR Spectra of All Compounds

Experimental Section

General Information

Yields refer to isolated yields of compounds estimated to be > 95 % pure as determined by ^{1}H-NMR (25 °C). NMR spectra were recorded on solutions in deuterated chloroform ($CDCl_3$) with residual chloroform (δ 7.24 ppm for ^{1}H NMR and δ 77.16 ppm for ^{13}C NMR). Column chromatographically purifications were performed using SiO_2 (130–150 mesh ASTM) from Merck if not indicated otherwise. [{$RuCl_2$(*p*-cymene)}$_2$], $AgSbF_6$, $Cu(OAc)_2 \cdot H_2O$, Pd/C, aromatic acids, alkenes, and other reagents or chemicals were used as purchased without further purification. Dry AcOH, dry CH_3CN, dry acetone and dry ethyl acetate were used for the recation. Starting materials substituted benzamides **1** were synthesized according to the literature procedures.[1]

General Procedure for the Cyclization of Aromatic Amides with Alkenes Catalyzed by Ruthenium Complex (Procedure for solid benzamides).

In a Schlenk tube (3cm width, 18cm hight) with a magnetic stirrer and septum containing [{$RuCl_2$(*p*-cymene)}$_2$] (5.0 mol %), $Cu(OAc)_2 \cdot H_2O$ (0.50 equiv, 50 mol %), $AgSbF_6$ (20 mol %) and benzamide **1** (75 mg, if solid) was evacuated and purged with nitrogen gas three times ($AgSbF_6$ was taken inside the glove box). To the tube, were then added alkene **2** (**2a** (1.5 equiv) or **2b-c** (4.00 equiv) or **2d-e** (2.0 equiv)) in dry acetic acid solvent (3.0 mL) via syringes. Then, the reaction was evacuated and purged with nitrogen gas three times and stir at room temperature for 5 min (during this type a cationic ruthenium complex was formed). Later, the tube was evacuated and purged with the oxygen gas three times. Then, the reaction mixture was allowed to stir at 120 °C for 16-72 h (the exact reaction time is mentioned in the spectral data information) under oxygen atmosphere which was taken in a balloon. After cooling to ambient temperature, the reaction mixture was diluted with CH_2Cl_2, filtered through Celite pad, and the filtrate was concentrated. The crude residue was purified through a silica gel column using hexanes and ethyl acetate as eluent to give pure **3**.

Note: The reaction of **3z** or **3wa** with **2a** was done in a 300 mg scale. In the reaction, 12 mL AcOH solvent was used. After reaction, the reaction mixture was diluted with CH_2Cl_2 and MeOH solvents, filtered through Celite pad, and the filtrate was concentrated.

General Procedure for the Cyclization of Aromatic Amides with Alkenes Catalyzed by Ruthenium Complex (Procedure for semisolid/liquid benzamides).

In a Schlenk tube (3cm width, 18cm hight) with a magnetic stirrer and septum containing [{$RuCl_2$(*p*-cymene)}$_2$] (5.0 mol %), $Cu(OAc)_2 \cdot H_2O$ (0.50 equiv, 50 mol %) and $AgSbF_6$ (20 mol %) was evacuated and purged with nitrogen gas three times ($AgSbF_6$ was taken inside the glove box). To the tube, were then added benzamide **1** (75 mg, if semisolid or liquid) and **2** (**2a** (1.5 equiv) or **2b-c** (4.00 equiv) or **2d-e** (2.0 equiv)) in dry acetic acid solvent (3.0 mL) via syringes. Then, the reaction was evacuated and purged with nitrogen gas three times and stir at room temperature for 5 min (during this type a cationic ruthenium complex was formed). Later, the tube was evacuated and purged with the oxygen gas three times. Then, the reaction mixture was allowed to stir at 120 °C for 16-72 h (the exact reaction time is mentioned in the spectral data information) under oxygen atmosphere which was taken in a balloon. After cooling to ambient temperature, the reaction mixture was diluted with CH_2Cl_2, filtered through Celite pad, and the filtrate was concentrated. The crude residue was purified through a silica gel column using hexanes and ethyl acetate as eluent to give pure **3**.

Note: The reaction of **3z** or **3wa** with **2a** was done in a 300 mg scale. In the reaction, 12 mL AcOH solvent was used. After reaction, the reaction mixture was diluted with CH_2Cl_2 and MeOH solvents, filtered through Celite pad, and the filtrate was concentrated.

General Procedure for the Cycloaddition reaction.

In a single neck tube (5cm width, 20cm hight) with a medium size magnetic stirrer and septum containing compound **3** (80 mg) in dry CH_3CN solvent (7.0 mL) was evacuated and purged with nitrogen gas three times. Later, benzyne precursor **7** (2.0 equiv) in 1.0 mL of dry CH_3CN was added into the tube via syringes. After stirring at room temperature for 3 min, CsF (5.0 equiv) was added into the tube and the reaction was evacuated and purged with nitrogen gas three times (CsF was taken inside the glove box). Then, the reaction mixture was allowed to stir at 30 °C for 24 h. Later, the reaction mixture was diluted with CH_2Cl_2, filtered through Celite pad, and the

filtrate was concentrated. The crude residue was purified through a silica gel column using hexanes and ethyl acetate as eluent to give pure **9**.

General Procedure for the Benzylation Reaction (preparation of 11a-b).

In a single neck 25-mL round bottom flask with a magnetic stirrer and septum containing compound **3z** or **3wa** (300 mg) and K_2CO_3 (2.0 equiv) in dry acetone solvent (15.0 mL) was evacuated and purged with nitrogen gas three times. Then, benzyl bromide (1.5 equiv) in a 5.0 mL acetone was added via syringe. Immediately, a spectrum was taken out and fitted with a condenser and the top of condenser was covered with a septum. Again, the reaction mixture was evacuated and purged with nitrogen gas three times. Then, the reaction mixture was refluxed under the nitrogen atmosphere which was taken in a balloon for 6 h. Later, the reaction mixture was diluted with DCM, filtered through Celite pad, and the filtrate was concentrated. The crude residue was purified through a silica gel column using hexanes and ethyl acetate as eluent to give pure **11a-b**.

General Procedure for the Cleavage of Benzyl Group (preparation of 12a-b).

In a single neck 25-mL round bottom flask with a magnetic stirrer and septum containing compound **11a** or **11b** (300 mg) with a mixture of dry ethyl acetate solvent (15.0 mL) and dry acetic acid (0.4 mL) was evacuated and purged with nitrogen gas three times. The reaction mixture was allowed to stir at room temperature for 10 min (to make uniform solubility). Later, Pd/C (45 mg, 10% w/w) was added into the round bottom flask. Again, the reaction was evacuated and purged with nitrogen gas three times. Then, the reaction mixture was allowed to stir at 25 °C under a hydrogen atmosphere which was taken in a balloon for 6 h. Later, the reaction mixture was diluted with MeOH, filtered through Celite pad, and the filtrate was concentrated. The crude residue was purified through a silica gel column using hexanes and ethyl acetate as eluent to give pure **10e** or **10f**.

General Procedure for the PMB Group Cleavage (preparation of 10h-i).

In a single neck 50-mL round bottom flask with a magnetic stirrer and septum containing compound **11c-e** (100 mg) and anisole (20 equiv) in trifluoroacetic acid solvent (10.0 mL) was evacuated and purged with nitrogen gas three times. Immediately, a spectrum was taken out and

fitted with a condenser and the top of condenser was covered with a septum. Again, the reaction mixture was evacuated and purged with nitrogen gas three times. Then, the reaction mixture was allowed to reflux for 12 h under the nitrogen atmosphere. Later, the reaction mixture was diluted with MeOH and DCM, filtered through Celite pad, and the filtrate was concentrated. The crude residue was purified through a silica gel column using hexanes and ethyl acetate as eluent to give pure **10h-i**.

Mechanistic Discussion

Scheme S1 Proposed Mechanism for Oxidative Cyclization of Benzamides with Alkenes

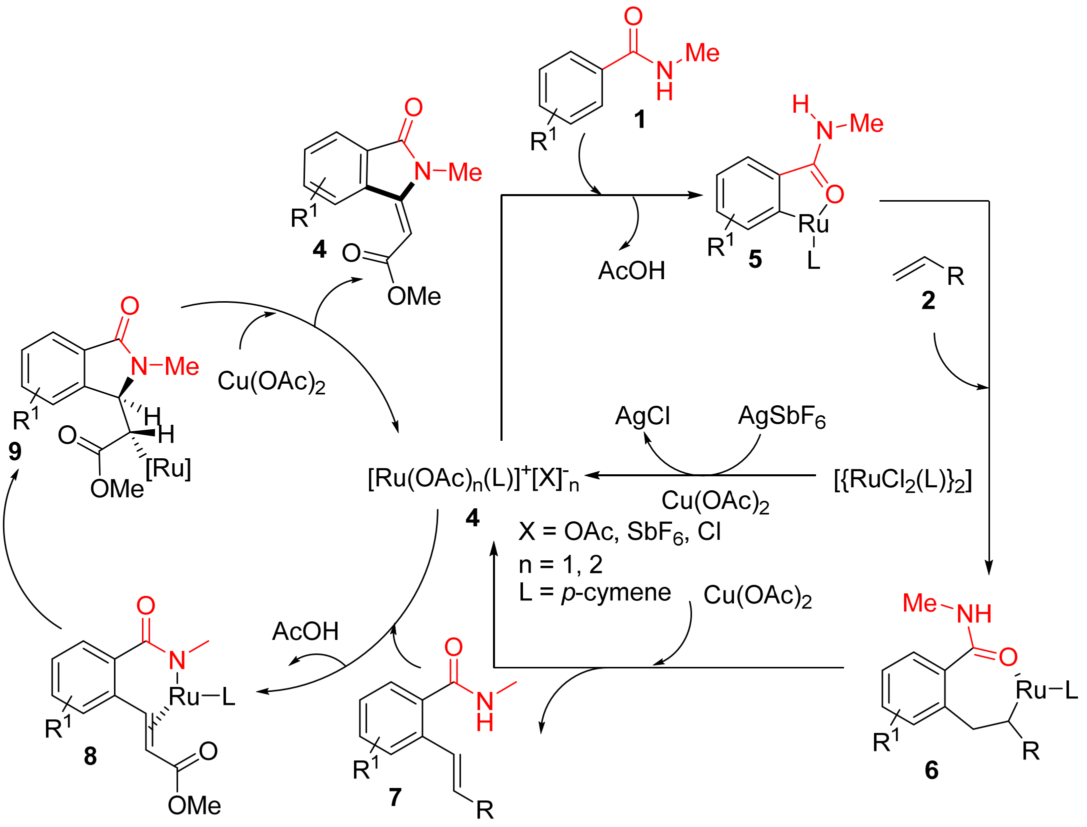

$$CuOAc + AcOH \xrightarrow{O_2} Cu(OAc)_2$$

A possible reaction mechanism for oxidative cyclization of *N*-substituted benzamides **1** with alkenes **2** is discussed in Scheme S1. $AgSbF_6$ likely removes the chloride ligand from [{$RuCl_2$(*p*-cymene)}$_2$] complex followed by the ligand exchange with $Cu(OAc)_2$ giving a cationic ruthenium species **4**. Coordination of the carbonyl group of amide **1** into the ruthenium cationic species followed by *ortho*-metalation provides a five-membered ruthenacycle **5** and AcOH. Coordinative insertion of alkene **2** into the Ru–carbon bond of ruthenacycle **5** affords a seven membered ruthenacycle intermediate **6**. β-Hydride elimination of intermediate **6** in the presence of $Cu(OAc)_2$ provides a Heck-type alkenylated product **7** and regenerates the active ruthenium species **4** for the next catalytic cycle.

Later, the amide group of product **7** coordinates with a ruthenium species **4** followed by the intramolecular coordination of double bond affords intermediate **8** and AcOH. Intramolecular coordinative insertion of N-Ru bond of intermediate **8** into an alkene moiety provides intermediate **9.** Subsequent β-hydride elimination of intermediate **9** in the presence of $Cu(OAc)_2$ provides product **3** and regenerates the active ruthenium species **4** for the next catalytic cycle.

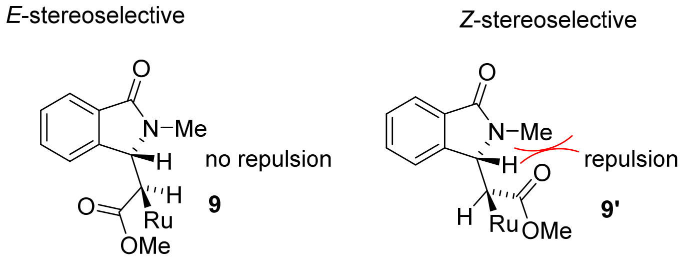

(S1)

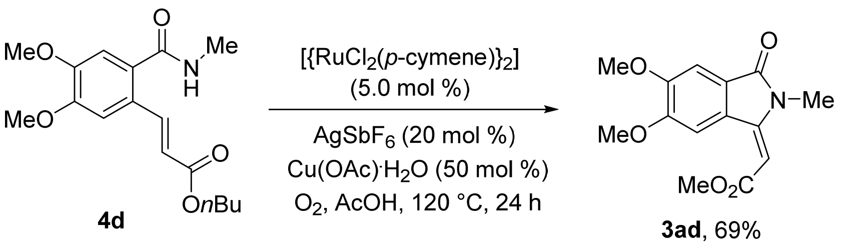

(S2)

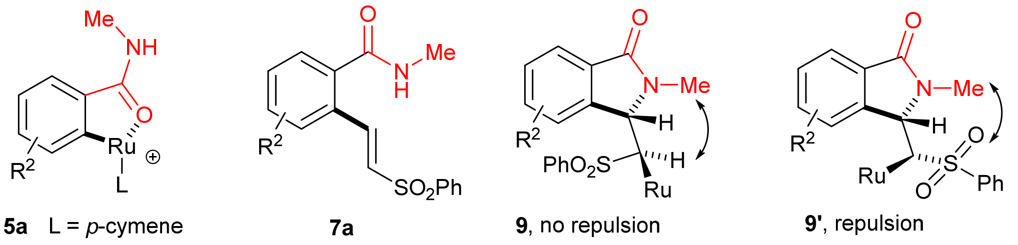

(3)

The stereoselectivity highly depends on the coordinative intramolecular insertion of N-Ru bond to the double of alkene in intermediate **8**. In the reaction, intermediate **9** was observed (eq S1). After that, β-Hydride elimination takes place of intermediate **9** in the presence of $Cu(OAc)_2$ providing *E*-stereoselective product **3** and regenerates the active ruthenium species **4**. In the intermediate **9**, an ester group of alkene and tertiary C-N Me of intermediate **9** stay anti to each other. This is mainly due to avoid the steric hindrance of *N*-Me group and ester moiety of intermediate **9'**. Meanwhile, intermediate **9'** accounts for the Z-stereoselective product.

To support the proposed mechanism in Scheme S1, *ortho*-alkenylated *N*-methyl benzamide **4d** was prepared separately and treated with [{$RuCl_2$(*p*-cymene)}$_2$] (5.0 mol %), AgSbF6 (20 mol

%) and $Cu(OAc)_2 \cdot H_2O$ (50 mol %) in acetic acid (AcOH) under oxygen at 120 °C for 24 h. In the reaction, the expected cyclization product **3v** was observed in 69% yield in a highly *E* stereoselective manner (eq S2).

In the reaction, only 30 mol % of $Cu(OAc)_2$ was used. In fact, a stoichiometric amount of 2.0 equiv $Cu(OAc)_2$ is needed for the reaction. The remaining amount of $Cu(OAc)_2$ has been regenerated by the reaction of CuOAc with AcOH under oxygen.

It has been previously observed from our group that a cationic ruthenium species **4** is very selective to the coordination of carbonyl group instead of the nitrogen group in benzamide **1** (please see: ACS Catal. **2016**, *6*, 230).

Scheme S2. Proposed Mechanism for Cycloaddition Reaction

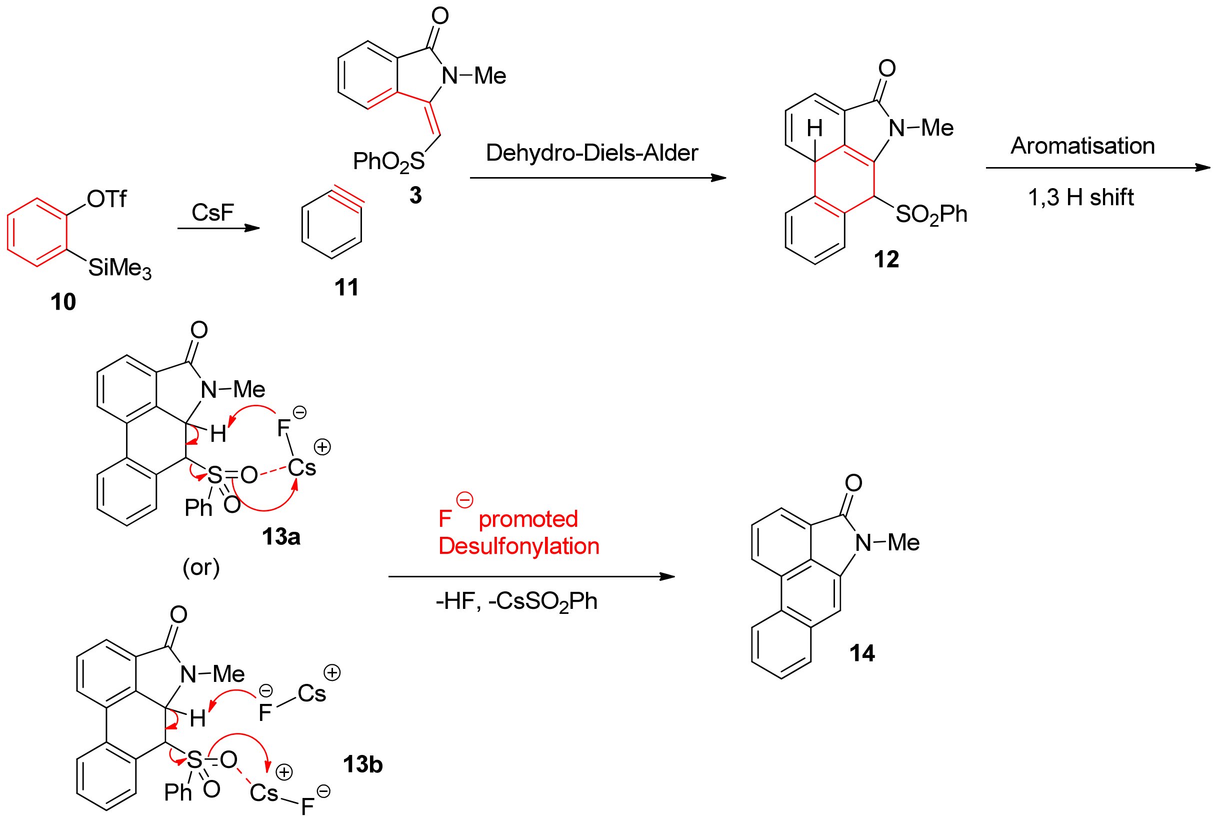

A possible reaction mechanism is proposed in Scheme S2 for the cycloaddition of product **3** with benzyne **11**. Isoindolone molecule **3** acts as a dienophile which undergoes dehydro-Diels-Alder

reaction with benzyne **11** giving intermediate **12**. CsF likely cleaves $SiMe_3$ of **10** followed by elimination of OTf generating benzyne **11**. Intermediate **12** undergoes 1,3-H shift due to aromatization as a driving force giving intermediate **13a** or **13b**. Later, fluoride ion abstracts the hydrogen which is adjacent to SO_2Ph carbon and promoted desulfonation either in inter- or intramolecular fashion providing the final cycloaddition product **14** (Sikervar, V.; Fleet, J. C.; Fuchs. P. L. *J. Org. Chem.* **2012**, *77*, 5132). It is important to note that the formation of key intermediate **13** by MALDI-TOF experiment (Li, L. Liu, Y.; Peng, Y.; Yu, L.; Wu, X.; Yan, H. *Angew. Chem. Int. Ed.* **2015**, *54*, 1). According to Woodward rules of [4+2] cycloaddition, there are four intermediates possible which is given below (eq S3).

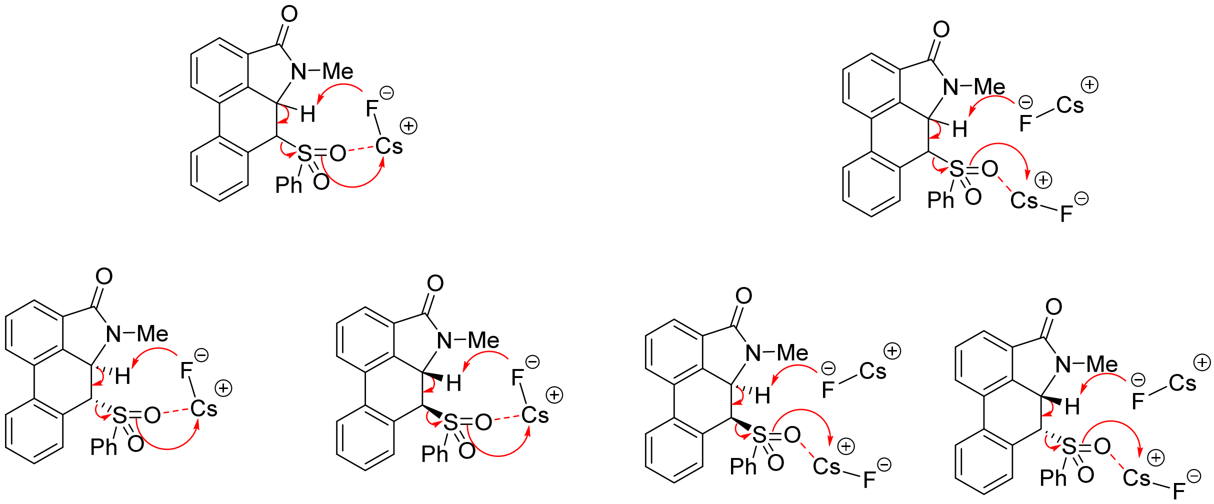

(S3)

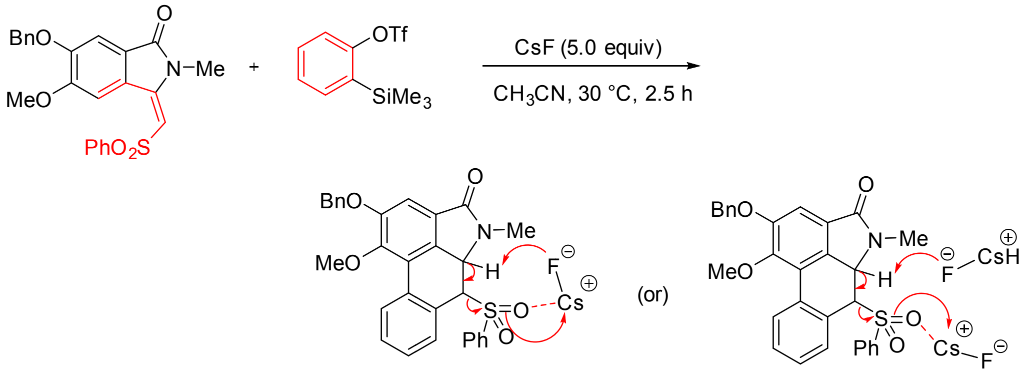

Exact Mass: 663.0492 Exact Mass: 663.0492

calc. for [($C_{30}H_{25}CsFNO_5S$)H] (M+H): 664.06 , found 664.06 .

Copy of MALDI-TOF Experimient

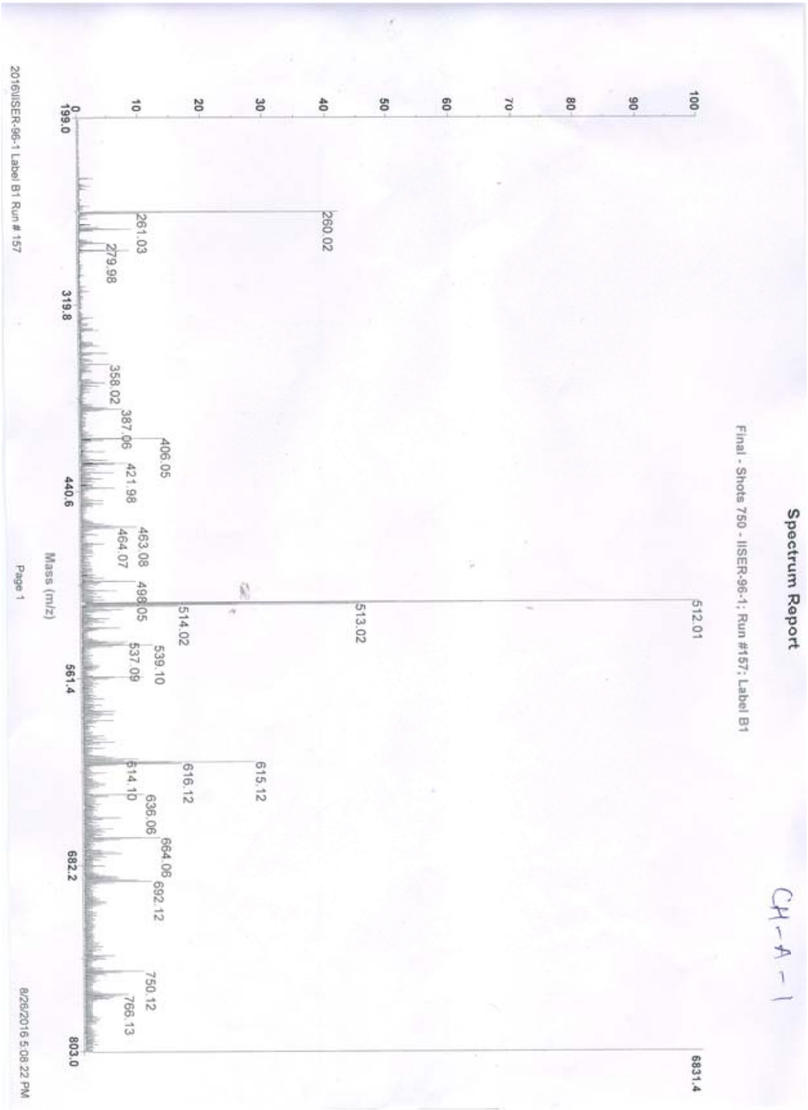

Spectral Data

(*E*)-5,6-Dimethoxy-2-methyl-3-((phenylsulfonyl)methylene)isoindolin-1-one (3a).

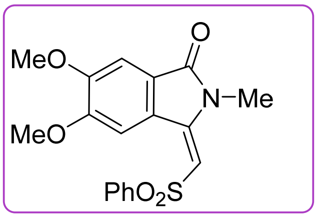

Yellow semi solid; eluent (35% ethyl acetate in hexanes). The reaction scale 75 mg (**1a** (75 mg), **2a** (1.5 equiv)), 108 mg of **3a** was isolated and yield is 78%. The reaction was done for 36 h at 120 °C.

^{1}H NMR ($CDCl_3$, 400 MHz): δ 8.37 (s, 1 H), 7.99 - 7.97 (m, 2 H), 7.62 - 7.50 (m, 3 H), 7.24 (s, 1 H), 6.02 (s, 1 H), 3.97 (s, 3 H), 3.94 (s, 3 H), 3.14 (s, 3 H).

^{13}C NMR ($CDCl_3$, 100 MHz): δ 166.9, 152.9, 152.3, 147.7, 142.7, 133.6, 129.5, 127.1, 126.7, 125.7, 123.7, 110.5, 106.5, 105.3, 56.6, 56.5, 26.5 (One carbon signal is missing due to overlap).

HRMS (ESI): calc. for [($C_{18}H_{17}NO_5S$)H] (M+H) 360.0906, measured 360.0898.

IR (ATR)$\tilde{\nu}$ (cm^{-1}): 3059, 2927,2850, 1723, 1596, 1466,1306, 1222, 1145, 1082, 1038, 856, 760.

(*E*)-5,6-Dimethoxy-3-((phenylsulfonyl)methylene)-2-propylisoindolin-1-one (3b).

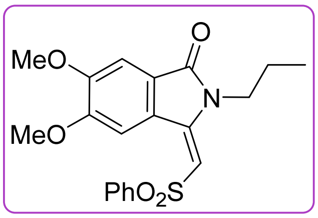

Light yellow semisolid; eluent (30% ethyl acetate in hexanes). The reaction scale is 75 mg (**1b** (75 mg), **2a** (1.5 equiv)), 90 mg of **3b** was isolated and yield is 69 %. The reaction was done for 36 h at 120 °C .

^{1}H NMR ($CDCl_3$, 400 MHz): δ 8.33 (s, 1 H), 7.99 - 7.96 (m, 2 H), 7.62 - 7.51 (m, 3 H), 7.25 (s, 1 H), 6.07 (s, 1 H), 3.96 (s, 3 H), 3.94 (s, 3 H), 3.62 (t, *J* = 7.2 Hz, 2 H), 1.63 – 1.55 (m, 2 H), 0.91 (t, *J* = 7.2 Hz, 3 H).

^{13}C NMR ($CDCl_3$, 100 MHz): δ 167.2, 152.9, 152.5, 147.0, 143.1, 133.5, 129.5, 126.6, 125.8, 123.6, 110.7, 106.2, 105.4, 56.6, 56.5, 41.4, 21.4, 11.4 (Two carbon signals are missing due to overlap).

HRMS (ESI): calc. for [($C_{20}H_{21}NO_5S$)H] (M+H) 388.1219, measured 388.1222.

IR (ATR)$\tilde{\nu}$ (cm^{-1}): 3059, 2964, 2839, 1722, 1591, 1499, 1463, 1303, 1051, 856, 721.

(*E*)-2-Butyl-5,6-dimethoxy-3-((phenylsulfonyl)methylene)isoindolin-1-one (3c).

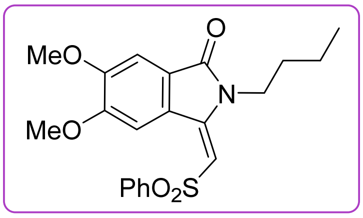

Off-white semisolid; eluent (30% ethyl acetate in hexanes). The reaction scale is 75 mg (**1c** (75 mg), **2a** (1.5 equiv)), 85 mg of **3c** was isolated and yield is 67 %. The reaction was done for 36 h at 120 °C.

^{1}H NMR ($CDCl_3$, 400 MHz): δ 8.33 (s, 1 H), 7.99 - 7.97 (m, 2 H), 7.62 - 7.58 (m, 1 H), 7.54 - 7.50 (m, 2 H), 7.24 (s, 1 H), 6.07 (s, 1 H), 3.96 (s, 3 H), 3.94 (s, 3 H), 3.65 (t, *J* = 7.2 Hz, 2 H), 1.57 – 1.50 (m, 2 H), 1.35 – 1.26 (m, 2 H), 0.89 (t, *J* = 7.2 Hz, 3 H).

^{13}C NMR ($CDCl_3$, 100 MHz): δ 167.1, 152.9, 152.4, 146.9, 143.0, 133.5, 129.5, 126.5, 125.8, 123.6, 110.6, 106.2, 105.3, 56.6, 56.5, 39.6, 30.1, 20.2, 13.8 (Two carbon signals are missing due to overlap).

HRMS (ESI): calc. for [($C_{21}H_{23}NO_5S$)H] (M+H) 402.1375, measured 402.1379.

IR (ATR)$\tilde{\nu}$ (cm^{-1}): 3090, 2957, 1719, 1590, 1497, 1463,1393, 1302, 1180, 1039, 994, 854, 753.

(*E*)-2-Isopropyl-5,6-dimethoxy-3-((phenylsulfonyl)methylene)isoindolin-1-one (3d).

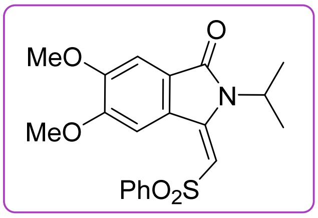

Brown semisolid; eluent (30% ethyl acetate in hexanes). The reaction scale is 75 mg (**1d** (75 mg), **2a** (1.5 equiv)), 76 mg of **3d** was isolated and yield is 58 %. The reaction was done for 36 h at 120 °C.

^{1}H NMR ($CDCl_3$, 400 MHz): δ 8.31 (s, 1 H), 8.03 - 8.01 (m, 2 H), 7.66 - 7.55 (m, 3 H), 7.27 (s, 1 H), 6.26 (s, 1 H), 4.45 - 4.38 (m, 1 H), 3.98 (s, 3 H), 3.97 (s, 3 H), 1.51 (s, 3 H), 1.49 (s, 3 H) .

^{13}C NMR ($CDCl_3$, 100 MHz): 167.4, 152.9, 152.4, 146.8, 143.1, 133.5, 129.5, 126.5, 125.8, 123.8, 110.5, 106.7, 105.1, 56.6, 56.5, 44.9, 20.1 (Three carbon signals are missing due to overlap).

HRMS (ESI): calc. for [($C_{20}H_{21}NO_5S$)H] (M+H) 388.1219, measured 388.1218.

IR (ATR)ṽ (cm^{-1}): 3060, 2929, 2848, 1722,1590, 1500, 1461, 1305, 1143, 691.

(*E*)-2-Cyclohexyl-5,6-dimethoxy-3-((phenylsulfonyl)methylene)isoindolin-1-one (3e).

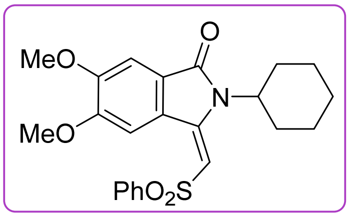

Off-white semisolid; eluent (30% ethyl acetate in hexanes). The reaction scale is 75 mg (**1e** (75 mg), **2a** (1.5 equiv)), 69 mg of **3e** was isolated and yield is 56 %. The reaction was done for 36 h at 120 °C.

^{1}H NMR ($CDCl_3$, 400 MHz): δ 8.22 (s, 1 H), 7.99 (d, *J* = 7.6 Hz, 2 H), 7.62 - 7.51 (m, 3 H), 7.19 (s, 1 H), 6.27 (s, 1 H), 3.93 (s, 3 H), 3.92 (s, 3 H), 3.85 - 3.74 (m, 1 H), 2.25 - 2.16 (m, 2 H), 1.88 - 1.67 (m, 5 H), 1.38 - 1.17 (m, 3 H).

^{13}C NMR ($CDCl_3$, 100 MHz): δ 167.4, 152.8, 152.4, 147.5, 143.1, 133.5, 129.5, 126.4, 125.6, 123.8, 110.4, 106.2, 104.9, 56.6, 56.5, 29.8, 26.4, 25.3 (Five carbon signals are missing due to overlap).

HRMS (ESI): calc. for [($C_{23}H_{25}NO_5S$)H] (M+H) 428.1532, measured 428.1537.

IR (ATR)ṽ (cm^{-1}): 3302, 2930,2855, 1719, 1587, 1499, 1464, 1304, 1080, 753.

(*E*)-2-Benzyl-5,6-dimethoxy-3-((phenylsulfonyl)methylene)isoindolin-1-one (3f).

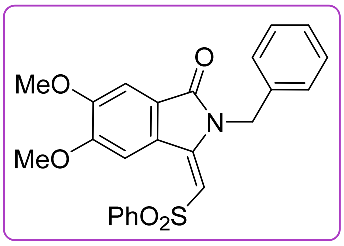

Yellow semisolid; eluent (30% ethyl acetate in hexanes). The reaction scale is 75 mg (**1f** (75 mg), **2a** (1.5 equiv)), 86 mg of **3f** was isolated and yield is 71 %. The reaction was done for 36 h at 120 °C.

^{1}H NMR ($CDCl_3$, 400 MHz): δ 8.34 (s, 1 H), 7.72 (d, *J* = 7.2 Hz, 2 H), 7.54 (t, *J* = 7.2 Hz, 1 H), 7.42 (t, *J* = 7.6 Hz, 2 H), 7.33 (s, 1 H), 7.28 - 7.24 (m, 3 H), 7.12 - 7.10 (m, 2 H), 6.02 (s, 1 H), 4.92 (s, 2 H), 3.98 (s, 3 H), 3.97 (s, 3 H).

^{13}C NMR (CDCl$_3$, 100 MHz): δ 167.2, 153.1, 152.4, 146.1, 142.8, 135.4, 133.3, 129.2, 129.0, 127.9, 126.8, 126.3, 125.9, 123.3, 110.6, 108.0, 105.5, 56.6, 56.5, 43.5 (Four carbon signals are missing due to overlap).

HRMS (ESI): calc. for [($C_{24}H_{21}NO_5S$)H] (M+H) 436.1219, measured 436.1222.

IR (ATR)$\tilde{\nu}$ (cm^{-1}): 3061, 2936, 2836, 1723, 1592, 1498, 1305, 1142, 1079, 854, 732.

(*E*)-2,5,6-Trimethyl-3-((phenylsulfonyl)methylene)isoindolin-1-one (3g).

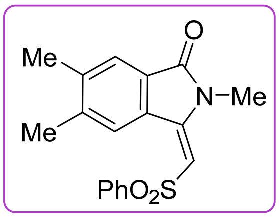

Colorless semisolid; eluent (25% ethyl acetate in hexanes). The reaction scale is 75 mg (**1g** (75 mg), **2a** (1.5 equiv)), 99 mg of **3g** was isolated and yield is 66%. The reaction was done for 72 h at 120 °C.

^{1}H NMR (CDCl$_3$, 400 MHz): δ 8.52 (s, 1 H), 8.00 (d, *J* = 7.2 Hz, 2 H), 7.61 - 7.50 (m, 4 H), 6.04 (s, 1 H), 3.14 (s, 3 H), 2.37 (s, 3 H), 2.33 (s, 3 H).

^{13}C NMR (CDCl$_3$, 100 MHz): δ 167.1, 147.8, 142.9, 142.7, 141.6, 133.5, 130.1, 129.4, 128.8, 128.1, 126.9, 124.5, 106.9, 26.4, 20.9, 20.4 (Two carbon signals are missing due to overlap).

HRMS (ESI): calc. for [($C_{18}H_{17}NO_3S$)H] (M+H) 328.1007, measured 328.1013.

IR (ATR)$\tilde{\nu}$ (cm^{-1}): 3052, 2925, 1724, 1599, 1431, 1389, 1311, 1145, 1020, 757, 723.

(*E*)-5-Methoxy-2-methyl-3-((phenylsulfonyl)methylene)isoindolin-1-one (3h).

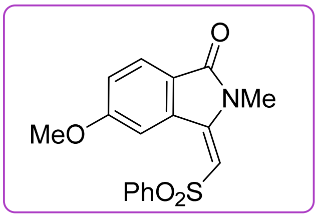

Colorless semisolid; eluent (30% ethyl acetate in hexanes). The reaction scale is 75 mg (**1h** (75 mg), **2a** (1.5 equiv)), 113 mg of **3h** was isolated and yield is 75 %. The reaction was done for 72 h at 120 °C.

^{1}H NMR (CDCl$_3$, 400 MHz): δ 8.38 (d, *J* = 2.4 Hz, 1 H), 8.01 - 7.99 (m, 2 H), 7.70 (d, *J* = 7.6 Hz, 1 H), 7.63 - 7.59 (m, 1 H), 7.55 - 7.52 (m, 2 H), 7.05 (dd, *J* = 7.6, 2.4 Hz, 1 H), 6.07 (s, 1 H), 3.90 (s, 3 H), 3.14 (s, 3 H).

^{13}C NMR ($CDCl_3$, 100 MHz):δ 166.6, 164.0, 147.4, 142.6, 134.4, 133.6, 129.5, 126.8, 125.0, 122.4, 118.4, 112.9, 107.6, 56.1, 26.5 (Two carbon signals are missing due to overlap).

HRMS (ESI): calc. for [($C_{17}H_{15}NO_4S$)H] (M+H) 330.0800, measured 330.0796.

IR (ATR)$\tilde{\nu}$ (cm^{-1}): 2932, 2850, 1727, 1600, 1480, 1434, 1383, 1320, 1291, 1147, 1081, 1040, 841, 761.

(*E*)-2,5-Dimethyl-3-((phenylsulfonyl)methylene)isoindolin-1-one (3i).

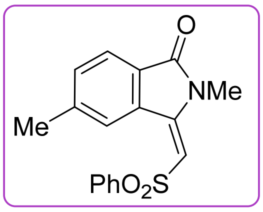

Colorless semisolid; eluent (20% ethyl acetate in hexanes). The reaction scale is 75 mg (**1i** (75 mg), **2a** (1.5 equiv)), 112 mg of **3i** was isolated and yield is 71 %. The reaction was done for 72 h at 120 °C.

^{1}H NMR ($CDCl_3$, 400 MHz): δ 8.58 (s, 1 H), 8.02 – 7.99 (m, 2 H), 7.68 (d, *J* = 8.0 Hz, 1 H), 7.62 – 7.51 (m, 3 H), 7.37 (d, *J* = 8.0 Hz, 1 H), 6.08 (s, 1 H), 3.15 (s, 3 H), 2.48 (s, 3 H).

^{13}C NMR ($CDCl_3$, 100 MHz): δ 166.8, 147.5, 144.3, 142.6, 133.6, 132.7, 132.5, 129.4, 128.5, 127.6, 126.9, 123.5, 107.8, 26.4, 22.4 (Two carbon signals are missing due to overlap) .

HRMS (ESI): calc. for [($C_{17}H_{15}NO_3S$)H] (M+H) 314.0851, measured 314.0857.

IR (ATR)$\tilde{\nu}$ (cm^{-1}): 3063, 2312, 1734, 1609, 1435, 1323, 1287, 1144, 1091, 1050, 910, 827, 761.

(*E*)-2-Methyl-3-((phenylsulfonyl)methylene)isoindolin-1-one (3j).

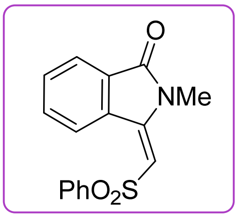

Colorless semisolid ; eluent (25% ethyl acetate in hexanes). The reaction scale is 75 mg (**1j** (75 mg), **2a** (1.5 equiv)), 108 mg of **3j** was isolated and yield is 65 %. The reaction was done for 72 h at 120 °C.

^{1}H NMR ($CDCl_3$, 400 MHz): δ 8.83 (d, *J* = 7.6 Hz, 1 H), 8.02 - 8.00 (m, 2 H), 7.81 (d, *J* = 7.6 Hz, 1 H), 7.68 - 7.58 (m, 3 H), 7.56 - 7.51 (m, 2 H), 6.12 (s, 1 H), 3.17 (s, 3 H).

^{13}**C NMR ($CDCl_3$, 100 MHz)**:δ 166.8, 147.3, 142.5, 133.7, 133.4, 132.1, 131.9, 130.1, 129.5, 127.9, 126.9, 123.7, 108.2, 26.5 (Two carbon signals are missing due to overlap).

HRMS (ESI): calc. for [($C_{16}H_{13}NO_3S$)H] (M+H) 300.0694, measured 300.0703.

IR (ATR)$\tilde{\nu}$ (cm^{-1}): 3058, 2922, 1726, 1606, 1436, 1391, 1319, 1145, 1082, 1041, 758, 690.

(*E*)-5-Iodo-2-methyl-3-((phenylsulfonyl)methylene)isoindolin-1-one (3k).

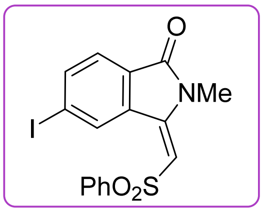

Off-white semisolid; eluent (20% ethyl acetate in hexanes). The reaction scale is 75 mg (**1k** (75 mg), **2a** (1.5 equiv)), 85 mg of **3k** was isolated and yield is 69 %. The reaction was done for 72 h at 120 °C.

^{1}**H NMR ($CDCl_3$, 400 MHz):** δ 9.17 (d, *J* = 1.2 Hz, 1 H), 8.01 – 8.98 (m, 2 H), 7.94 (dd, *J* = 8.0, 1.6 Hz, 1 H), 7.65 – 7.56 (m, 3 H), 7.52 (dd, *J* = 7.6, 0.4 Hz, 1 H), 6.15 (s, 1 H), 3.17 (s, 3 H).

^{13}**C NMR ($CDCl_3$, 100 MHz)**: δ 166.1, 145.7, 142.1, 140.9, 136.9, 133.9, 133.5, 129.6, 129.4, 127.1, 124.8, 109.3, 100.4, 26.6 (Two carbon signals are missing due to overlap).

HRMS (ESI): calc. for [($C_{16}H_{12}INO_3S$)H] (M+H) 425.9661, measured 425.9656.

IR (ATR)$\tilde{\nu}$ (cm^{-1}): 3059, 1727, 1605, 1386, 1317, 1147, 1084, 1043, 894, 753.

(*E*)-5-Bromo-2-methyl-3-((phenylsulfonyl)methylene)isoindolin-1-one (3l).

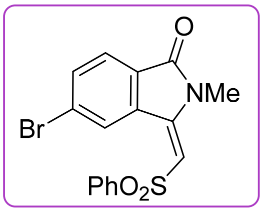

Colorless semisolid; eluent (20% ethyl acetate in hexanes). The reaction scale is 75 mg (**1l** (75 mg), **2a** (1.5 equiv)), 89 mg of **3l** was isolated and yield is 67 %. The reaction was done for 72 h at 120 °C.

^{1}**H NMR ($CDCl_3$, 400 MHz):** δ 9.01 (d, *J* = 1.2 Hz, 1 H), 8.01 – 7.98 (m, 2 H), 7.73 (dd, *J* = 9.6, 1.6 Hz, 1 H), 7.67 – 7.53 (m, 4 H), 6.15 (s, 1 H), 3.16 (s, 3 H).

^{13}C NMR (CDCl$_3$, 100 MHz): δ 165.8, 145.7, 142.2, 135.1, 133.8, 133.7, 131.2, 129.6, 128.9, 128.3, 127.1, 124.8, 109.4, 26.6 (Two carbon signals are missing due to overlap).

HRMS (ESI): calc. for [($C_{16}H_{12}BrNO_3S$)H] (M+H) 377.9800, measured 377.9805.

IR (ATR)ṽ (cm^{-1}): 3115, 3063, 2311, 1727, 1605, 1429, 1386, 1083, 1042, 847, 808.

(*E*)-5-Chloro-2-methyl-3-((phenylsulfonyl)methylene)isoindolin-1-one (3m).

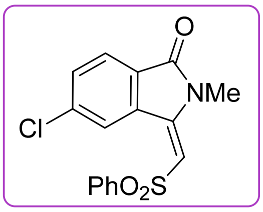

Off-white semisolid; eluent (25% ethyl acetate in hexanes). The reaction scale is 75 mg (**1m** (75 mg), **2a** (1.5 equiv)), 96 mg of **3m** was isolated and yield is 65 %. The reaction was done for 72 h at 120 °C.

^{1}H NMR (CDCl$_3$, 400 MHz): δ 8.86 (d, *J* = 1.2 Hz, 1 H), 8.00 – 7.98 (m, 2 H), 7.73 (d, *J* = 8.0 Hz, 1 H), 7.64 – 7.53 (m, 4 H), 6.14 (s, 1 H), 3.16 (s, 3 H).

^{13}C NMR (CDCl$_3$, 100 MHz): δ 165.7, 145.8, 142.2, 139.9, 133.8, 133.6, 132.1, 129.6, 128.4, 128.4, 127.1, 124.6, 109.4, 26.6 (Two carbon signals are missing due to overlap).

HRMS (ESI): calc. for [($C_{16}H_{12}ClNO_3S$)H] (M+H) 334.0305, measured 334.0317.

IR (ATR)ṽ (cm^{-1}): 3117, 3061, 2943, 2312, 1725, 1604, 1430, 1386, 1315, 1146, 1080, 1042, 902, 753.

(*E*)-5-Fluoro-2-methyl-3-((phenylsulfonyl)methylene)isoindolin-1-one (3n).

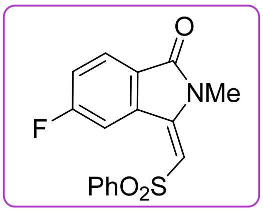

Yellow semisolid; eluent (25% ethyl acetate in hexanes). The reaction scale is 75 mg (**1n** (75 mg), **2a** (1.5 equiv)), 92 mg of **3n** was isolated and yield is 59 %. The reaction was done for 72 h at 120 °C.

^{1}H NMR (CDCl$_3$, 400 MHz): δ 8.63 (dd, *J* = 9.6, 2.4 Hz, 1 H), 8.01 – 7.99 (m, 2 H), 7.81 (dd, *J* = 8.4, 5.2 Hz, 1 H), 7.65 – 7.53 (m, 3 H), 7.30 – 7.25 (m, 1 H), 6.12 (s, 1 H), 3.16 (s, 3 H).

^{13}C NMR (CDCl$_3$, 100 MHz): δ 167.15 and 164.64 (F coupling), 165.7, 145.95 and 145.92(F coupling), 142.3, 134.53 and 134.42 (F coupling), 133.9, 129.6, 128.4, 127.0, 126.2, 125.65 and 125.55 (F coupling), 119.40 and 119.17 (F coupling), 116.15 and 115.88 (F coupling), 109.2, 26.7 (Two carbon signals are missing due to overlap).

HRMS (ESI): calc. for [($C_{16}H_{12}FNO_3S$)H] (M+H) 318.0600, measured 318.0612.

IR (ATR)$\tilde{\nu}$ (cm^{-1}): 3119, 3058, 2922, 1726, 1603, 1473, 1437, 1186, 1075, 848, 721.

(*E*)-2-Methyl-3-((phenylsulfonyl)methylene)-5-(trifluoromethyl)isoindolin-1-one (3o).

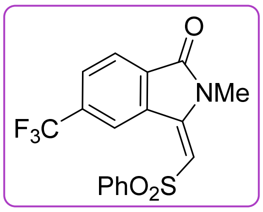

Off-white semisolid; eluent (25% ethyl acetate in hexanes). The reaction scale is 75 mg (**1o** (75 mg), **2a** (1.5 equiv)), 90 mg of **3o** was isolated and yield is 66 %. The reaction was done for 72 h at 120 °C.

^{1}H NMR (CDCl$_3$, 400 MHz): δ 9.14 (s, 1 H), 8.01 – 7.99 (m, 2 H), 7.93 (d, *J* = 8.0 Hz, 1 H), 7.85 (d, *J* = 8.0 Hz, 1 H), 7.64 – 7.53 (m, 3 H), 6.25 (s, 1 H), 3.22 (s, 3 H).

^{13}C NMR (CDCl$_3$, 100 MHz): δ 165.4, 145.7, 142.1, (135.67, 135.35, 135.02, 134.70) F coupling, 133.9, 133.0, 132.6, 129.6, (129.04, 129.00, 128.97, 128.93) F couplling, 127.1, (125.40, 125.36, 125.32, 125.28) F coupling, (127.59, 124.88, 122.16, 119.45) F coupling, 124.1, 111.2, 26.8 (Two carbon signals are missing due to overlap).

HRMS (ESI): calc. for [($C_{17}H_{12}F_3NO_3S$)H] (M+H) 368.0568, measured 368.0578.

(*E*)-2-Methyl-5-nitro-3-((phenylsulfonyl)methylene)isoindolin-1-one (3p).

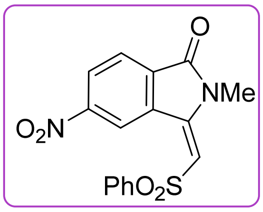

Tan semisolid; eluent (30% ethyl acetate in hexanes). The reaction scale is 75 mg (**1p** (75 mg), **2a** (1.5 equiv)), 83 mg of **3p** was isolated and yield is 58 %. The reaction was done for 72 h at 120 °C.

^{1}H NMR (CDCl$_3$, 400 MHz): δ 9.72 (d, *J* = 1.6 Hz, 1 H), 8.45 (dd, *J* = 8.0, 1.6 Hz, 1 H), 8.04 (d, *J* = 7.2 Hz, 2 H), 7.97 (d, *J* = 8.0 Hz, 1 H), 7.65 – 7.55 (m, 3 H), 6.32 (s, 1 H), 3.25 (s, 3 H).

^{13}**C NMR ($CDCl_3$, 100 MHz)**: δ 164.6, 151.1, 144.8, 141.7, 134.7, 134.1, 133.2, 129.7, 127.3, 127.1, 124.5, 123.6, 111.1, 27.0 (Two carbon signals are missing due to overlap).

HRMS (ESI): calc. for [($C_{16}H_{12}N_2O_5S$)H] (M+H) 345.0545, measured 345.0561.

IR (ATR)$\tilde{\nu}$ (cm^{-1}): 3129, 3060, 2312, 1731, 1610, 1536, 1436, 1346, 1148, 1074, 855, 746.

(*E*)-2,7-Dimethyl-3-((phenylsulfonyl)methylene)isoindolin-1-one (3q).

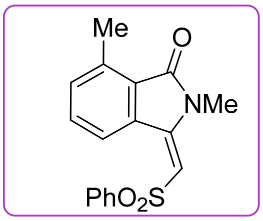

Yellow semisolid; eluent (20% ethyl acetate in hexanes). The reaction scale is 75 mg (**1q** (75 mg), **2a** (1.5 equiv)), 74 mg of **3q** was isolated and yield is 47 %. The reaction was done for 72 h at 120 °C.

^{1}**H NMR ($CDCl_3$, 400 MHz):** δ 8.64 (d, *J* = 8.0 Hz, 1 H), 8.02 – 7.99 (m, 2 H), 7.62 –7.57 (m, 1 H), 7.54 – 7.48 (m, 3 H), 7.32 (d, *J* = 7.6 Hz, 1 H), 6.06 (s, 1 H), 3.15 (s, 3 H), 2.66 (s, 3 H).

^{13}**C NMR ($CDCl_3$, 100 MHz)**: δ 167.4, 147.4, 142.7, 138.1, 134.4, 133.5, 132.8, 129.5, 126.9, 125.6, 107.1, 26.3, 17.6 (Four carbon signals are missing due to overlap).

HRMS (ESI): calc. for [($C_{17}H_{15}NO_3S$)H] (M+H) 314.0851, measured 314.0863.

IR (ATR)$\tilde{\nu}$ (cm^{-1}): 3058, 2929, 1720, 1600, 1435, 1145, 1045, 754.

(*E*)-6-Iodo-2-methyl-3-((phenylsulfonyl)methylene)isoindolin-1-one (3r).

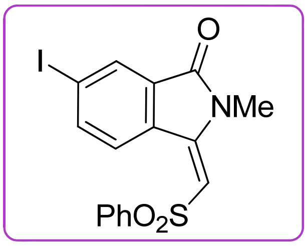

Colorless semisolid; eluent (20% ethyl acetate in hexanes). The reaction scale is 75 mg (**1r** (75 mg), **2a** (1.5 equiv)), 78 mg of **3r** was isolated and yield is 64 %. The reaction was done for 72 h at 120 °C.

^{1}**H NMR ($CDCl_3$, 400 MHz):** δ 8.58 (d, *J* = 8.4 Hz, 1 H), 8.15 (d, *J* = 1.2 Hz, 1 H), 8.00 – 7.97 (m, 3 H), 7.64 – 7.59 (m, 1 H), 7.56 – 7.52 (m, 2 H), 6.13 (s, 1 H), 3.16 (s, 3 H).

^{13}C NMR (CDCl$_3$, 100 MHz): δ 165.3, 146.4, 142.3, 133.9, 132.8, 131.7, 131.4, 129.6, 129.3, 126.9, 109.1, 98.67, 26.6 (Three carbon signals are missing due to overlap).

HRMS (ESI): calc. for [($C_{16}H_{12}INO_3S$)H] (M+H) 425.9661, measured 425.9658.

IR (ATR)$\tilde{\nu}$ (cm^{-1}): 3048, 1725, 1598, 1431, 1311, 1148, 1084, 841, 720.

(*E*)-6-Chloro-2-methyl-3-((phenylsulfonyl)methylene)isoindolin-1-one (3s).

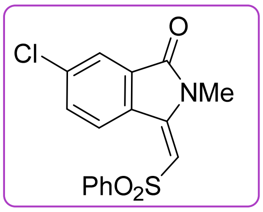

Yellow semisolid; eluent (25% ethyl acetate in hexanes). The reaction scale is 75 mg (**1s** (75 mg), **2a** (1.5 equiv)), 90 mg of **3s** was isolated and yield is 61 %. The reaction was done for 72 h at 120 °C.

^{1}H NMR (CDCl$_3$, 400 MHz): δ 8.80 (d, *J* = 8.4 Hz, 1 H), 8.00 – 7.97 (m, 2 H), 7.77 (d, *J* = 2.0 Hz, 1 H), 7.64 – 7.60 (m, 2 H), 7.56 – 7.52 (m, 2 H), 6.12 (s, 1 H), 3.17 (s, 3 H).

^{13}C NMR (CDCl$_3$, 100 MHz): δ 165.5, 146.1, 142.2, 138.6, 133.8, 133.4, 131.9, 130.3, 129.6, 129.3, 126.9, 123.9, 108.9, 26.7 (Three carbon signals are missing due to overlap).

HRMS (ESI): calc. for [($C_{16}H_{12}ClNO_3S$)H] (M+H) 334.0305, measured 334.0306.

IR (ATR)$\tilde{\nu}$ (cm^{-1}): 3123, 3065, 2925, 1726, 1601, 1458, 1323, 1149, 1083, 901, 756.

(*E*)-6,7-Dimethoxy-2-methyl-3-((phenylsulfonyl)methylene)isoindolin-1-one (3x).

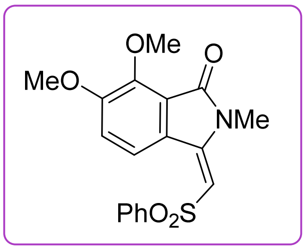

Yellow semisolid; eluent (35% ethyl acetate in hexanes). The reaction scale is 75 mg (**1t** (75 mg), **2a** (2.0 equiv)), 91 mg of **3x** was isolated and yield is 66 %. The reaction was done for 36 h at 120 °C.

^{1}H NMR (CDCl$_3$, 400 MHz): δ 8.50 (d, *J* = 8.8 Hz, 1 H), 7.98 (d, *J* = 7.6 Hz, 2 H), 7.60 – 7.49 (m, 3 H), 7.07 (d, *J* = 8.8 Hz, 1 H), 5.97 (s, 1 H), 4.00 (s, 3 H), 3.91 (s, 3 H), 3.12 (s, 3 H).

^{13}C NMR (CDCl$_3$, 100 MHz): δ 164.8, 155.9, 147.3, 147.1, 142.9, 133.4, 129.4, 126.8, 124.8, 124.5, 121.8, 115.9, 105.6, 62.5, 56.6, 26.4 (Two carbon signals are missing due to overlap).

HRMS (ESI): calc. for [($C_{18}H_{17}NO_5S$)H] (M+H) 360.0906, measured 300.0907.

IR (ATR)$\tilde{\nu}$ (cm^{-1}): 3060, 2942, 2839, 1723, 1596, 1499, 1434, 1390, 1275, 1144, 941, 754.

(*E*)-5,6,7-Trimethoxy-2-methyl-3-((phenylsulfonyl)methylene)isoindolin-1-one (3y).

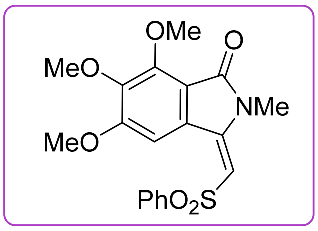

Yellow semisolid; eluent (40% ethyl acetate in hexanes). The reaction scale 75 mg (**1u** (75 mg), **2a** (2.0 equiv)), 84 mg of **3y** was isolated and yield is 65%. The reaction was done for 36 h at 120 °C.

^{1}H NMR (CDCl$_3$, 400 MHz): δ 8.24 (s, 1 H), 7.99 - 7.97 (m, 2 H), 7.62 - 7.58 (m, 1 H), 7.54 - 7.51 (m, 2 H), 5.99 (s, 1 H), 4.03 (s, 3 H), 3.94 (s, 3 H), 3.89 (s, 3 H), 3.10 (s, 3 H).

^{13}C NMR (CDCl$_3$, 100 MHz): δ 164.9, 157.7, 151.4, 147.2, 144.9, 142.8, 133.6, 129.5, 128.8, 126.7, 114.7, 107.9, 106.2, 62.6, 61.6, 56.7, 26.3 (Two carbon signals are missing due to overlap).

IR (ATR)$\tilde{\nu}$ (cm^{-1}): 3133, 2938, 2849, 1720, 1685, 1594, 1423, 138 6, 1139, 1074, 739

HRMS (ESI): calc. for [($C_{19}H_{19}NO_6S$)H] (M+H) 390.1011, measured 390.1009.

(*E*)-6-Hydroxy-5-methoxy-2-methyl-3-((phenylsulfonyl)methylene)isoindolin-1-one (3z).

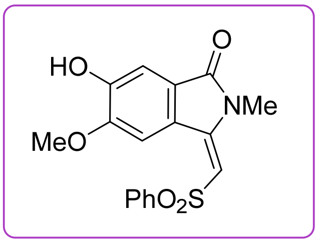

Yellow solid; eluent (50% ethyl acetate in hexanes). The reaction scale is 300 mg (**1v** (300 mg), **2a** (2.0 equiv)), 372 mg of **3z** was isolated and the yield is 65%. The reaction was done for 36 h at 120 °C.

^{1}H NMR (DMSO-*d*$_6$, 400 MHz): δ 10.46 (bs, 1 H), 8.06 - 8.04 (m, 3 H), 7.70 - 7.61 (m, 3 H), 7.08 (s, 1 H), 6.51 (s, 1 H), 3.82 (s, 3 H), 3.12 (s, 3 H).

^{13}C NMR (DMSO-d_6, 100 MHz):δ 166.2, 151.4, 150.5, 147.6, 142.8, 133.7, 129.7, 126.4, 123.6, 123.3, 110.6, 109.3, 105.9, 56.1, 26.4 (Two carbon signals are missing due to overlap).

HRMS (ESI): calc. for [($C_{17}H_{15}NO_5S$)H] (M+H) 346.0749, measured 346.0753.

IR (ATR)$\tilde{\nu}$ (cm^{-1}): 3003, 2945, 2836, 1714, 1597, 1499, 1307, 1143, 1082, 721.

(*E*)-6-(Benzyloxy)-5-methoxy-2-methyl-3-((phenylsulfonyl)methylene)isoindolin-1-one (11a).

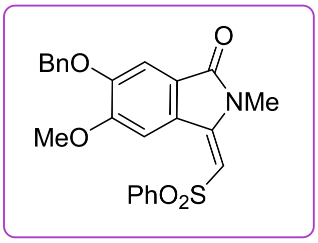

Light yellow solid; eluent (30% ethyl acetate in hexanes). The reaction scale is 300 mg (**3z**), 329 mg of **11a** was isolated and the yield is 87%.

^{1}H NMR ($CDCl_3$, 400 MHz):δ 8.43 (s, 1 H), 8.04 – 8.02 (m, 2 H), 7.65 - 7.54 (m, 3 H), 7.45 - 7.28 (m, 6 H), 6.06 (s, 1 H), 5.23 (s, 2 H), 4.01 (s, 3 H), 3.16 (s, 3 H).

^{13}C NMR ($CDCl_3$, 100 MHz):δ 166.9, 153.4, 151.3, 147.7, 142.8, 135.9, 133.6, 129.5, 128.8, 128.4, 127.5, 126.7, 125.9, 123.5, 110.8, 107.1, 106.4, 71.0, 56.6, 26.4 (Four carbon signals are missing due to overlap).

HRMS (ESI): calc. for [($C_{24}H_{21}NO_5S$)H] (M+H) 436.1219, measured 436.1223.

IR (ATR)$\tilde{\nu}$ (cm^{-1}): 3060, 2928, 1715, 1596, 1465, 1300, 1214, 1033, 733.

(*E*)-5-Hydroxy-6-methoxy-2-methyl-3-((phenylsulfonyl)methylene)isoindolin-1-one (3wa).

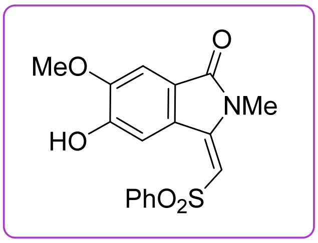

Yellow solid; **m.p.** 197-198 °C, eluent (50% ethyl acetate in hexanes). The reaction scale is 300 mg (**1w** (300 mg), **2a** (2.0 equiv)), 361 mg of **3wa** was isolated and the yield is 63%. The reaction was done for 48 h at 120 °C.

^{1}H NMR (DMSO-d_6, 400 MHz): δ 10.37 (s, 1 H), 8.12 (s, 1 H), 8.08 - 8.06 (m, 2 H), 7.71 - 7.67 (m, 1 H), 7.65 -7 .61 (m, 2 H), 7.26 (s, 1 H), 6.51 (s, 1 H), 3.87 (s, 3 H), 3.12 (s, 3 H).

^{13}C NMR (DMSO-d_6, 100 MHz): δ 166.2, 150.9, 150.9, 147.2, 142.6, 133.5, 129.5, 126.6, 124.9, 121.7, 114.1, 106.6, 106.1, 56.1, 26.3 (Two carbon signals are missing due to overlap).

HRMS (ESI): calc. for [($C_{17}H_{15}NO_5S$)H] (M+H) 346.0749, measured 346.0745.

IR (ATR) $\tilde{\nu}$ (cm^{-1}): 3000, 2845, 2311, 1736, 1598, 1495, 1371, 1313, 1217, 1144, 1083, 1037, 863.

(*E*)-5-(Benzyloxy)-6-methoxy-2-methyl-3-((phenylsulfonyl)methylene)isoindolin-1-one (11b).

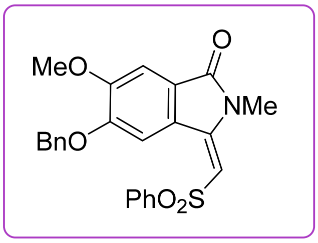

Light Yellow solid; **m.p.** 195-196 °C, eluent (30% ethyl acetate in hexanes). The reaction scale is 300 mg (**3wa**), 337 mg of **11b** was isolated and the yield is 89%.

^{1}H NMR ($CDCl_3$, 400 MHz): δ 8.51 (s, 1 H), 7.81 (d, J = 7.6 Hz, 2 H), 7.58 - 7.27 (m, 9 H), 5.96 (s, 1 H), 5.28 (s, 2 H), 3.95 (s, 3 H), 3.11 (s, 3 H).

^{13}C NMR ($CDCl_3$, 100 MHz): δ 166.9, 152.8, 151.9, 147.5, 142.8, 136.0, 133.4, 129.5, 128.8, 128.3, 127.7, 126.7, 125.6, 123.9, 112.2, 106.7, 105.6, 71.1, 56.5, 26.5 (Four carbon signals are missing due to overlap).

HRMS (ESI): calc. for [($C_{24}H_{21}NO_5S$)H] (M+H) 436.1219, measured 436.1223.

IR (ATR) $\tilde{\nu}$ (cm^{-1}): 3059, 2935, 1722, 1595, 1496, 1430, 1303, 1145, 1034, 855, 750.

(*E*)-5,6-Dimethoxy-2-(4-methoxybenzyl)-3-((phenylsulfonyl)methylene)isoindolin-1-one (3xa).

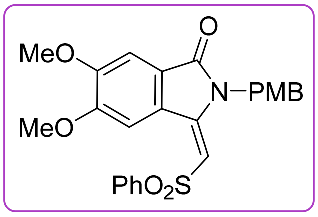

Yellow semisolid; eluent (35% ethyl acetate in hexanes). The reaction scale 75 mg (**1v** (75 mg), **2a** (2.0 equiv)), 79 mg of **3xa** was isolated and yield is 63%. The reaction was done for 16 h at 120 °C.

^{1}H NMR ($CDCl_3$, 400 MHz): δ 8.31 (s, 1 H), 7.72 (d, *J* = 7.6 Hz, 2 H), 7.53 (t, *J* = 7.6 Hz, 1 H), 7.42 (t, *J* = 7.6 Hz, 2 H), 7.31 (s, 1 H), 7.02 (d, *J* = 8.0 Hz, 2 H), 6.76 (d, *J* = 8.0 Hz, 2 H), 6.02 (s, 1 H), 4.84 (s, 2 H), 3.96 (s, 3 H), 3.96 (s, 3 H), 3.76 (s, 3 H).

^{13}C NMR ($CDCl_3$, 100 MHz):δ 162.9, 159.1, 149.4, 134.1, 133.3, 129.6, 129.4, 129.3, 128.2, 126.4, 125.3, 114.5, 114.4, 114.2, 108.0, 107.2, 105.5, 55.8, 55.4, 54.7, 43.2, 18.2 (Three carbon signals are missing due to overlap).

HRMS (ESI): calc. for [($C_{25}H_{23}NO_6S$)H] (M+H) 466.1324, measured 466.1326.

IR (ATR)$\tilde{\nu}$ (cm^{-1}): 2923, 2845, 1727, 1596, 1507, 1459, 1309, 1009, 831, 752.

(*E*)-5,6,7-Trimethoxy-2-(4-methoxybenzyl)-3-((phenylsulfonyl)methylene)isoindolin-1-one (3ya).

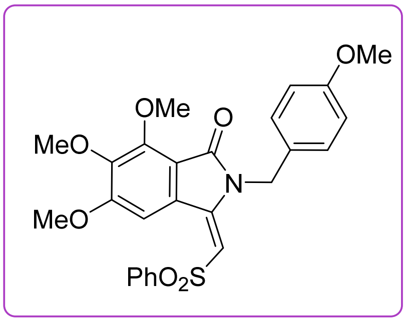

Brickred semisolid; eluent (35% ethyl acetate in hexanes). The reaction scale is 75 mg (**1y** (75 mg), **2a** (2.0 equiv)), 70 mg of **5cg** was isolated and yield is 62%. The reaction was done for 16 h at 120 °C.

^{1}H NMR ($CDCl_3$, 400 MHz): δ 8.28 (s, 1 H), 7.72 (d, *J* = 8.0 Hz, 2 H), 7.54 (t, *J* = 7.6 Hz, 1 H), 7.43 (t, *J* = 7.6 Hz, 2 H), 7.04 (d, *J* = 7.6 Hz, 2 H), 6.76 (d, *J* = 7.6 Hz, 2 H), 6.00 (s, 1 H), 4.80 (s, 2 H), 4.09 (s, 3 H), 3.93 (s, 3 H), 3.90 (s, 3 H), 3.76 (s, 3 H).

^{13}C NMR ($CDCl_3$, 100 MHz): δ 165.2, 159.3, 157.9, 151.6, 145.6, 144.9, 142.8, 133.3, 129.3, 129.0, 128.4, 127.4, 126.4, 114.4, 107.9, 107.7, 62.6, 61.6, 56.8, 55.4, 42.8 (Five carbon signals are missing due to overlap).

HRMS (ESI): calc. for [($C_{26}H_{25}NO_7S$)H] (M+H) 496.1430, measured 496.1434.

IR (ATR)$\tilde{\nu}$ (cm^{-1}): 3003, 2944, 1737, 1598, 1514, 1448, 1367, 1216.

Methyl (*E*)-2-(5,6-dimethoxy-2-methyl-3-oxoisoindolin-1-ylidene)acetate (3t).

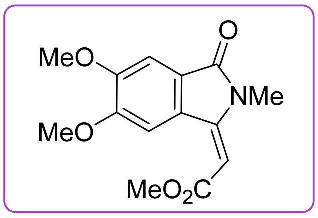

Golden yellow semisolid; eluent (25% ethyl acetate in hexanes). The reaction scale is 75 mg (**1a** (75 mg), **2b** (4.0 equiv)), 79 mg of **3t** was isolated and yield is 74%. The reaction was done for 36 h at 120 °C.

^{1}H NMR (DMSO-*d*$_6$, 400 MHz): δ 8.59 (s, 1 H), 7.24 (s, 1 H), 5.69 (s, 1 H), 3.88 (s, 3 H), 3.85 (s, 3 H), 3.74 (s, 3 H), 3.13 (s, 3 H).

^{13}C NMR (DMSO-*d*$_6$, 100 MHz):δ 166.3, 152.3, 151.5, 149.2, 126.4, 123.0, 109.9, 104.9, 96.6, 55.9, 55.7, 51.5, 26.0 (One carbon signal is missing due to overlap).

HRMS (ESI): calc. for [($C_{14}H_{15}NO_5$)H] (M+H) 278.1028, measured 278.1038.

IR (ATR)$\tilde{\nu}$ (cm^{-1}): 2928, 2848, 1712, 1624, 1592, 1429, 1337, 1298, 1166, 1045, 774.

Butyl (*E*)-2-(5,6-dimethoxy-2-methyl-3-oxoisoindolin-1-ylidene)acetate (3u).

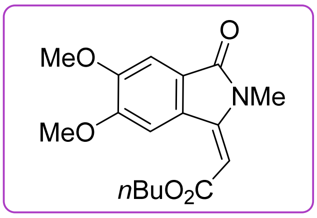

Golden yellow semisolid; eluent (20% ethyl acetate in hexanes). The reaction scale is 75 mg (**1a** (75 mg), **2c** (4.0 equiv)), 88 mg of **3u** was isolated and yield is 72%. The reaction was done for 36 h at 120 °C.

^{1}H NMR (DMSO-*d*$_6$, 400 MHz): δ 8.54 (s, 1 H), 7.16 (s, 1 H), 5.62 (s, 1 H), 4.15 (t, *J* = 6.8 Hz, 2 H), 3.87 (s, 3 H), 3.83 (s, 3 H), 3.11 (s, 3 H), 1.67 - 1.60 (m, 2 H), 1.44 - 1.35 (m, 2 H), 0.92 (t, *J* = 7.2 Hz, 3 H).

^{13}C NMR (DMSO-*d*$_6$, 100 MHz):δ 166.2, 165.8, 152.1, 151.4, 149.1, 126.4, 122.9, 109.9, 104.7, 96.7, 63.7, 55.8, 55.6, 30.3, 25.9, 18.7, 13.6.

HRMS (ESI): calc. for [($C_{17}H_{21}NO_5$)H] (M+H) 320.1498, measured 320.1496.

IR (ATR)$\tilde{\nu}$ (cm^{-1}): 2923, 1713, 1626, 1501, 1466, 1380, 1300, 1167, 1040.

Cyclohexyl (*E*)-2-(5,6-dimethoxy-2-methyl-3-oxoisoindolin-1-ylidene)acetate (3v).

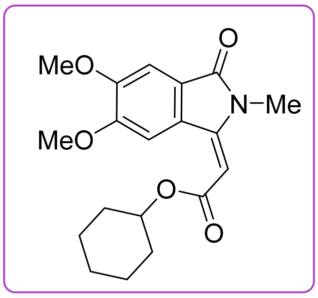

Colorless semisolid; eluent (20% ethyl acetate in hexanes). The reaction scale is 75 mg (**1a** (75 mg), **2d** (2.0 equiv)), 84 mg of **3v** was isolated and yield is 63%. The reaction was done for 36 h at 120 °C.

^{1}H NMR ($CDCl_3$, 400 MHz): δ 8.75 (s, 1 H), 7.26 (s, 1 H), 5.60 (s, 1 H), 4.89 - 4.83 (m, 1 H), 4.01 (s, 3 H), 3.95 (s, 3 H), 3.22 (s, 3 H), 1.95 – 1.91 (m, 2 H), 1.78 – 1.74 (m, 2 H), 1.59 – 1.55 (m, 1 H), 1.51 – 1.36 (m, 4 H), 1.31 – 1.22 (m, 1 H).

^{13}C NMR ($CDCl_3$, 100 MHz): δ 167.6, 165.9, 152.9, 151.7, 149.6, 127.7, 123.8, 110.8, 104.9, 98.3, 72.8, 56.6, 56.4, 32.0, 26.4, 25.6, 24.1 (Two carbon signals are missing due to overlap).

HRMS (ESI): calc. for [($C_{19}H_{23}NO_5$)H] (M+H) 346.1654, measured 346.1662.

IR (ATR)$\tilde{\nu}$ (cm^{-1}): 2934, 2857, 1711, 1627, 1495, 1336, 1169, 1042, 826, 732.

5,6-Dimethoxy-2-methyl-3-methyleneisoindolin-1-one (3w).

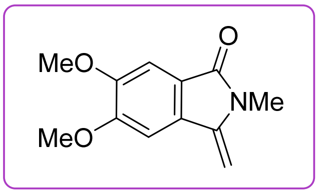

Pale yellow solid; eluent (20% ethyl acetate in hexanes). The reaction scale is 75 mg (**1a** (75 mg), **2e** (2.0 equiv)), 45 mg of **3w** was isolated and yield is 54%. The reaction was done for 36 h at 120 °C.

^{1}H NMR ($CDCl_3$, 400 MHz): δ 7.25 (s, 1 H), 7.09 (s, 1 H), 5.03 (d, J = 2.0 Hz, 1 H), 4.76 (d, J = 2.0 Hz, 1 H), 3.97 (s, 3 H), 3.93 (s, 3 H), 3.23 (s, 3 H).

^{13}C NMR ($CDCl_3$, 100 MHz):δ 167.5, 152.9, 151.1, 143.2, 130.0, 122.7, 104.8, 102.1, 87.7, 56.5, 56.4, 25.9.

HRMS (ESI): calc. for [($C_{12}H_{13}NO_3$)H] (M+H) 220.0974, measured 220.0970.

IR (ATR)$\tilde{\nu}$ (cm^{-1}): 2924, 2847, 2312, 1740, 1705, 1612, 1498, 1433, 1305, 1218, 1030, 779, 741.

1,2,5-Trimethyldibenzo[*cd,f*]indol-4(5*H*)-one (9a).

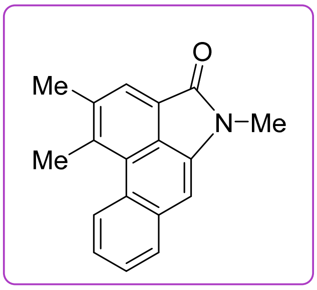

Off-white solid; **m.p.** 194-195 °C, eluent (15% ethyl acetate in hexanes). The reaction scale is 80 mg (**3g** (80 mg), **7a** (2.0 equiv)), 42 mg of **9a** was isolated and yield is 66%.

^{1}H NMR ($CDCl_3$, 400 MHz): δ 8.74 (d, *J* = 7.6 Hz, 1 H), 7.90 (s, 1 H), 7.85 (d, *J* = 7.6 Hz, 1 H), 7.57 - 7.50 (m, 2 H), 6.99 (s, 1 H), 3.44 (s, 3 H), 2.97 (s, 3 H), 2.61 (s, 3 H).

^{13}C NMR ($CDCl_3$, 100 MHz): δ 168.5, 139.6, 138.7, 137.9, 135.6, 129.7, 129.1, 127.6, 127.0, 126.7, 126.7, 125.8, 125.1, 123.9, 104.7, 26.3, 22.7, 20.5. **HRMS (ESI):** calc. for [($C_{18}H_{15}NO$)H] (M+H) 262.1232, measured 262.1223.

IR (ATR)$\tilde{\nu}$ (cm^{-1}): 3048, 2920, 1697, 1650, 1458, 1338, 1306, 1249, 1085, 836, 750.

Compounds 9b (major isomer).

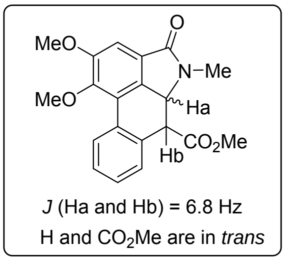

Pale yellow solid; eluent (40% ethyl acetate in hexanes).

^{1}H NMR ($CDCl_3$, 400 MHz): δ 8.42 (d, *J* = 8.0 Hz, 1 H), 7.47 - 7.42 (m, 1 H), 7.33 - 7.30 (m, 2 H), 7.24 (s, 1 H), 4.57 (d, *J* = 6.8 Hz, 1 H), 4.22 (d, *J* = 6.8 Hz, 1 H), 3.92 (s, 3 H), 3.82 (s, 3 H), 3.37 (s, 3 H), 3.20 (s, 3 H).

^{13}C NMR ($CDCl_3$, 100 MHz): 169.8, 169.1, 155.4, 149.3, 134.4, 132.9, 132.27, 130.5, 129.6, 128.6, 128.5, 126.3, 123.8, 105.7, 60.4, 58.9, 56.4, 52.3, 48.7, 28.3.

HRMS (ESI): calc. for [($C_{20}H_{19}NO_5$)H] (M+H) 354.1341, measured 354.1332.

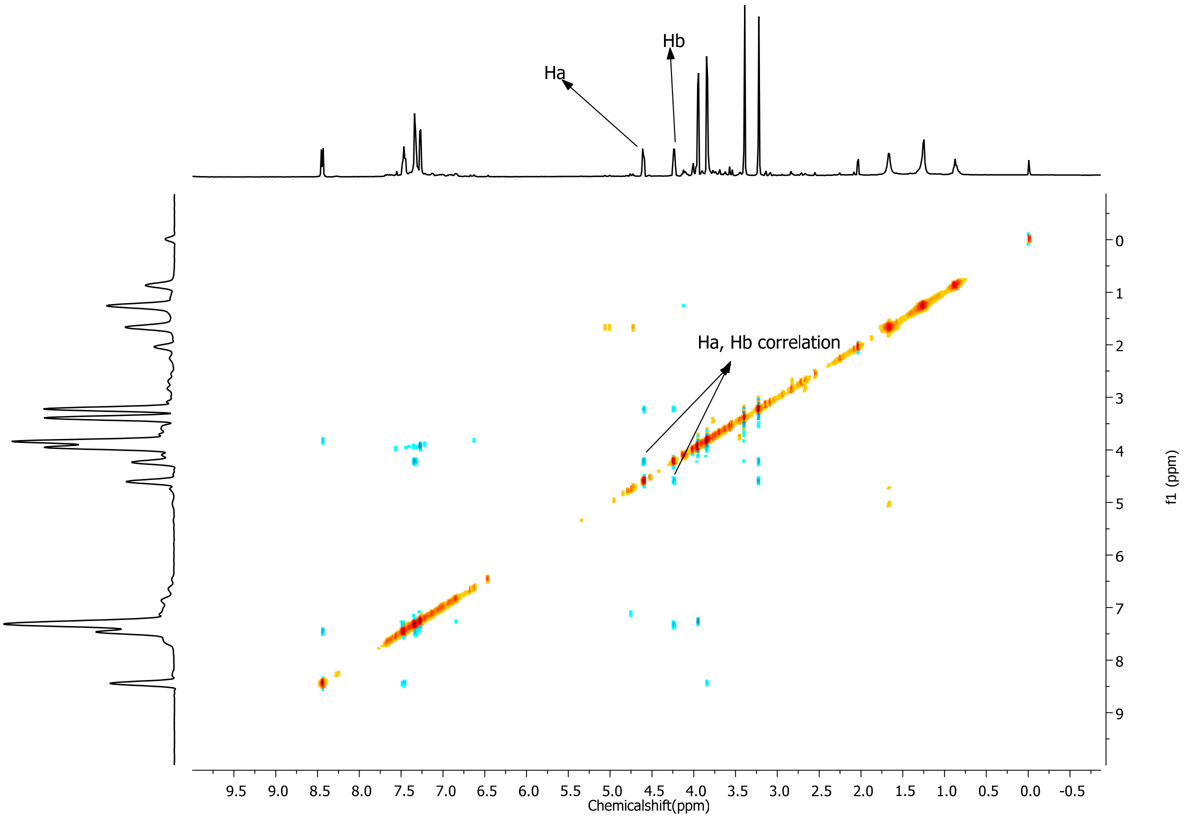

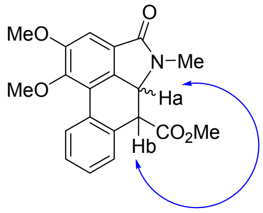

There is a strong NOE correlation between H_a (δ 4.57, d) and H_b (δ 4.22, d). It clearly indicates that H_a and CO_2Me are in trans to each other.

Compound 9b' (minor isomer).

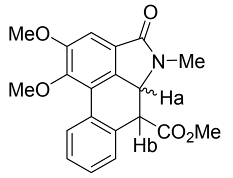

J (Ha and Hb) = 12.8 Hz
H and CO_2Me are in *cis*

Pale yellow solid; eluent (40% ethyl acetate in hexanes).

^{1}H NMR ($CDCl_3$, 400 MHz): δ 8.40 (dd, *J* = 8.0, 4.0 Hz, 1 H), 7.44 - 7.40 (m, 1 H), 7.34 - 7.30 (m, 2 H), 7.05 (d, *J* = 8.0 Hz, 1 H), 4.72 (d, *J*

= 12.8 Hz, 1 H), 3.95 (s, 3 H), 3.94 (s, 3 H), 3.84 (s, 3 H), 3.61 (d, J = 12.8 Hz, 1 H), 3.07 (s, 3 H).

^{13}C NMR ($CDCl_3$, 100 MHz): δ 173.7, 169.8, 155.4, 149.3, 135.2, 133.3, 131.3, 128.9, 128.9, 128.2, 127.1, 125.6, 122.9, 105.9, 60.5, 59.8, 56.5, 52.8, 52.1, 28.8.

HRMS (ESI): calc. for [($C_{20}H_{19}NO_5$)H] (M+H) 354.1341, measured 354.1333.

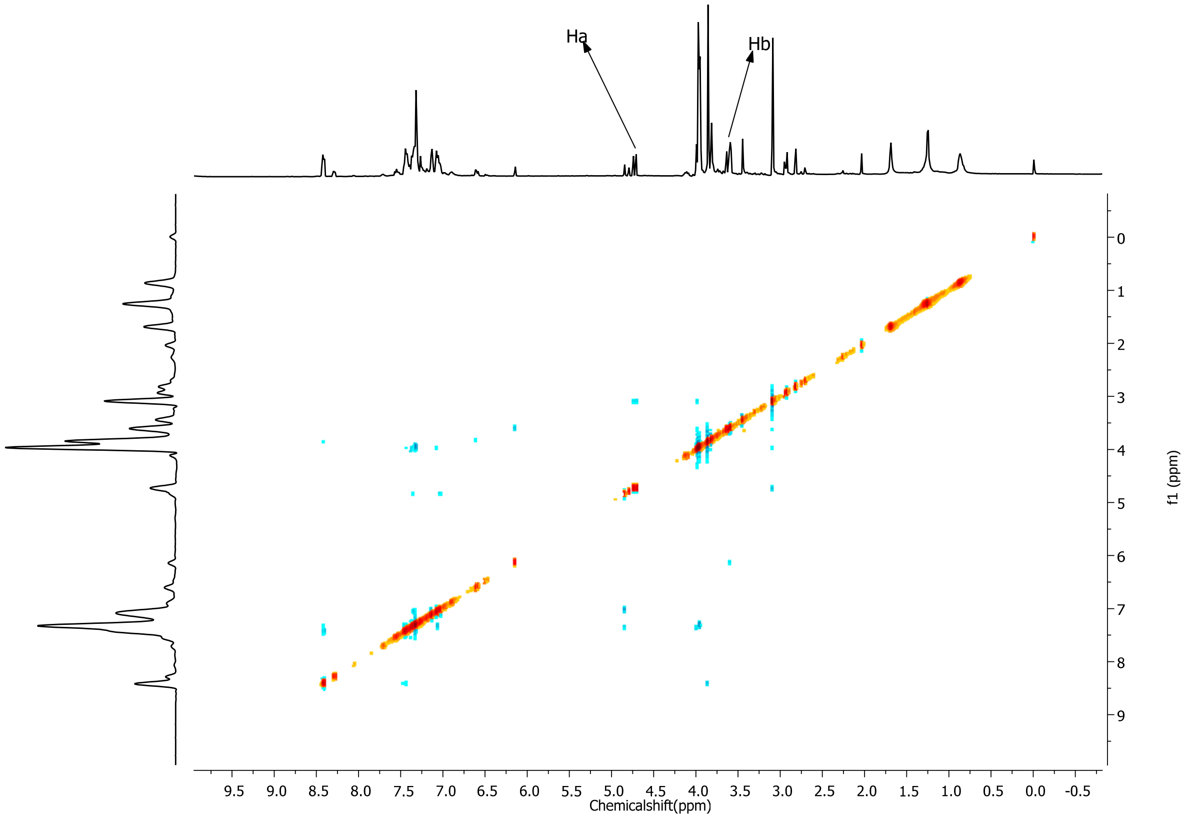

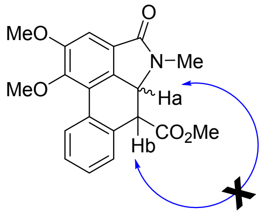

There is no NOE correlation between H_a (δ 4.57, d) and H_b (δ 4.22, d). It clearly indicates that H_a and CO_2Me are in cis to each other.

1,2-Dimethoxy-5-propyldibenzo[*cd,f*]indol-4(5*H*)-one (9c).

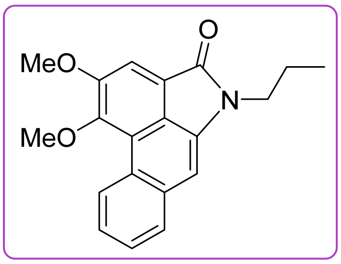

Pale yellow solid; **m.p.** 153-154 °C, eluent (20% ethyl acetate in hexanes). The reaction scale is 80 mg (**3b** (80 mg), **7a** (2.0 equiv)), 44 mg of **9c** was isolated and yield is 66%.

^{1}H NMR ($CDCl_3$, 400 MHz): δ 9.22 – 9.20 (m, 1 H), 7.82 – 7.79 (m, 2 H), 7.58 – 7.51 (m, 2 H), 6.99 (s, 1 H), 4.09 (s, 3 H), 4.05 (s, 3 H), 3.92 (t, J = 8.0 Hz, 2 H), 1.89 – 1.80 (m, 2 H), 1.01 (t, J = 7.2 Hz, 3 H).

^{13}C NMR ($CDCl_3$, 100 MHz): δ 168.1, 154.6, 151.2, 136.8, 134.9, 129.1, 127.7, 127.6, 127.2, 125.9, 123.2, 121.5, 120.9, 109.7, 104.5, 60.5, 57.1, 42.1, 22.4, 11.6.

HRMS (ESI): calc. for [($C_{20}H_{19}NO_3$)H] (M+H) 322.1443, measured 322.1448.

IR (ATR)$\tilde{\nu}$ (cm^{-1}): 2926, 2854, 1698, 1644, 1607, 1498, 1456, 1281, 1038, 751.

5-Butyl-1,2-dimethoxydibenzo[*cd,f*]indol-4(5*H*)-one (9d).

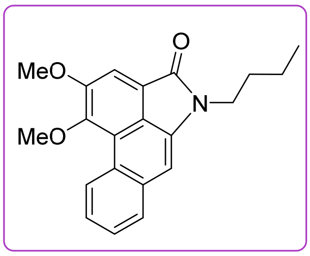

Brick red solid; **m.p.** 108-109 °C, eluent (20% ethyl acetate in hexanes). The reaction scale is 80 mg (**3c** (80 mg), **7a** (2.0 equiv)), 46 mg of **9d** was isolated and yield is 68%.

^{1}H NMR ($CDCl_3$, 400 MHz): δ 9.22-9.20 (m, 1 H), 7.82 - 7.78 (m, 2 H), 7.57 - 7.52 (m, 2 H), 6.98 (s, 1 H), 4.09 (s, 3 H), 4.05 (s, 3 H), 3.95 (t, J = 7.2 Hz, 2 H), 1.83 - 1.76 (m, 2 H), 1.48 - 1.39 (m, 2 H), 0.96 (t, J = 7.2 Hz, 3 H).

^{13}C NMR ($CDCl_3$, 100 MHz): δ 168.0, 154.5, 151.2, 136.8, 134.9, 129.1, 127.7, 127.6, 127.2, 125.9, 123.2, 121.5, 120.9, 109.7, 104.5, 60.5, 57.1, 40.2, 31.2, 20.4, 13.9.

HRMS (ESI): calc. for [($C_{21}H_{21}NO_3$)H] (M+H) 336.1600, measured 336.1599.

IR (ATR)$\tilde{\nu}$ (cm^{-1}): 2931, 2864, 1697, 1645, 1405, 1317, 979, 752.

5-Isopropyl-1,2-dimethoxydibenzo[*cd,f*]indol-4(5*H*)-one (9e).

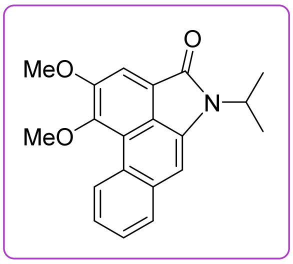

Colorless solid; **m.p.** 158-159 °C, eluent (20% ethyl acetate in hexanes). The reaction scale is 80 mg (**3d** (80 mg), **7a** (2.0 equiv)), 37 mg of **9e** was isolated and yield is 56%.

^{1}H NMR ($CDCl_3$, 400 MHz): δ 9.21 (d, *J* = 6.8 Hz, 1 H), 7.82 - 7.77 (m, 2 H), 7.57 - 7.52 (m, 2 H), 7.14 (s, 1 H), 4.92 - 4.82 (m, 1 H), 4.08 (s, 3 H), 4.05 (s, 3 H), 1.60 (d, *J* = 7.2 Hz, 6 H).

^{13}C NMR ($CDCl_3$, 100 MHz): δ 167.7, 154.5, 151.3, 135.4, 134.9, 129.2, 127.6, 126.9, 126.0, 123.3, 121.5, 121.1, 109.5, 106.3, 60.5, 57.1, 44.0, 21.0 (Two carbon signals are missing due to overlap).

HRMS (ESI): calc. for [($C_{20}H_{19}NO_3$)H] (M+H) 322.1443, measured 322.1439.

IR (ATR)$\tilde{\nu}$ (cm^{-1}): 2959, 2309, 1697, 1641, 1606, 1511, 1210, 746.

5-Cyclohexyl-1,2-dimethoxydibenzo[*cd,f*]indol-4(5*H*)-one (9f).

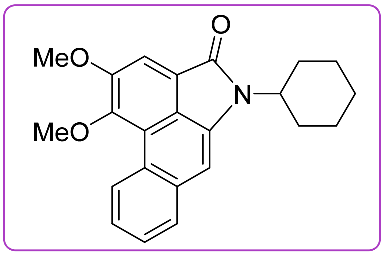

Paleyellow semisolid; **m.p.** 171-172 °C, eluent (20% ethyl acetate in hexanes). The reaction scale is 80 mg (**3e** (80 mg), **7a** (2.0 equiv)), 40 mg of **9f** was isolated and yield is 59%.

^{1}H NMR ($CDCl_3$, 400 MHz): δ 9.22 – 9.19 (m, 1 H), 7.83 – 7.80 (m, 1 H), 7.77 (s, 1 H), 7.58 – 7.51 (m, 2 H), 7.17 (s, 1 H), 4.46 – 4.38 (m, 1 H), 4.08 (s, 3 H), 4.05 (s, 3 H), 2.25 – 2.15 (m, 2 H), 1.96 – 1.78 (m, 5 H), 1.56 – 1.45 (m, 2 H), 1.40 – 1.32 (m, 1 H).

^{13}C NMR ($CDCl_3$, 100 MHz): δ 167.7, 154.5, 151.3, 135.9, 134.9, 129.2, 127.6, 127.6, 126.9, 125.9, 123.3, 121.5, 121.0, 109.6, 106.5, 60.5, 57.1, 52.2, 31.0, 26.3, 25.7 (Two carbon signals are missing due to overlap).

HRMS (ESI): calc. for [($C_{23}H_{23}NO_3$)H] (M+H) 362.1756, measured 362.1756.

IR (ATR)$\tilde{\nu}$ (cm^{-1}): 3055, 2926, 2854, 1729, 1694, 1640, 1539, 1399, 1280, 1042, 849, 737.

5-Benzyl-1,2-dimethoxydibenzo[*cd,f*]indol-4(5*H*)-one(9g).

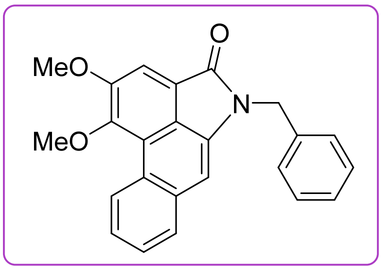

Greenish yellow solid; **m.p.** 166-167 °C, eluent (20% ethyl acetate in hexanes). The reaction scale is 80 mg (**3f** (80 mg), **7a** (2.0 equiv)), 44 mg of **9g** was isolated and yield is 65%.

^{1}H NMR ($CDCl_3$, 400 MHz): δ 9.24 – 9.23 (m, 1 H), 7.87 (s, 1 H), 7.76 – 7.74 (m, 1 H), 7.56 – 7.28 (m, 7 H), 6.90 (s, 1 H), 5.20 (s, 2 H), 4.13 (s, 3 H), 4.10 (s, 3 H).

^{13}C NMR ($CDCl_3$, 100 MHz): δ 168.0, 154.6, 151.5, 137.0, 136.3, 134.8, 129.2, 128.9, 127.7, 127.6, 127.4, 127.2, 126.0, 123.4, 121.1, 120.9, 109.9, 105.4, 60.5, 57.1, 44.1 (Three carbon signals are missing due to overlap).

HRMS (ESI): calc. for [($C_{24}H_{19}NO_3$)H] (M+H) 370.1443, measured 370.1450.

IR (ATR)$\tilde{\nu}$ (cm^{-1}): 2932, 2847, 1698, 1646, 1454, 1316, 1279, 1070, 702.

1-Methoxy-5-methyldibenzo[cd,f]indol-4(5*H*)-one (9h).

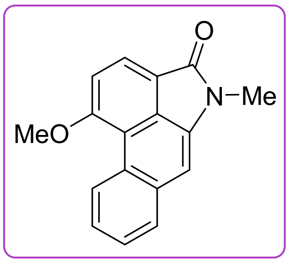

Pale yellow solid; eluent (20% ethyl acetate in hexanes). The reaction scale 80 mg (**3h** (80 mg), **7a** (2.0 equiv)), 48 mg of **9h** was isolated and yield is 75%.

^{1}H NMR ($CDCl_3$, 400 MHz): δ 9.24 - 9.22 (m, 1 H), 8.04 (d, *J* = 8.0 Hz, 1 H), 7.87 - 7.84 (m, 1 H), 7.57 - 7.52 (m, 2 H), 7.21 (d, *J* = 8.0 Hz, 1 H), 7.09 (s, 1 H), 4.19 (s, 3 H), 3.47 (s, 3 H).

^{13}C NMR ($CDCl_3$, 100 MHz): δ 168.1, 162.3, 137.4, 134.3, 129.6, 128.9, 128.3, 127.5, 126.9, 125.7, 125.5, 118.8, 116.9, 110.2, 105.5, 56.4, 26.4.

HRMS (ESI): calc. for [($C_{17}H_{13}NO_2$)H] (M+H) 264.1025, measured 264.1018.

IR (ATR)$\tilde{\nu}$ (cm^{-1}): 2924, 2847, 1740, 1705, 1612, 1498, 1433, 1305, 1218, 1030, 779, 741.

1,5-Dimethyldibenzo[*cd,f*]indol-4(5*H*)-one (9i).

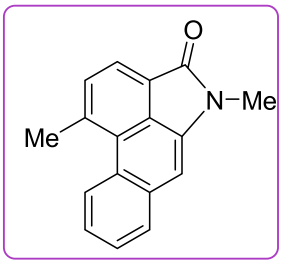

Yellow solid; **m.p.** 185-185 °C, eluent (15% ethyl acetate in hexanes). The reaction scale is 80 mg (**3i** (80 mg), **7a** (2.0 equiv)), 50 mg of **9i** was isolated and yield is 79%.

^{1}H NMR ($CDCl_3$, 400 MHz): δ 8.70 (dd, *J* = 7.6, 2.0 Hz, 1 H), 7.95 (d, *J* = 7.6 Hz, 1 H), 7.87 (dd, *J* = 7.6, 2.0 Hz, 1 H), 7.59 – 7.53 (m, 3 H), 7.05 (s, 1 H), 3.46 (s, 3 H), 3.10 (s, 3 H).

^{13}C NMR ($CDCl_3$, 100 MHz): δ 168.2, 141.2, 138.0, 135.2, 132.9, 129.6, 129.1, 128.5, 127.2, 126.9, 126.3, 125.4, 124.9, 123.5, 105.4, 26.3, 25.9.

HRMS (ESI): calc. for [($C_{17}H_{13}NO$)H] (M+H) 248.1075, measured 248.1078.

IR (ATR)$\tilde{\nu}$ (cm^{-1}): 3034, 2956, 1701, 1649, 1606, 1514, 1393, 716.

5-Methyldibenzo[*cd,f*]indol-4(5*H*)-one (9j).

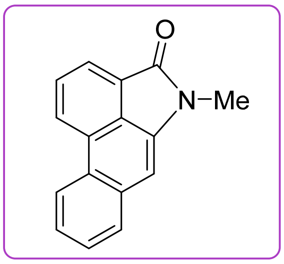

Yellowish green solid; **m.p.** 174-175 °C. (lit.[2] 176 °C), eluent (15% ethyl acetate in hexanes). The reaction scale is 80 mg (**3j** (80 mg), **7a** (2.0 equiv)), 45 mg of **9j** was isolated and yield is 71%.

^{1}H NMR ($CDCl_3$, 400 MHz): δ 8.56 (d, *J* = 8.0 Hz, 1 H), 8.50 (dd, *J* = 7.6, 1.6 Hz, 1 H), 8.08 (d, J = 7.6 Hz, 1 H), 7.86 – 7.79 (m, 2 H), 7.60 – 7.54 (m, 2 H), 7.07 (s, 1 H), 3.49 (s, 3 H).

^{13}C NMR ($CDCl_3$, 100 MHz): δ 168.5, 137.9, 134.5, 129.2, 127.8, 127.7, 127.4, 127.2, 126.7, 126.3, 125.6, 123.8, 123.4, 104.8, 26.5 (One carbon signal is missing due to overlap).

HRMS (ESI): calc. for [($C_{16}H_{11}NO$)H] (M+H) 234.0919, measured 234.0926.

IR (ATR)$\tilde{\nu}$ (cm^{-1}): 3054, 2932, 1722, 1647, 1438, 1318, 1147, 1082, 921, 726.

1-Iodo-5-methyldibenzo[*cd,f*]indol-4(5*H*)-one (9k).

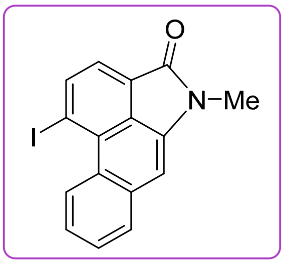

Brickred solid; **m.p.** 239-240 °C, eluent (15% ethyl acetate in hexanes). The reaction scale is 80 mg (**3k** (80 mg), **7a** (2.0 equiv)), 46 mg of **9k** was isolated and yield is 68%.

^{1}H NMR ($CDCl_3$, 400 MHz): δ 10.02 – 10.00 (m, 1 H), 8.45 (d, *J* = 7.2 Hz, 1 H), 7.82 – 7.80 (m, 1 H), 7.64 – 7.60 (m, 3 H), 7.06 (s, 1 H), 3.44 (s, 3 H).

^{13}C NMR ($CDCl_3$, 100 MHz): δ 167.6, 144.6, 137.2, 135.1, 129.6, 128.5, 128.4, 127.8, 127.4, 126.9, 124.8, 124.3, 123.9, 106.6, 95.8, 26.3.

HRMS (ESI): calc. for [($C_{16}H_{10}INO$)H] (M+H) 359.9885, measured 359.9892.

IR (ATR)$\tilde{\nu}$ (cm^{-1}): 2941, 1738, 1649, 1488, 1434, 1370, 1217.

1-Bromo-5-methyldibenzo[*cd,f*]indol-4(5H)-one (9l).

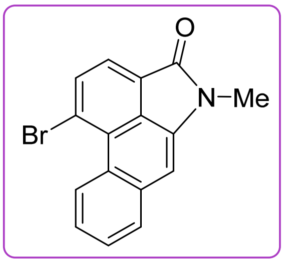

Yellow solid; **m.p.** 206-207 °C, eluent (15% ethyl acetate in hexanes). The reaction scale is 80 mg (**3l** (80 mg), **7a** (2.0 equiv)), 49 mg of **9l** was isolated and yield is 74%.

^{1}H NMR ($CDCl_3$, 400 MHz): δ 9.75 (d, *J* = 8.0 Hz, 1 H), 7.98 (d, *J* = 7.6 Hz, 1 H), 7.76 (d, *J* = 7.6 Hz, 2 H), 7.60 – 7.3 (m, 2 H), 6.96 (s, 1 H), 3.40 (s, 3 H).

^{13}C NMR ($CDCl_3$, 100 MHz): δ 167.3, 137.1, 136.4, 135.1, 129.5, 129.3, 128.2, 127.3, 126.0, 125.6, 125.4, 124.3, 123.8, 106.5, 26.3 (One carbon signal is missing due to overlap).

HRMS (ESI): calc. for [($C_{16}H_{10}BrNO$)H] (M+H) 312.0024, measured 312.0021.

IR (ATR)$\tilde{\nu}$ (cm^{-1}): 3050, 2921, 1708, 1649, 1596, 1439, 1387, 1307, 1027, 906, 745.

1-Chloro-5-methyldibenzo[*cd,f*]indol-4(5*H*)-one (9m).

Golden yellow solid; **m.p.** 215-216 °C, eluent (15% ethyl acetate in hexanes). The reaction scale is 80 mg (**3m** (80 mg), **7a** (2.0 equiv)), 50 mg of **9m** was isolated and yield is 77%.

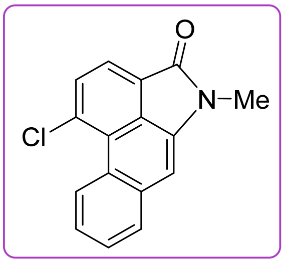

^{1}H NMR ($CDCl_3$, 400 MHz): δ 9.52 (dd, *J* = 7.6, 1.2 Hz, 1 H), 7.86 (d, *J* = 7.6 Hz, 1 H), 7.78 (dd, *J* = 7.6, 1.2 Hz, 1 H), 7.73 (d, *J* = 7.6 Hz, 1 H), 7.60 – 7.52 (m, 2 H), 6.97 (s, 1 H), 3.41 (s, 3 H).

^{13}C NMR ($CDCl_3$, 100 MHz): δ 167.2, 137.1, 135.9, 135.0, 132.4, 129.4, 129.4, 128.1, 127.1, 126.9, 125.8, 125.4, 124.2, 123.7, 106.4, 26.3.

HRMS (ESI): calc. for [($C_{16}H_{10}ClNO$)H] (M+H) 268.0529, measured 268.0532.

IR (ATR)$\tilde{\nu}$ (cm^{-1}): 3047, 2940, 2379, 1706, 1647, 1445, 1389, 1069, 1029, 869, 745.

1-Fluoro-5-methyldibenzo[*cd,f*]indol-4(5*H*)-one (9n).

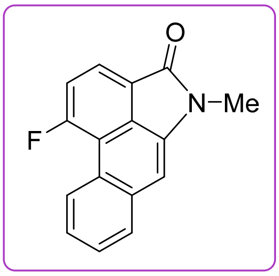

Golden yellow solid; **m.p.** 176-177 °C, eluent (15% ethyl acetate in hexanes). The reaction scale is 80 mg (**3n** (80 mg), **7a** (2.0 equiv)), 45 mg of **9n** was isolated and yield is 71%.

^{1}H NMR ($CDCl_3$, 400 MHz): δ 8.78 - 8.75 (m, 1 H), 8.01 - 7.98 (m, 1 H), 7.85 - 7.83 (m, 1 H), 7.61 - 7.55 (m, 2 H), 7.46 - 7.40 (m, 1 H), 7.06 (s, 1 H), 3.46 (s, 3 H).

^{13}C NMR ($CDCl_3$, 100 MHz): δ 167.4, 164.59 and 161.99 (F coupling), 137.01 and 136.97 (F coupling), 134.4, 129.99 and 129.91 (F coupling), 129.2, 128.03 and 128.01 (F coupling), 127.82 and 127.67 (F coupling), 126.3, 125.50 and 125.45 (F coupling), 125.31 and 125.21 (F coupling), 122.41 and 122.37 (F coupling), 116.34 and 116.09 (F coupling), 116.05 and 115.92 (F coupling), 105.8, 26.5 (One carbon signal is missing due to overlap).

HRMS (ESI): calc. for [($C_{16}H_{10}FNO$)H] (M+H) 252.0825, measured 252.0830.

IR (ATR)$\tilde{\nu}$ (cm^{-1}): 3053, 1701, 1649, 1615, 1419, 1263, 997, 895, 729.

5-Methyl-1-(trifluoromethyl)dibenzo[*cd,f*]indol-4(5*H*)-one (9o).

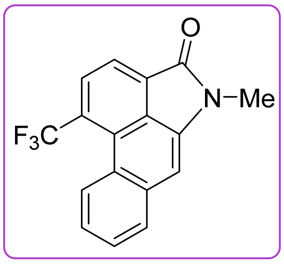

Greenish yellow solid; **m.p.** 164-165 °C, eluent (15% ethyl acetate in hexanes). The reaction scale is 80 mg (**3o** (80 mg), **7a** (2.0 equiv)), 32 mg of **9o** was isolated and yield is 48%.

^{1}H NMR ($CDCl_3$, 400 MHz): δ 8.79 (d, *J* = 8.4 Hz, 1 H), 8.25 (d, *J* = 7.6 Hz, 1 H), 8.12 (d, *J* = 8.4 Hz, 1 H), 7.84 (dd, *J* = 7.6, 1.6 Hz, 1 H), 7.63 – 7.54 (m, 2 H), 7.13 (s, 1 H), 3.49 (s, 3 H).

^{13}C NMR ($CDCl_3$, 100 MHz): δ 166.8, 137.4, 135.7, 130.6, 129.8, (129.25, 128.99, 128.81, 128.76) F coupling, 128.8, 128.6, (128.01, 127.93, 127.85, 127.76) F coupling, 126.2, 125.9, 125.5, 122.8, 107.1, 26.5 (Two carbon signals are missing due to overlap).

HRMS (ESI): calc. for [($C_{17}H_{10}F_3NO$)H] (M+H) 302.0793, measured 302.0794.

IR (ATR)$\tilde{\nu}$ (cm^{-1}): 3064, 2921, 2851, 1714, 1654, 1605, 1494, 1463, 1202, 1085, 905, 753.

2-Iodo-5-methyldibenzo[*cd,f*]indol-4(5*H*)-one (9p).

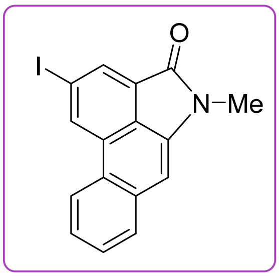

Colorless semisolid; eluent (15% ethyl acetate in hexanes). The reaction scale is 80 mg (**3r** (80 mg), **7a** (2.0 equiv)), 44 mg of **9p** was isolated and yield is 65%.

^{1}H NMR ($CDCl_3$, 400 MHz): δ 8.81 (s, 1 H), 8.35 - 8.30 (m, 2 H), 7.79 (d, *J* = 7.2 Hz, 1 H), 7.60 - 7.52 (m, 2 H), 6.98 (s, 1 H), 3.43 (s, 3 H).

^{13}C NMR ($CDCl_3$, 100 MHz): δ 166.8, 137.3, 134.9, 134.5, 132.2, 129.3, 128.7, 128.4, 128.1, 126.3, 125.9, 123.4, 105.3, 94.6, 26.5.

HRMS (ESI): calc. for [($C_{16}H_{10}INO$)H] (M+H) 359.9885, measured 359.9881.

IR (ATR)$\tilde{\nu}$ (cm^{-1}): 2926, 1703, 1646, 1391, 1308, 1020, 749.

2-Chloro-5-methyldibenzo[*cd,f*]indol-4(5*H*)-one (9q).

Greenishyellow solid; **m.p.** 185-186 °C, eluent (15% ethyl acetate in hexanes). The reaction scale is 80 mg (**3s** (80 mg), **7a** (2.0 equiv)), 43 mg of **9q** was isolated and yield is 66%.

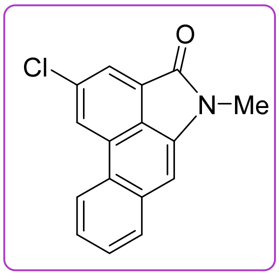

^{1}H NMR ($CDCl_3$, 400 MHz): δ 8.40 (d, J = 1.2 Hz, 1 H), 8.32 (d, J = 7.6 Hz, 1 H), 7.95 (d, J = 1.2 Hz, 1 H), 7.79 (d, J = 7.6 Hz, 1 H), 7.60 - 7.51 (m, 2 H), 6.95 (s, 1 H), 3.42 (s, 3 H).

^{13}C NMR ($CDCl_3$, 100 MHz): δ 167.0, 137.2, 135.5, 134.7, 129.3, 128.4, 127.9, 126.6, 125.9, 125.9, 125.4, 124.1, 123.4, 105.0, 26.5 (One carbon signal is missing due to overlap).

HRMS (ESI): calc. for [($C_{16}H_{10}ClNO$)Na] (M+Na) 290.0349, measured 290.0362.

IR (ATR)$\tilde{\nu}$ (cm^{-1}): 2925, 1696, 1646, 1467, 1351, 1080, 746.

8,9-Dimethoxy-5-methyldibenzo[*cd,f*]indol-4(5*H*)-one (9r).

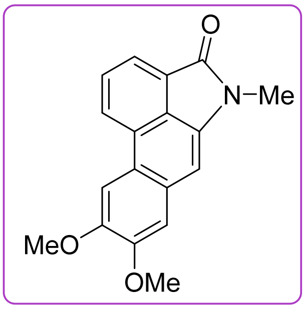

Yellow solid; **m.p.** 207-208 °C, eluent (20% ethyl acetate in hexanes). The reaction scale is 80 mg (**3j** (80 mg), **7b** (2.0 equiv)), 49 mg of **9r** was isolated and yield is 62%.

^{1}H NMR ($CDCl_3$, 400 MHz): δ 8.43 (d, J = 8.0 Hz, 1 H), 8.02 (d, J = 7.6 Hz, 1 H), 7.84 (s, 1 H), 7.76 (t, J = 7.6 Hz, 1 H), 7.23 (s, 1 H), 6.98 (s, 1 H), 4.08 (s, 3 H), 4.02 (s, 3 H), 3.46 (s, 3 H).

^{13}C NMR ($CDCl_3$, 100 MHz): δ 168.2, 149.7, 148.2, 136.6, 129.0, 128.6, 126.7, 126.5, 126.0, 125.6, 122.7, 121.5, 109.5, 104.2, 104.1, 56.1, 55.9, 26.3.

HRMS (ESI): calc. for [($C_{18}H_{15}NO_3$)H] (M+H) 294.1130, measured 294.1137.

IR (ATR)$\tilde{\nu}$ (cm^{-1}): 3003, 2938, 2845, 1738, 1693, 1646, 1500, 1252, 1060, 867, 761.

5-Methyl-[1,3]dioxolo[4',5':4,5]benzo[1,2-*f*]benzo[*cd*]indol-4(5*H*)-one (9s).

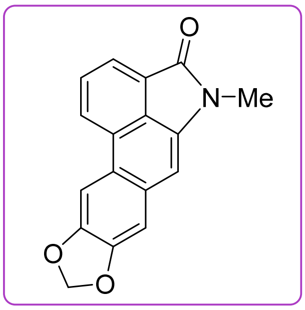

Darkyellow solid; **m.p.** 297-298 °C, eluent (25% ethyl acetate in hexanes). The reaction scale is 80 mg (**3j** (80 mg), **7c** (2.0 equiv)), 45 mg of **9s** was isolated and yield is 61%.

^{1}H NMR ($CDCl_3$, 400 MHz): δ 8.41 (d, *J* = 7.6 Hz, 1 H), 8.05 (d, *J* = 7.0 Hz, 1 H), 7.87 (s, 1 H), 7.78 (t, *J* = 7.6 Hz, 1 H), 7.25 (s, 1 H), 7.00 (s, 1 H), 6.11 (s, 2 H), 3.49 (s, 3 H).

^{13}C NMR ($CDCl_3$, 100 MHz): δ 168.4, 148.4, 147.1, 136.9, 130.6, 128.9, 127.2, 126.9, 126.2, 126.1, 123.2, 123.2, 107.1, 104.9, 101.9, 101.7, 26.5.

HRMS (ESI): calc. for [($C_{17}H_{11}NO_3$)H] (M+H) 278.0817, measured 278.0824.

IR (ATR)$\tilde{\nu}$ (cm^{-1}): 2951, 2311, 1701, 1646, 1516, 1160, 742.

5-Methyl-5,8,9,10-tetrahydro-4*H*-benzo[*cd*]indeno[5,6-*f*]indol-4-one (9t).

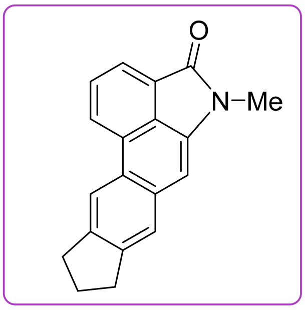

Yellow solid; **m.p.** 179-180 °C, eluent (15% ethyl acetate in hexanes). The reaction scale is 80 mg (**3j** (80 mg), **7d** (2.0 equiv)), 49 mg of **9t** was isolated and yield is 67%.

^{1}H NMR ($CDCl_3$, 400 MHz): δ 8.48 (d, *J* = 8.0 Hz, 1 H), 8.30 (s, 1 H), 8.01 (d, *J* = 7.0 Hz, 1 H), 7.74 (t, *J* = 7.6 Hz, 1 H), 7.63 (s, 1 H), 6.96 (s, 1 H), 3.45 (s, 3 H), 3.12 - 3.05 (m, 4 H), 2.22 - 2.15 (m, 2 H).

^{13}C NMR ($CDCl_3$, 100 MHz): δ 168.5, 144.9, 142.8, 136.9, 133.3, 128.8, 127.2, 126.9, 126.6, 126.4, 126.0, 124.2, 123.1, 118.4, 105.1, 33.0, 32.9, 26.4, 26.1.

HRMS (ESI): calc. for [($C_{19}H_{15}NO$)H] (M+H) 274.1232, measured 274.1236.

IR (ATR)$\tilde{\nu}$ (cm^{-1}): 2945, 1698, 1648, 1487, 1028, 876, 765.

5,8,9-Trimethyldibenzo[cd,f]indol-4(5*H*)-one (9u).

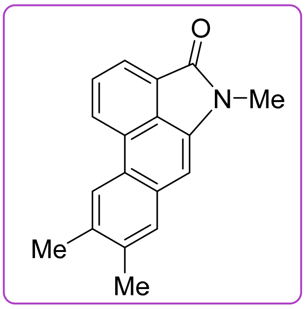

Yellow solid; **m.p.** 153-154 °C, eluent (15% ethyl acetate in hexanes). The reaction scale is 80 mg (**3j** (80 mg), **7e** (2.0 equiv)), 49 mg of **9u** was isolated and yield is 70%.

^{1}H NMR ($CDCl_3$, 400 MHz): δ 8.52 (d, *J* = 7.6 Hz, 1 H), 8.24 (s, 1 H), 8.03 (d, *J* = 7.0 Hz, 1 H), 7.77 (t, *J* = 7.6 Hz, 1 H), 7.60 (s, 1 H), 6.99 (s, 1 H), 3.48 (s, 3 H), 2.48 (s, 3 H), 2.44 (s, 3 H).

^{13}C NMR ($CDCl_3$, 100 MHz): δ 168.5, 137.2, 137.1, 134.9, 132.7, 129.6, 128.9, 127.1, 127.0, 126.7, 126.0, 125.8, 123.8, 123.2, 104.5, 26.5, 20.4, 20.3.

HRMS (ESI): calc. for [($C_{18}H_{15}NO$)H] (M+H) 262.1232, measured 262.1233.

IR (ATR)$\tilde{\nu}$ (cm^{-1}): 2993, 1699, 1644, 1515, 1165.

5-Methylbenzo[*cd*]naphtho[2,1-f]indol-4(5H)-one (9v) (major isomer).

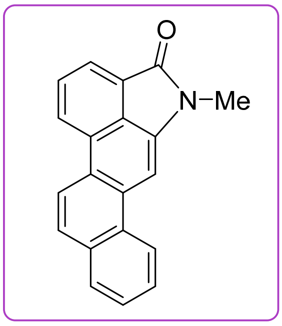

Yellow solid; eluent (15% ethyl acetate in hexanes). The reaction scale is 80 mg (**3j** (80 mg), **7f** (2.0 equiv)), 50 mg of **9v + 9v'** was isolated and yield is 66%.

^{1}H NMR ($CDCl_3$, 400 MHz): δ 9.22 (d, *J* = 8.4 Hz, 1 H), 9.12 (d, *J* = 8.4 Hz, 1 H), 8.16 (d, *J* = 7.6 Hz, 1 H), 8.01 - 7.89 (m, 3 H), 7.85 (d, *J* = 8.4 Hz, 1 H), 7.76 - 7.72 (m, 1 H), 7.64 - 7.60 (m, 1 H), 7.26 (s, 1 H), 3.55 (s, 3 H).

^{13}C NMR ($CDCl_3$, 100 MHz): δ 168.6, 138.9, 134.1, 132.9, 131.7, 131.5, 129.4, 129.1, 128.6, 128.0, 127.2, 127.2, 126.9, 125.7, 123.7, 123.7, 106.2, 26.5 (Two carbon signals are missing due to overlap).

HRMS (ESI): calc. for [($C_{20}H_{13}NO$)H] (M+H) 284.1075, measured 284.1088.

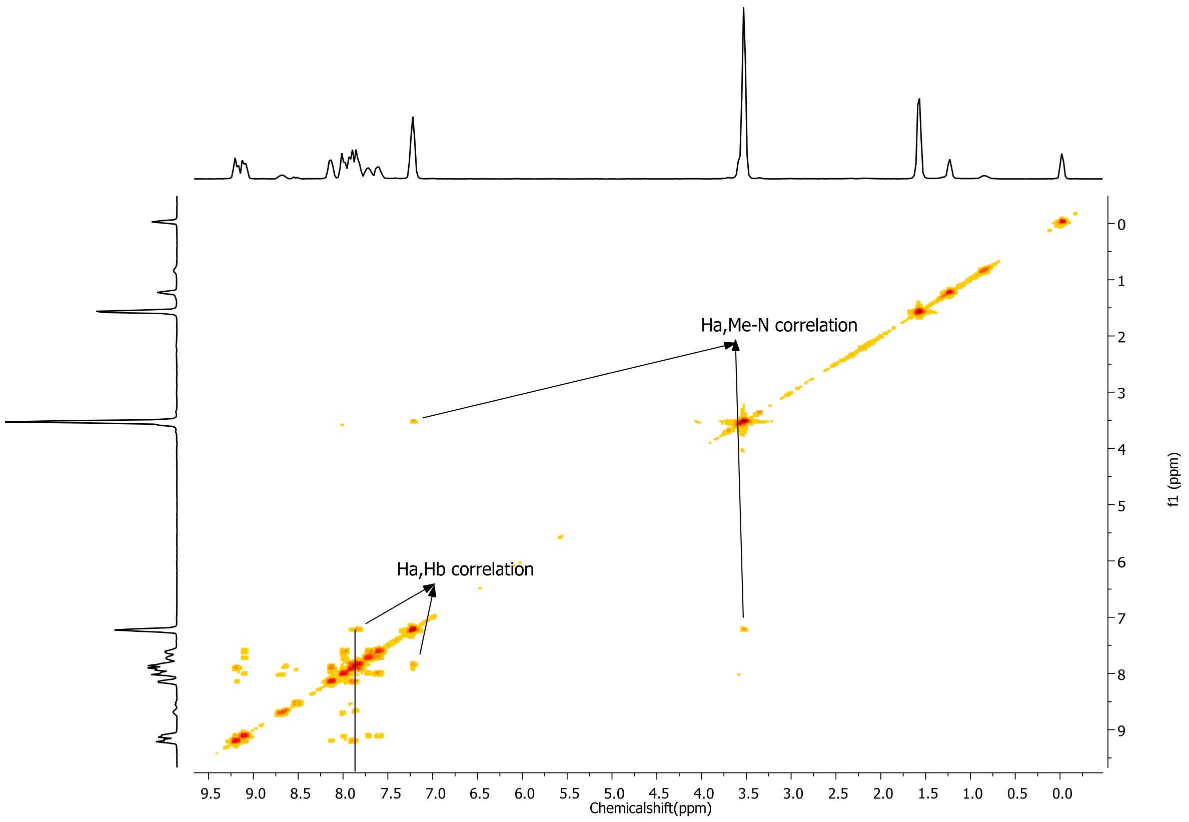

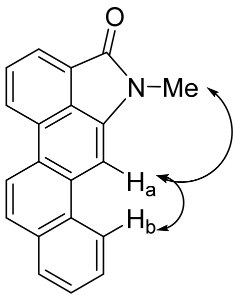

There is a NOE correlation between H_a (δ 7.29, s) and H_b (δ 7.88, d). And there is other NOE correlation between Ha (δ 7.29, s) and Me-N (δ 3.58, s). It clearly indicates that above structure is correct.

7-Methoxy-5-methyldibenzo[*cd,f*]indol-4(5*H*)-one (9w).

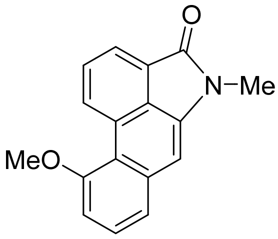

Brwon solid; **m.p.** 154-155 °C, eluent (20% ethyl acetate in hexanes). The reaction scale is 80 mg (**3j** (80 mg), **7g** (2.0 equiv)), 49 mg of **9w** was isolated and yield is 69%.

^{1}H NMR ($CDCl_3$, 400 MHz): δ 9.32 (d, *J* = 8.4 Hz, 1 H), 8.06 (d, *J* = 7.6 Hz, 1 H), 7.81 - 7.77 (m, 1 H), 7.54 - 7.48 (m, 2 H), 7.09 - 7.07 (m, 2 H), 4.14 (s, 3 H), 3.49 (s, 3 H).

^{13}C NMR ($CDCl_3$, 100 MHz): δ 159.4, 138.4, 136.5, 132.9, 129.1, 127.8, 126.7, 125.9, 123.2, 122.1, 117.6, 107.4, 105.1, 55.7, 26.4 (Two carbon signals are missing due to overlap).

HRMS (ESI): calc. for [($C_{17}H_{13}NO_2$)H] (M+H) 264.1025, measured 264.1031.

IR (ATR)$\tilde{\nu}$ (cm^{-1}): 2925, 1700, 1649, 1470, 1329, 1275, 1094, 716.

Crystallographic Data of Compound 9w

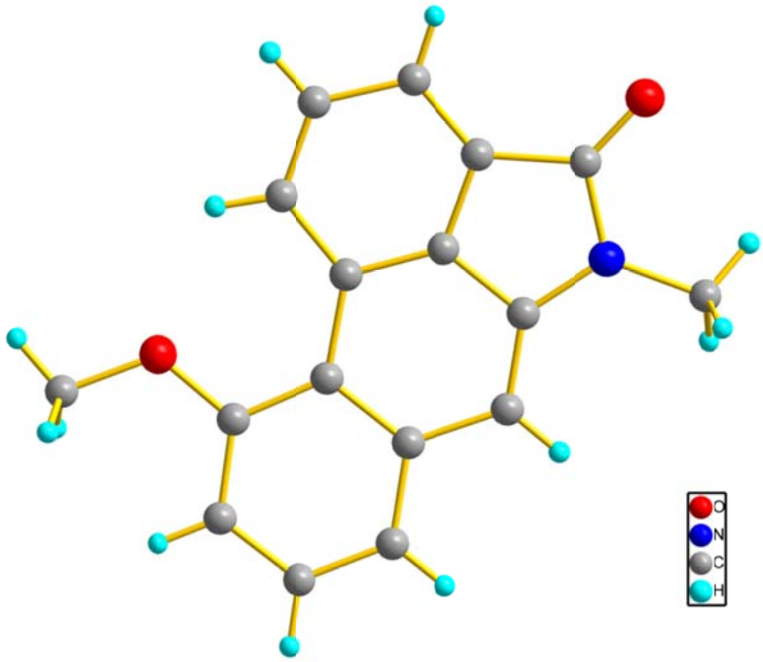

Table 1. Crystal data and structure refinement for TR1_a.

Identification code	TR1_a	
Empirical formula	C17 H13 N O2	
Formula weight	263.28	
Temperature	296(2) K	
Wavelength	0.71073 Å	
Crystal system	monoclinic	
Space group	P 21/c	
Unit cell dimensions	a = 8.3660(14) Å	a= 90°.
	b = 21.114(3) Å	b= 111.841(9) °.
	c = 7.4894(14) Å	g = 90°.
Volume	1228.0(4) $Å^3$	
Z	4	
Density (calculated)	1.424 Mg/m^3	
Absorption coefficient	0.094 mm^{-1}	
F(000)	552.0	
Theta range for data collection	2.623 to 28.550 °.	
Index ranges	-11<=h<=11, -28<=k<=28, -9<=l<=10	
Reflections collected	40781	
Independent reflections	3119 [R(int) = 0. 0581]	
Completeness to theta = 25.242°	98.7 %	
Refinement method	Full-matrix least-squares on F^2	
Data / restraints / parameters	3080 / 0 / 183	
Goodness-of-fit on F^2	0.897	
Final R indices [I>2sigma(I)]	R1 = 0.0581, wR2 = 0.1742	
R indices (all data)	R1 = 0.1164, wR2 = 0.1462	
Largest diff. peak and hole	0.228 and -0.308 e.$Å^{-3}$	

Caldensine (10a).

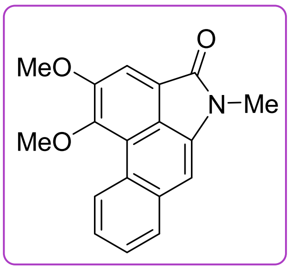

Yellowish green solid; **m.p.** 188-189 °C. (lit.[2] 189 °C, lit.[3] 193–194 °C), eluent (25% ethyl acetate in hexanes). The reaction scale is 80 mg (**3a** (80 mg), **7a** (2.0 equiv)), 41 mg of **10a** was isolated and yield is 63%.

^{1}H NMR ($CDCl_3$, 400 MHz): δ 9.21 - 9.19 (m, 1 H), 7.82 - 7.79 (m, 1 H), 7.77 (s, 1 H), 7.58 - 7.52 (m, 2 H), 6.97 (s, 1 H), 4.09 (s, 3 H), 4.05 (s, 3 H), 3.47 (s, 3 H).

^{13}C NMR ($CDCl_3$, 100 MHz): δ 168.2, 154.6, 151.3, 137.4, 134.9, 129.1, 127.8, 127.6, 127.3, 125.9, 123.2, 121.5, 120.9, 109.7, 104.2, 60.5, 57.1, 26.5.

HRMS (ESI): calc. for [($C_{18}H_{15}NO_3$)Na] (M+Na) 316.0950, measured 316.0952.

IR (ATR)$\tilde{\nu}$ (cm^{-1}): 2953, 2905, 1697, 1646, 1513, 1341.

1,2,8,9-Tetramethoxy-5-methyldibenzo[*cd,f*]indol-4(5*H*)-one (10b).

Yellow solid; **m.p.** 218-219 °C. (lit.[2] 218 °C, lit.[1] 216–217 °C), eluent (35% ethyl acetate in hexanes). The reaction scale is 80 mg (**3a** (80 mg), **7b** (2.0 equiv)), 43 mg of **10b** was isolated and yield is 55%.

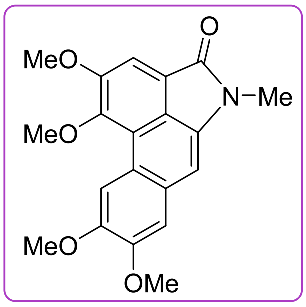

^{1}H NMR ($CDCl_3$, 400 MHz): δ 8.72 (s, 1 H), 7.71 (s, 1 H), 7.18 (s, 1 H), 6.86 (s, 1 H), 4.08 (s, 3 H), 4.05 (s, 3 H), 4.02 (s, 3 H), 4.01 (s, 3 H), 3.42 (s, 3 H).

^{13}C NMR ($CDCl_3$, 100 MHz): δ 167.9, 154.1, 150.2, 149.3, 147.9, 136.2, 129.8, 122.1, 121.6, 121.1, 120.4, 109.6, 109.1, 108.8, 103.7, 60.4, 57.0, 55.9, 26.4 (One carbon signal is missing due to overlap).

HRMS (ESI): calc. for [($C_{20}H_{19}NO_5$)H] (M+H) 354.1341, measured 354.1343.

IR (ATR)$\tilde{\nu}$ (cm^{-1}): 3000, 2941, 2848, 1737, 1691, 1505, 1403, 1296, 1250,1025, 758.

2,3-Dimethoxy-5-methyldibenzo[*cd,f*]indol-4(5*H*)-one (10c).

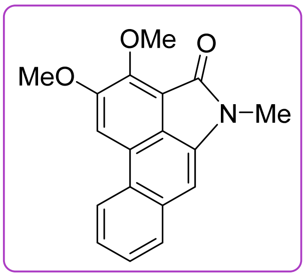

Yellow solid; **m.p.** 150-151 °C. eluent (20% ethyl acetate in hexanes). The reaction scale is 80 mg, 38 mg of **10c** was isolated and yield is 58%.

^{1}H NMR ($CDCl_3$, 400 MHz): δ 8.36 – 8.34 (m, 1 H), 7.89 (s, 1 H), 7.84 – 7.82 (m, 1 H), 7.54 – 7.49 (m, 2 H), 7.00 (s, 1 H), 4.56 (s, 3 H), 4.07 (s, 3 H), 3.45 (s, 3 H).

^{13}C NMR ($CDCl_3$, 100 MHz): δ 166.1, 153.5, 150.2, 136.8, 133.5, 128.9, 126.9, 126.6, 124.9, 122.6, 121.8, 121.3, 112.2, 108.1, 102.6, 63.3, 57.2, 26.7.

HRMS (ESI): calc. for [($C_{18}H_{15}NO_3$)H] (M+H) 294.1130, measured 294.1131.

IR (ATR)$\tilde{\nu}$ (cm^{-1}): 2996, 2948, 1691, 1647, 1492, 1375, 1061, 750.

1,2,3-Trimethoxy-5-methyldibenzo[*cd,f*]indol-4(5*H*)-one(10d).

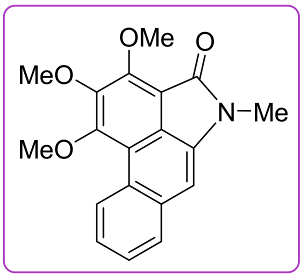

Yellow solid; **m.p.** 115-116 °C, eluent (35% ethyl acetate in hexanes). The reaction scale is 80 mg, 40 mg of **10d** was isolated and yield is 60%.

^{1}H NMR ($CDCl_3$, 400 MHz): δ 9.18 – 9.16 (m, 1 H), 7.85 – 7.82 (m, 1 H), 7.55 – 7.51 (m, 2 H), 7.08 (s, 1 H), 4.52 (s, 3 H), 4.16 (s, 3 H), 3.97 (s, 3 H), 3.47 (s, 3 H).

^{13}C NMR ($CDCl_3$, 100 MHz): δ 165.8, 156.9, 154.0, 146.4, 136.6, 133.5, 128.8, 127.0, 126.7, 126.6, 125.8, 124.7, 116.2, 108.9, 104.6, 63.2, 61.8, 60.9, 26.5.

HRMS (ESI): calc. for [($C_{19}H_{17}NO_4$)H] (M+H) 324.1236, measured 324.1238.

IR (ATR)$\tilde{\nu}$ (cm^{-1}): 2940, 1742, 1689, 1563, 1515, 1457, 1395.

2-(Benzyloxy)-1-methoxy-5-methyldibenzo[*cd,f*]indol-4(5*H*)-one (12a).

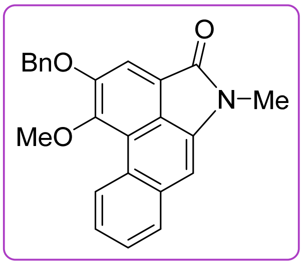

Off-white solid; **m.p.** 151-152 °C, eluent (20% ethyl acetate in hexanes). The reaction scale is 300 mg, 161 mg of **12a** was isolated and yield is 63 %.

^{1}H NMR ($CDCl_3$, 400 MHz): δ 9.23 – 9.20 (m, 1 H), 7.83 – 7.80 (m, 2 H), 7.58 – 7.50 (m, 4 H), 7.41 (t, *J* = 7.6 Hz, 2 H), 7.34 (t, *J* = 7.6 Hz, 1 H), 6.98 (s, 1 H), 5.28 (s, 2 H), 4.13 (s, 3 H), 3.46 (s, 3 H).

^{13}C NMR ($CDCl_3$, 100 MHz): δ 168.1, 153.5, 151.9, 137.3, 136.6, 134.9, 129.1, 128.86, 128.4, 127.7, 127.7, 127.6, 127.4, 126.0, 123.5, 121.4, 121.0, 111.8, 104.4, 72.2, 60.6, 26.5 (One carbon signal is missing due to overlap).

HRMS (ESI): calc. for [($C_{24}H_{19}NO_3$)H] (M+H) 370.1443, measured 370.1441.

IR (ATR)$\tilde{\nu}$ (cm^{-1}): 3054, 2927, 1696, 1458, 1312, 1183, 1018, 842, 735.

Sauristolactam (10e).

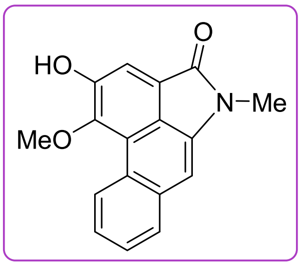

Yellow solid; **m.p.** 293-294 °C (lit.[2] 276 °C, lit.[3] > 290 °C), eluent (55% ethyl acetate in hexanes). The reaction scale is 300 mg, 218 mg of **5cg** was isolated and yield is 66%.

^{1}H NMR (DMSO-*d*$_6$, 400 MHz): δ 10.35 (s, 1 H), 9.10 (dd, *J* = 7.6, 1.6 Hz, 1 H), 7.92 (dd, *J* = 7.6, 1.6 Hz, 1 H), 7.62 (s, 1 H), 7.60 – 7.53 (m, 2 H), 7.25 (s, 1 H), 4.01 (s, 3 H), 3.36 (s, 3 H).

^{13}C NMR (DMSO-*d*$_6$, 100 MHz): δ 166.8, 152.2, 148.8, 136.9, 134.7, 129.1, 127.5, 126.9, 126.3, 125.6, 120.9, 120.2, 113.6, 103.5, 59.5, 26.2 (One carbon signal is missing due to overlap).

HRMS (ESI): calc. for [($C_{17}H_{13}NO_3$)H] (M+H) 280.0974, measured 280.0983.

IR (ATR)$\tilde{\nu}$ (cm^{-1}): 2926, 2856, 2311, 1697, 1645, 1509, 1448, 1149, 750.

1-(Benzyloxy)-2-methoxy-5-methyldibenzo[*cd,f*]indol-4(5*H*)-one (12b).

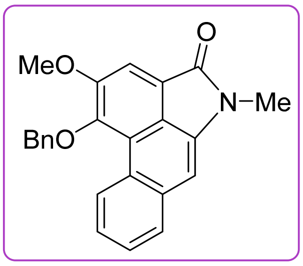

Greenish yellow solid; eluent (20% ethyl acetate in hexanes). The reaction scale is 300 mg, 158 mg of **12b** was isolated and yield is 62%.

^{1}H NMR (CDCl$_3$, 400 MHz): δ 9.22 (d, *J* = 8.0 Hz, 1 H), 7.77 – 7.73 (m, 2 H), 7.57 (d, *J* = 7.2 Hz, 2 H), 7.51 (t, *J* = 7.2 Hz, 1 H), 7.43 – 7.34 (m, 4 H), 6.92 (s, 1 H), 5.22 (s, 2 H), 4.01 (s, 3 H), 3.44 (s, 3 H).

^{13}C NMR (CDCl$_3$, 100 MHz): δ 168.1, 154.4, 149.8, 137.2, 137.1, 134.8, 129.0, 128.7, 128.4, 127.9, 127.5, 127.2, 125.8, 123.0, 121.4, 121.0, 109.5, 104.2, 74.9, 56.9, 26.4 (Three carbon signals are missing due to overlap).

HRMS (ESI): calc. for [($C_{24}H_{19}NO_3$)H] (M+H) 370.1443, measured 370.1446.

N-Methyl piperolactam A (10f).

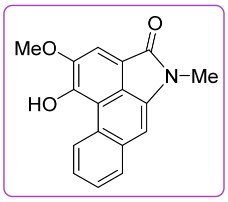

Grey solid; eluent (55% ethyl acetate in hexanes). **m.p.** 204-205 °C (lit.[2] 201 °C, lit.[6] 249-251 °C), The reaction scale is 300 mg, 220 mg of **10f** was isolated and yield is 97%.

^{1}H NMR (DMSO-*d*$_6$, 400 MHz): δ 10.68 (s, 1 H), 9.25 – 9.22 (m, 1 H), 7.90 – 7.87 (m, 1 H), 7.75 (s, 1 H), 7.55 – 7.53 (m, 2 H), 7.22 (s, 1 H), 4.03 (s, 3 H), 3.36 (s, 3 H).

^{13}C NMR (DMSO-*d*$_6$, 100 MHz): δ 167.2, 149.3, 148.1, 136.7, 133.9, 128.7, 127.5, 126.9, 126.7, 125.1, 122.9, 115.1, 114.2, 108.7, 103.8, 57.9, 26.0.

HRMS (ESI): calc. for [($C_{17}H_{13}NO_3$)H] (M+H) 280.0974, measured 280.0981.

IR (ATR)$\tilde{\nu}$ (cm^{-1}): 2387, 1685, 1645, 1514, 1421, 1364.

1,2-Dimethoxy-5-(4-methoxybenzyl)dibenzo[*cd,f*]indol-4(5*H*)-one (11c).

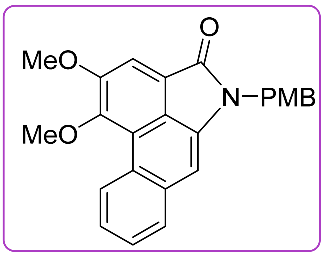

Yellow solid; **m.p.** 190-191 °C; eluent (25% ethyl acetate in hexanes). The reaction scale is 80 mg, 46 mg of **11c** was isolated and yield is 66%.

^{1}H NMR ($CDCl_3$, 400 MHz): δ 9.19 – 9.17 (m, 1 H), 7.81 (s, 1 H), 7.73 – 7.71 (m, 1 H), 7.52 – 7.50 (m, 1 H), 7.30 (d, *J* = 8.4 Hz, 2 H), 6.87 (s, 1 H), 6.82 (d, *J* = 8.4 Hz, 2 H), 5.09 (s, 2 H), 4.08 (s, 3 H), 4.06 (s, 3 H), 3.74 (s, 3 H).

^{13}C NMR ($CDCl_3$, 100 MHz): δ 167.9, 159.2, 154.6, 151.5, 136.4, 134.8, 129.2, 128.9, 127.7, 127.6, 127.2, 126.0, 123.4, 121.2, 120.9, 114.3, 109.9, 105.3, 60.5, 57.1, 55.4, 43.6 (Three carbon signals are missing due to overlap).

HRMS (ESI): calc. for [($C_{25}H_{21}NO_4$)H] (M+H) 400.1549, measured 400.1556.

IR (ATR)$\tilde{\nu}$ (cm^{-1}): 2942, 2837, 1698, 1646, 1404, 1243, 1076, 996, 752.

Cepharanone B (aristolactam BII) (10g).

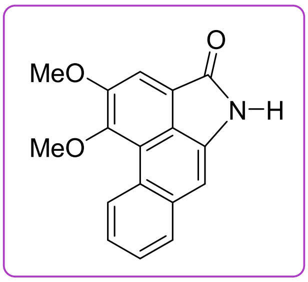

Light yellow solid; **m.p.** 252-253 °C. (lit.[2] 254 °C, lit.[4] 249-251), eluent (55% ethyl acetate in hexanes). The reaction scale is 100 mg, 65 mg of **10g** was isolated and yield is 92%.

^{1}H NMR (DMSO-d_6, 400 MHz): δ 10.87 (bs, 1 H), 9.1 (d, *J* = 7.6 Hz, 1 H), 7.93 (d, *J* = 7.6 Hz, 1 H), 7.84 (s, 1 H), 7.60 – 7.53 (m, 2 H), 7.14 (s, 1 H), 4.03 (s, 3 H), 4.02 (s, 3 H).

^{13}C NMR (DMSO-d_6, 100 MHz): δ 168.5, 154.3, 150.5, 135.2, 134.9, 129.1, 127.6, 126.9, 126.0, 125.6, 123.4, 121.6, 120.0, 109.9, 104.8, 60.0, 57.0.

HRMS (ESI): calc. for [($C_{17}H_{13}NO_3$)H] (M+H) 280.0974, measured 280.0977.

IR (ATR)$\tilde{\nu}$ (cm^{-1}): 2923, 2855, 1698, 1512, 1462, 1262, 1025, 741.

1,2,8,9-Tetramethoxy-5-(4-methoxybenzyl)dibenzo[*cd,f*]indol-4(5*H*)-one (11d).

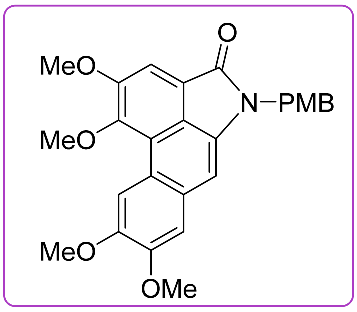

yellow solid; eluent (35% ethyl acetate in hexanes). The reaction scale is 80 mg, 79 mg of **11d** was isolated and yield is 61%.

^{1}H NMR ($CDCl_3$, 400 MHz): δ 8.73 (s, 1 H), 7.79 (s, 1 H), 7.30 (d, *J* = 8.4 Hz, 2 H), 7.12 (s, 1 H), 6.83 – 6.80 (m, 3 H), 5.07 (s, 2 H), 4.08 (s, 3 H), 4.05 (s, 3 H), 4.04 (s, 3 H), 3.98 (s, 3 H), 3.74 (s, 3 H).

^{13}C NMR ($CDCl_3$, 100 MHz): δ 167.9, 159.1, 154.2, 150.5, 149.4, 148.0, 135.3, 129.8, 129.4, 128.8, 122.5, 121.4, 121.1, 120.7, 114.3, 109.8, 109.5, 108.9, 104.8, 60.5, 57.2, 56.0, 55.9, 55.4, 43.6 (Two carbon signals are missing due to overlap).

HRMS (ESI): calc. for [($C_{27}H_{25}NO_6$)H] (M+H) 460.1760, measured 460.1757.

IR (ATR)$\tilde{\nu}$ (cm^{-1}): 2946, 2836, 1689, 1506, 1403, 1032, 864.

Norcepharanone (10h).

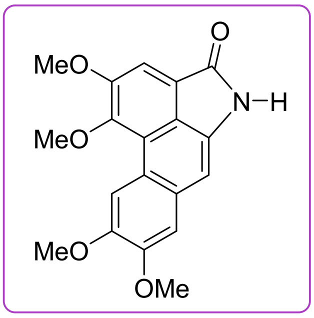

Yellow solid; eluent (65% ethyl acetate in hexanes). **m.p.** 209-210 °C (lit.[5] 208-209 °C), The reaction scale is 100 mg, 67 mg of **10h** was isolated and yield is 91%.

^{1}H NMR (DMSO-d_6, 400 MHz): δ 10.76 (bs, 1 H), 8.61 (s, 1 H), 7.78 (s, 1 H), 7.50 (s, 1 H), 7.09 (s, 1 H), 4.04 (s, 3 H), 4.03 (s, 3 H), 3.93 (s, 3 H), 3.91 (s, 3 H).

^{13}C NMR (DMSO-d_6, 100 MHz): δ 168.3, 153.8, 149.5, 149.1, 147.3, 133.7, 129.9, 122.5, 121.7, 119.8, 119.6, 110.2, 109.2, 108.2, 104.5, 59.9, 56.9, 55.5, 55.3.

HRMS (ESI): calc. for [($C_{19}H_{17}NO_5$)H] (M+H) 340.1185, measured 340.1186.

IR (ATR)$\tilde{\nu}$ (cm^{-1}): 2950, 1685, 1503, 1465, 1353, 1314, 1215.

1,2,3-Trimethoxy-5-(4-methoxybenzyl)dibenzo[*cd,f*]indol-4(5*H*)-one (11e).

Yellow solid; eluent (30% ethyl acetate in hexanes). The reaction scale is 80 mg, 45 mg of **11e** was isolated and yield is 65%.

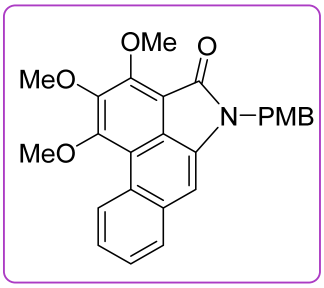

^{1}H NMR ($CDCl_3$, 400 MHz): δ 9.16 (d, J = 8.4 Hz, 1 H), 7.76 (d, J = 7.2 Hz, 1 H), 7.53 – 7.47 (m, 2 H), 7.31 (d, J = 8.4 Hz, 2 H), 7.01 (s, 1 H), 6.82 (d, J = 8.4 Hz, 2 H), 5.10 (s, 2 H), 4.55 (s, 3 H), 4.16 (s, 3 H), 4.98 (s, 3 H), 3.74 (s, 3 H).

^{13}C NMR ($CDCl_3$, 100 MHz): δ 165.7, 159.1, 157.1, 154.2, 146.5, 135.7, 133.4, 129.2, 128.9, 128.9, 126.9, 126.6, 126.6, 125.8, 124.9, 116.3, 114.3, 108.8, 105.7, 63.2, 61.8, 60.9, 55.4, 43.6 (Two carbon signals are missing due to overlap).

HRMS (ESI): calc. for [($C_{26}H_{23}NO_5$)H] (M+H) 430.1654, measured 430.1652.

IR (ATR)$\tilde{\nu}$ (cm^{-1}): 2928, 2851, 1692, 1645, 1513, 1462, 1389, 1040, 738.

Piperolactam C (10i).

Yellow color solid; **m.p.** 190-191 °C, eluent (65% ethyl acetate in hexanes). The reaction scale is 100 mg, 65 mg of **10i** was isolated and yield is 90%.

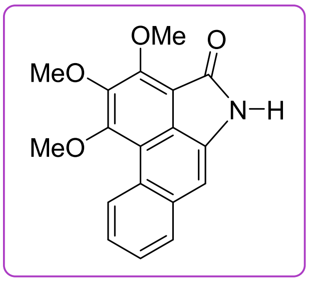

^{1}H NMR (DMSO-d_6, 400 MHz): δ 10.98 (bs, 1 H), 9.09 – 9.07 (m, 1 H), 7.97 – 7.95 (m, 1 H), 7.55 – 7.53 (m, 2 H), 7.25 (s, 1 H), 4.39 (s, 3 H), 4.11 (s, 3 H), 3.91 (s, 3 H).

^{13}C NMR (DMSO-d_6, 100 MHz): δ 166.3, 156.1, 153.3, 146.0, 134.4, 133.4, 128.8, 126.5, 125.9, 125.6, 125.4, 125.1, 115.5, 109.6, 105.3, 62.5, 61.4, 60.8.

HRMS (ESI): calc. for [($C_{18}H_{15}NO_4$)H] (M+H) 310.1079, measured 310.1085.

IR (ATR)$\tilde{\nu}$ (cm^{-1}): 2943, 1690, 1604,1518, 1461, 1386, 1314, 1121, 1083, 752.

References

(1) Castedo, L.; Suau, R.; Mouriño, A. *Tetrahedron Lett.* **1976**, *6*, 501.

(2) Kim, J. K.; Kim, Y. H.; Nam, H. T.; Kim, B. T.; Heo, J.-N. *Org. Lett.* **2008**, *10*, 3543.

(3) Rao, K. V.; Reddy, G. C. S. *J. Nat. Prod.* **1990**, *53*, 309–312.

(4) Castedo, L.; Guitian, E.; Saa, J. M.; Suau, R. *Heterocycles* **1982**, *19*, 279–280.

(5) Estevez, J.C.; Villaverde, M. C.; Estevez, R.J.; Castedo, L. *Tetrahedron* **1995**, *51*, 4075.

(6) Ruangrungsi, N.; Prathanturarug, S.; Lange, G. L.; Organ, M. G. *Phytochemistry* **1992**, *31*, 2397.

Spectral Data of Compound **3a.**

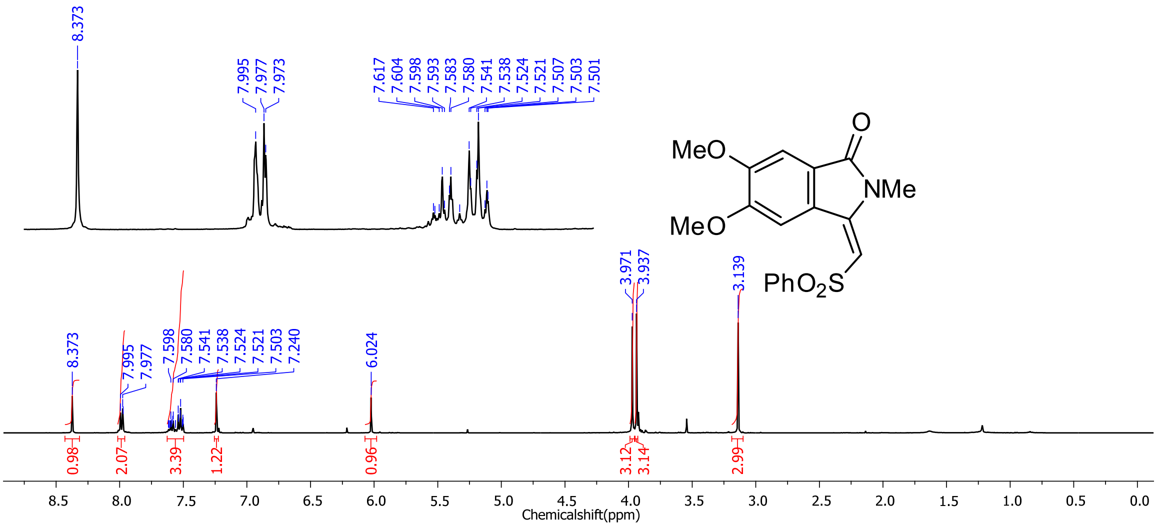

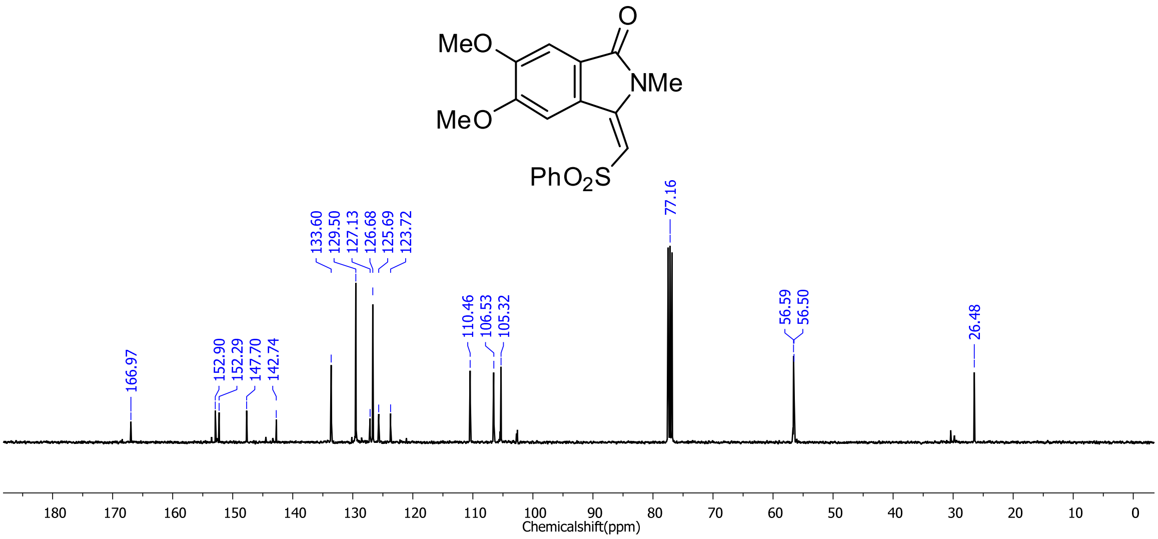

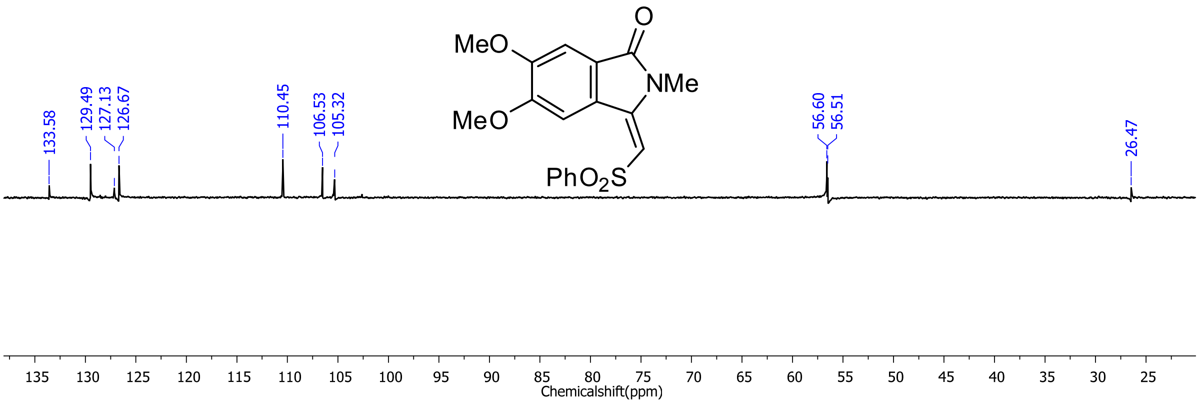

Spectral Data of Compound **3b.**

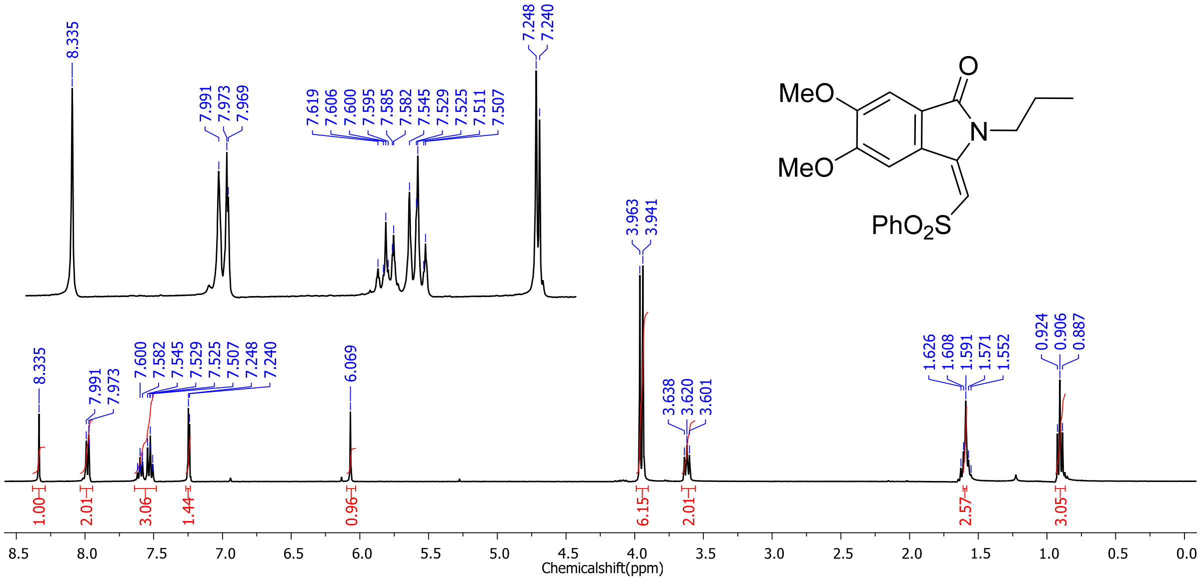

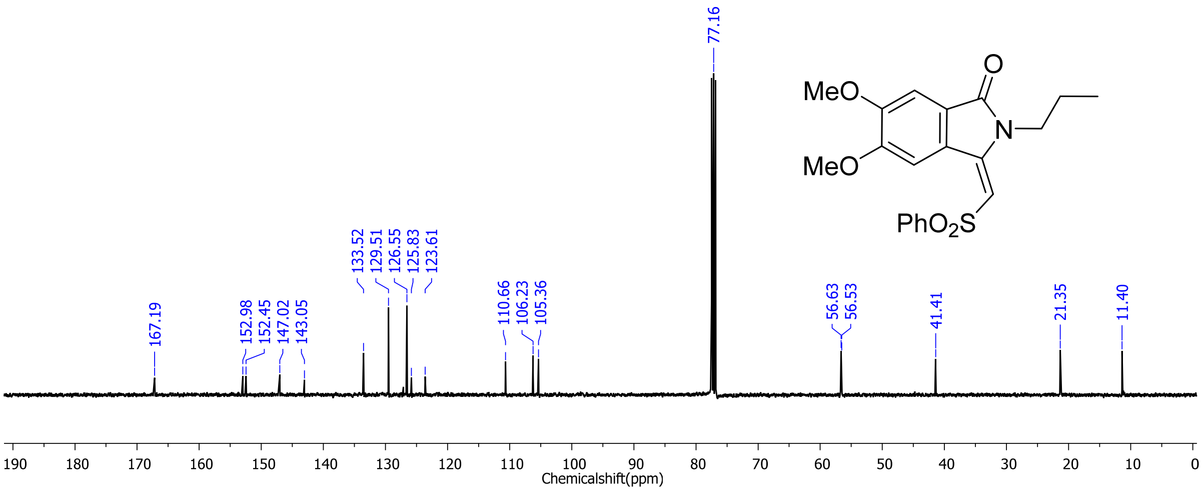

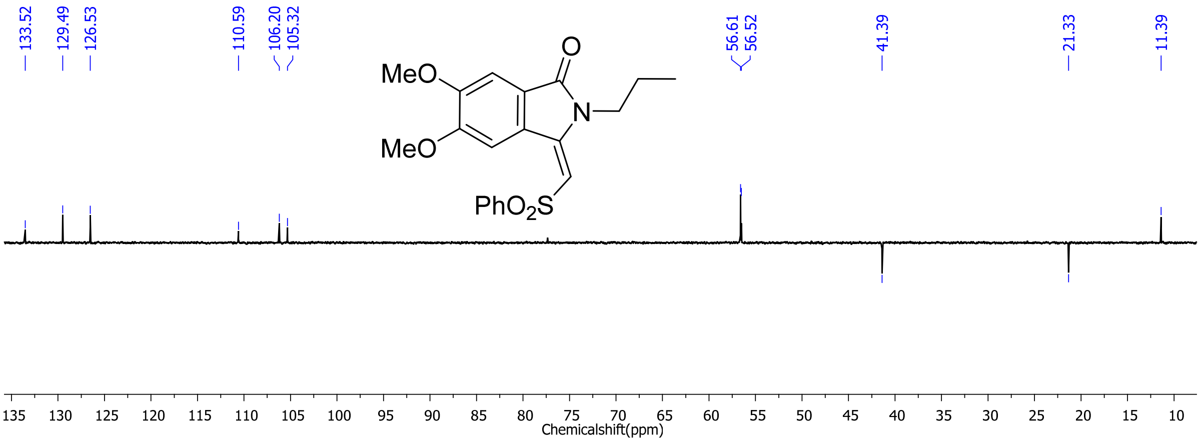

Spectral Data of Compound **3c.**

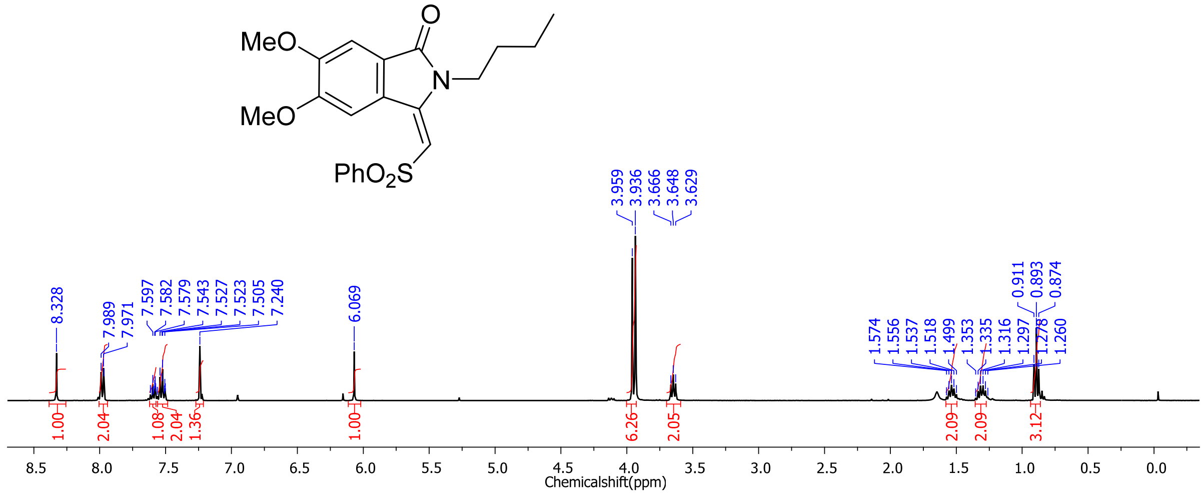

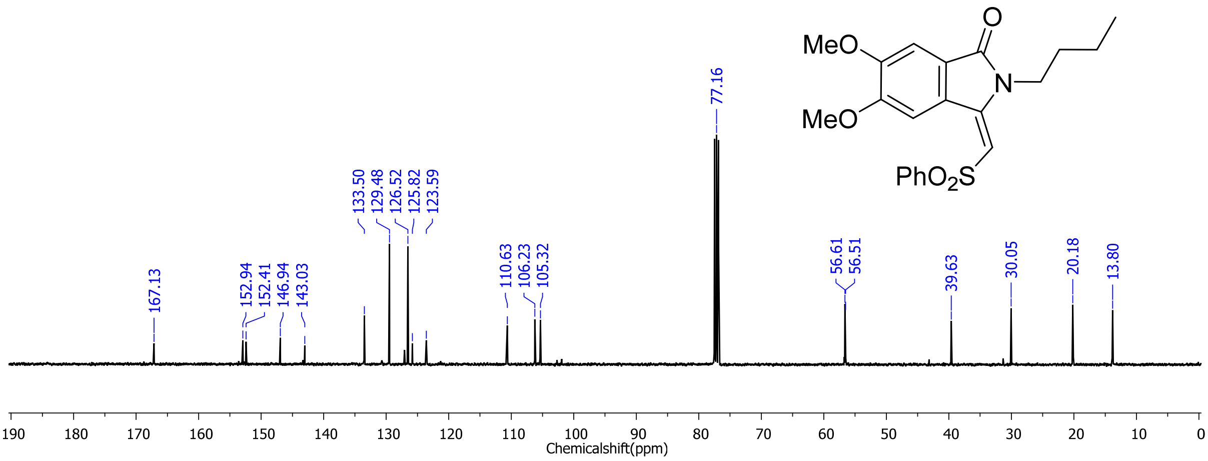

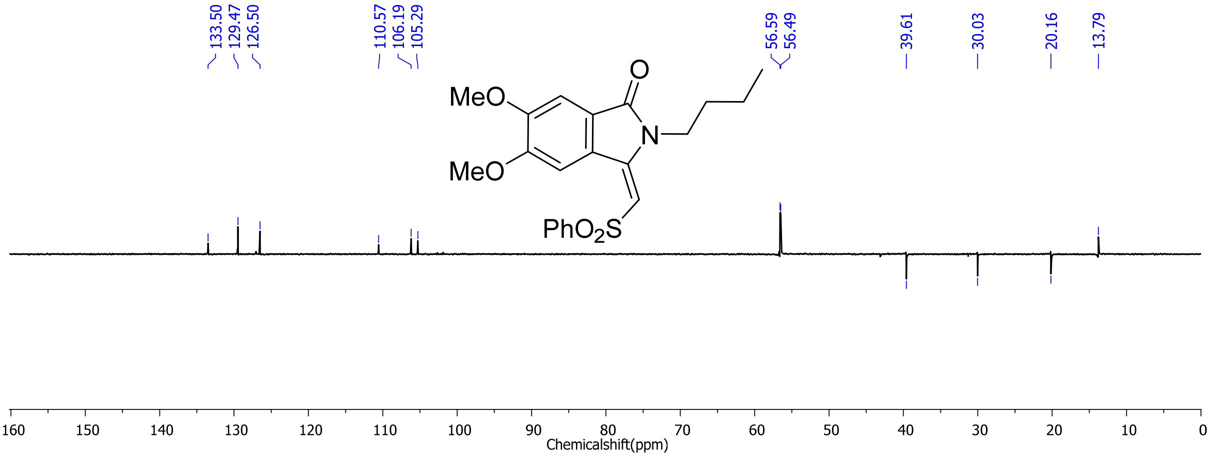

Spectral Data of Compound **3d.**

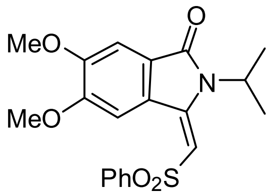

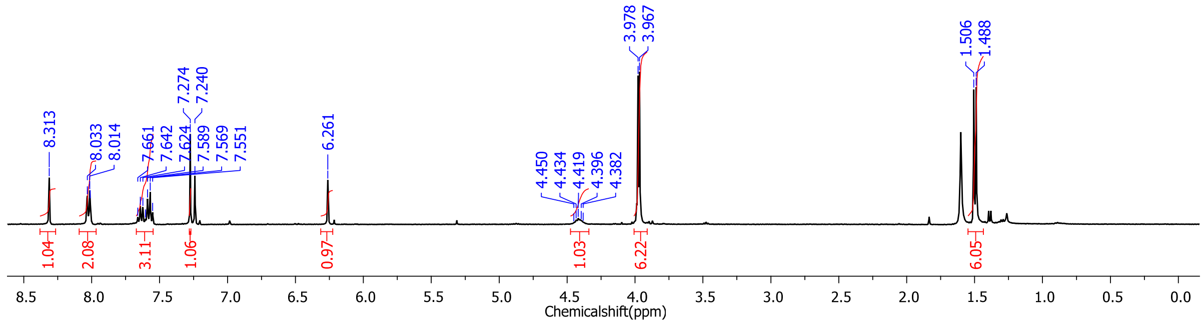

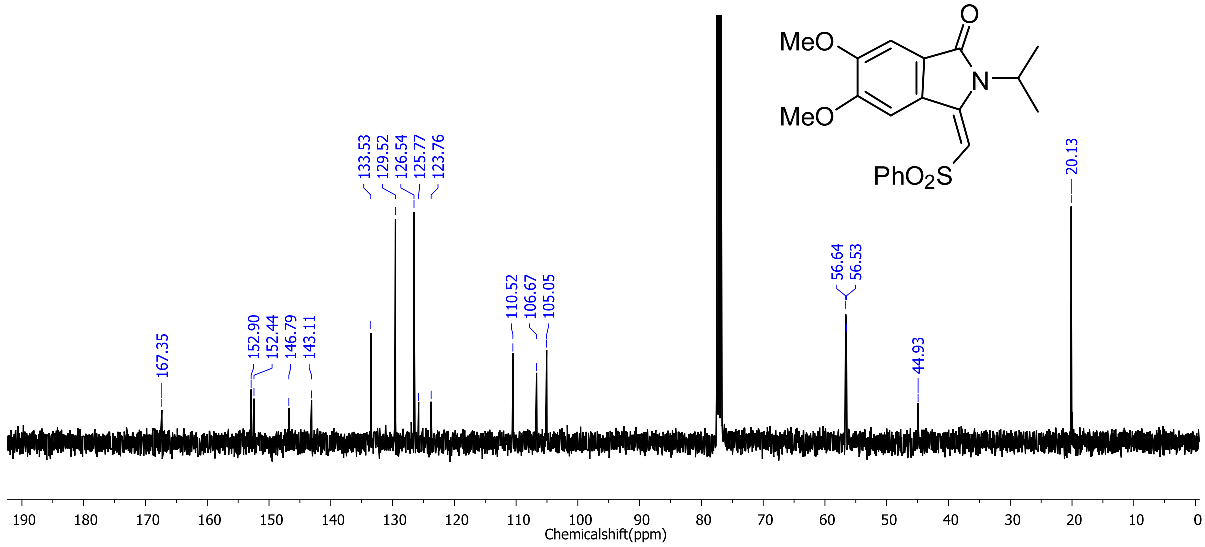

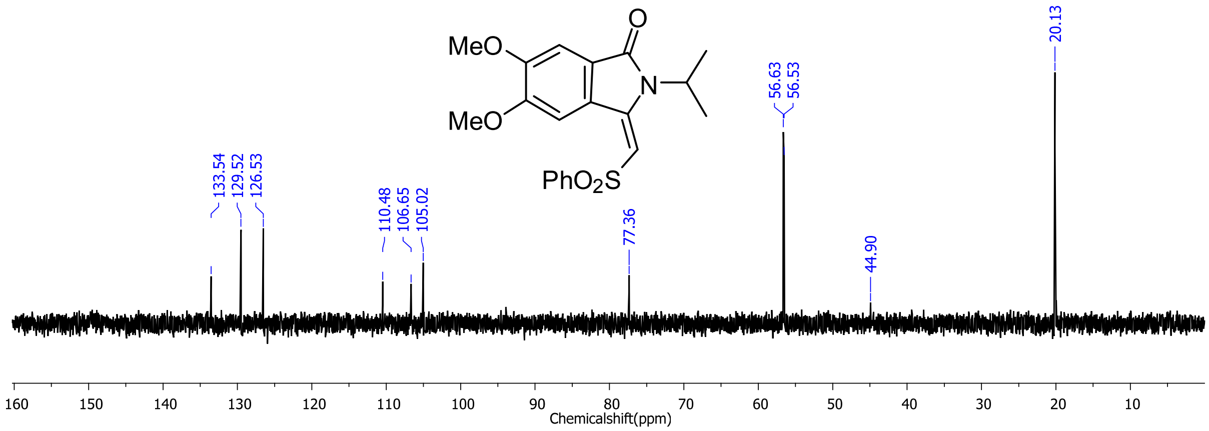

Spectral Data of Compound **3e.**

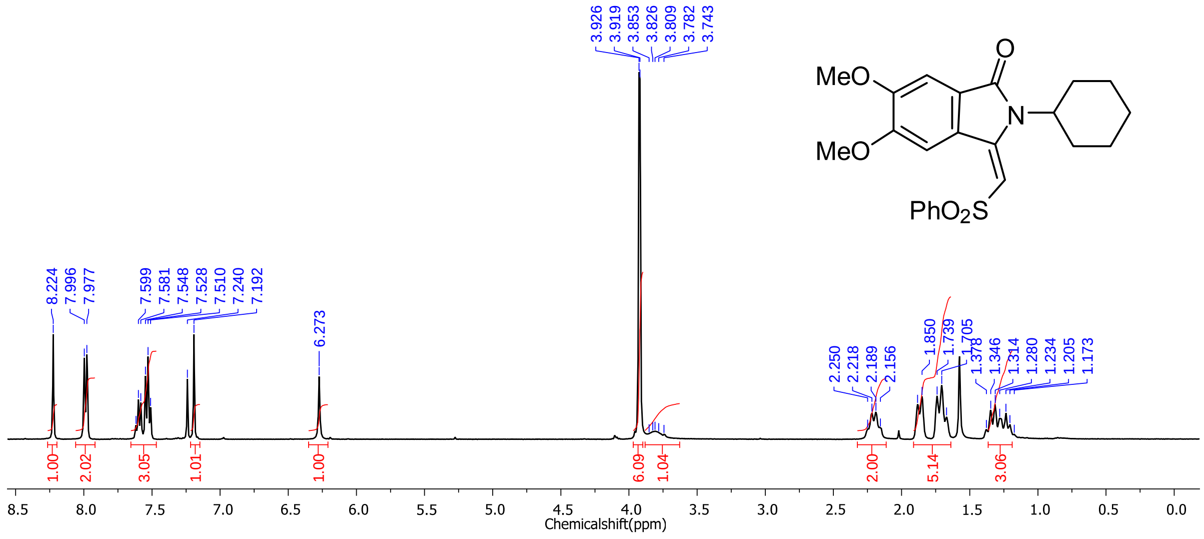

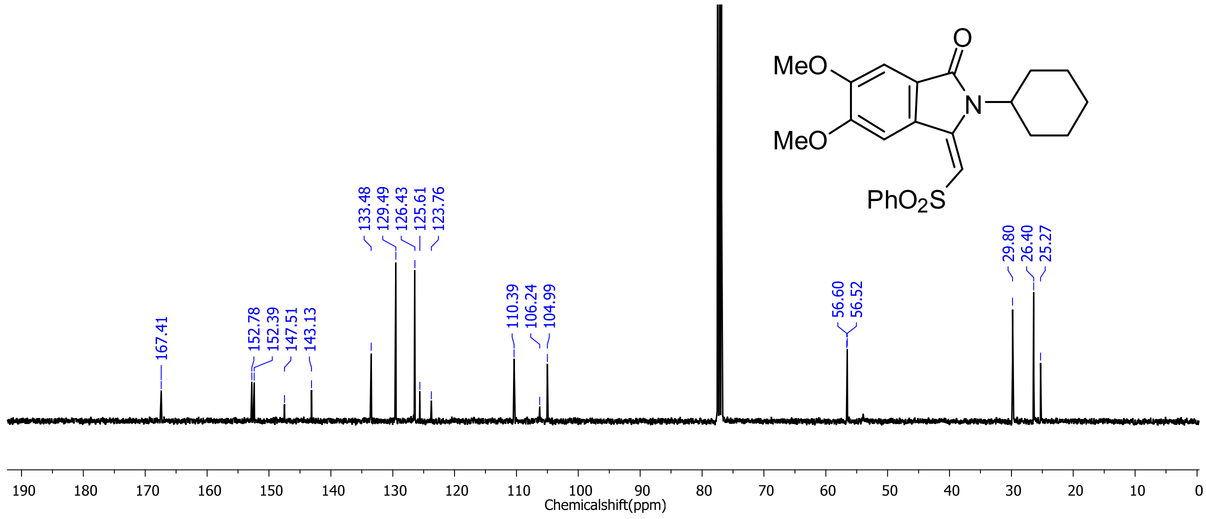

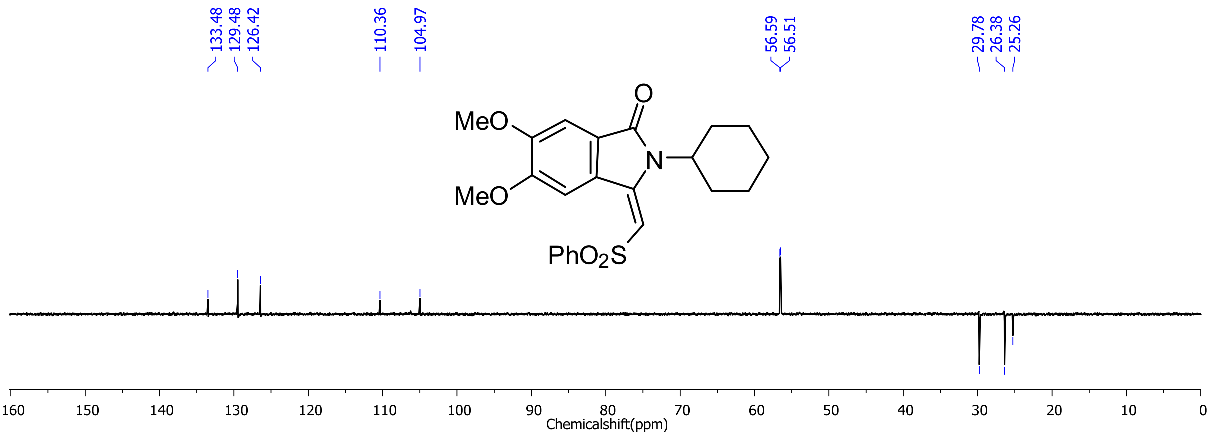

Spectral Data of Compound **3f.**

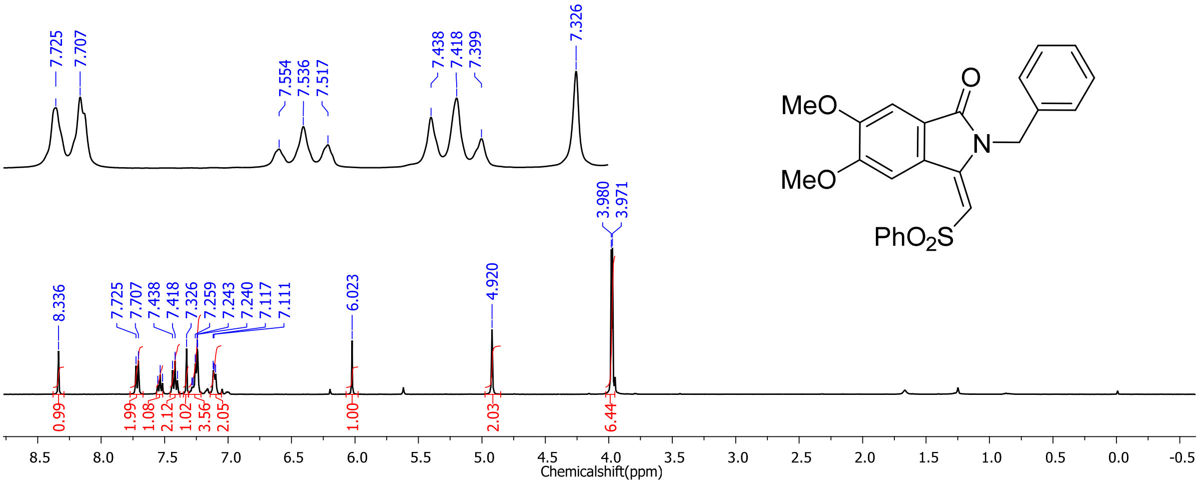

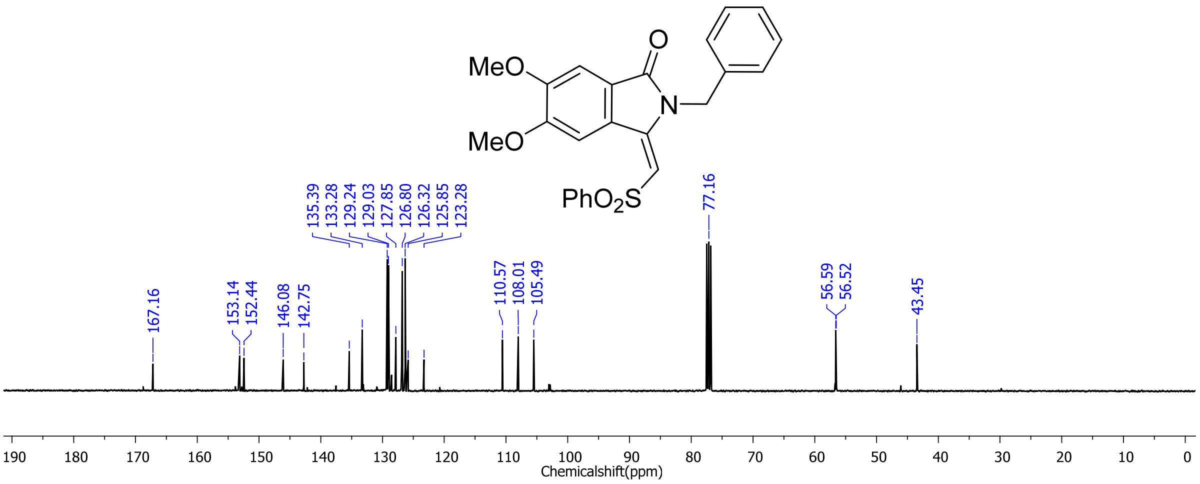

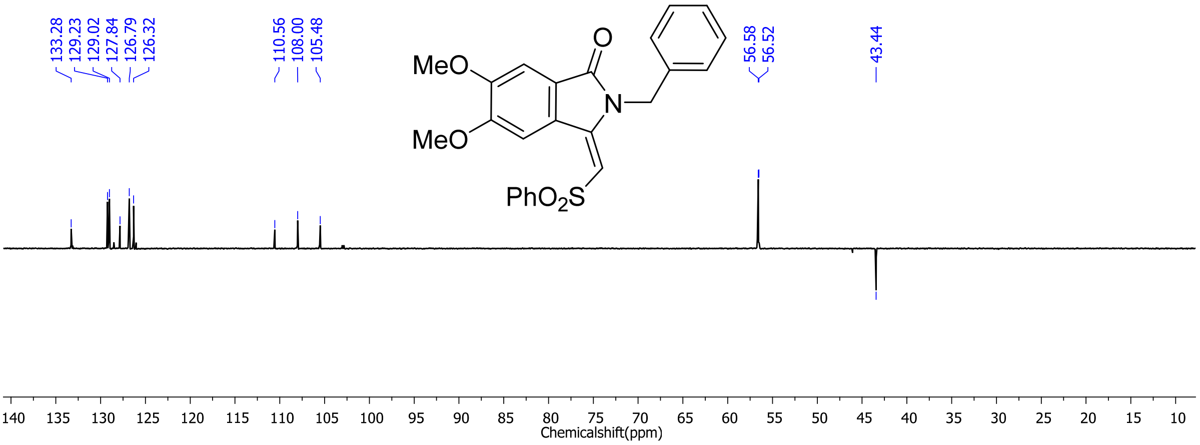

Spectral Data of Compound **3g.**

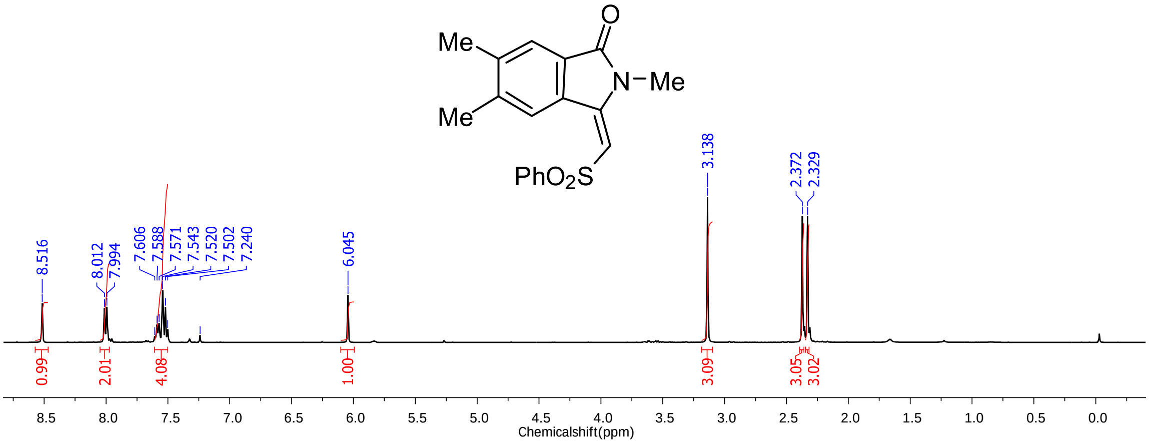

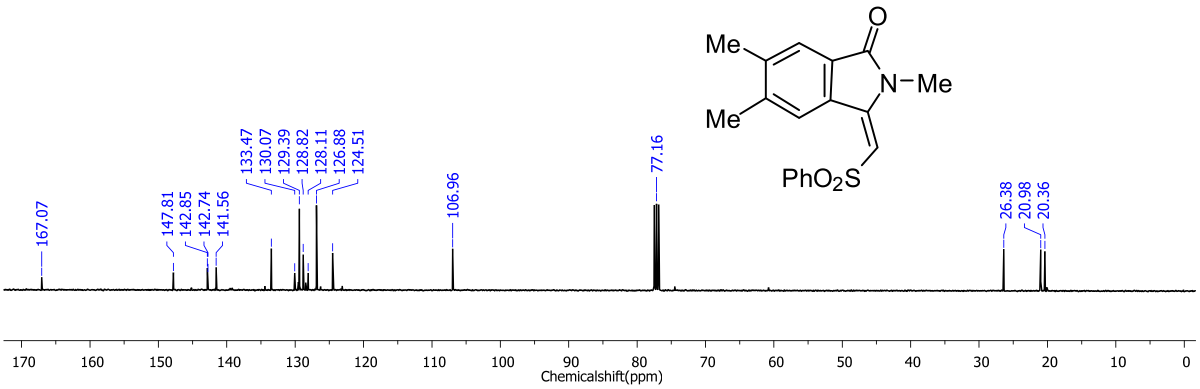

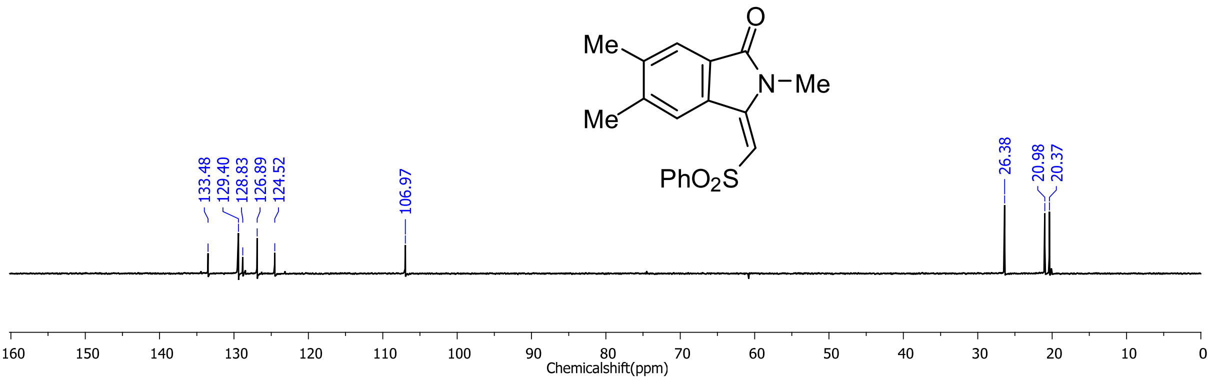

Spectral Data of Compound **3h.**

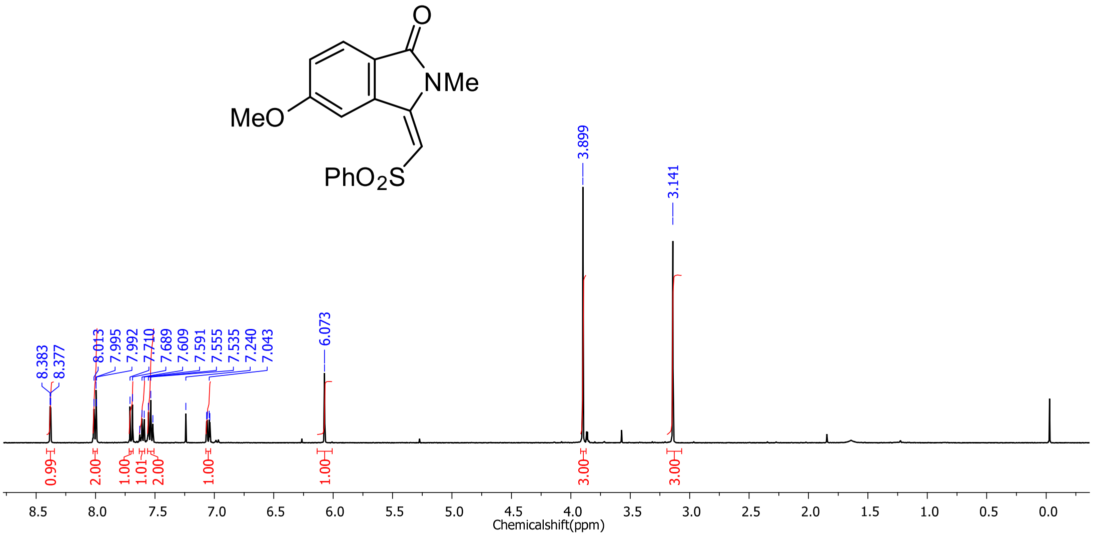

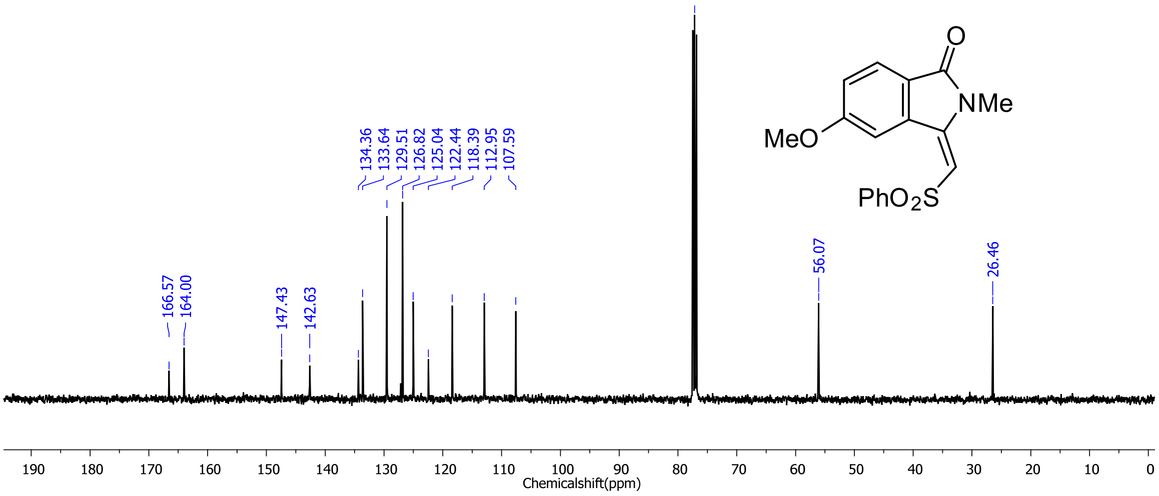

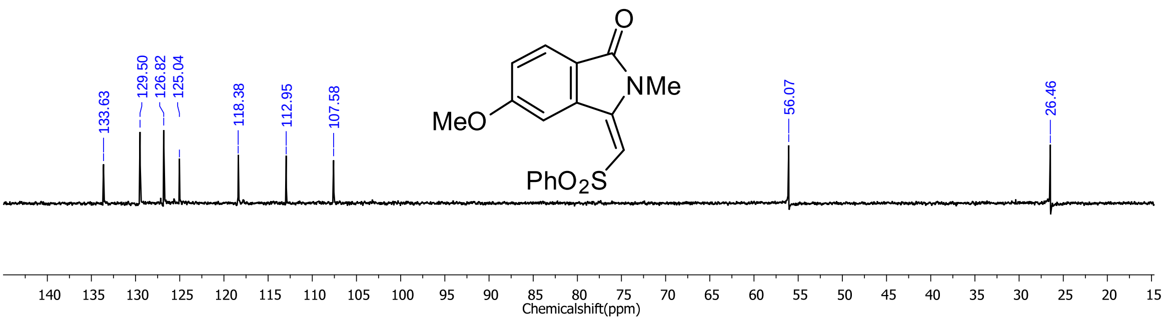

Spectral Data of Compound **3i.**

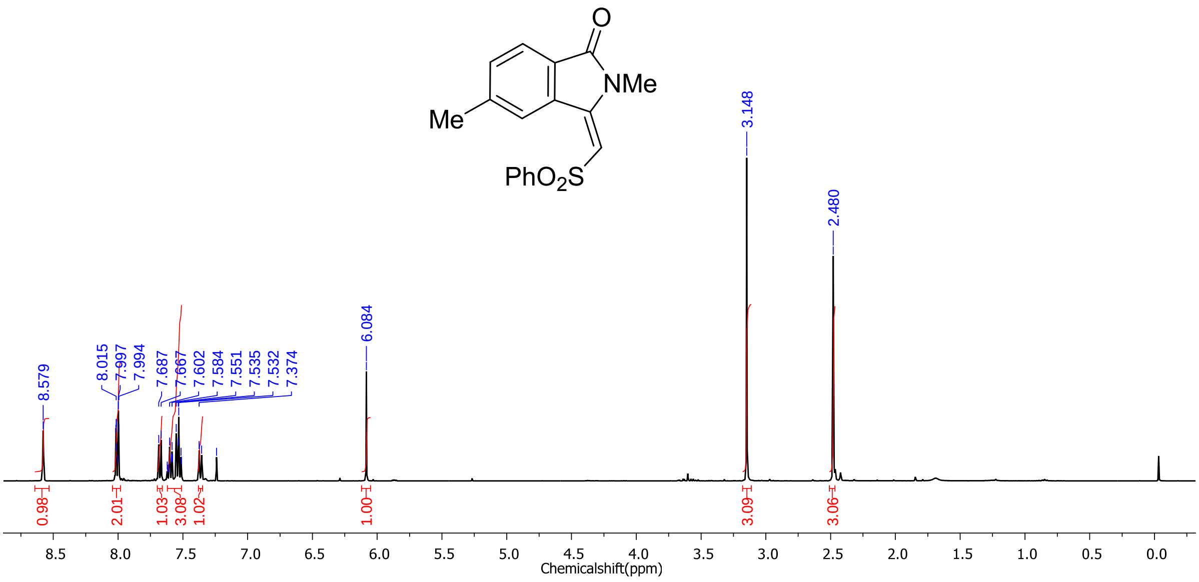

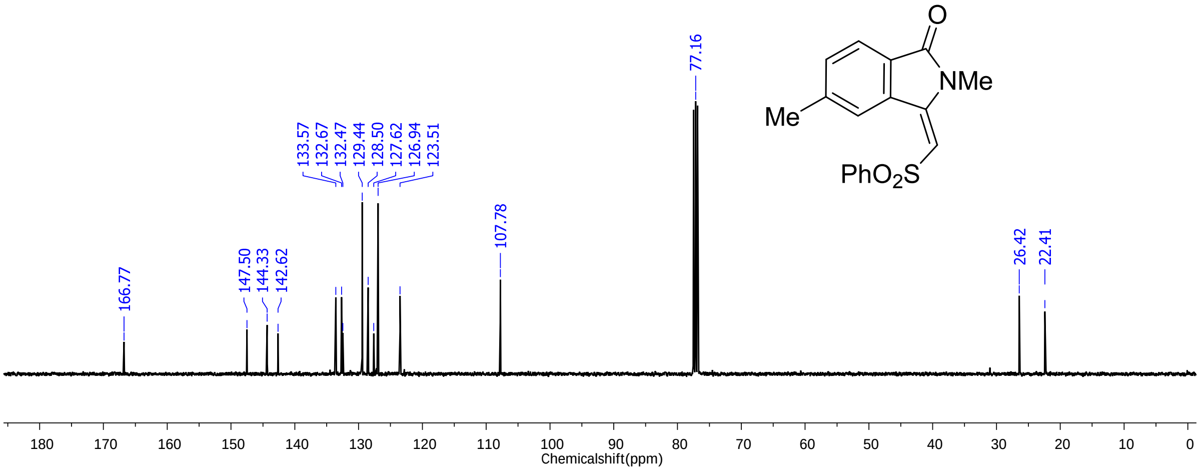

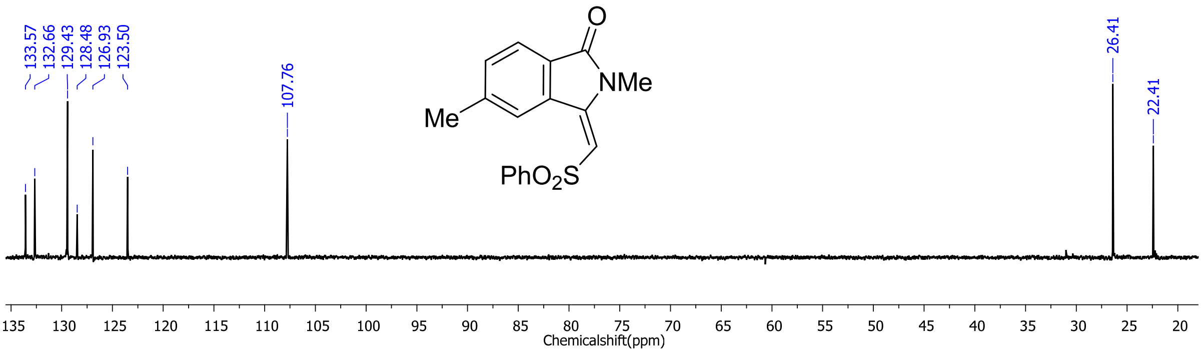

Spectral Data of Compound **3j.**

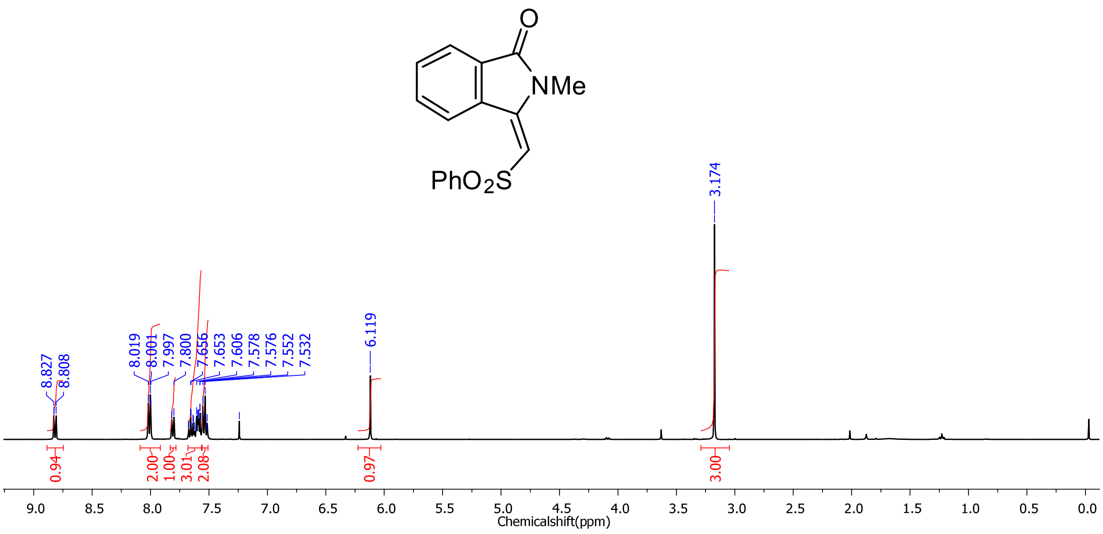

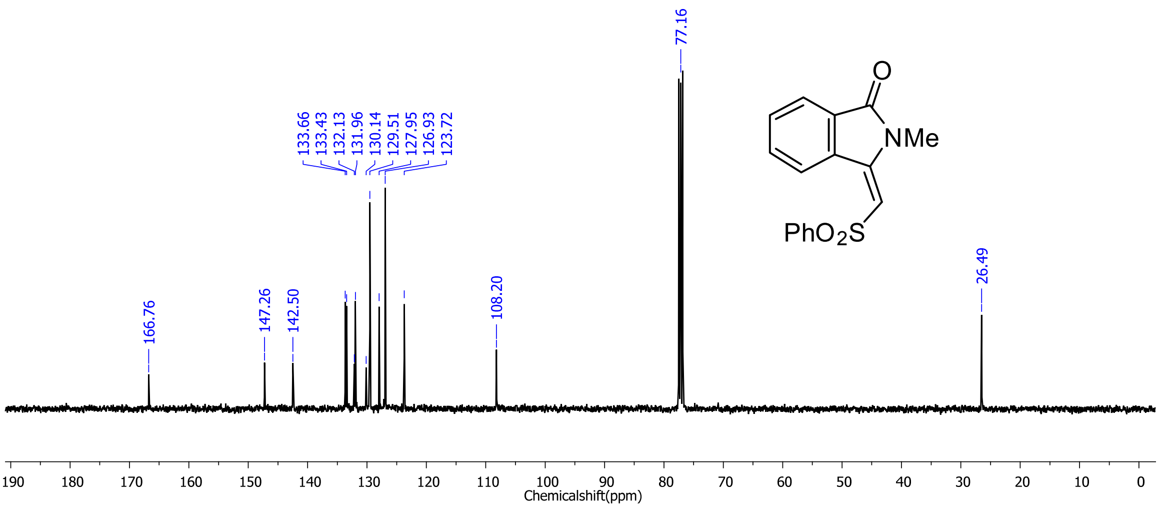

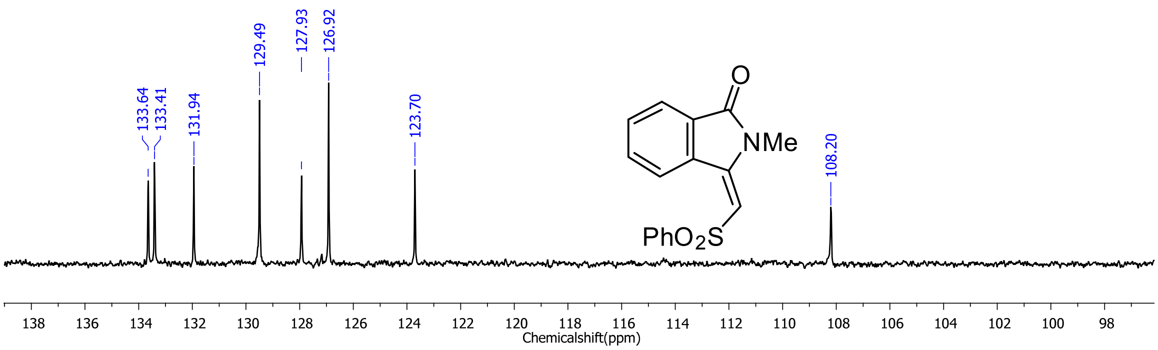

Spectral Data of Compound **3k.**

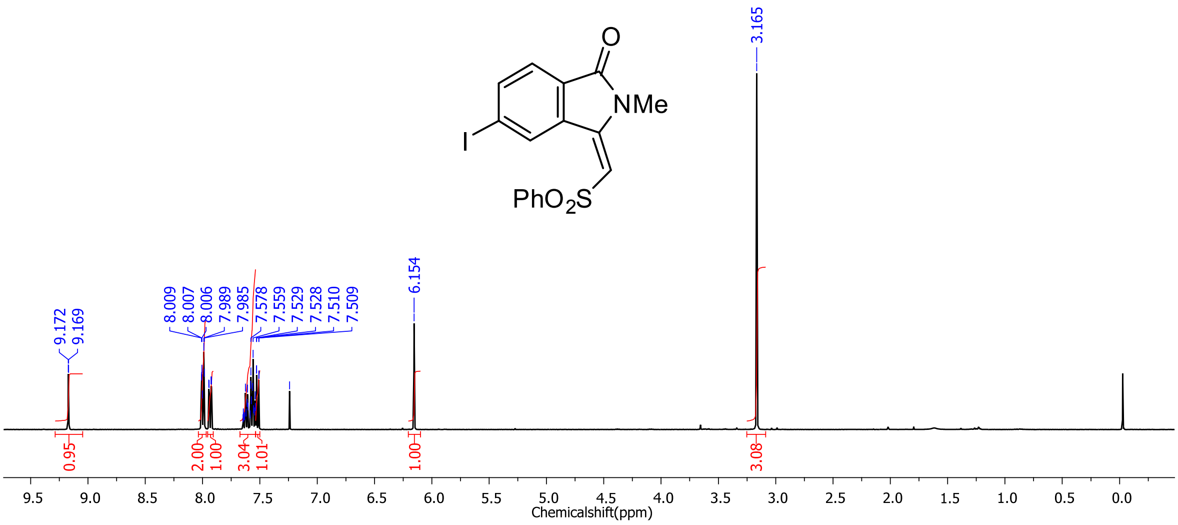

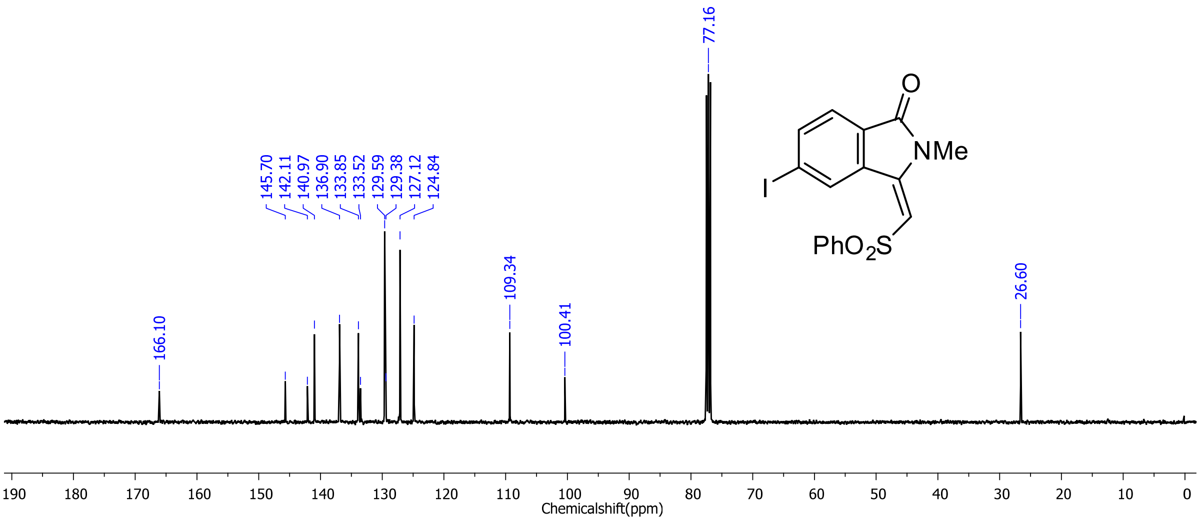

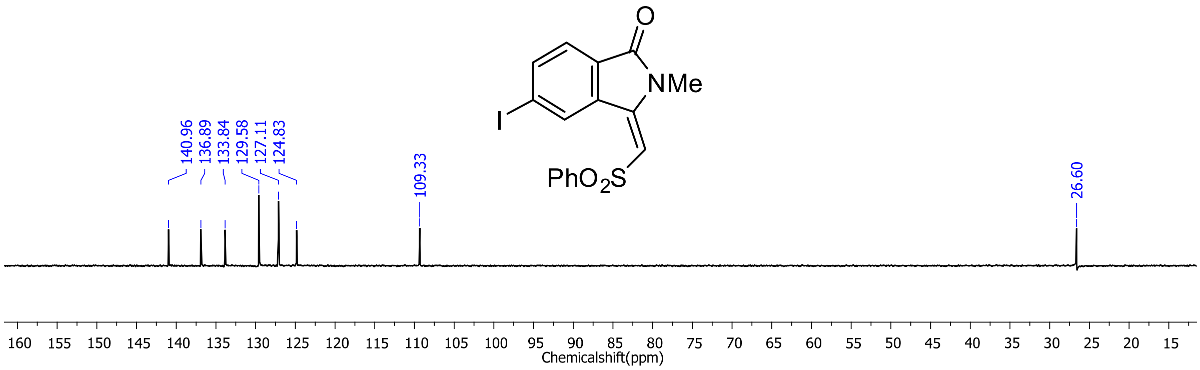

Spectral Data of Compound **3l.**

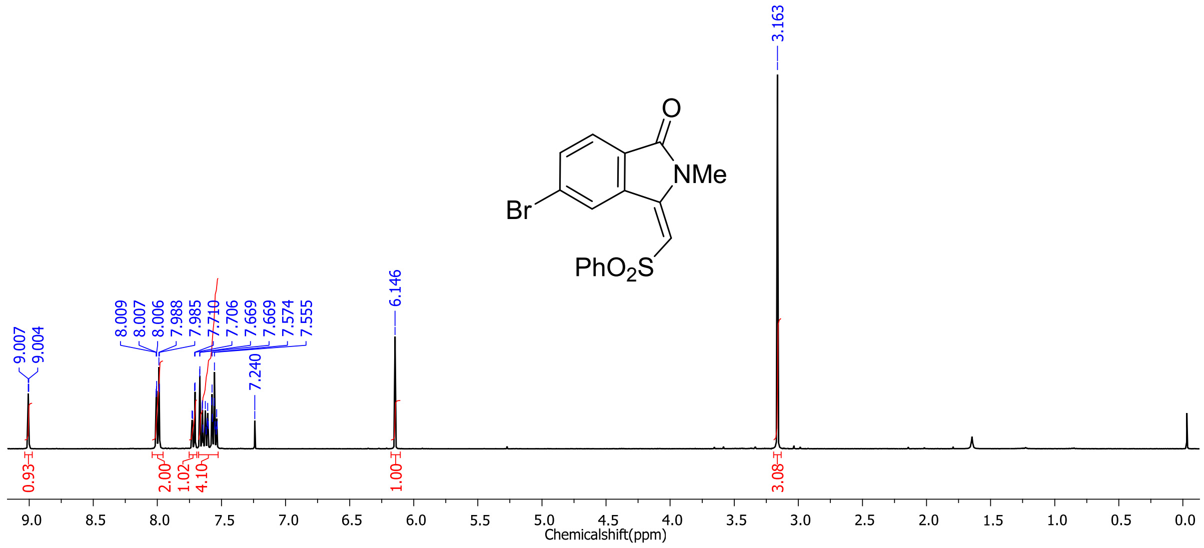

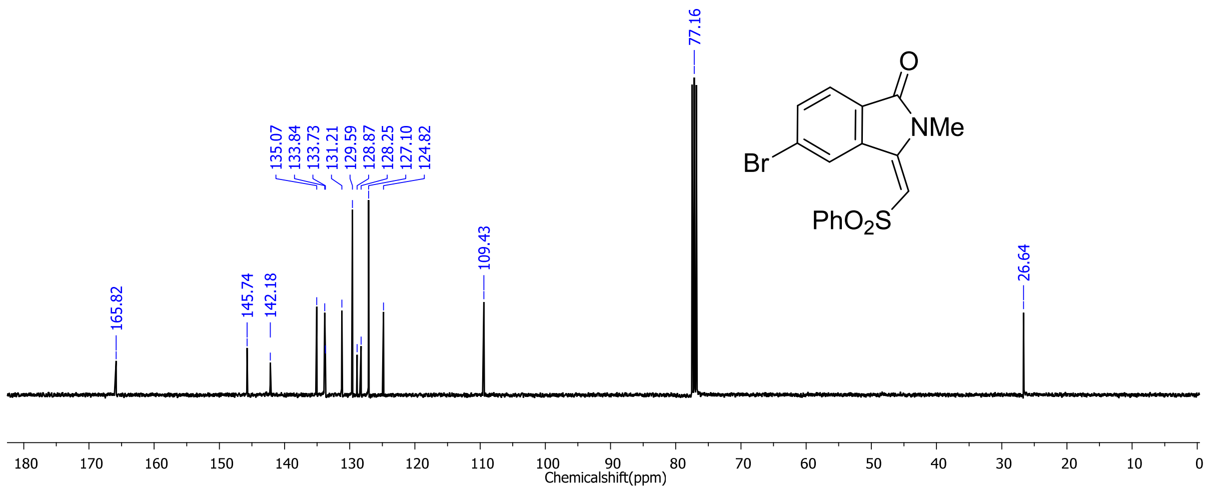

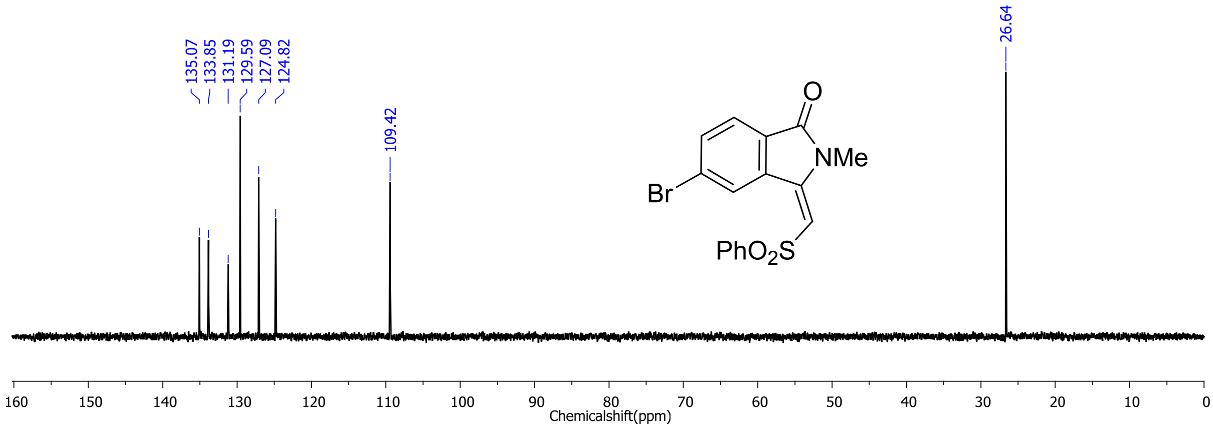

Spectral Data of Compound **3m.**

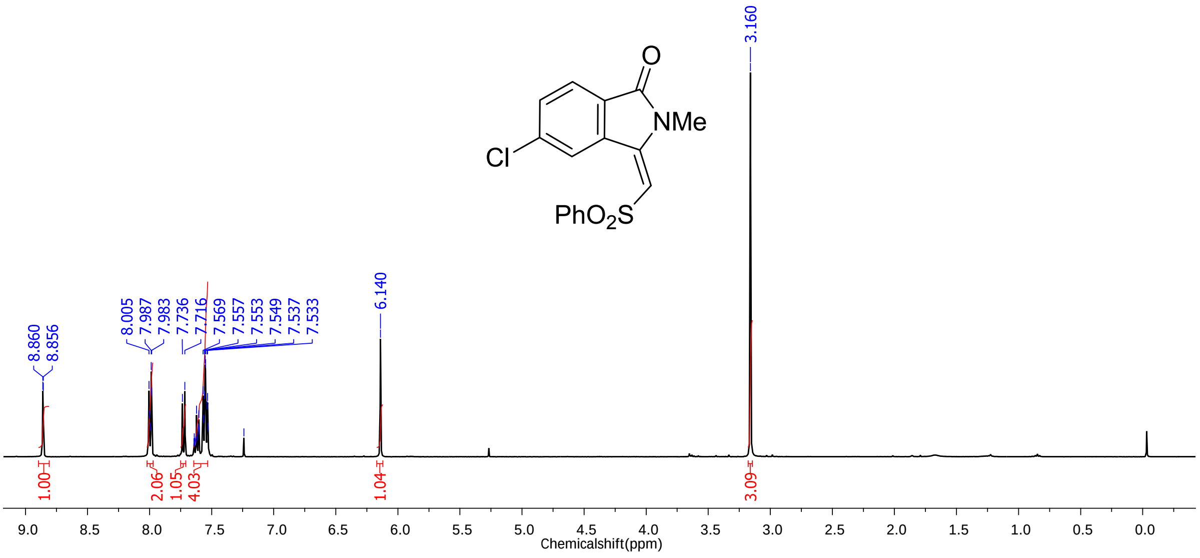

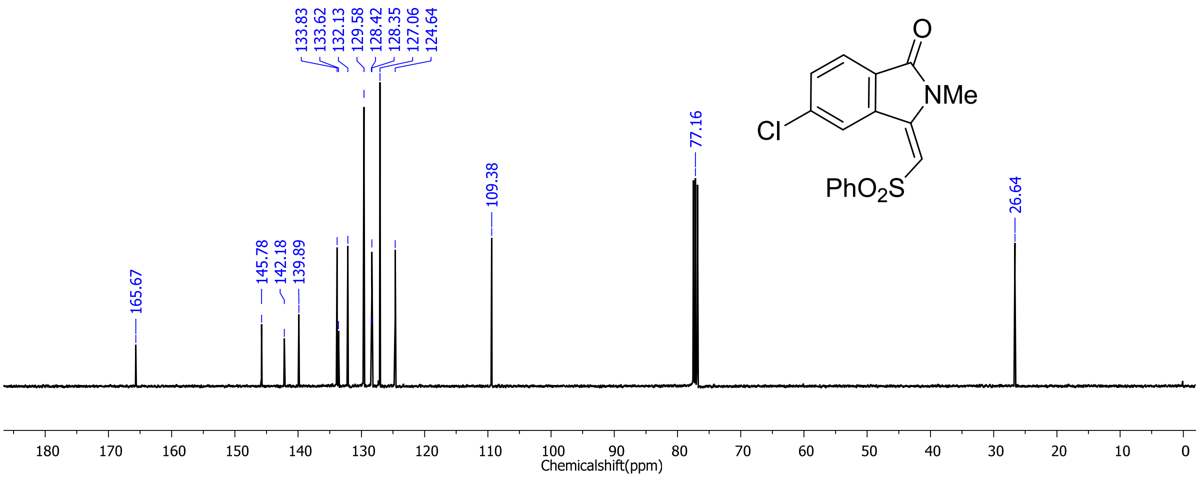

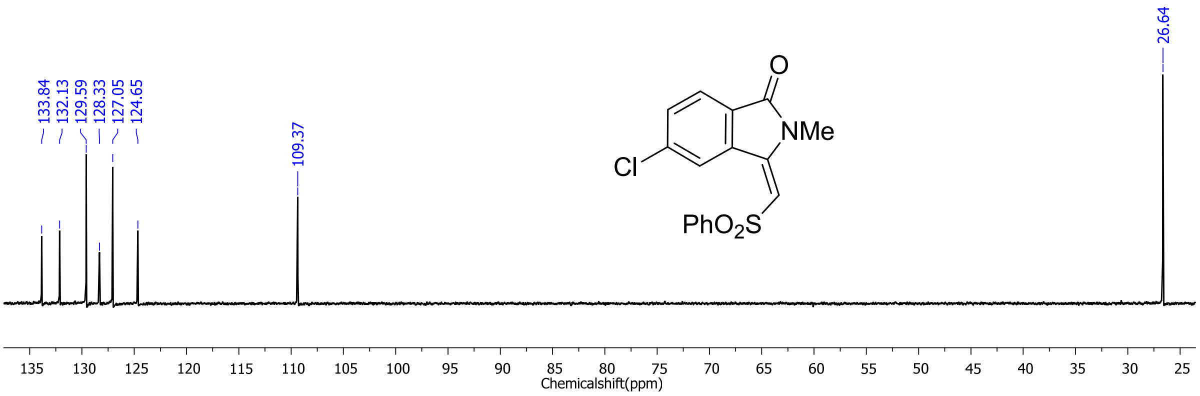

Spectral Data of Compound **3n.**

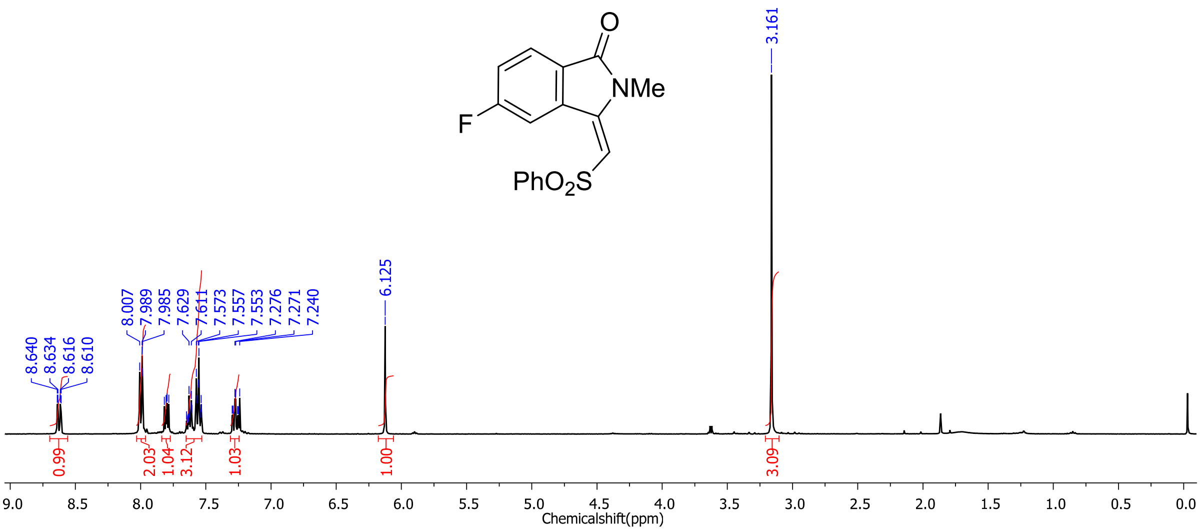

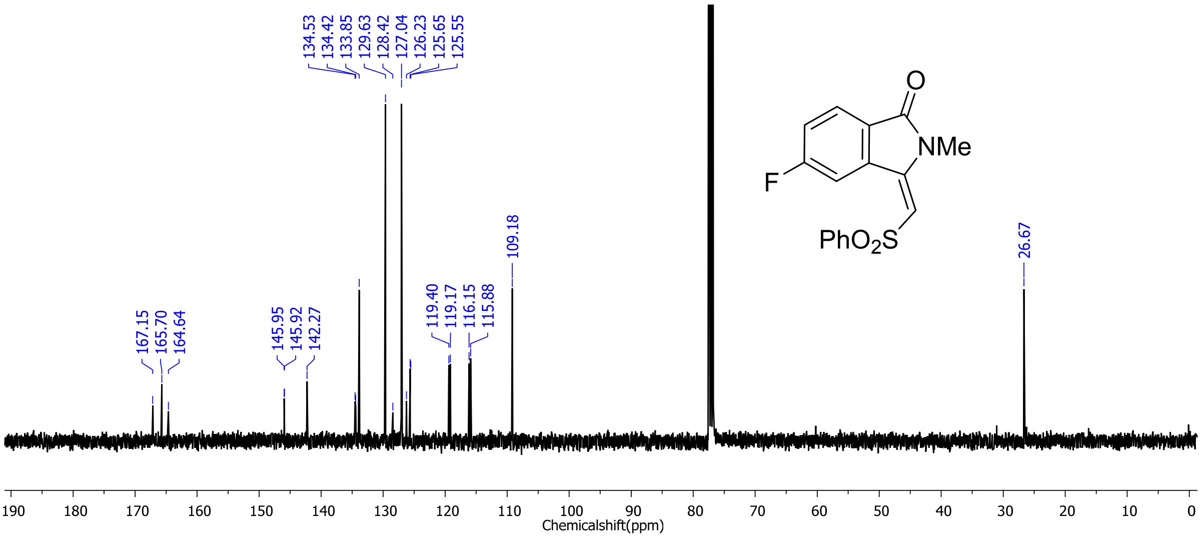

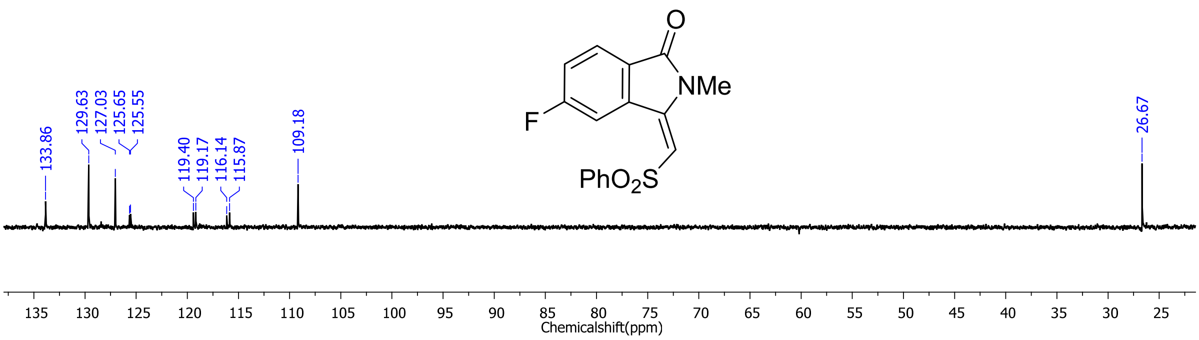

Spectral Data of Compound **3o.**

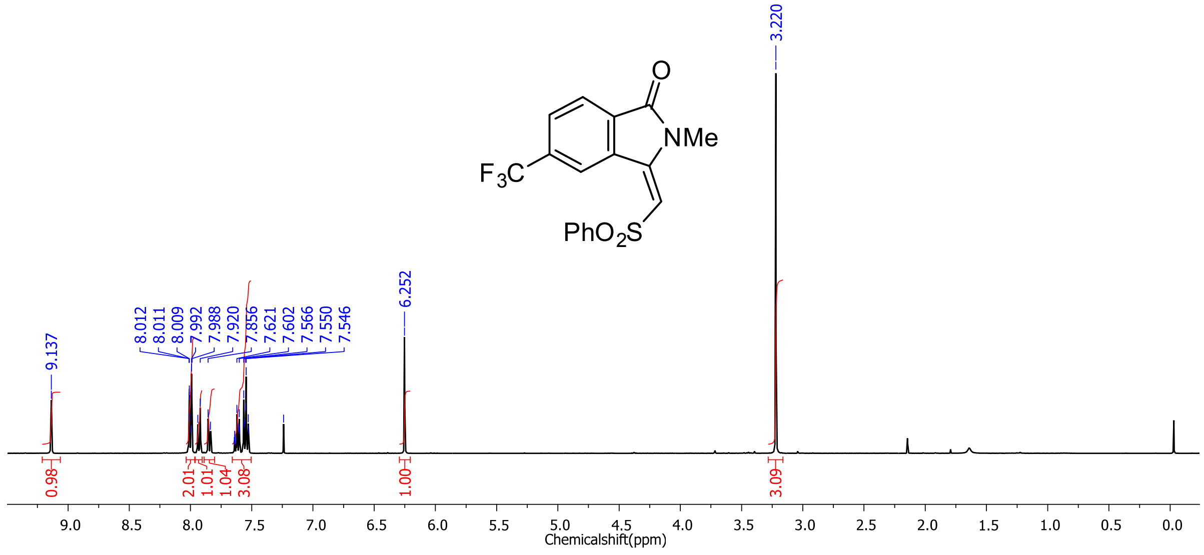

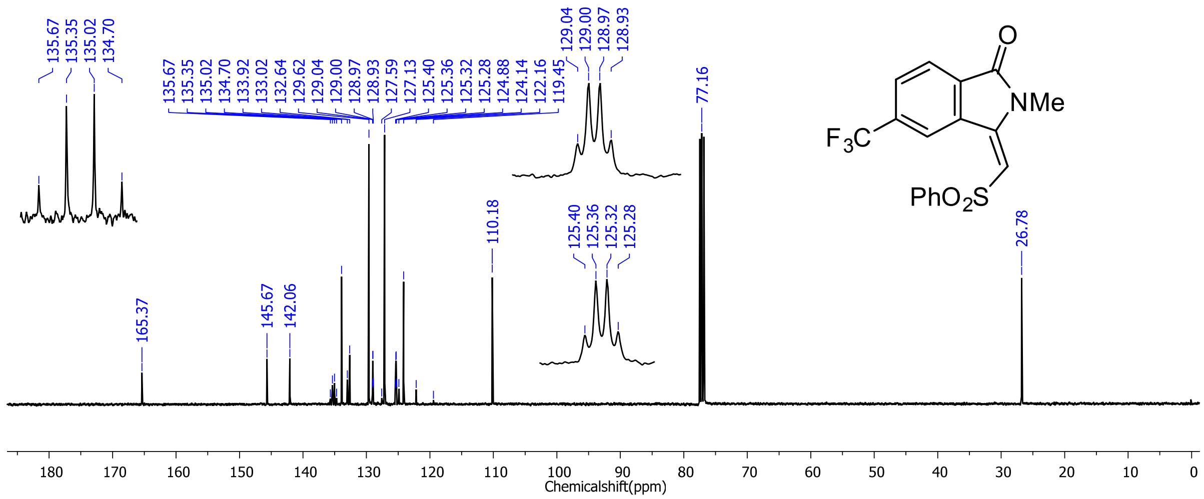

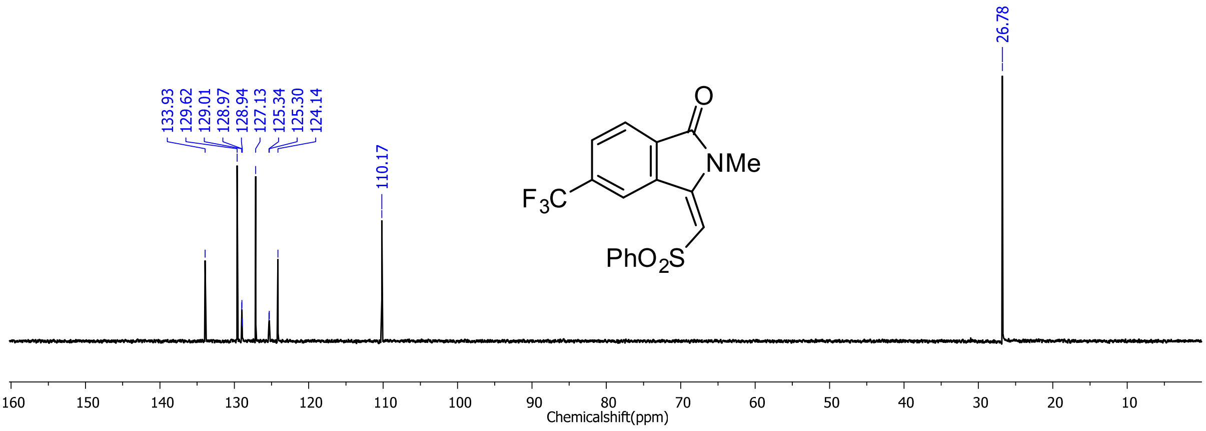

Spectral Data of Compound **3p.**

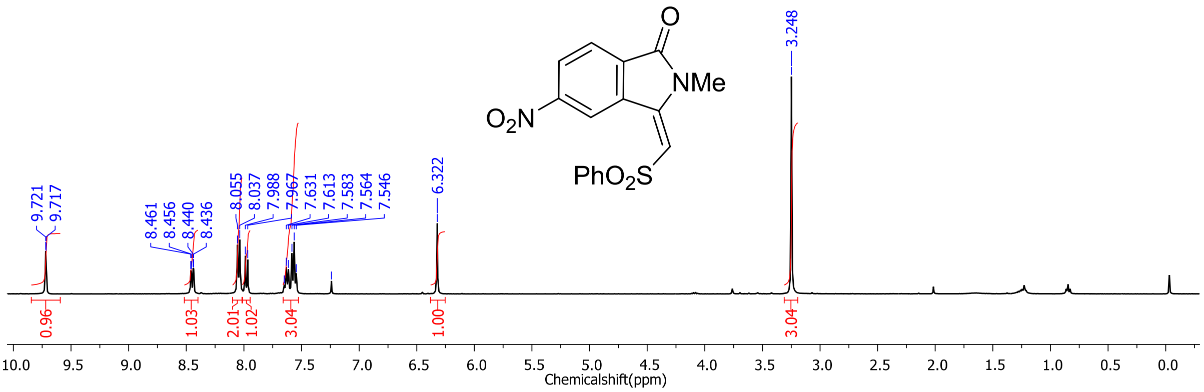

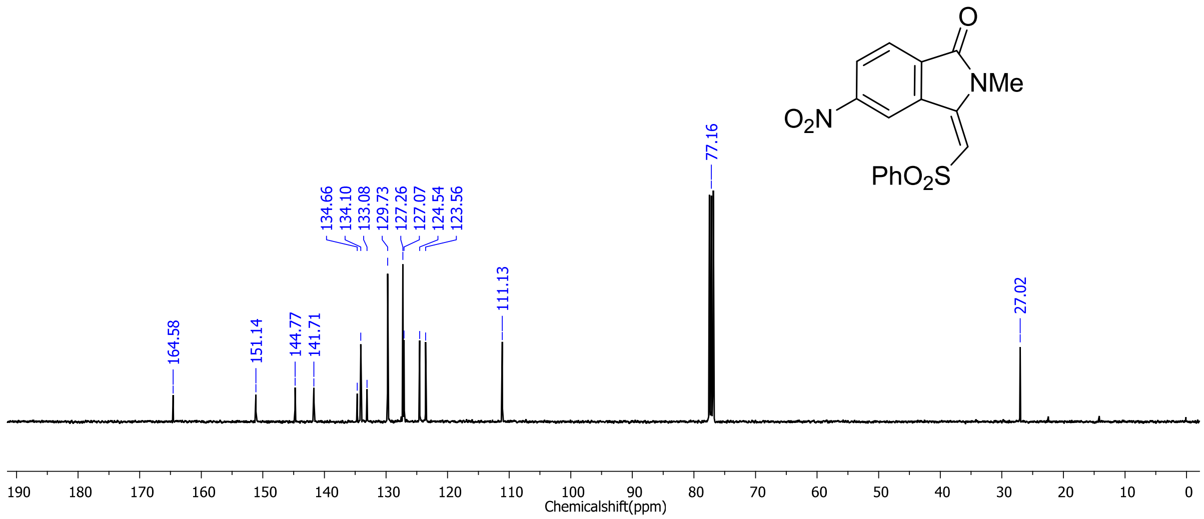

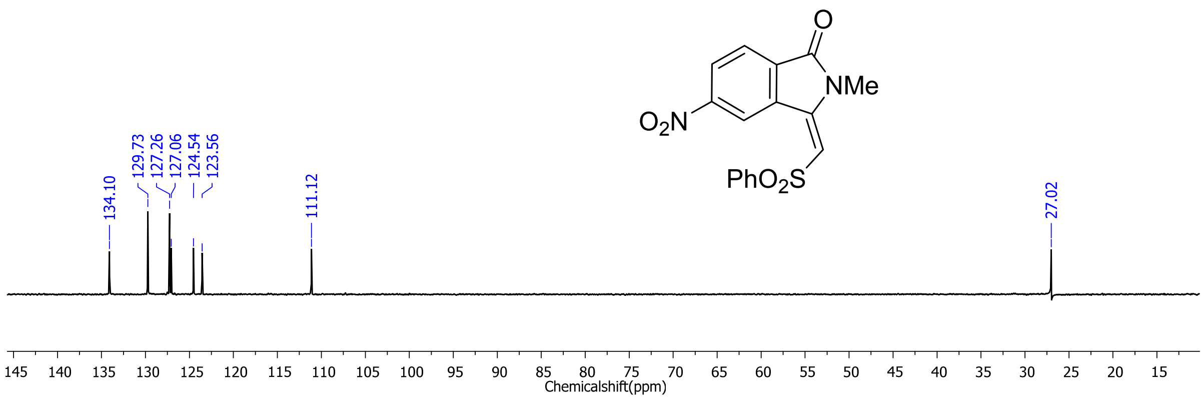

Spectral Data of Compound **3q.**

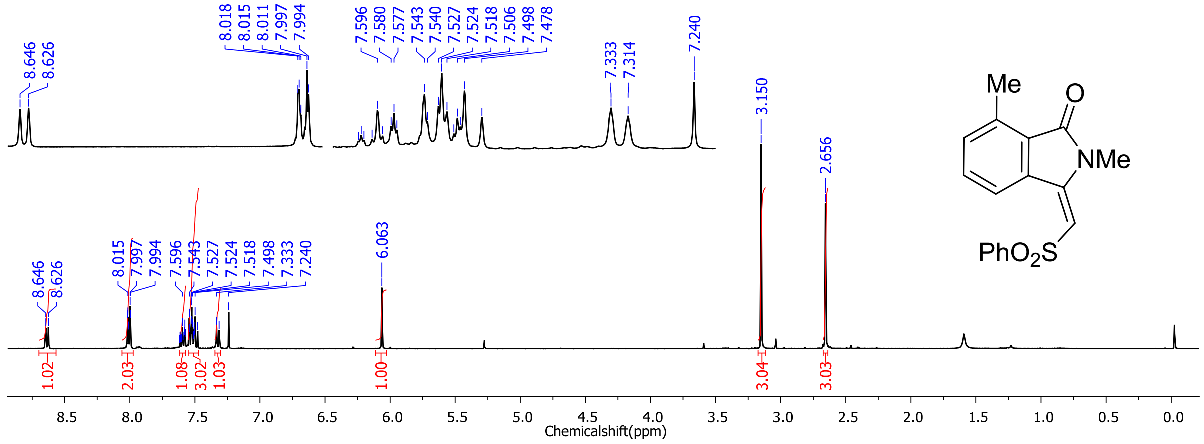

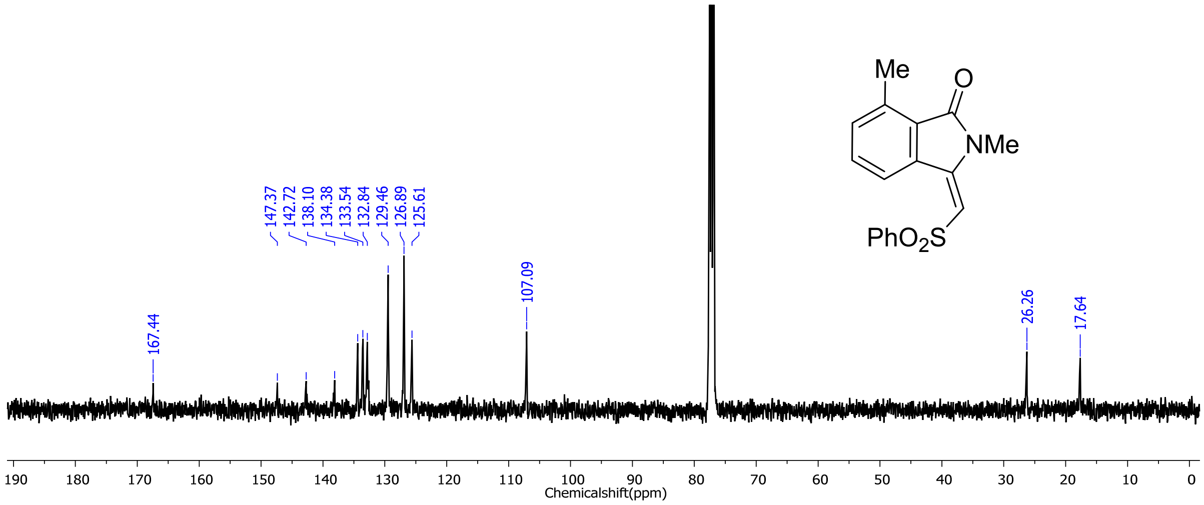

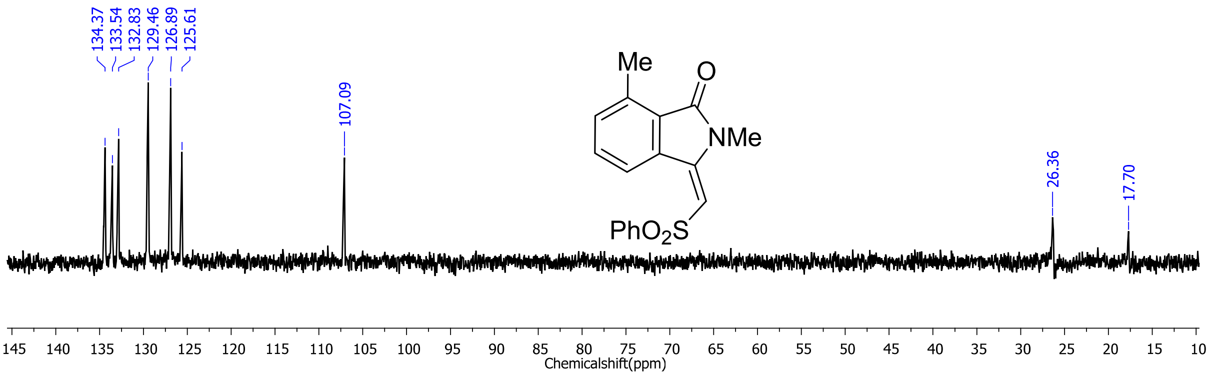

Spectral Data of Compound **3r.**

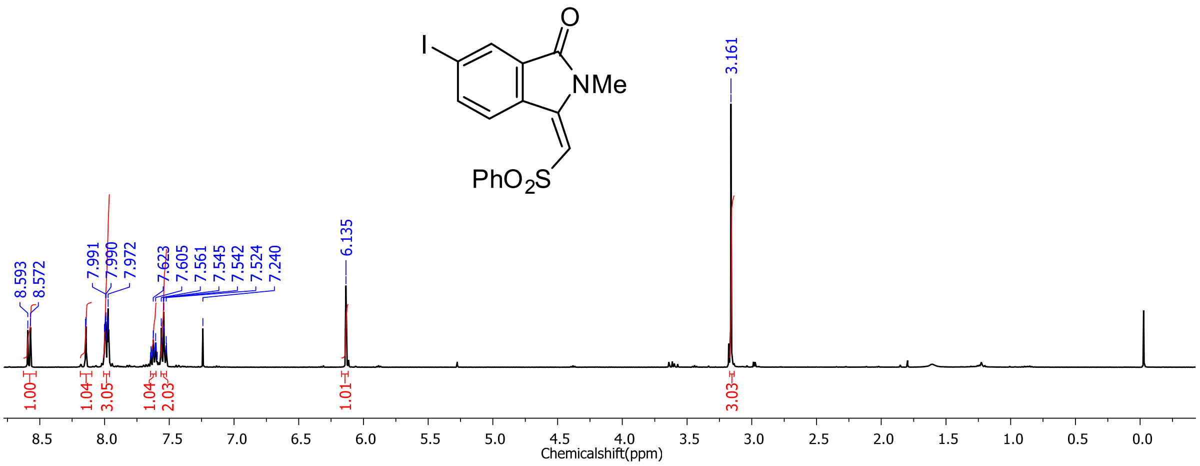

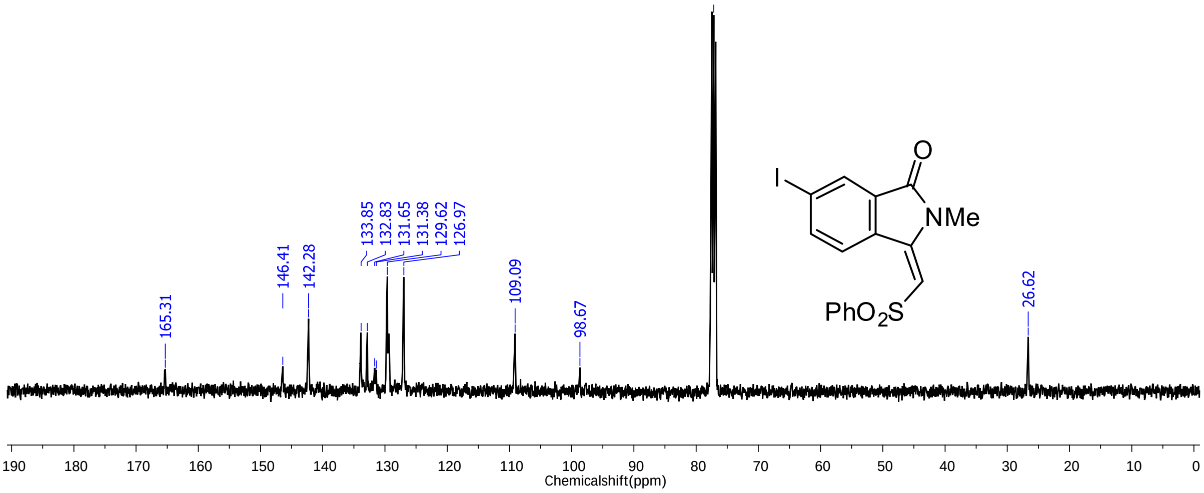

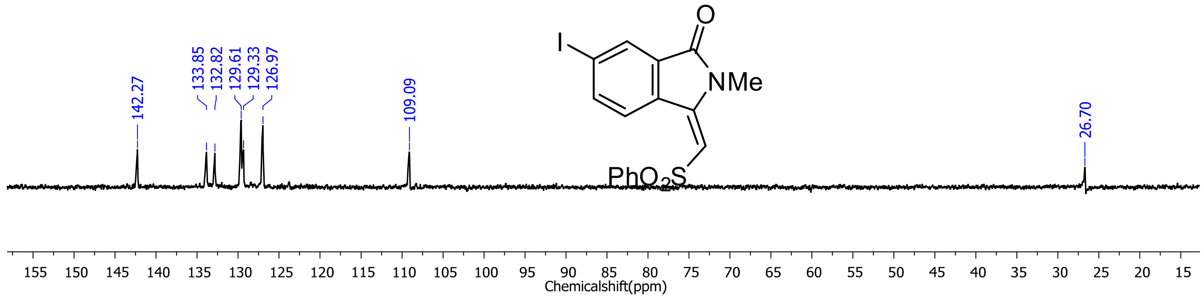

Spectral Data of Compound **3s.**

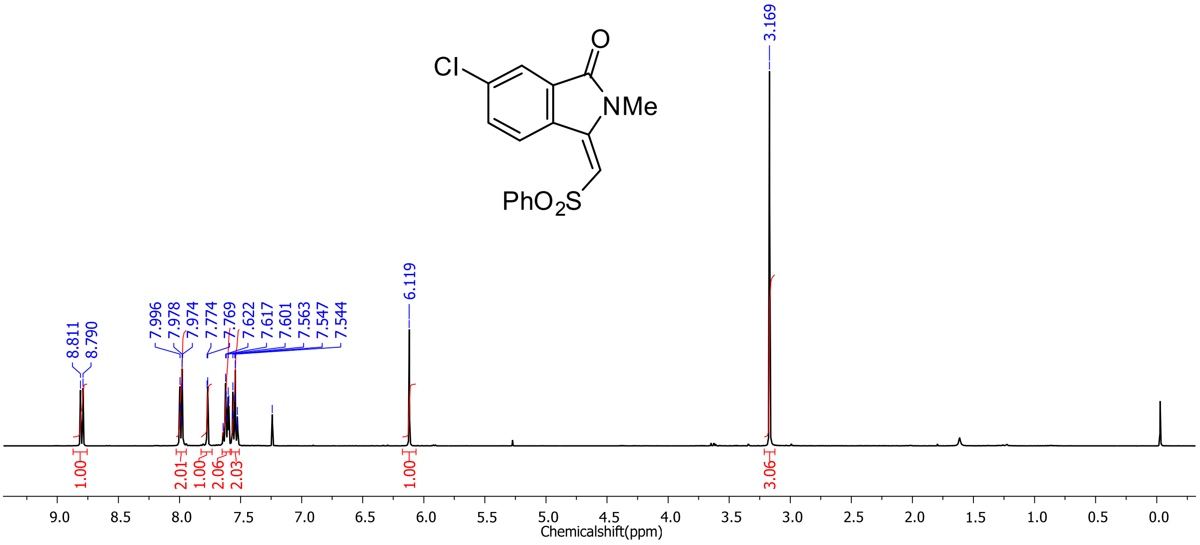

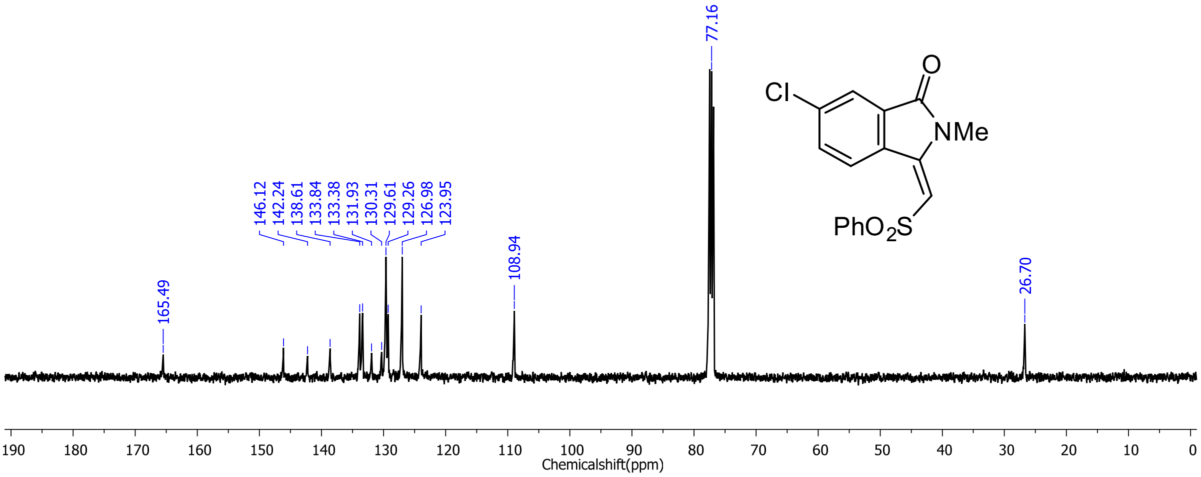

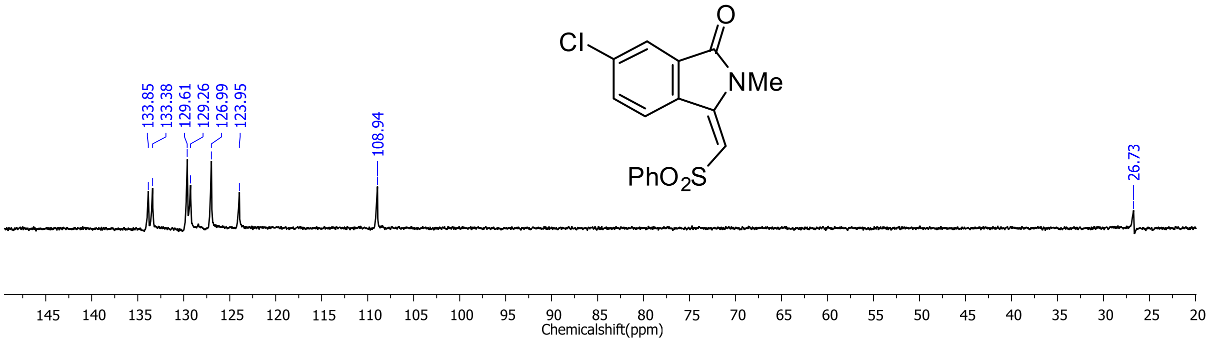

Spectral Data of Compound **3x.**

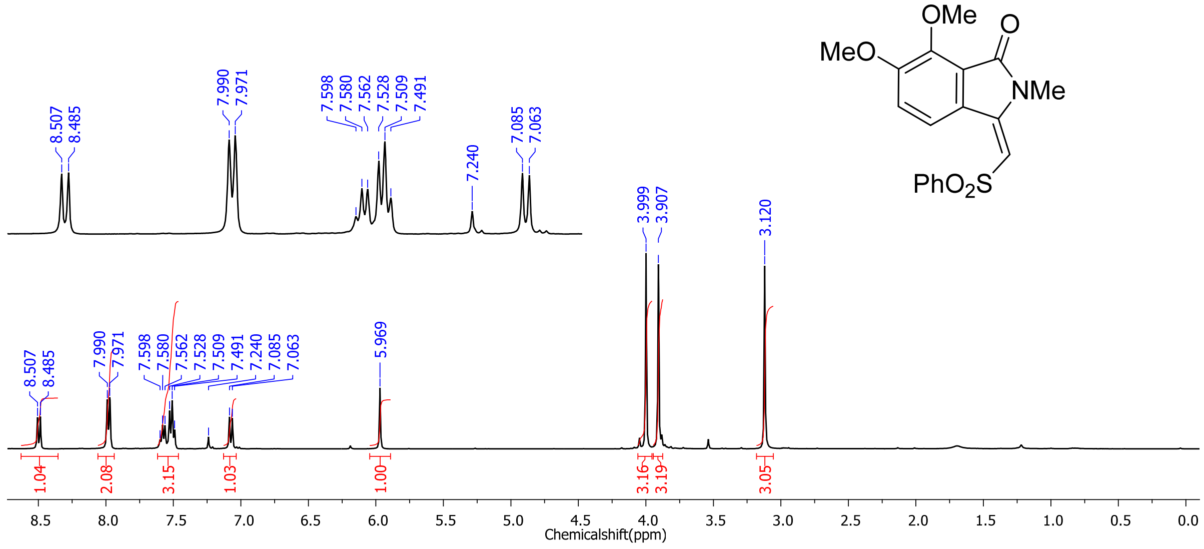

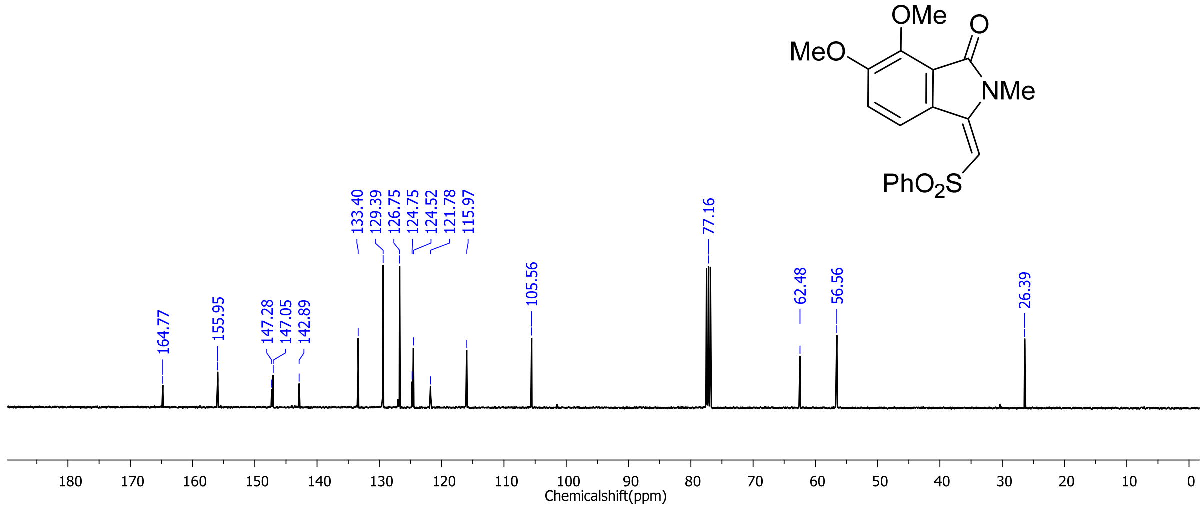

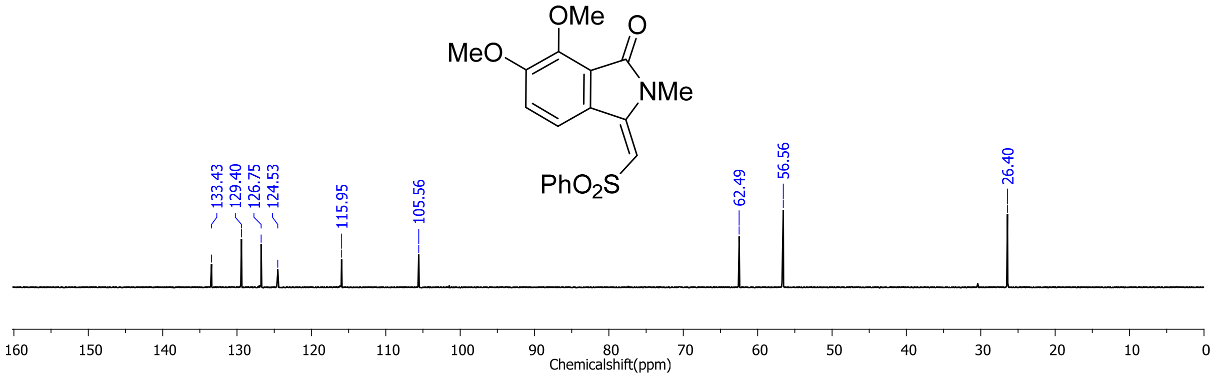

Spectral Data of Compound **3y.**

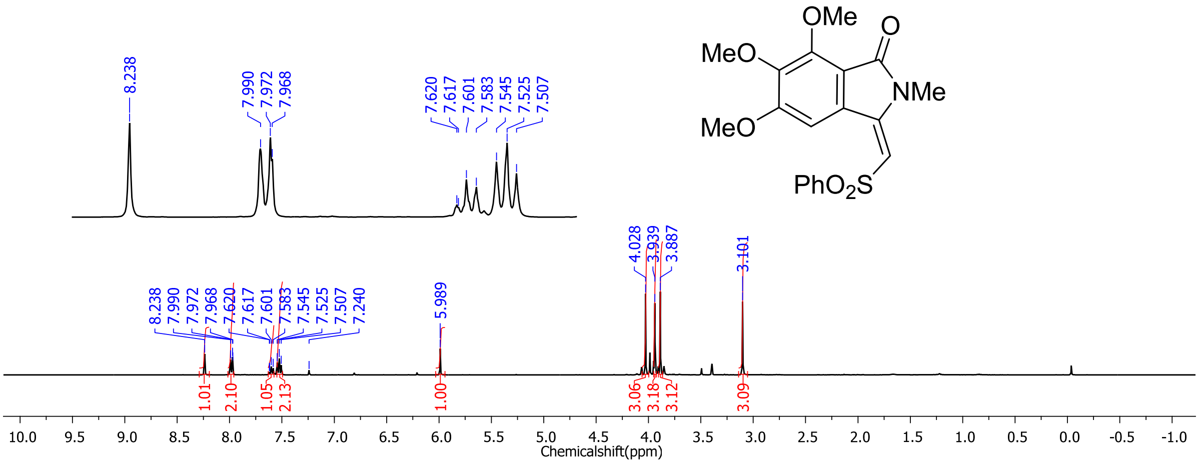

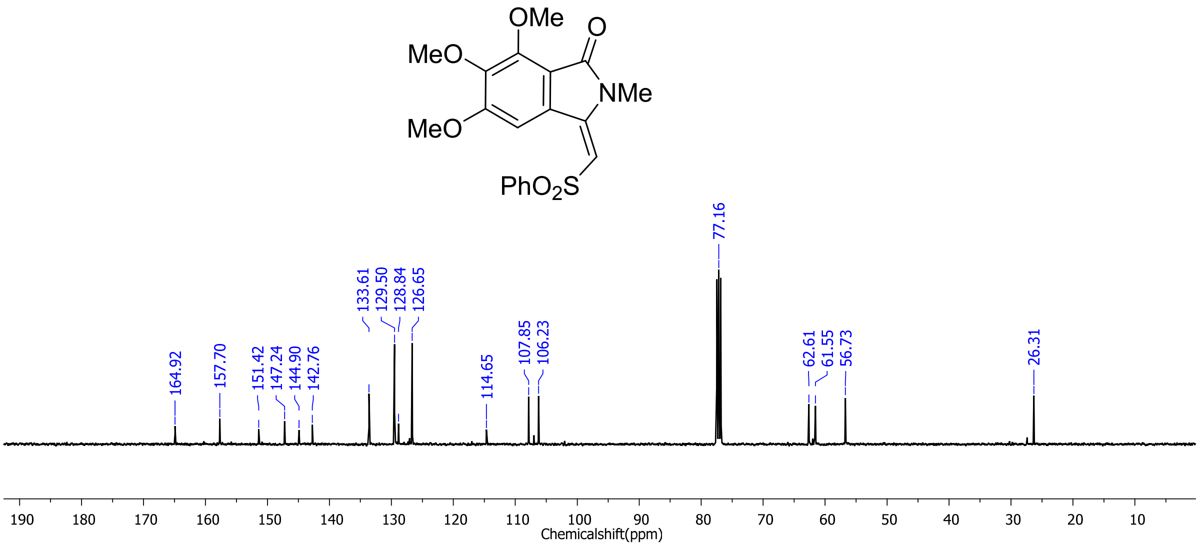

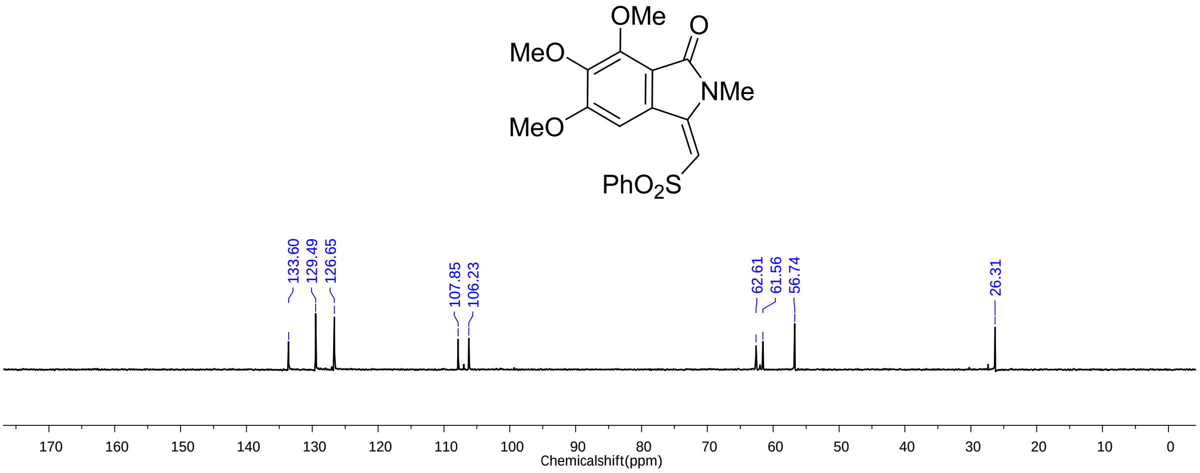

Spectral Data of Compound **3z.**

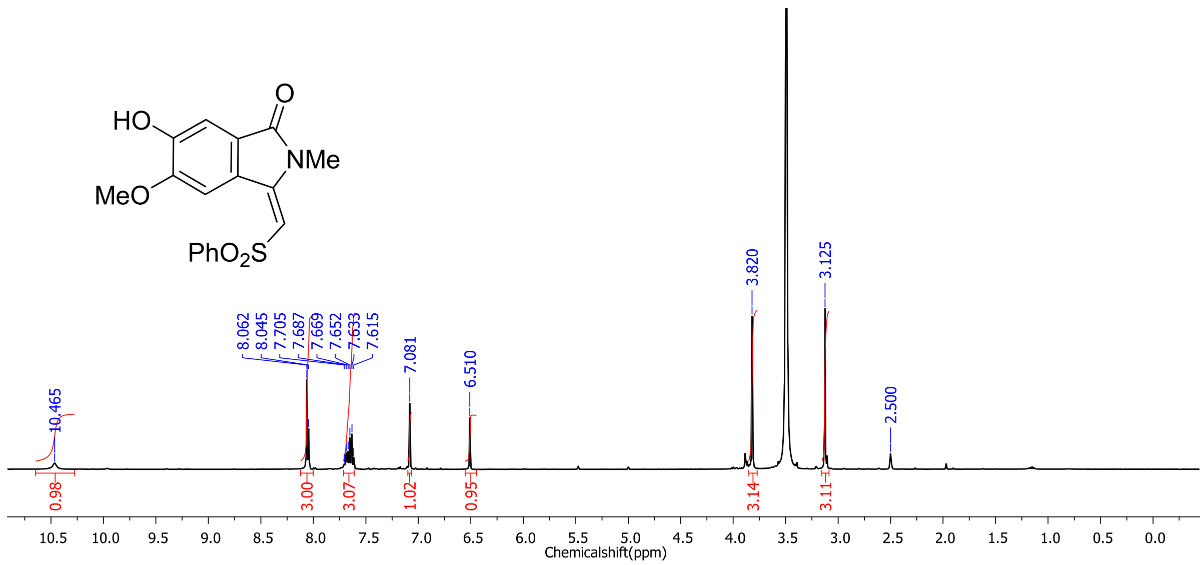

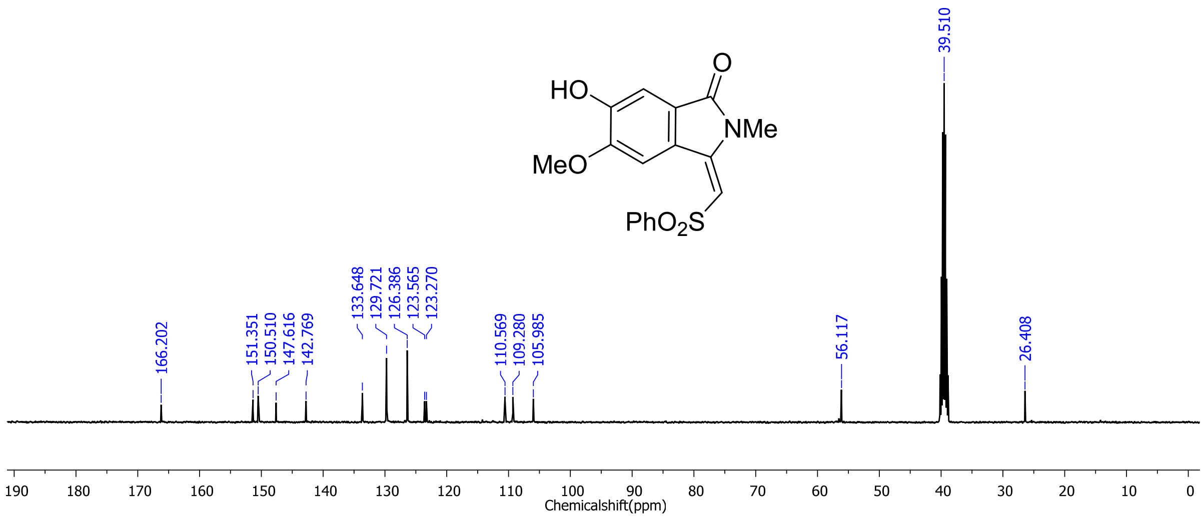

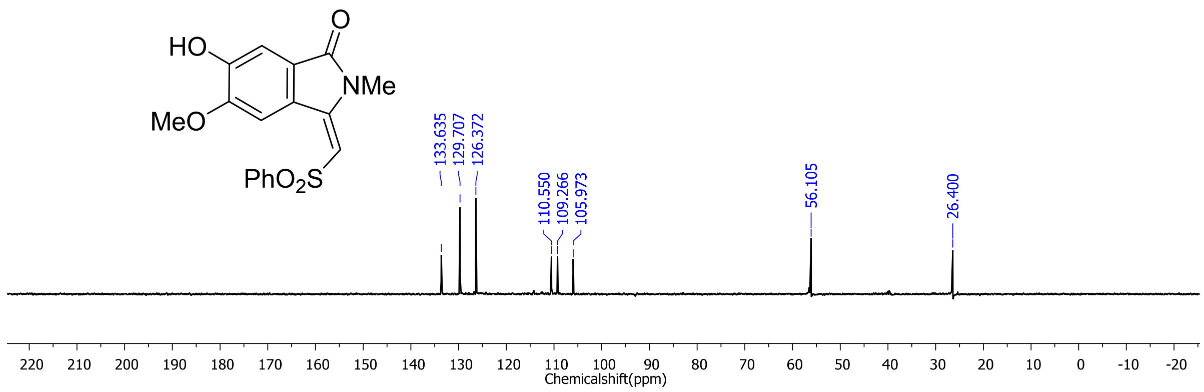

Spectral Data of Compound **3wa.**

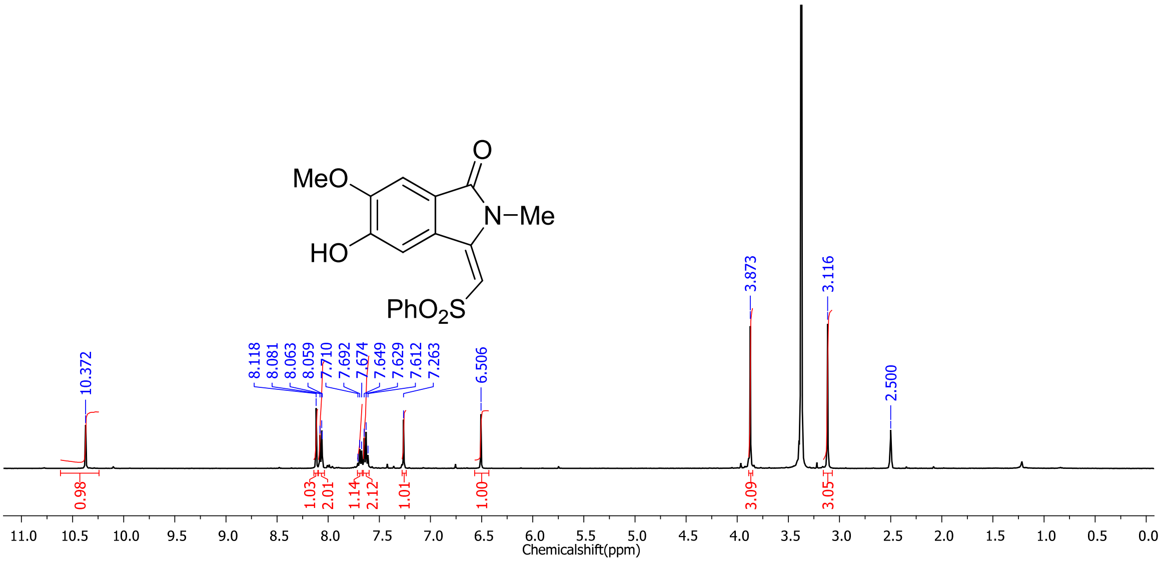

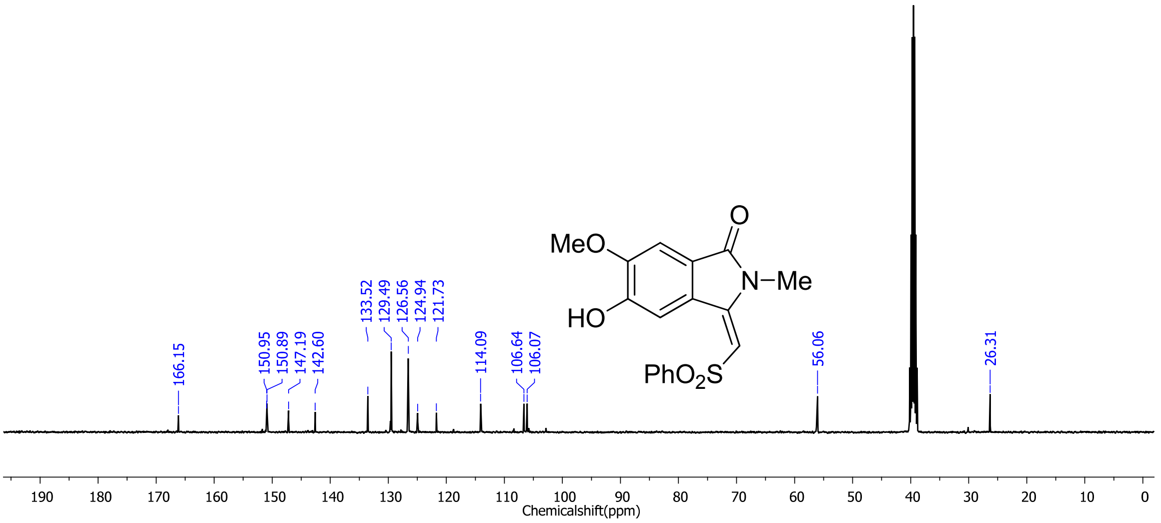

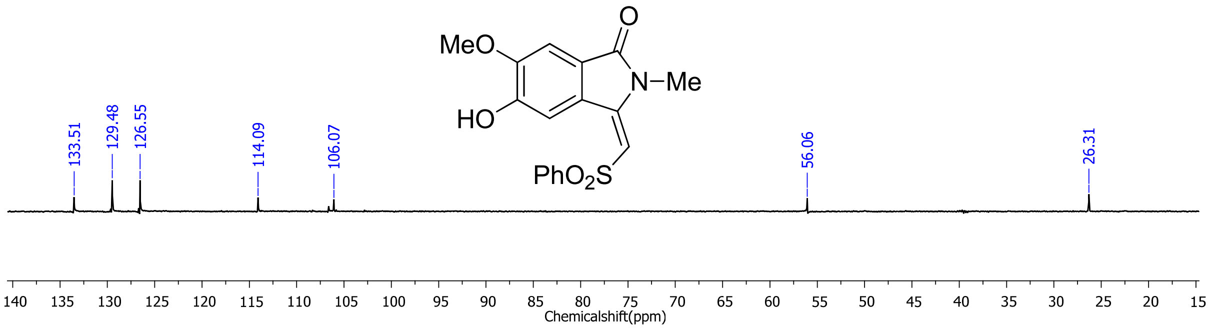

Spectral Data of Compound **3xa.**

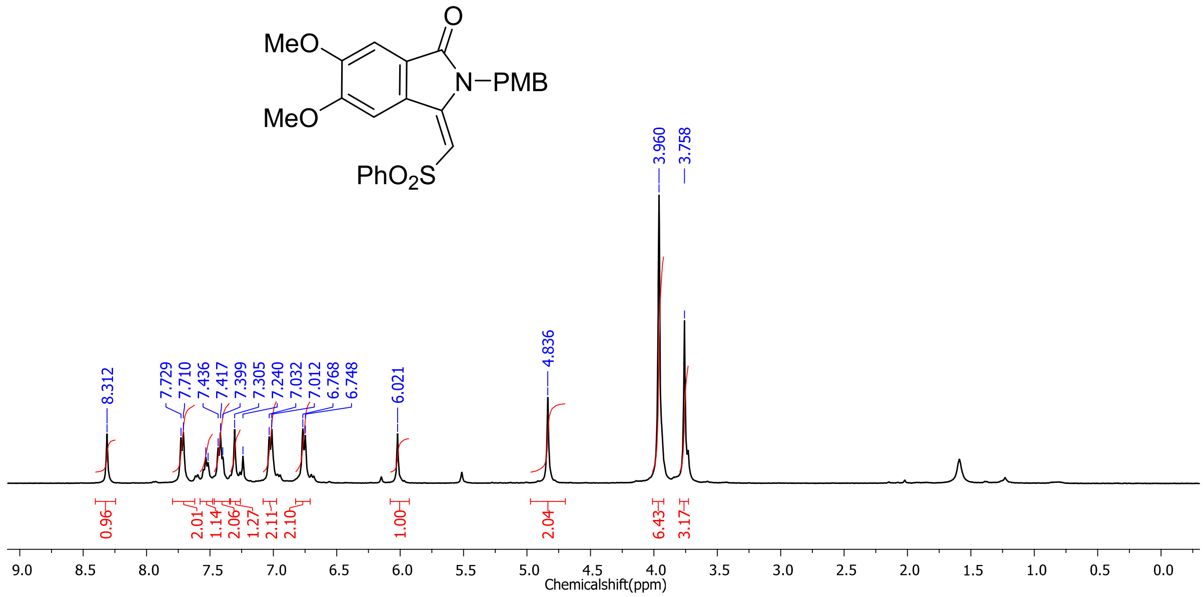

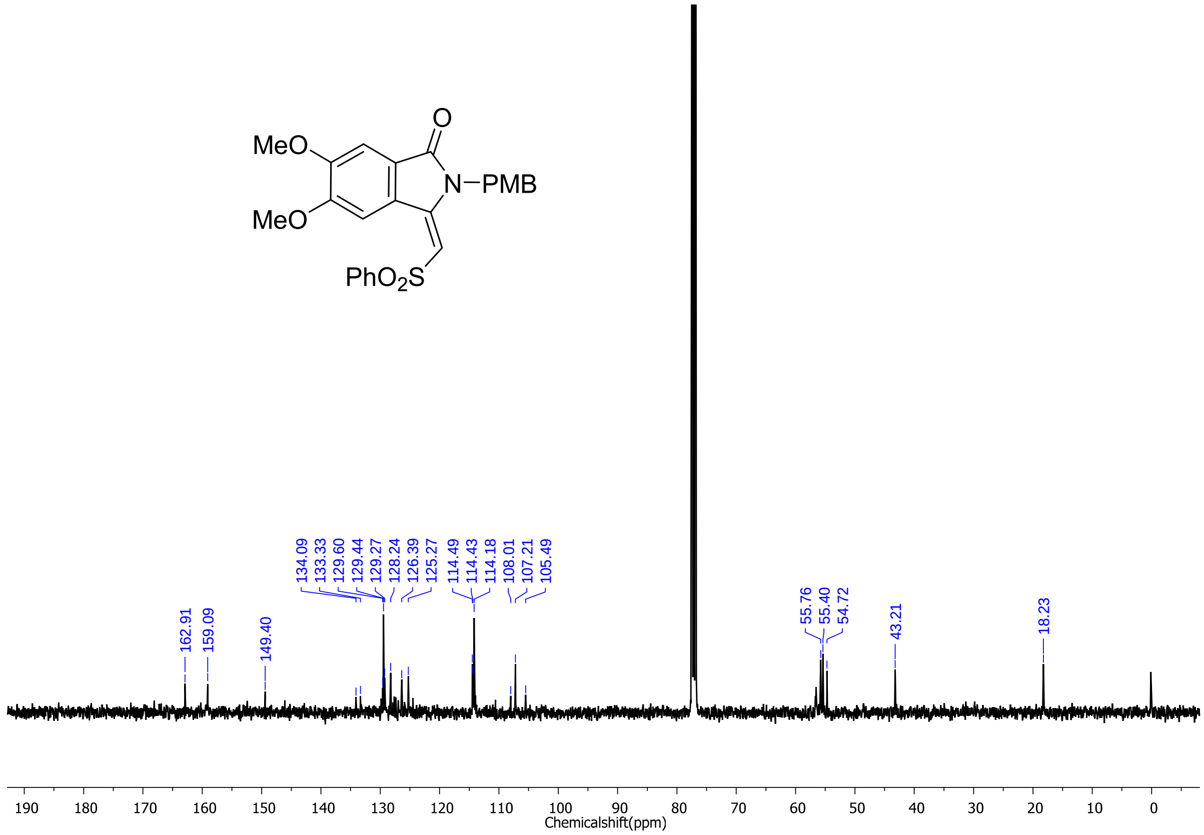

Spectral Data of Compound **3ya.**

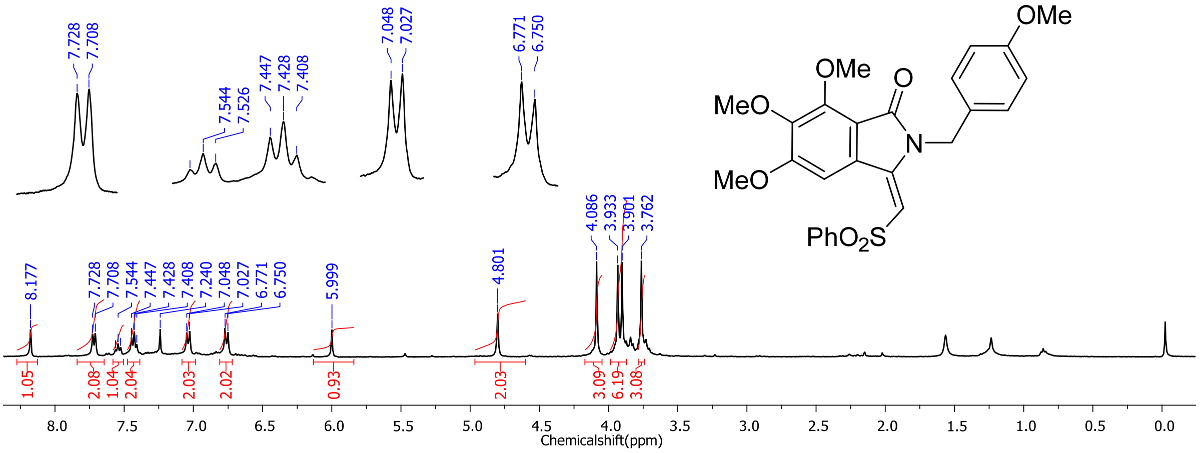

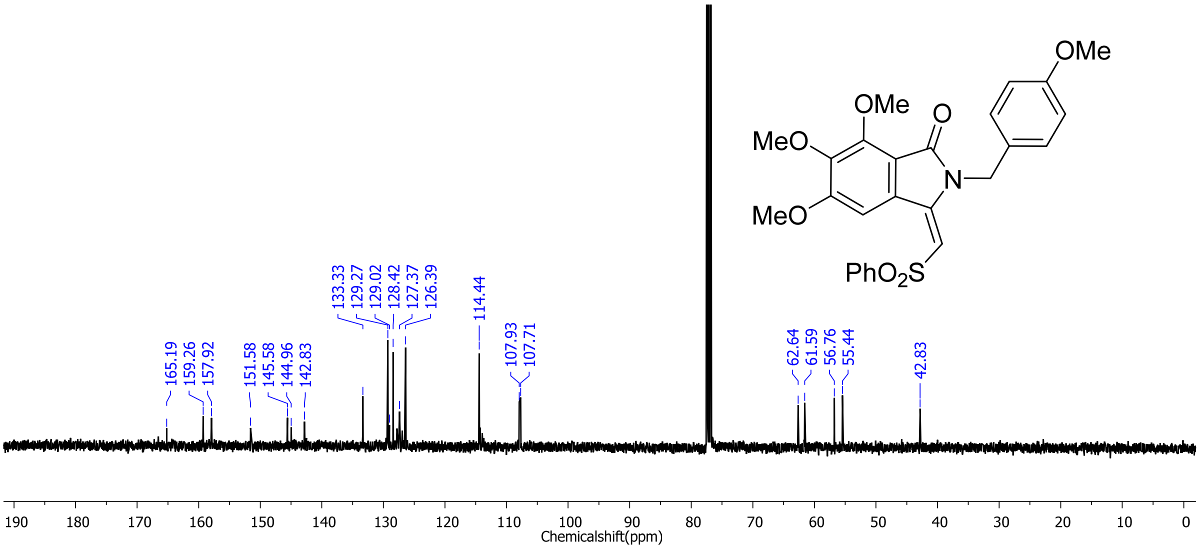

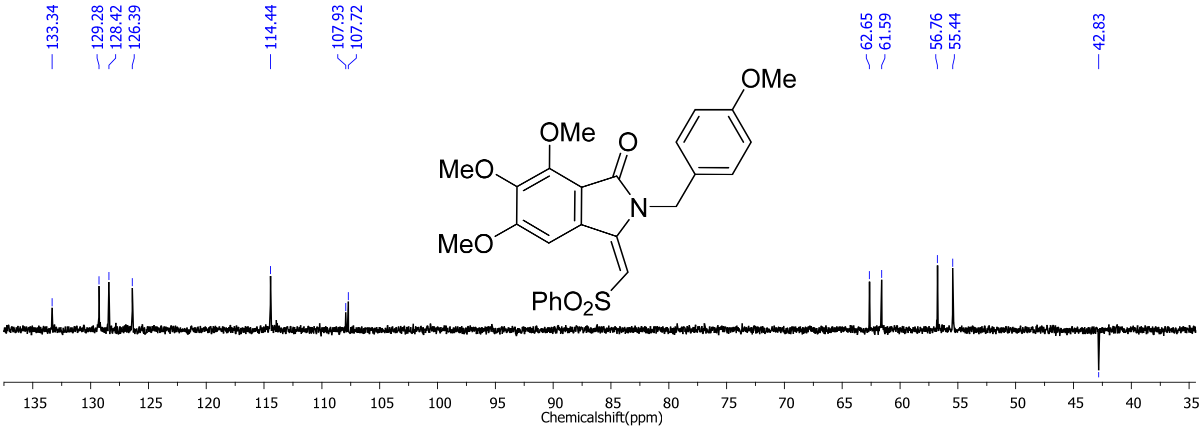

Spectral Data of Compound **3t.**

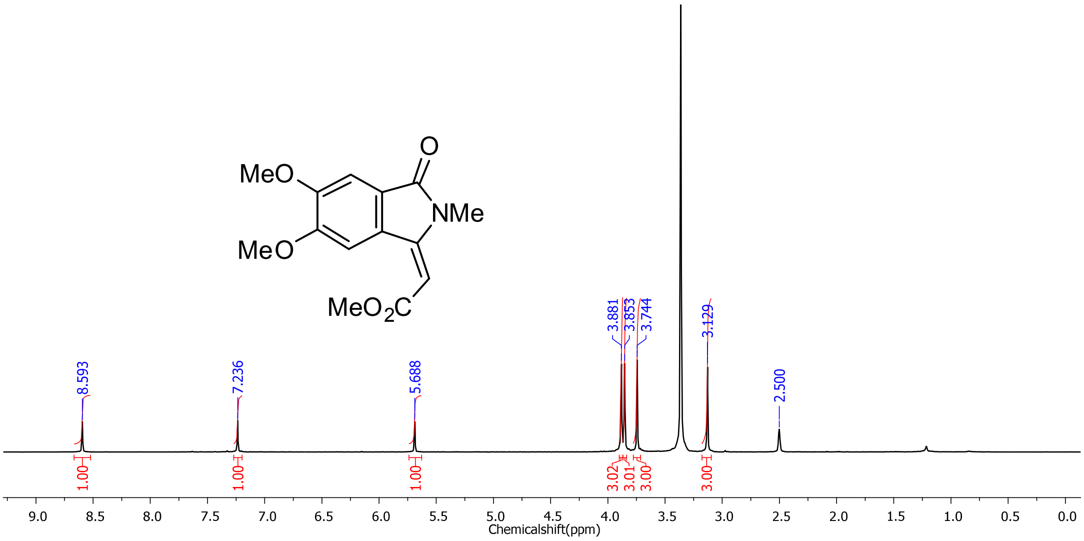

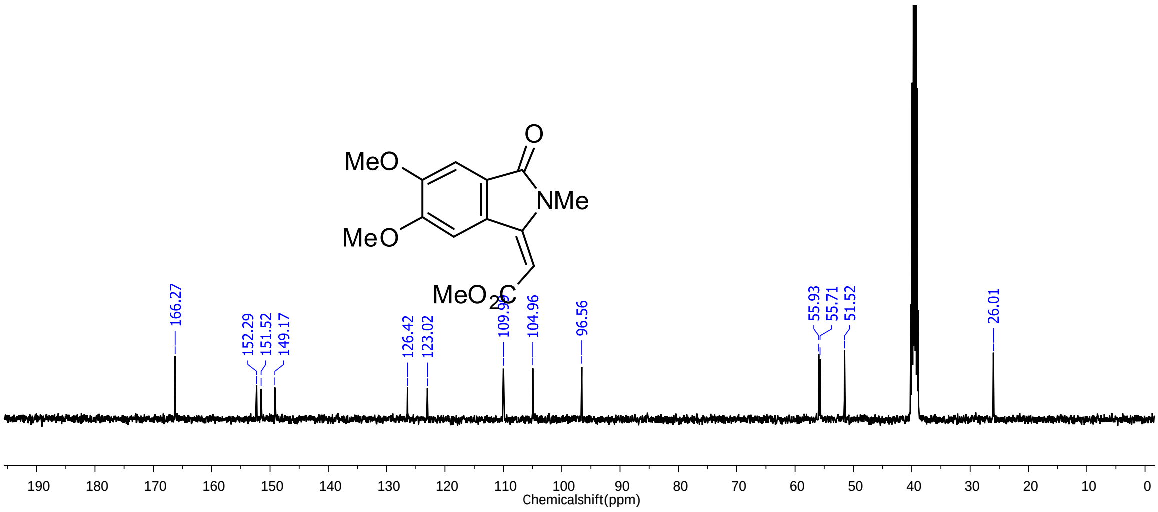

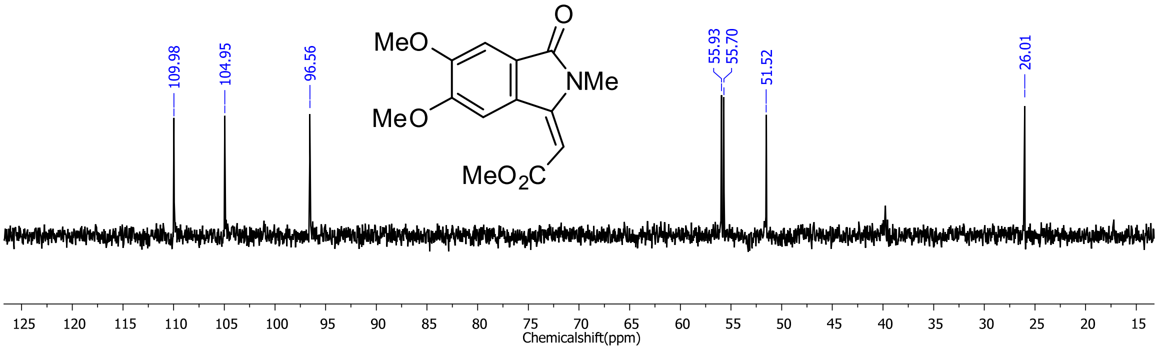

Spectral Data of Compound **3u.**

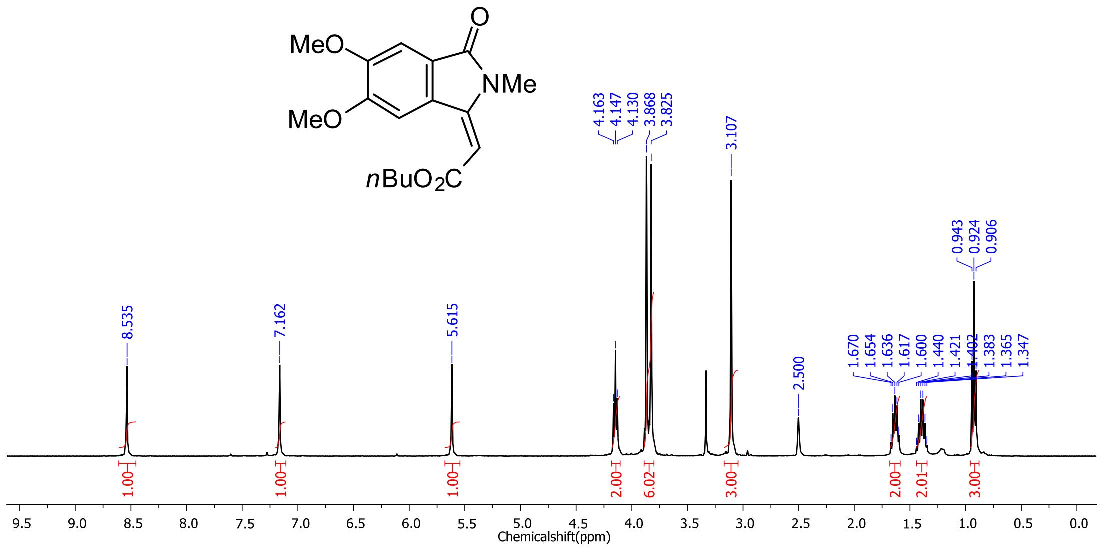

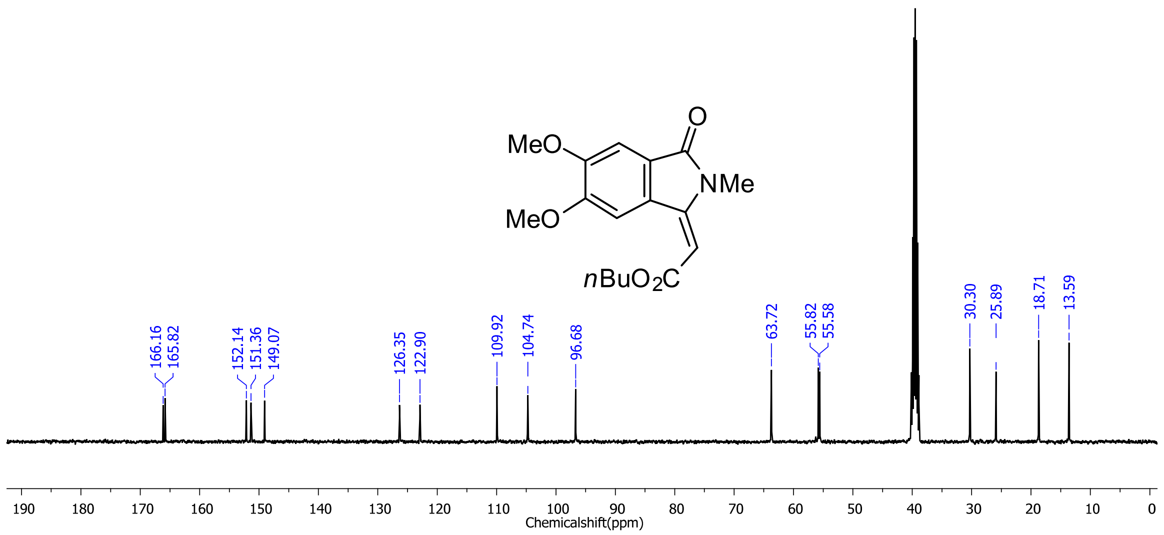

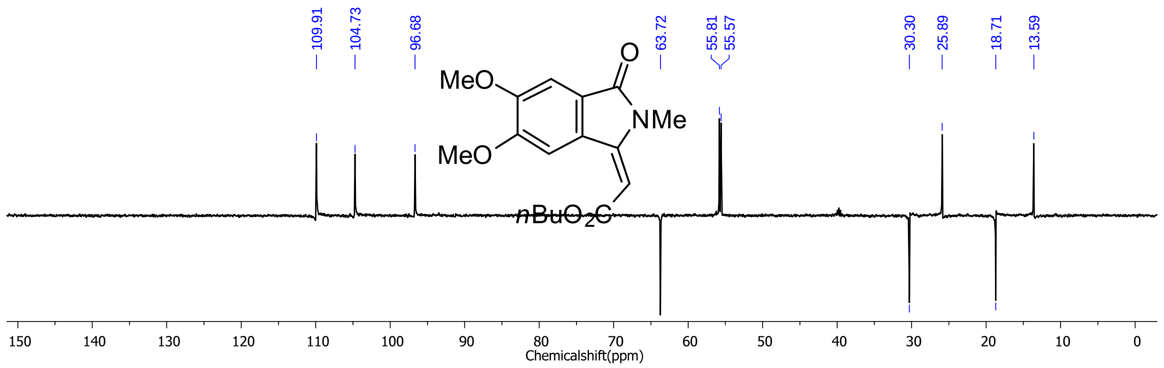

Spectral Data of Compound **3v.**

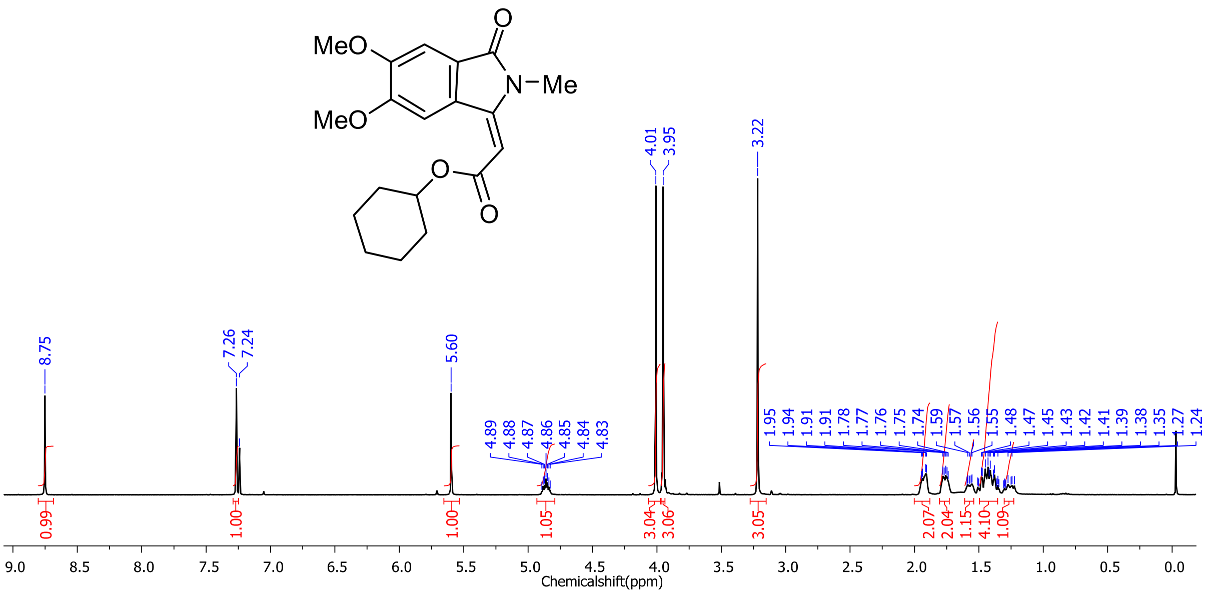

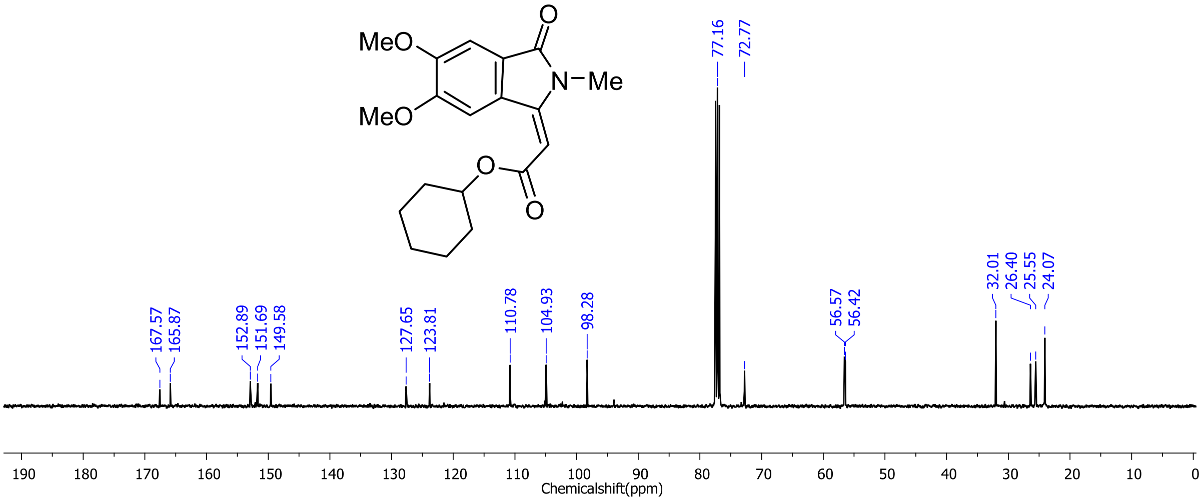

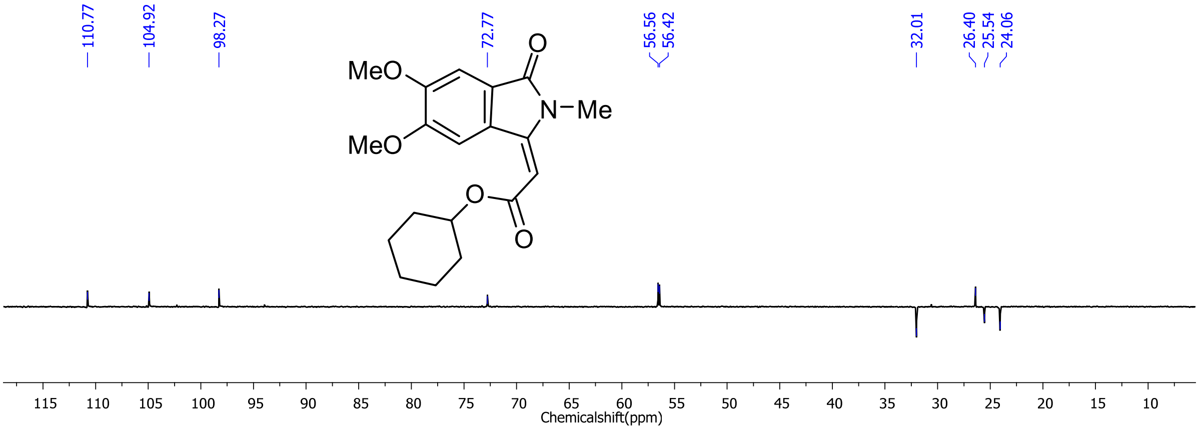

Spectral Data of Compound **3w.**

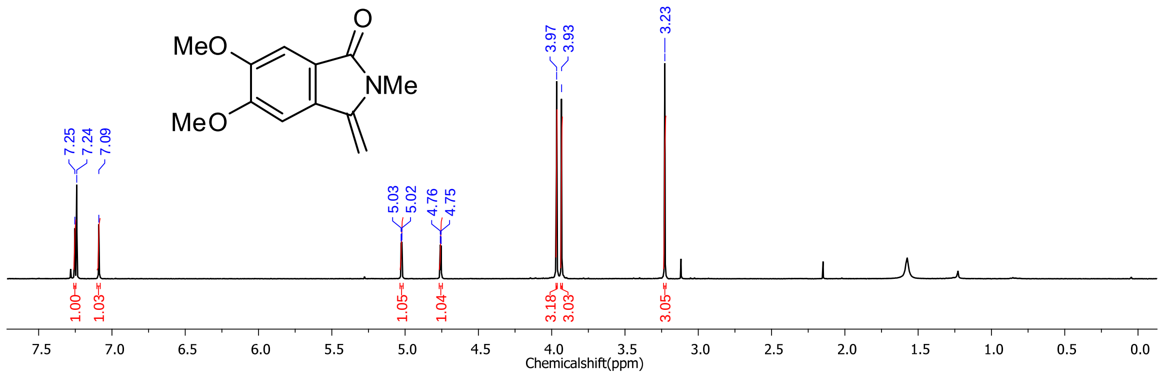

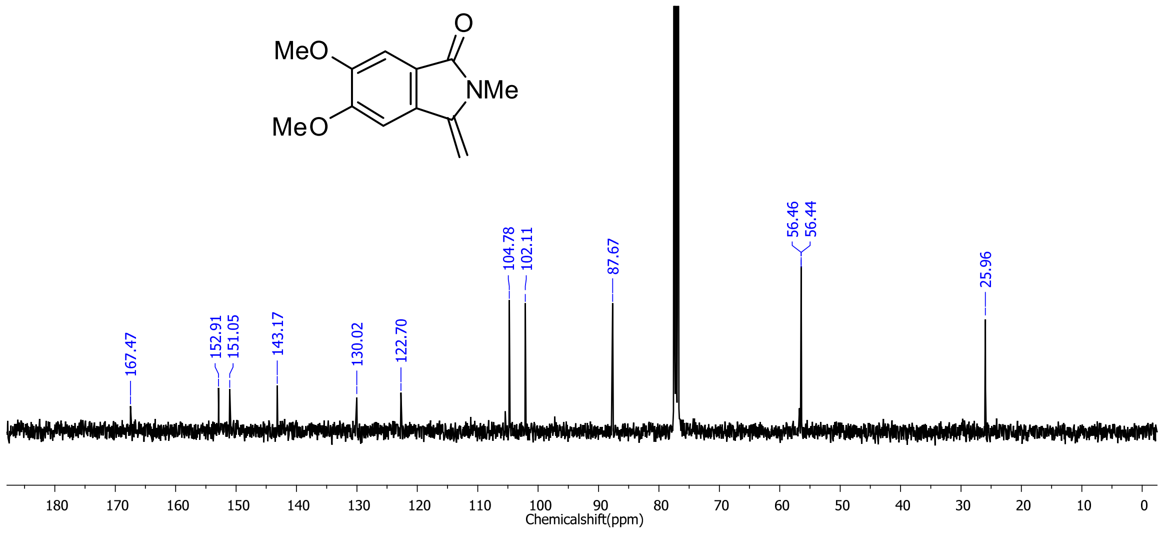

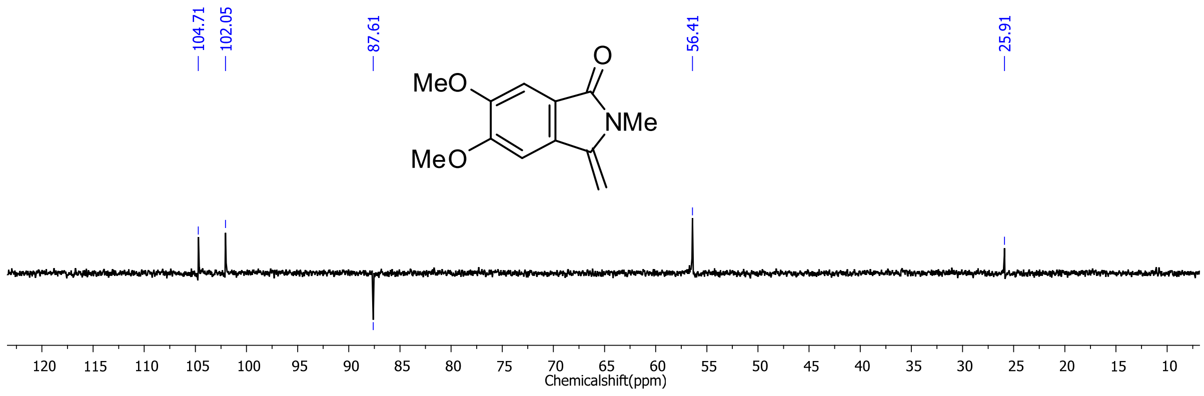

Spectral Data of Compoun **9a**.

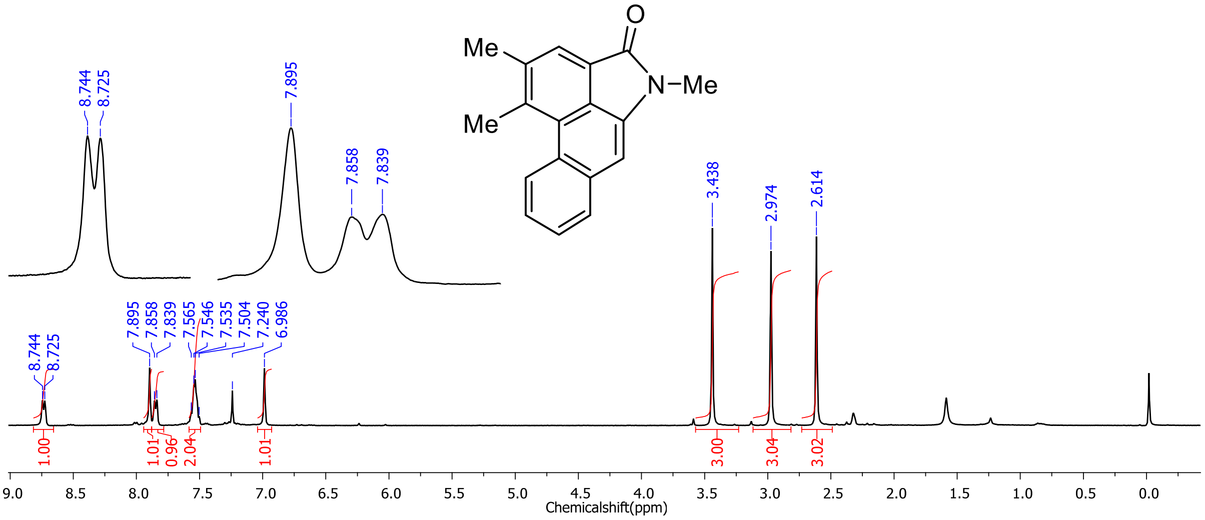

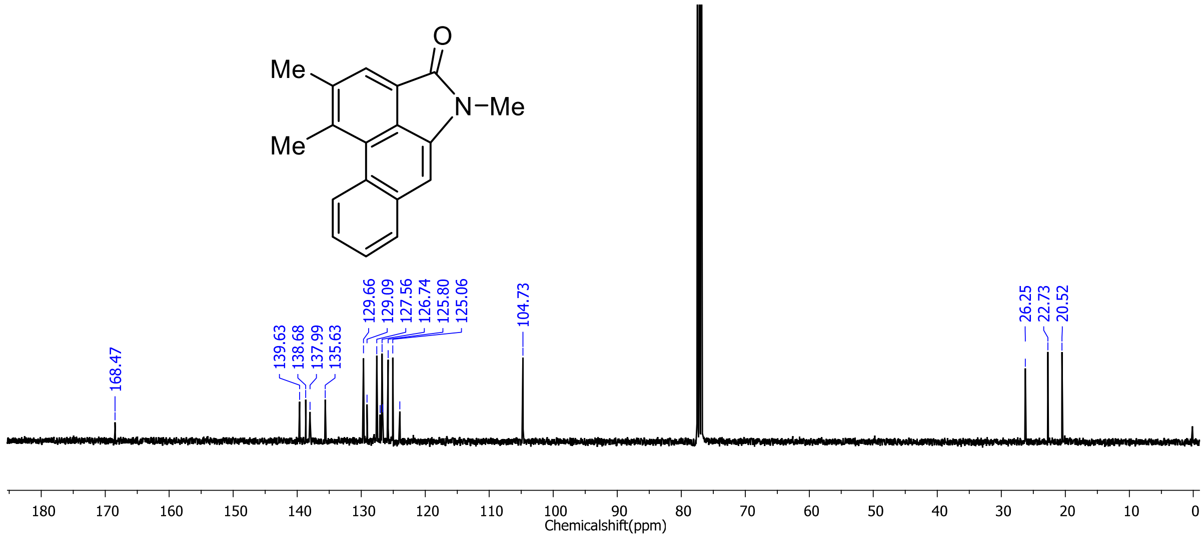

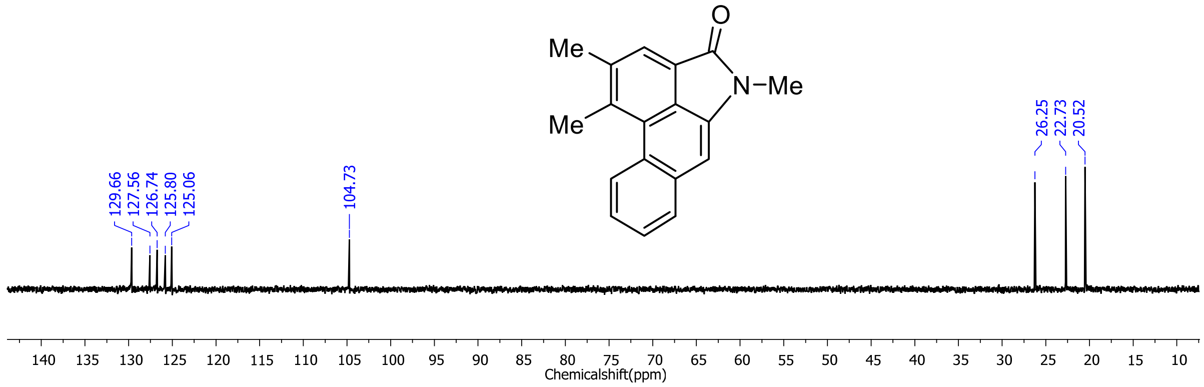

Spectral Data of Compound **9b (major amount).**

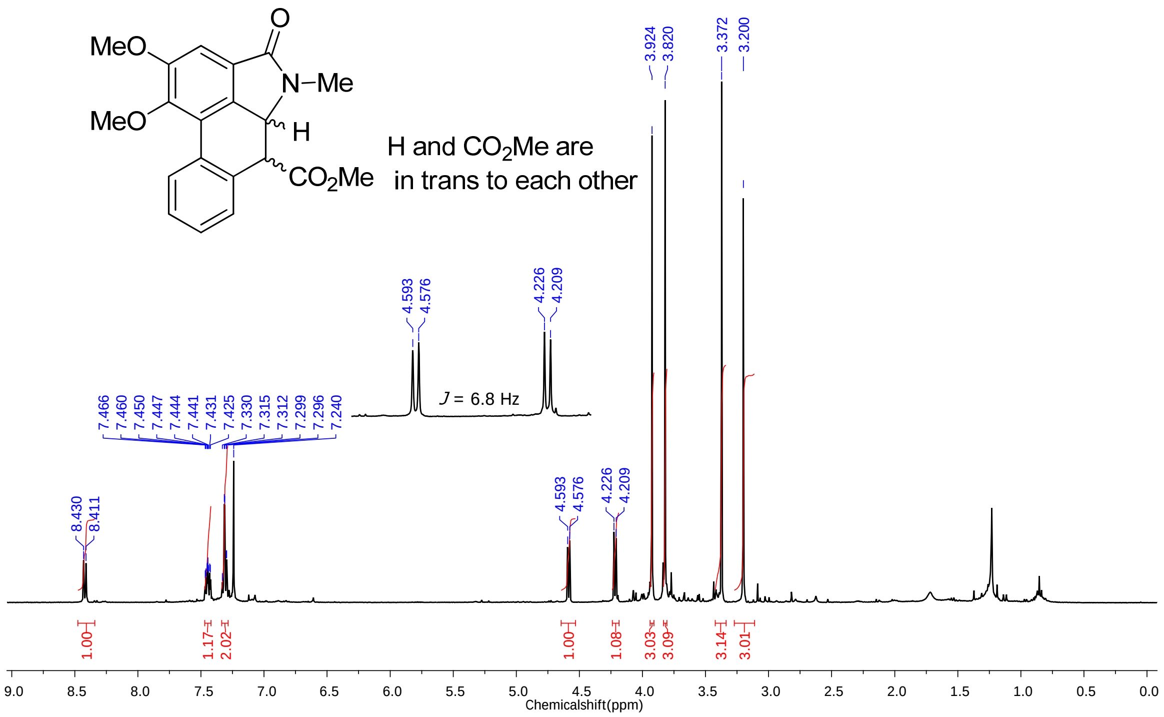

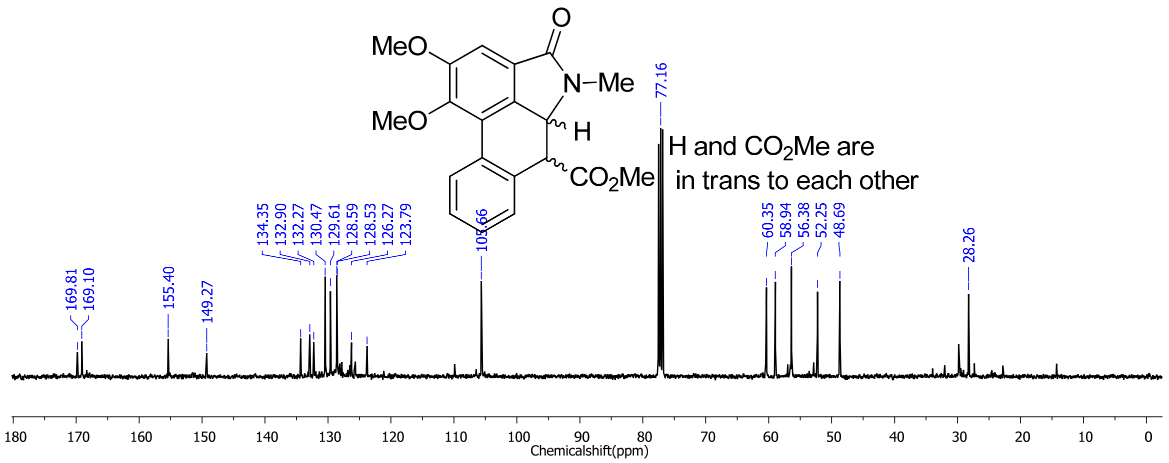

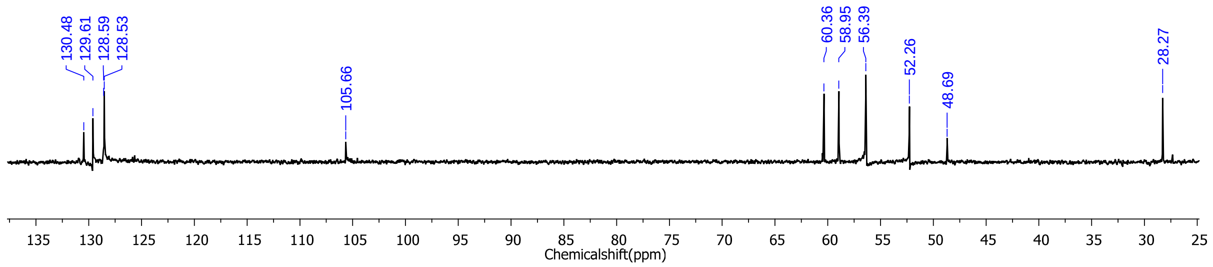

Spectral Data of Compound **9b' (minor isomer).**

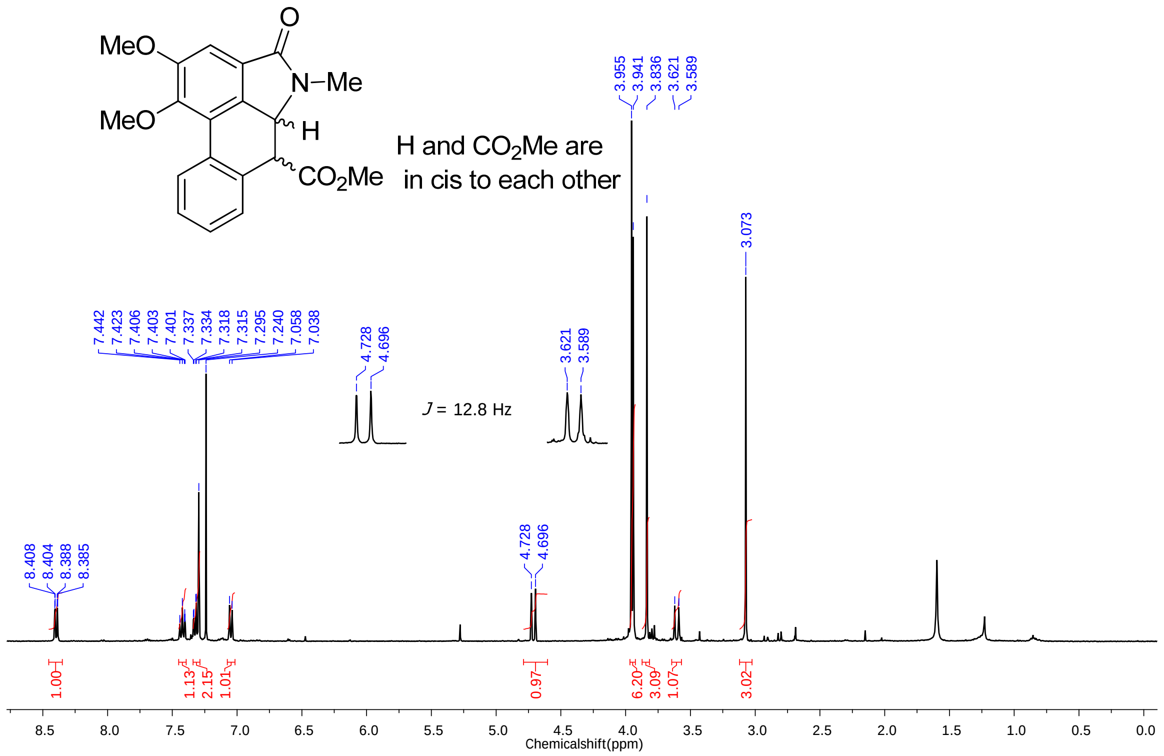

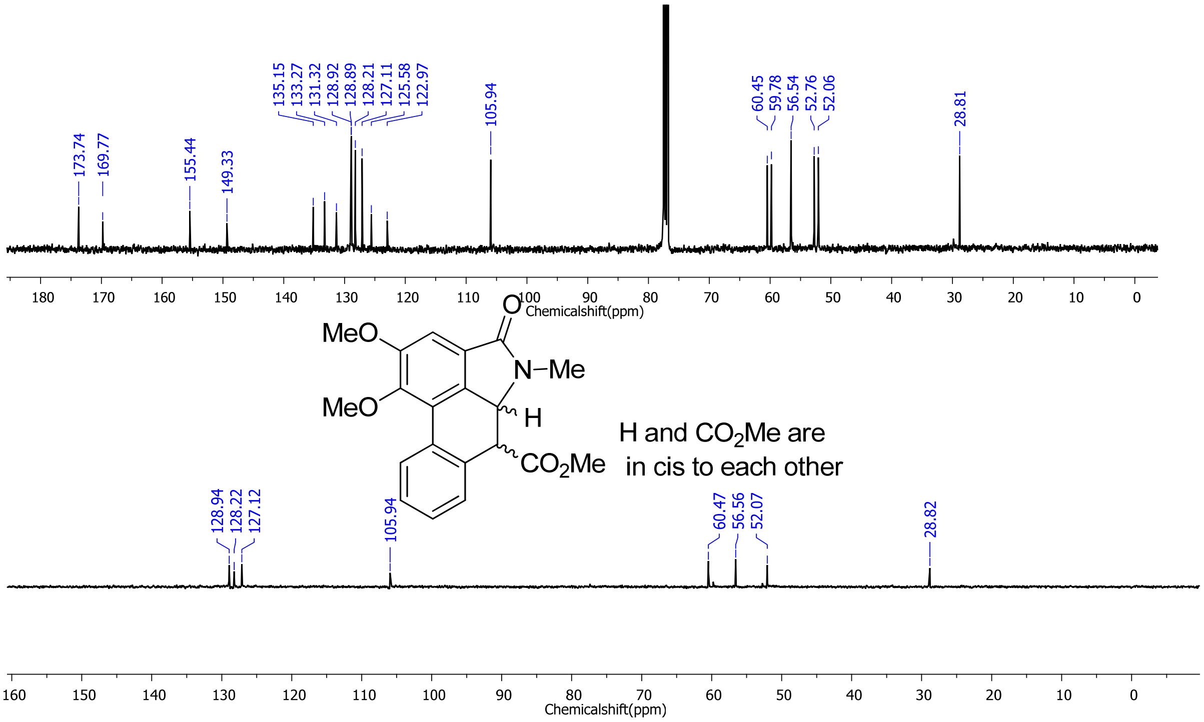

Spectral Data of Compound **9c.**

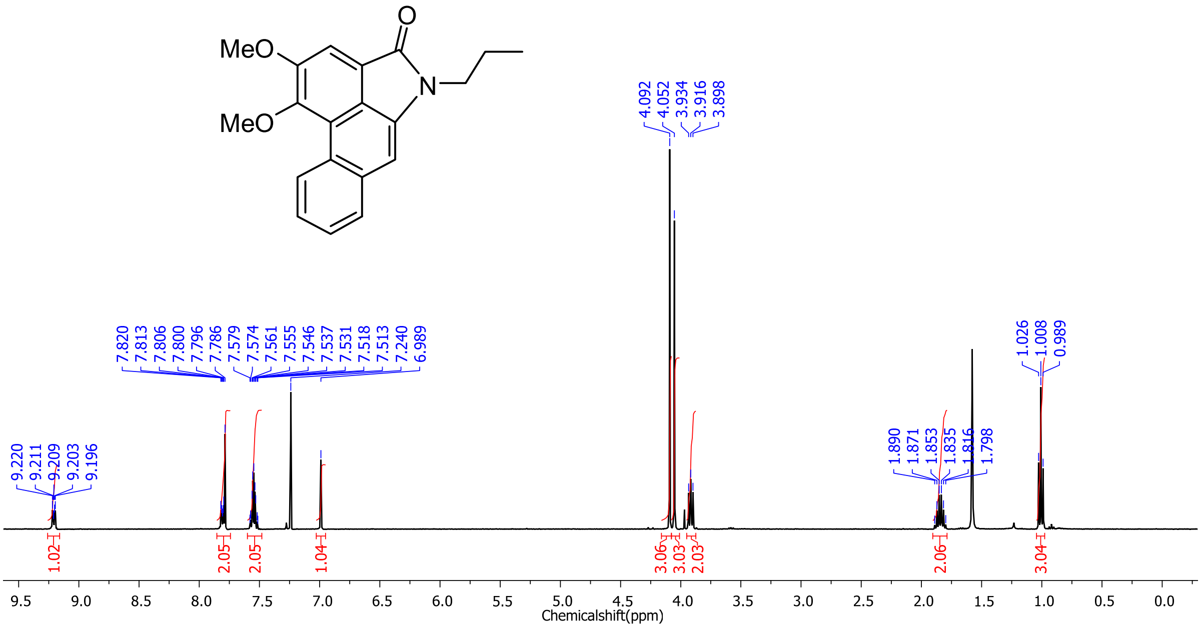

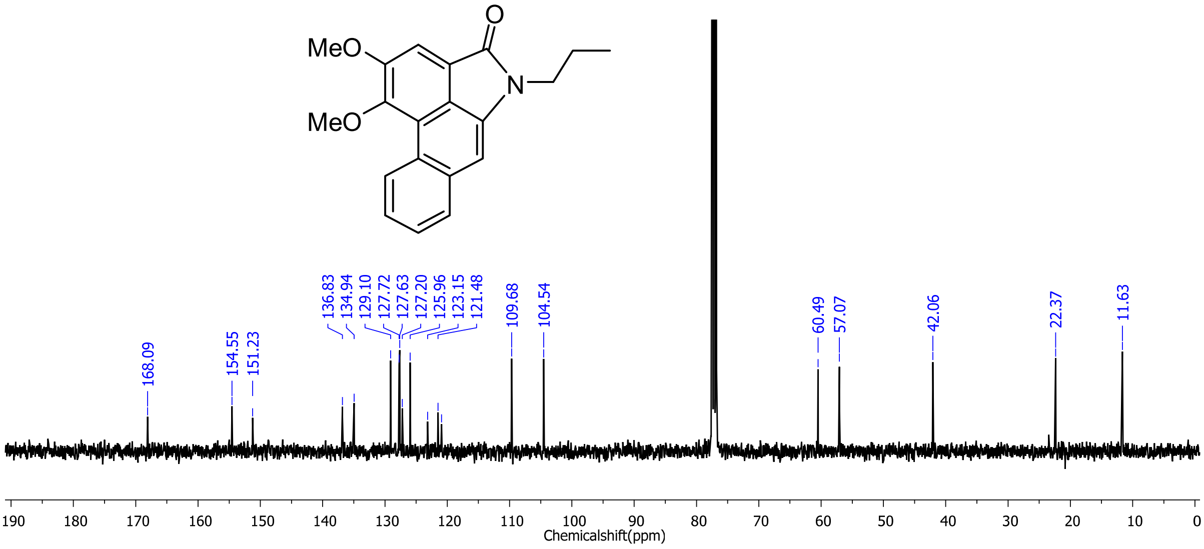

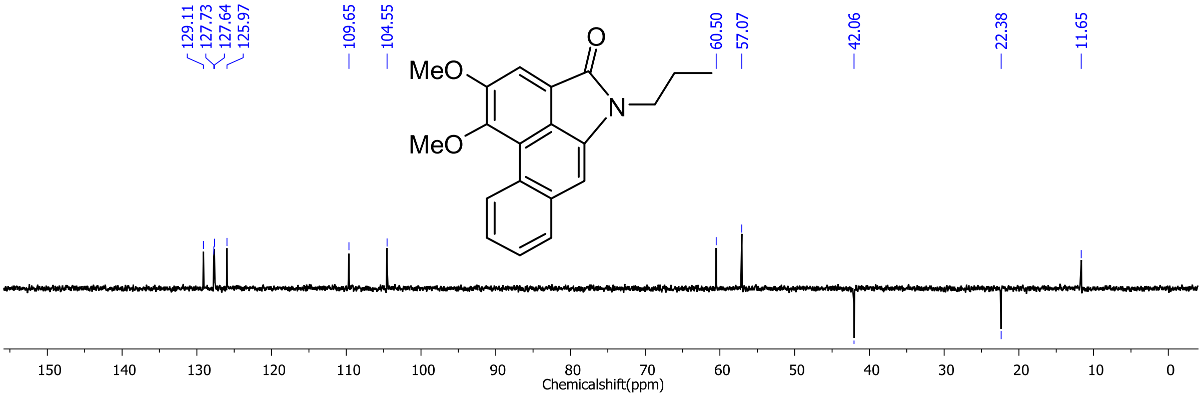

Spectral Data of Compound **9d.**

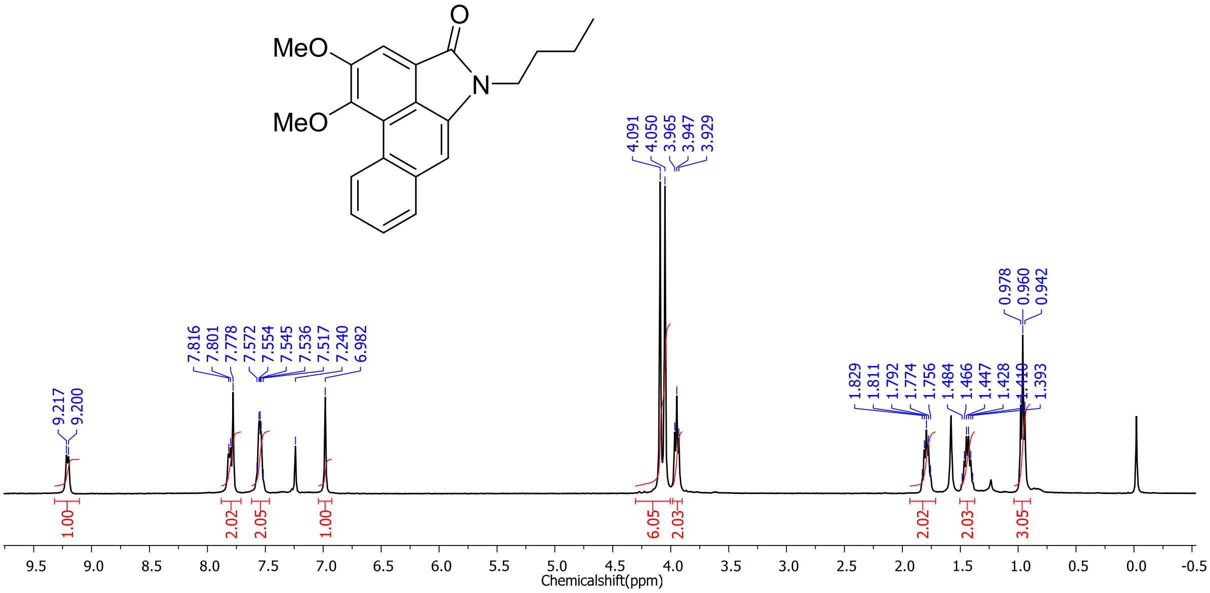

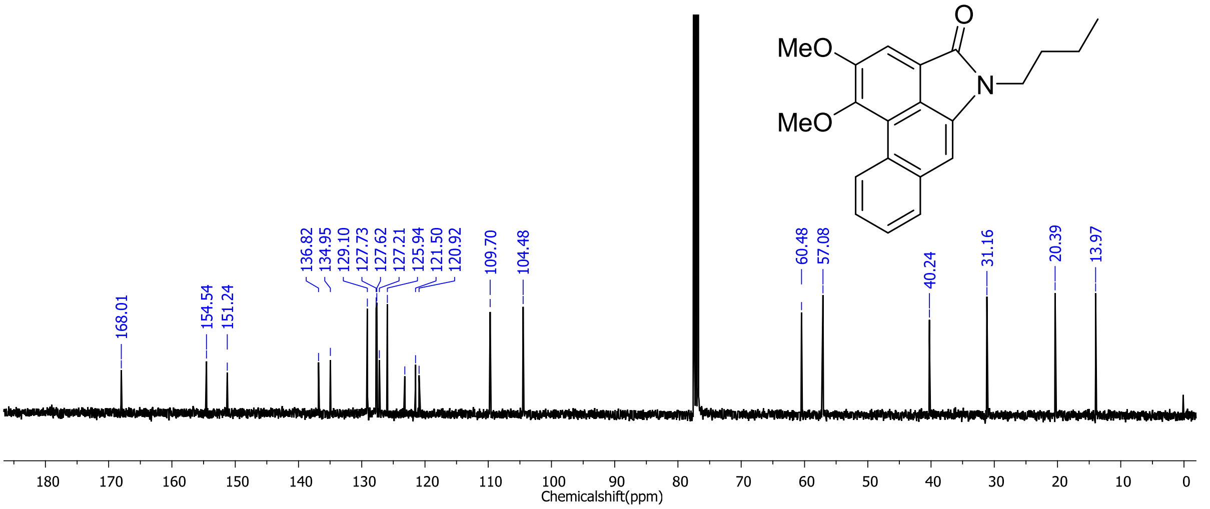

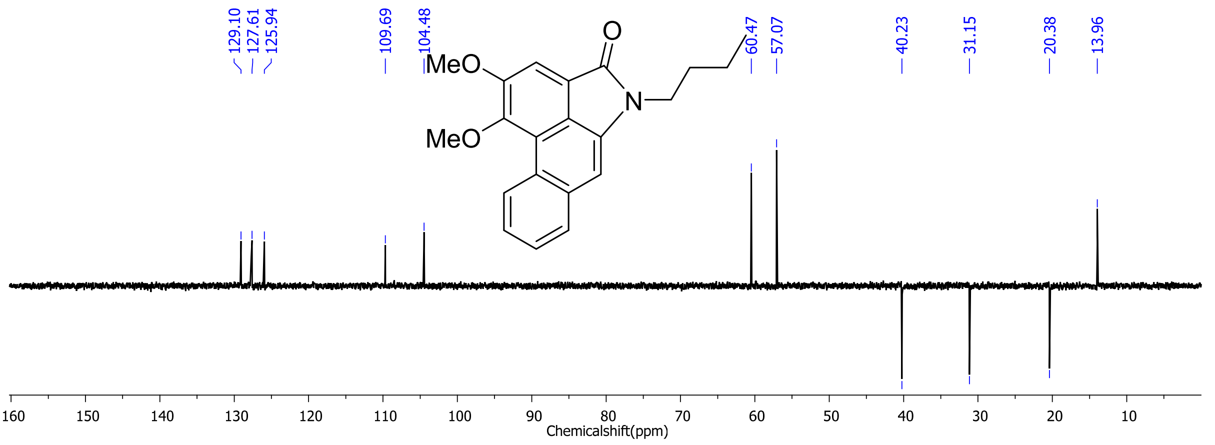

Spectral Data of Compound **9e.**

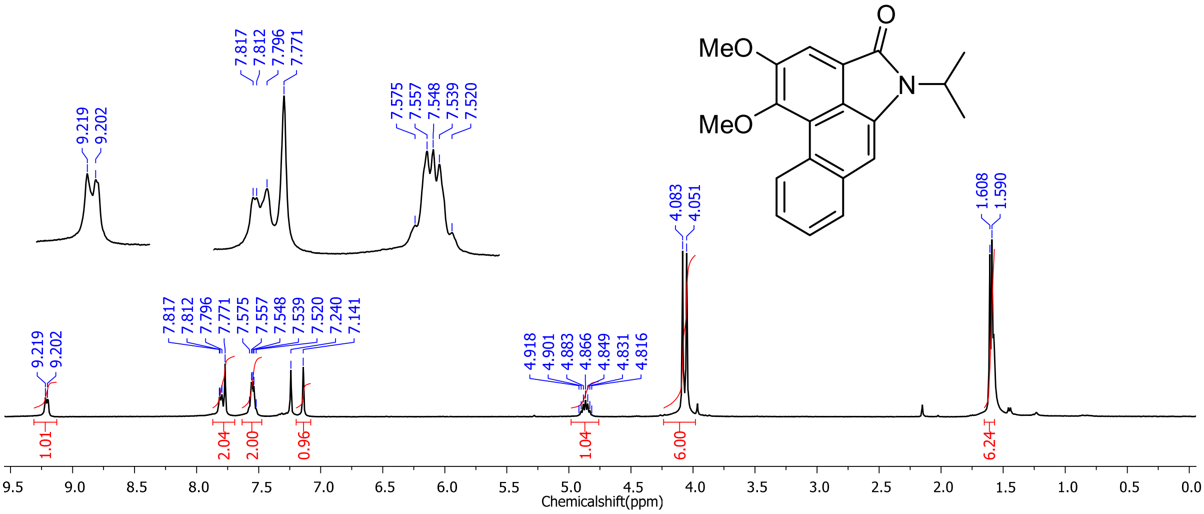

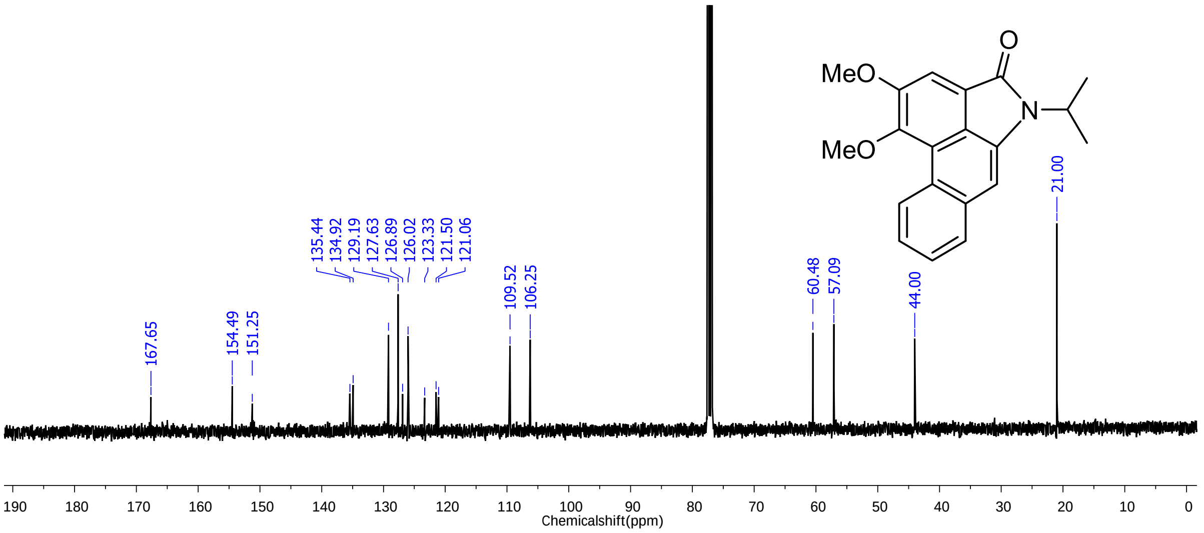

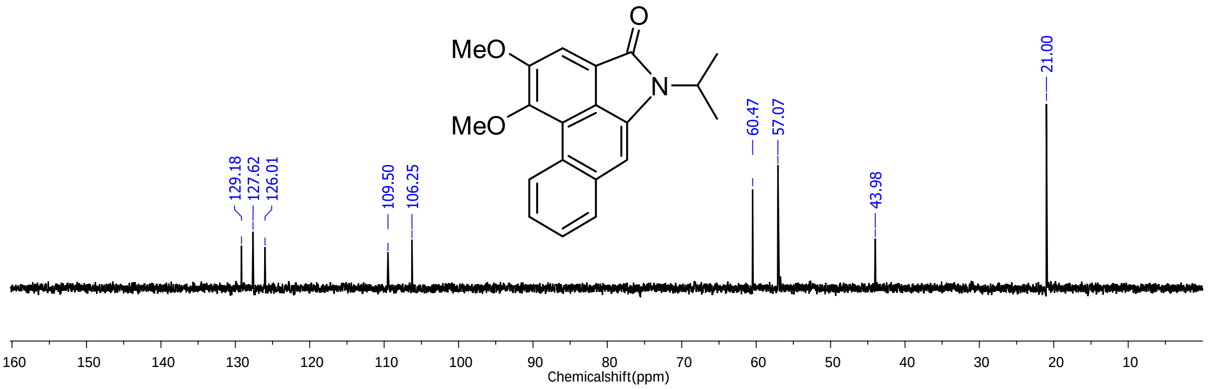

Spectral Data of Compound **9f.**

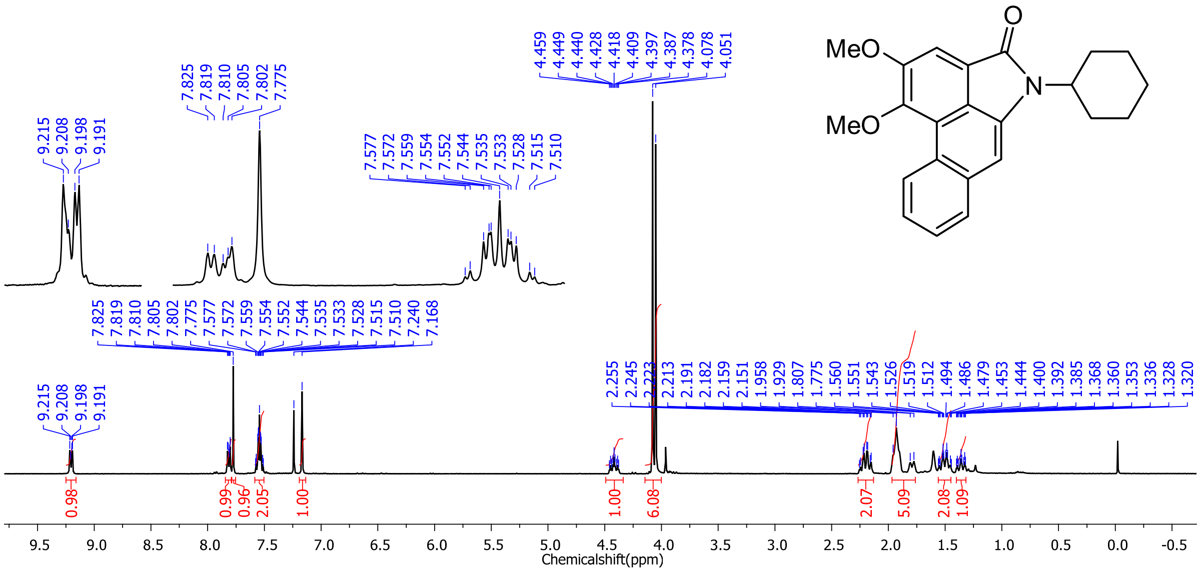

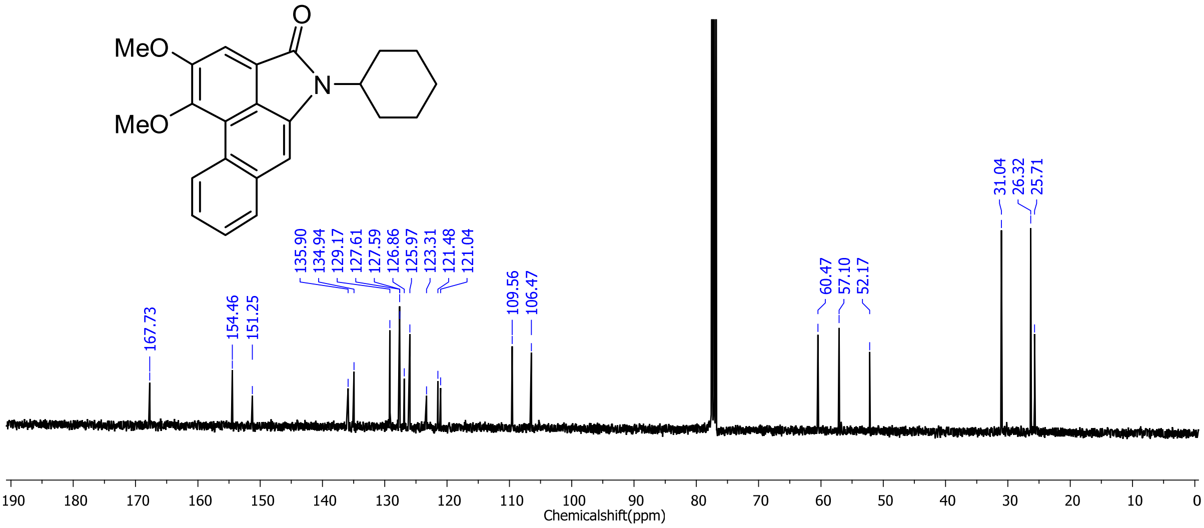

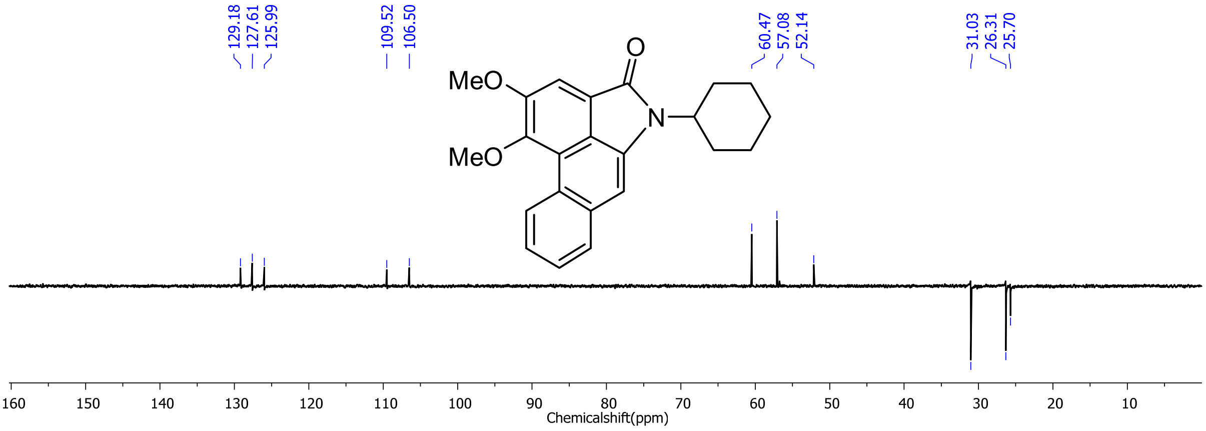

Spectral Data of Compound **9g.**

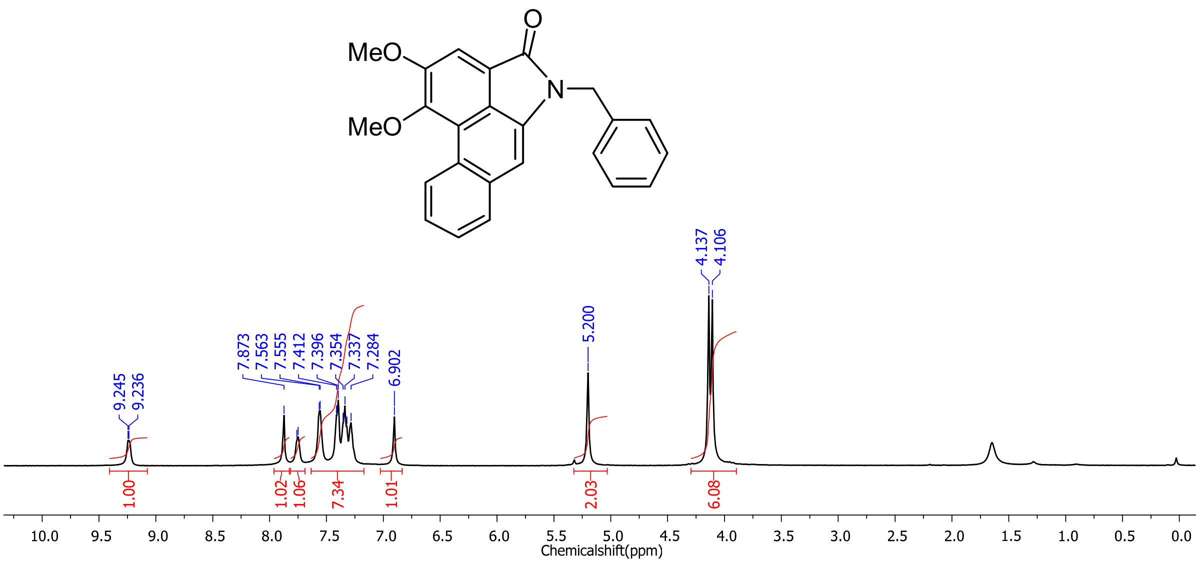

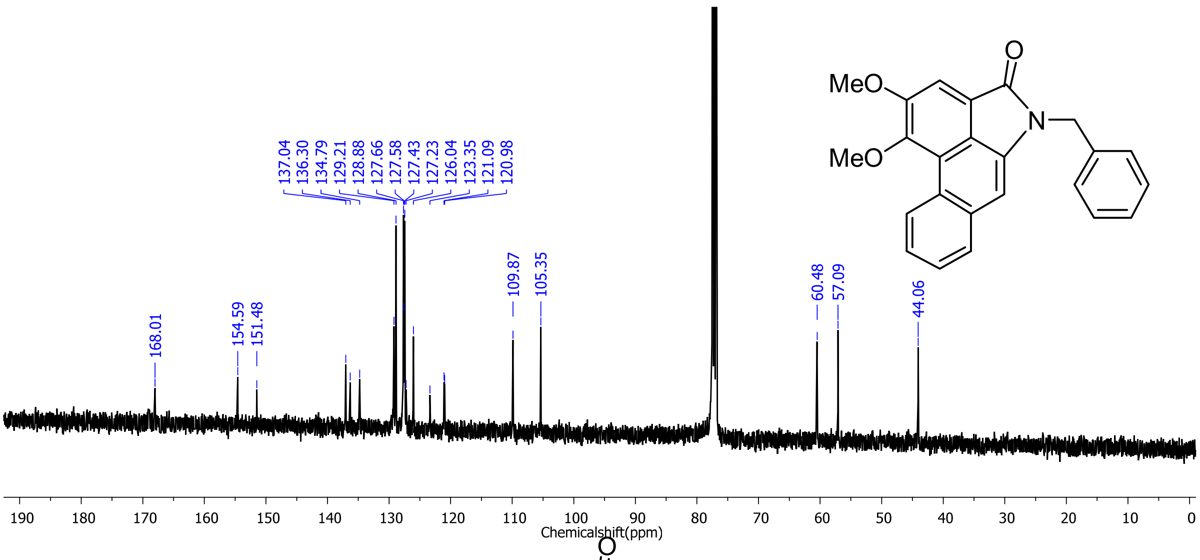

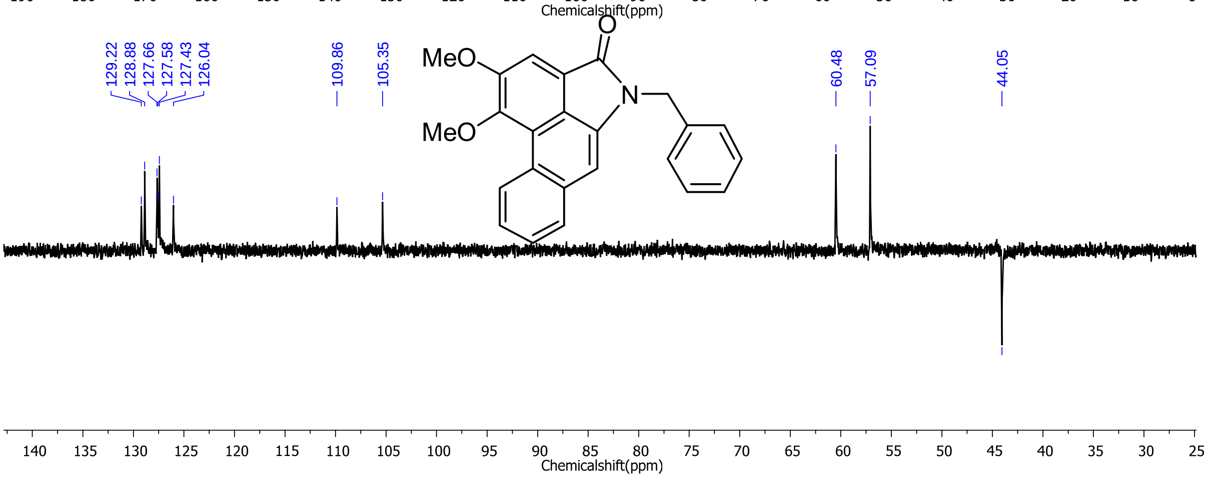

Spectral Data of Compound **9h.**

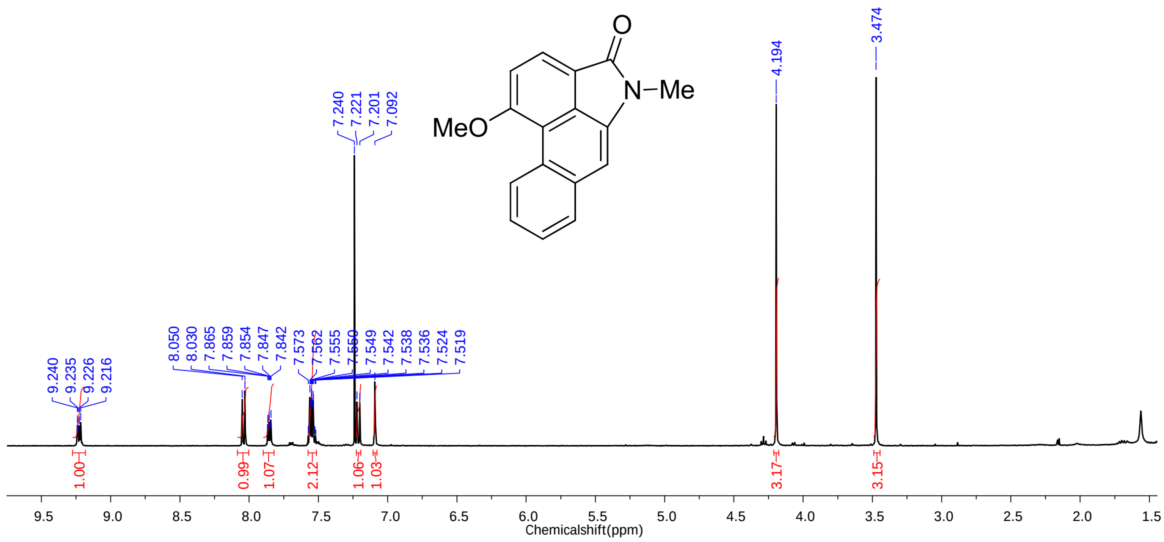

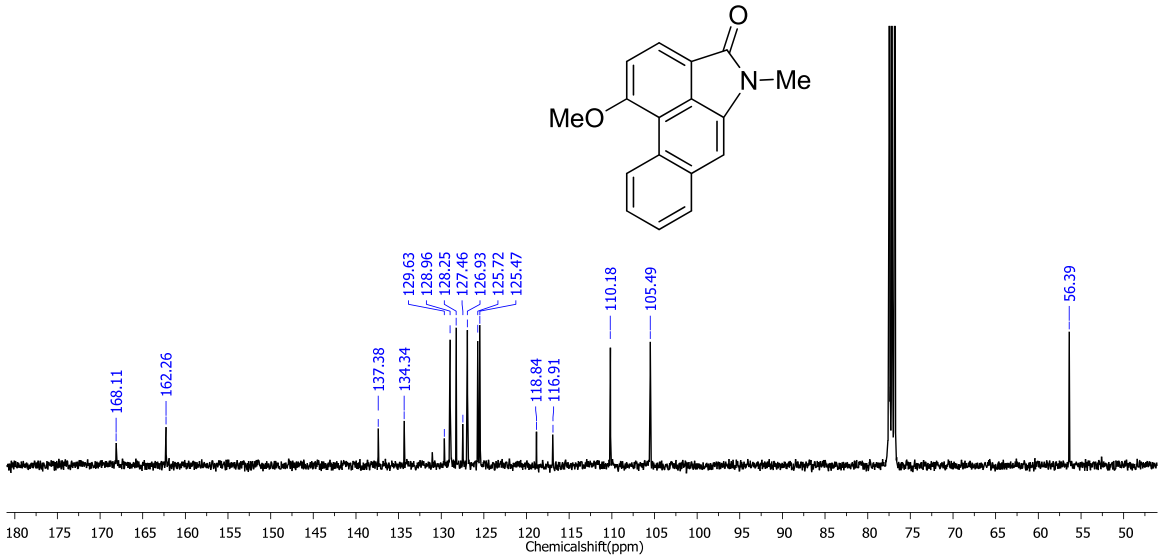

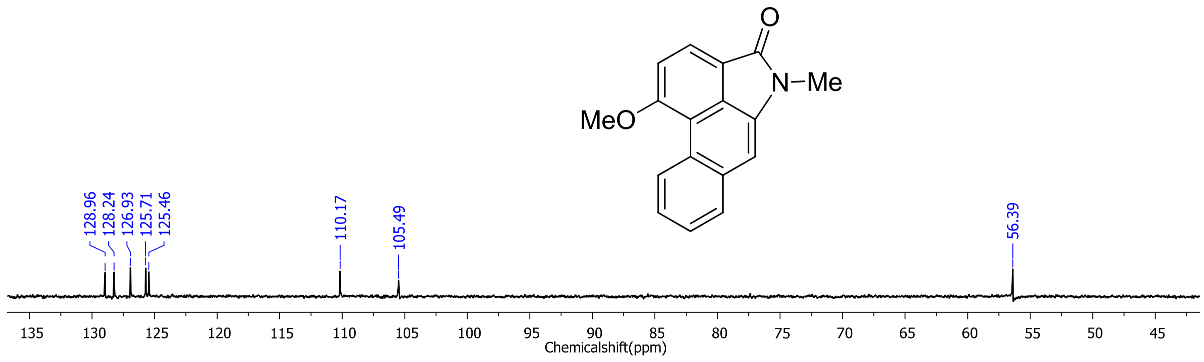

Spectral Data of Compound **9i.**

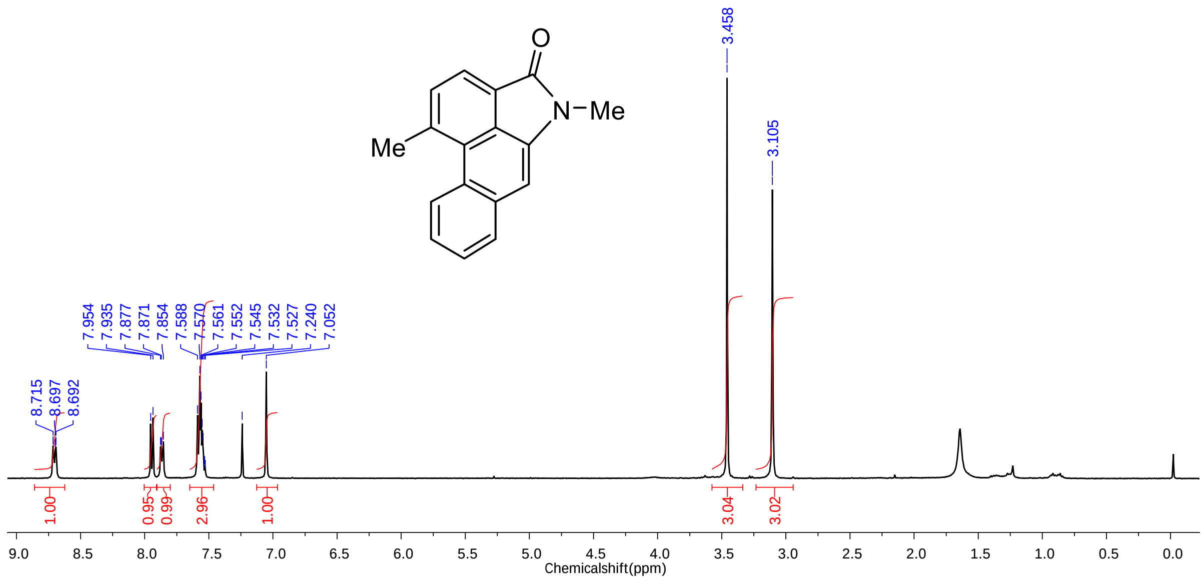

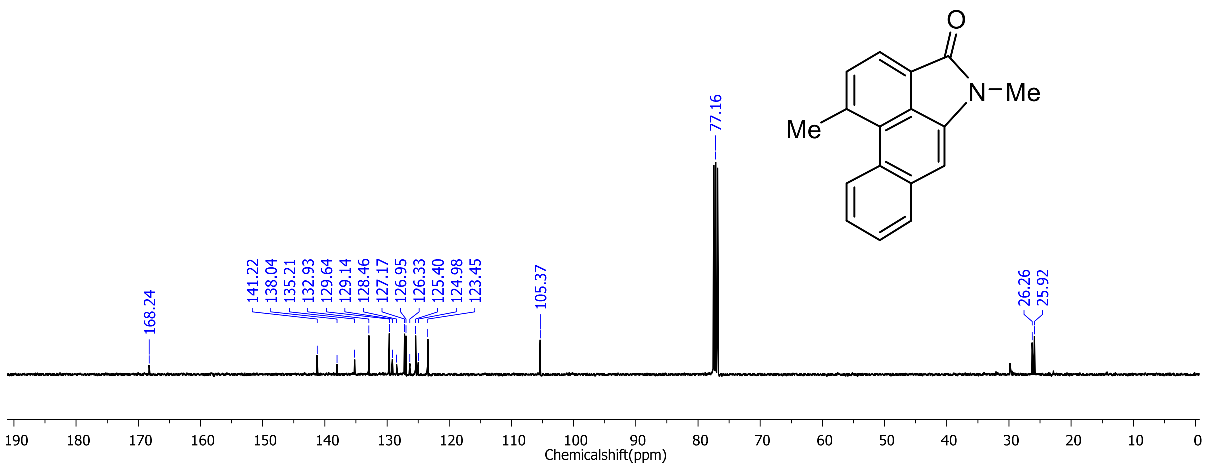

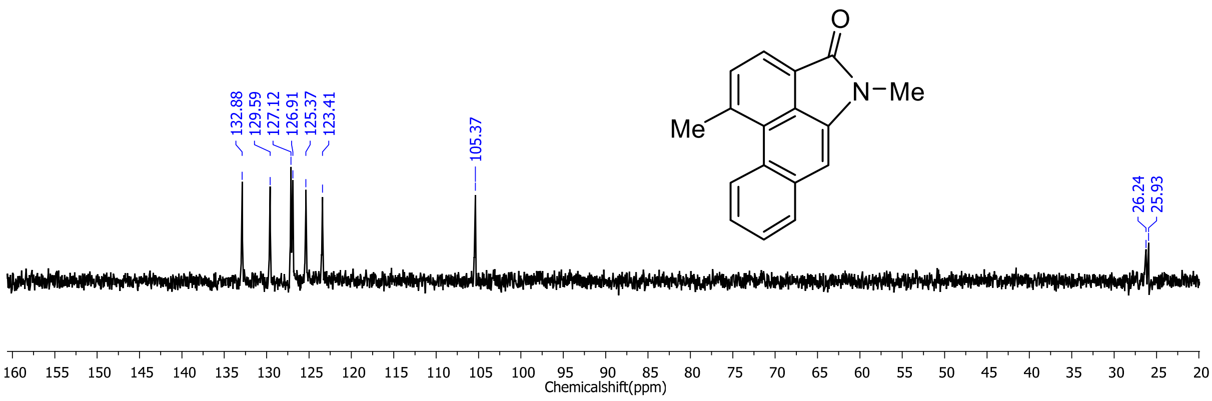

Spectral Data of Compound **9j.**

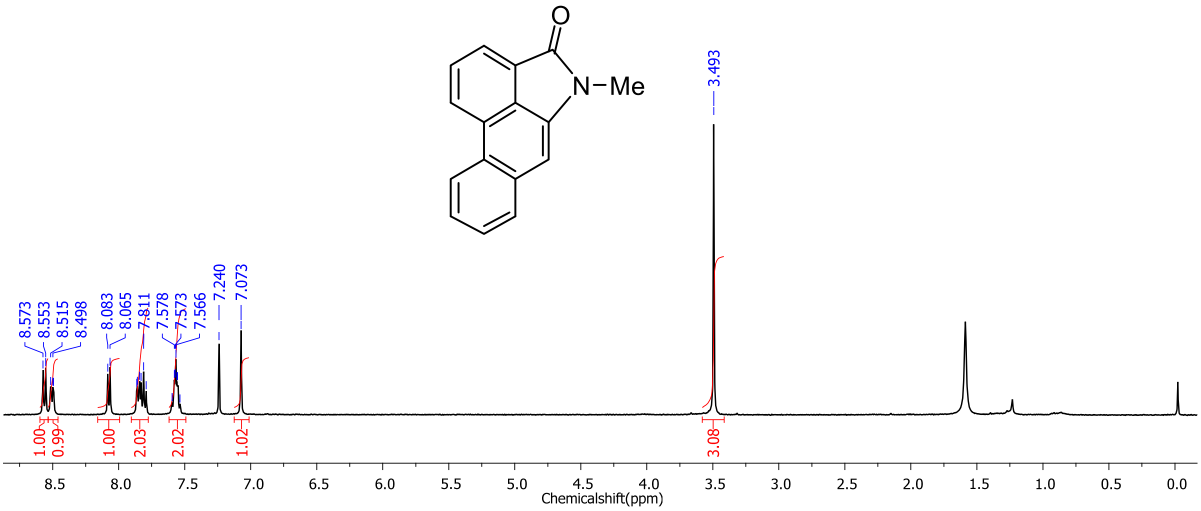

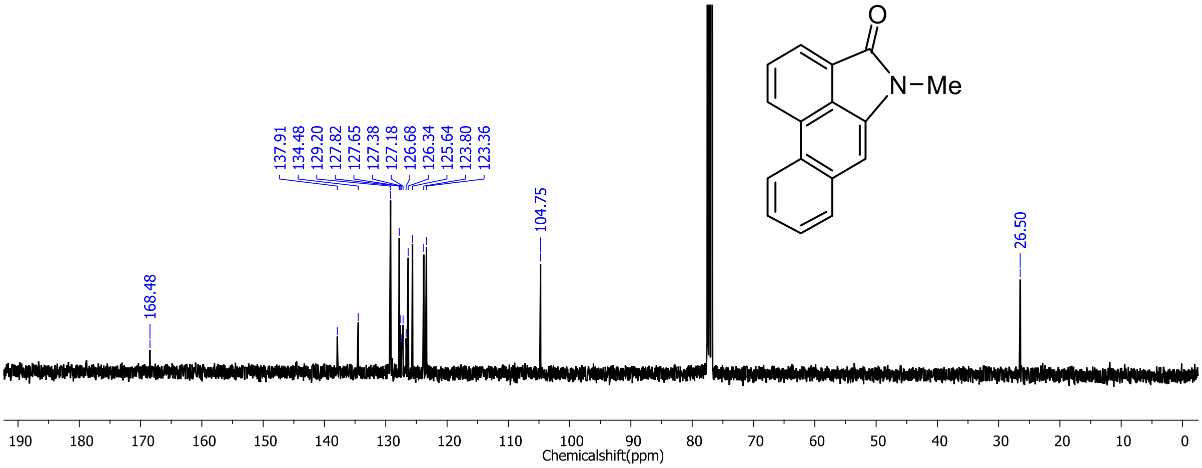

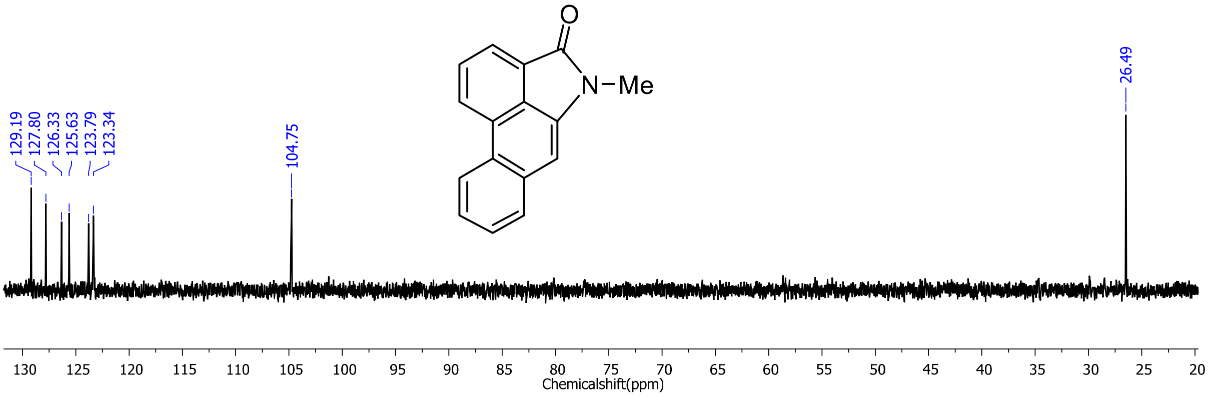

Spectral Data of Compound **9k.**

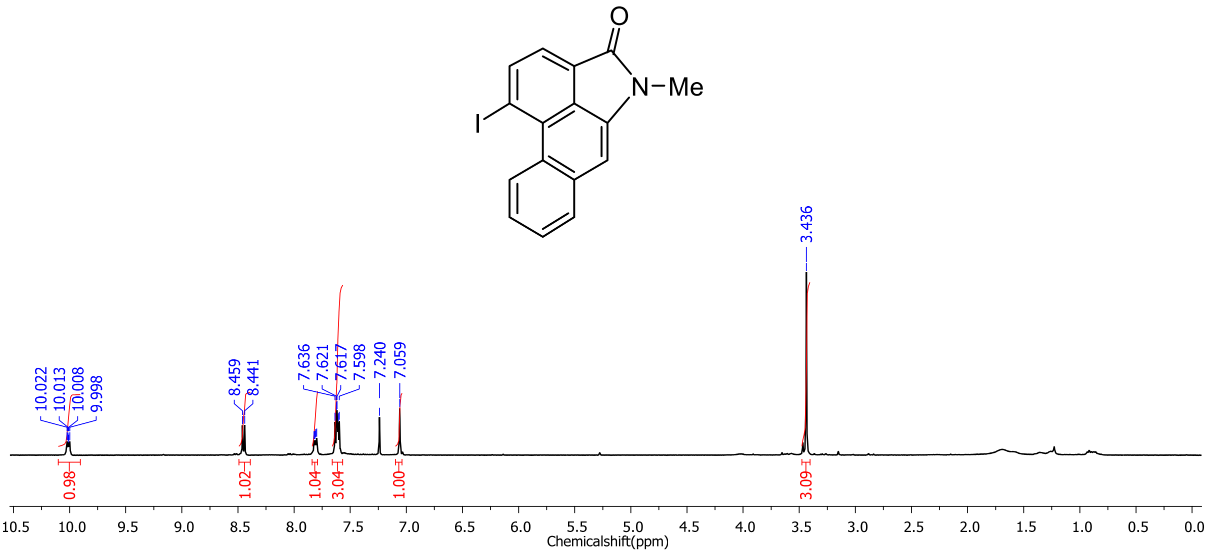

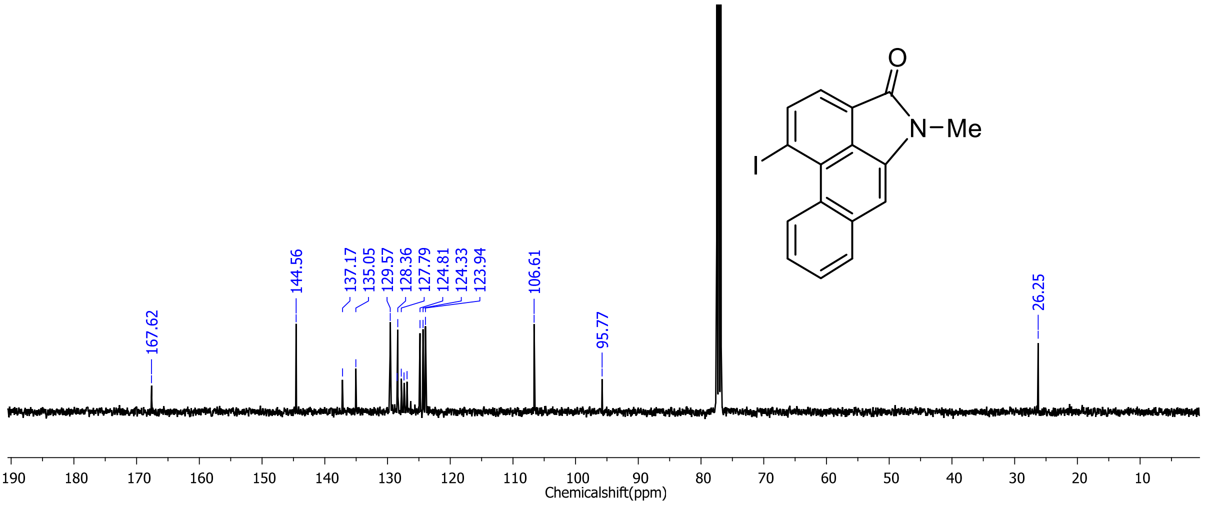

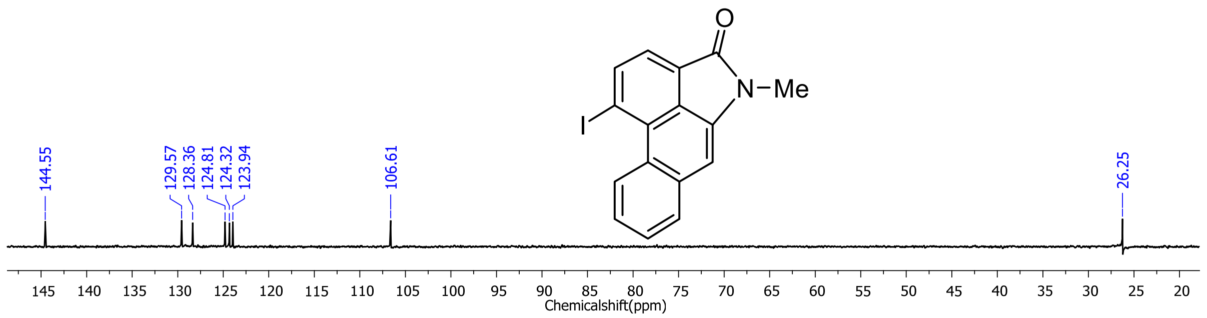

Spectral Data of Compound **9l.**

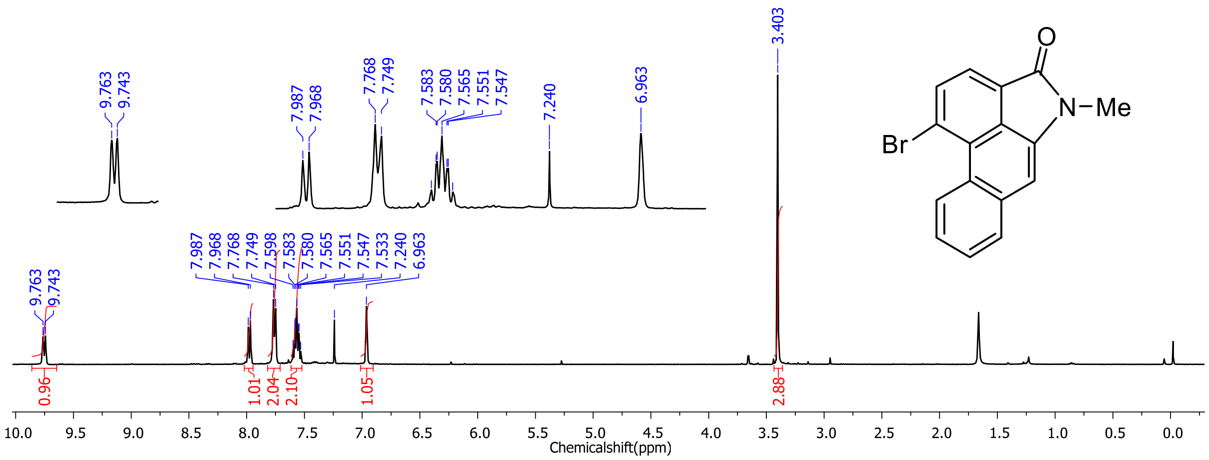

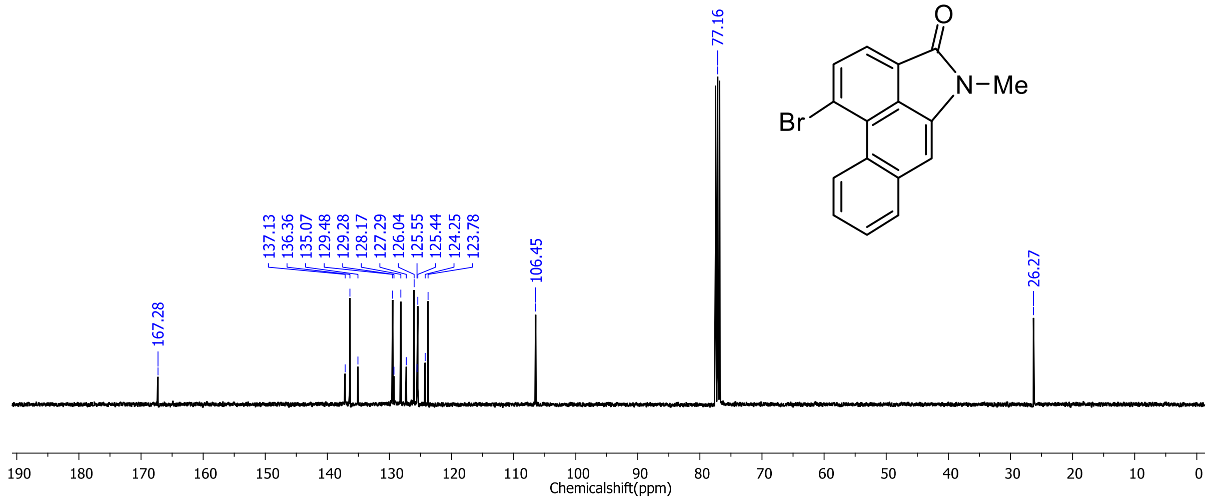

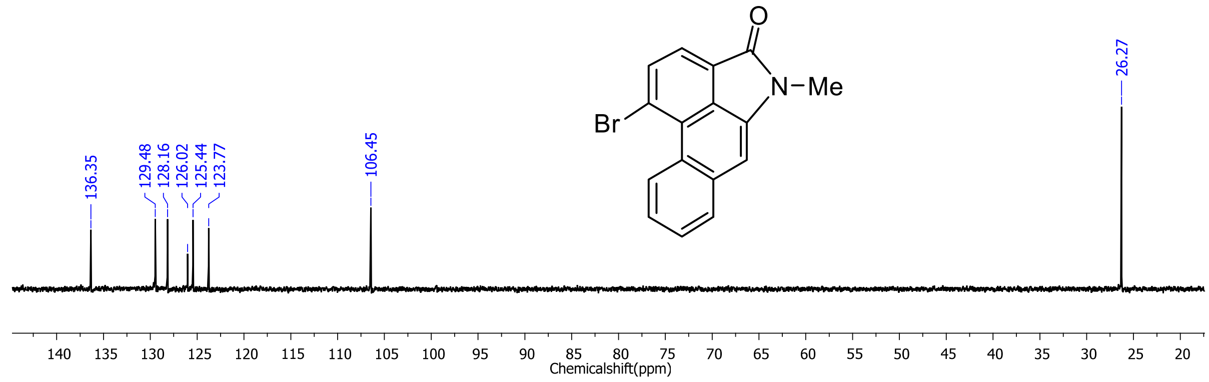

Spectral Data of Compound **9m.**

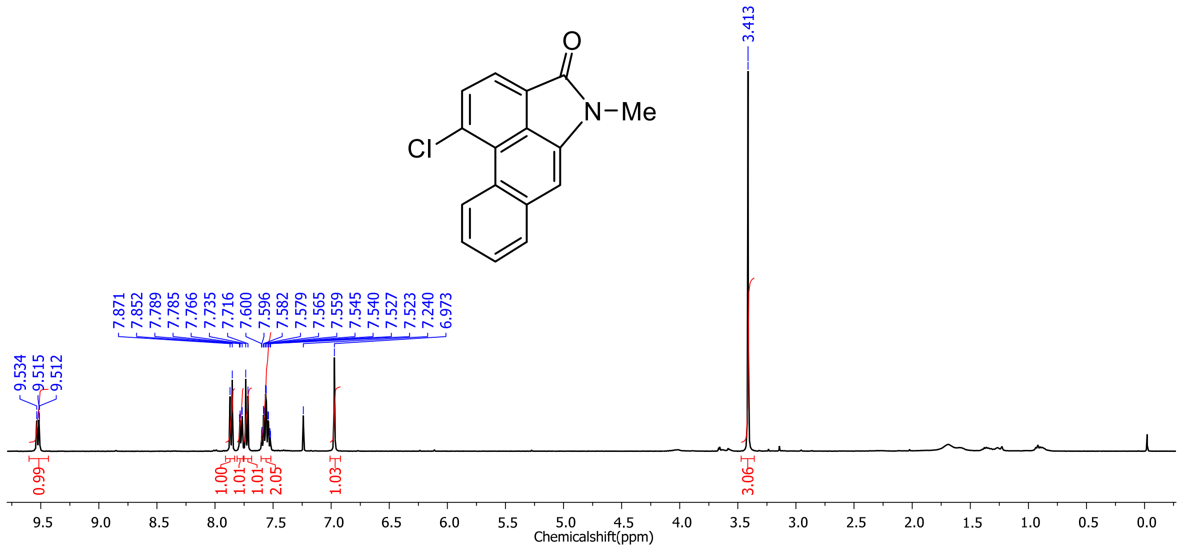

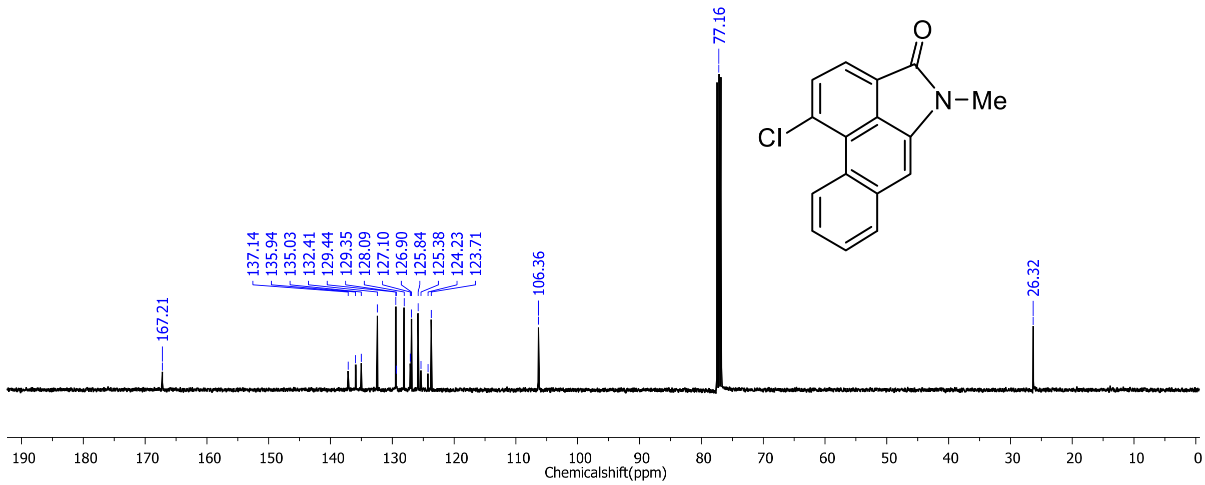

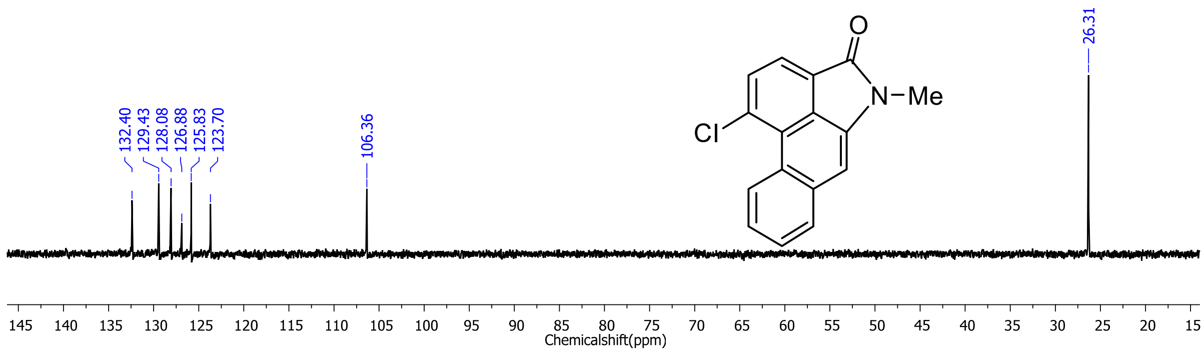

Spectral Data of Compound **9n.**

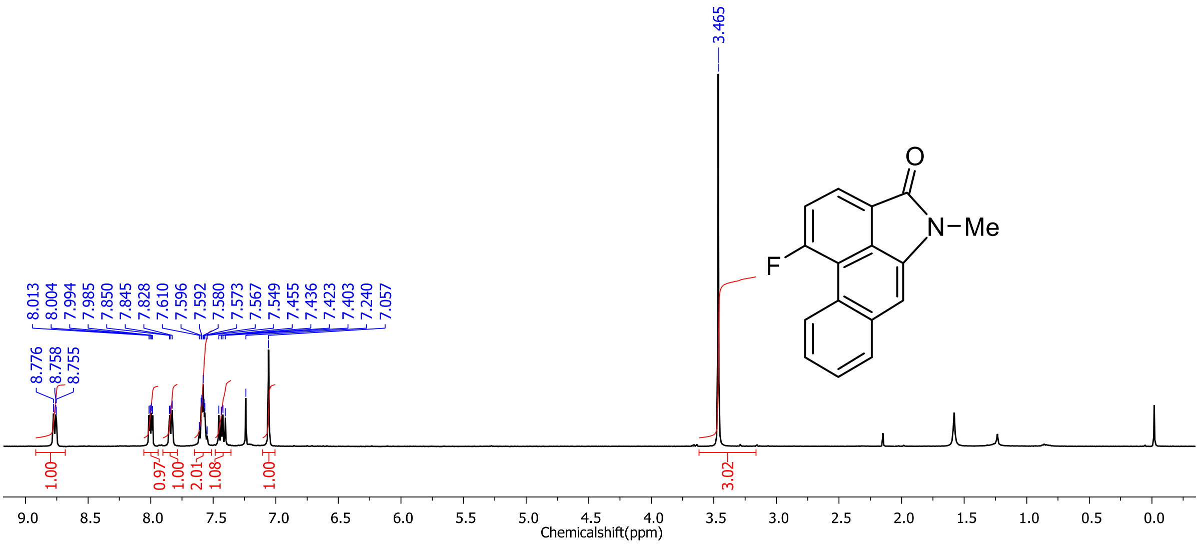

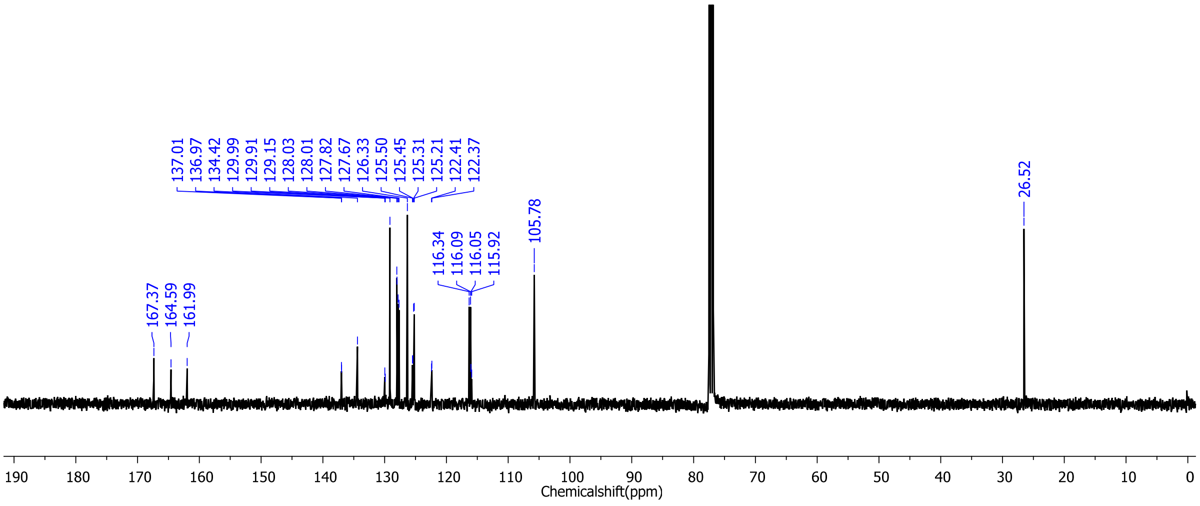

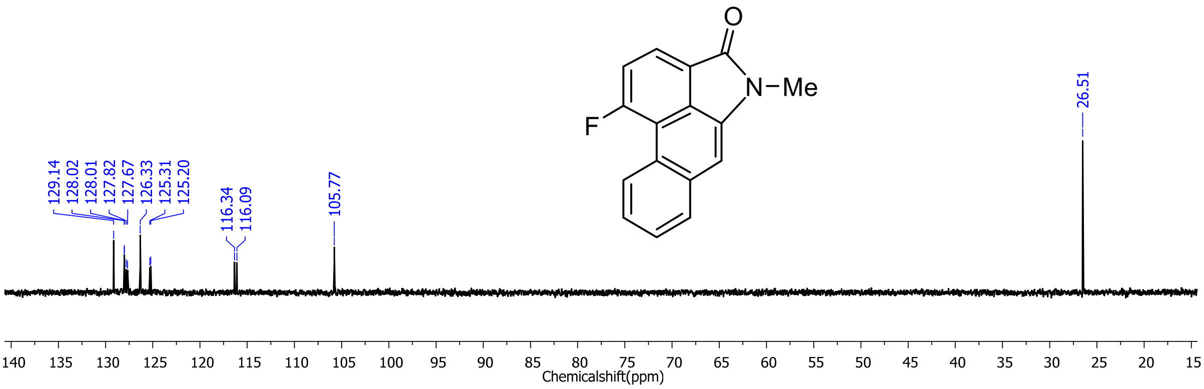

Spectral Data of Compound **9o.**

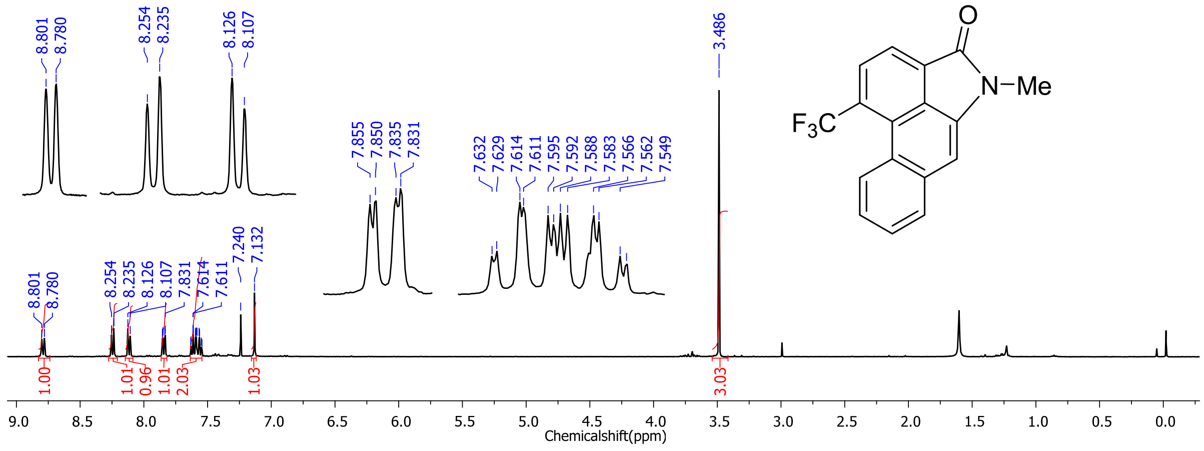

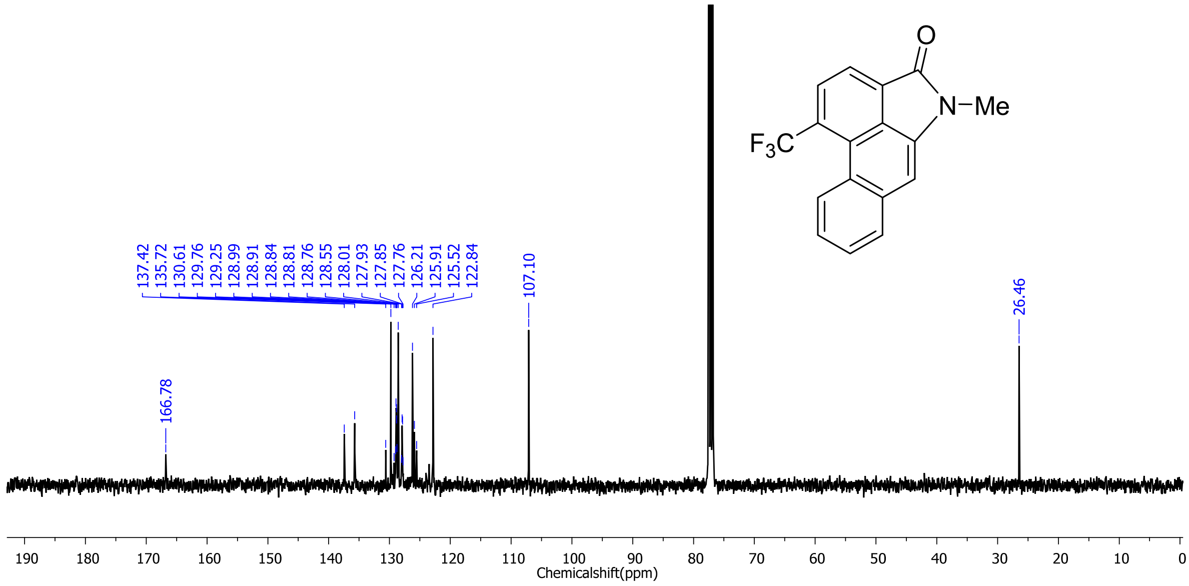

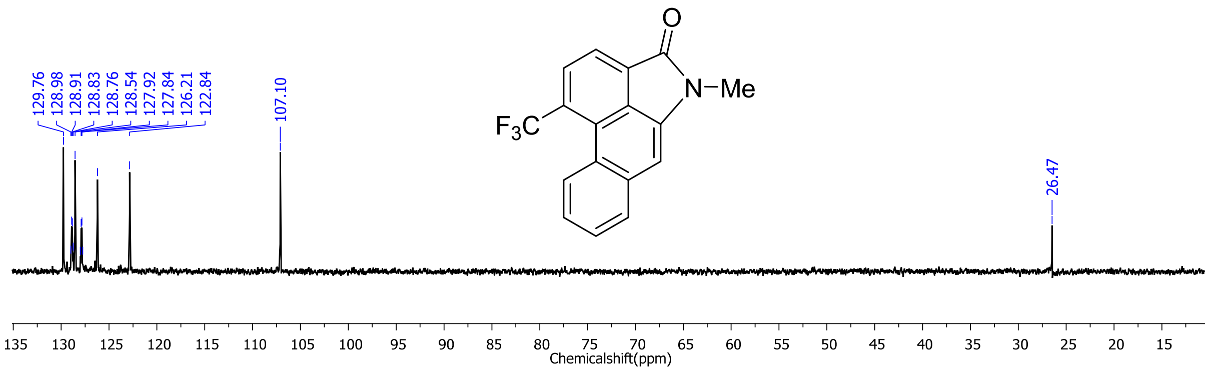

Spectral Data of Compound **9p.**

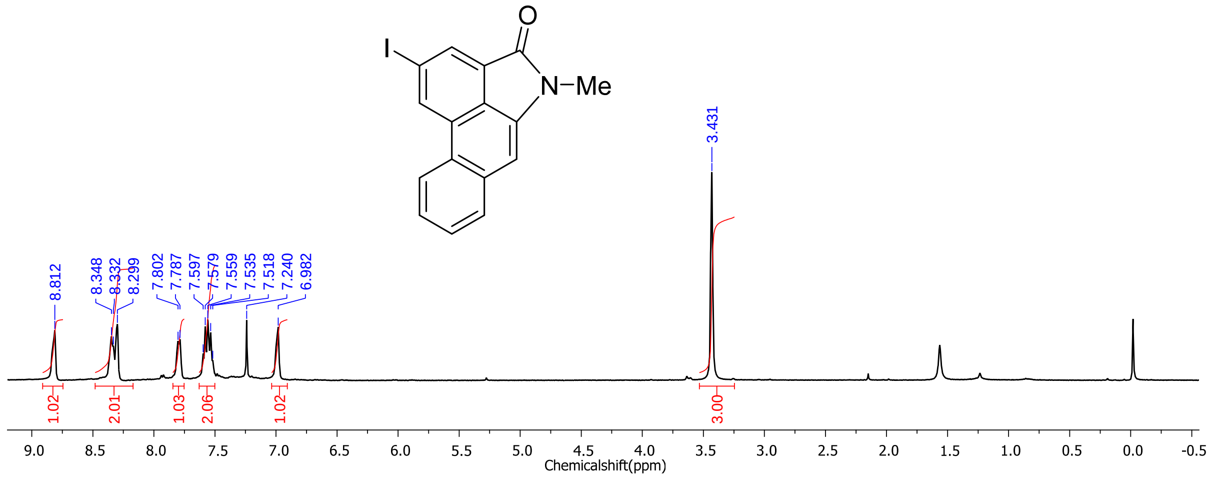

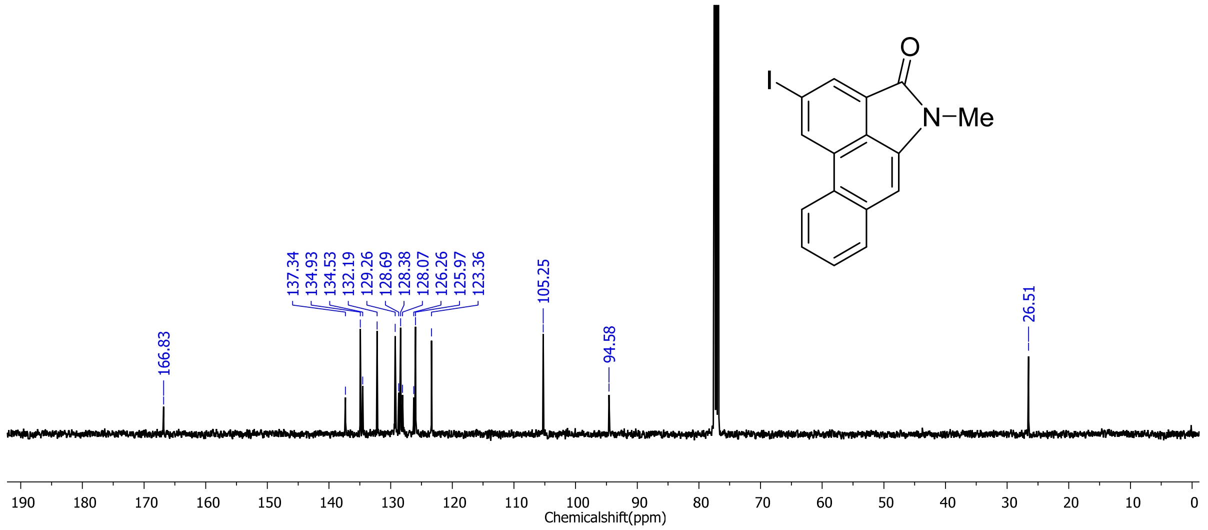

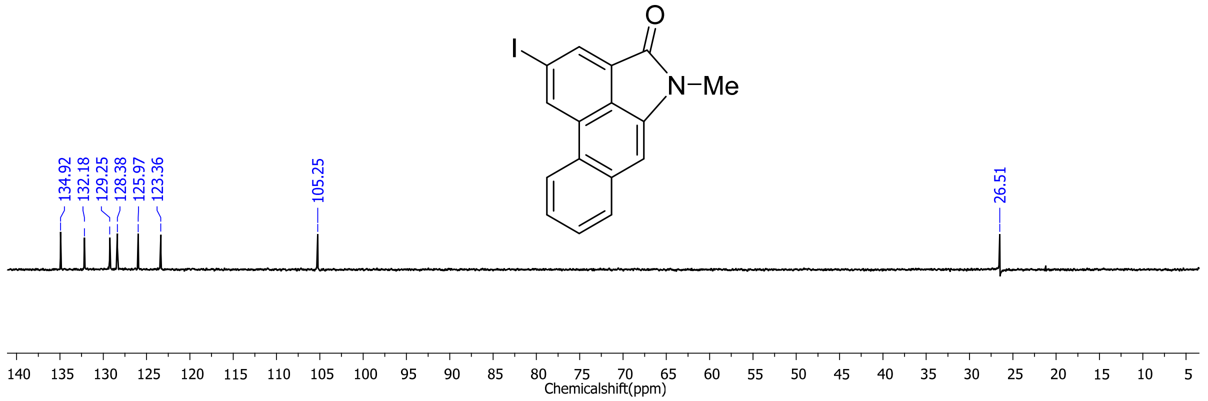

Spectral Data of Compound **9q.**

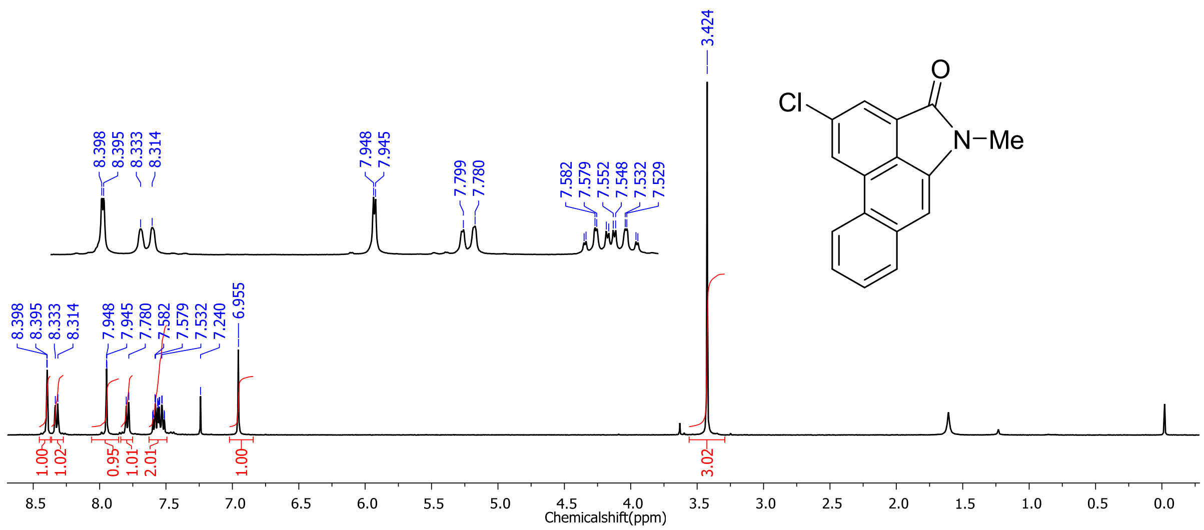

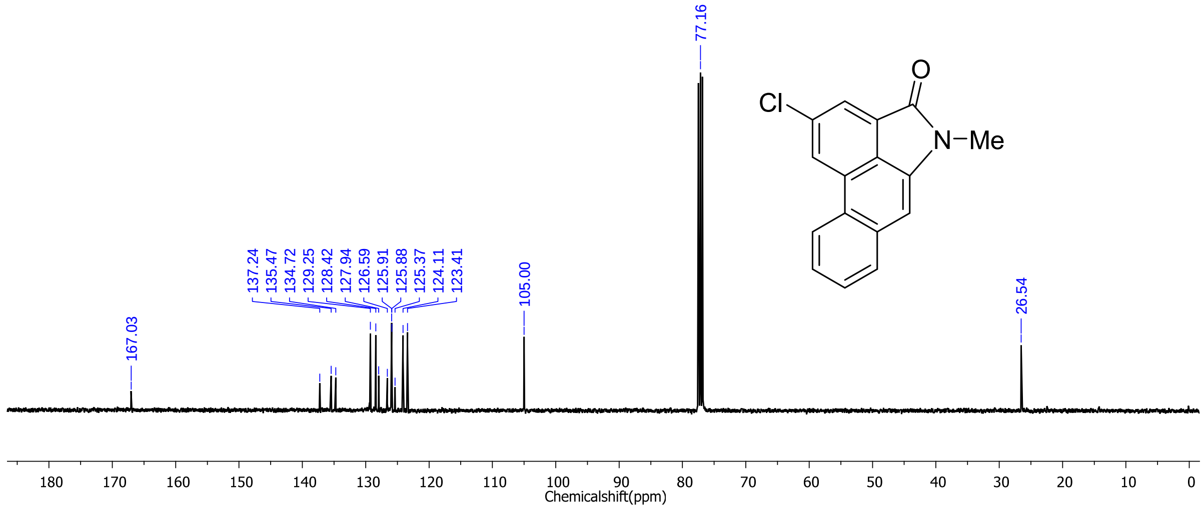

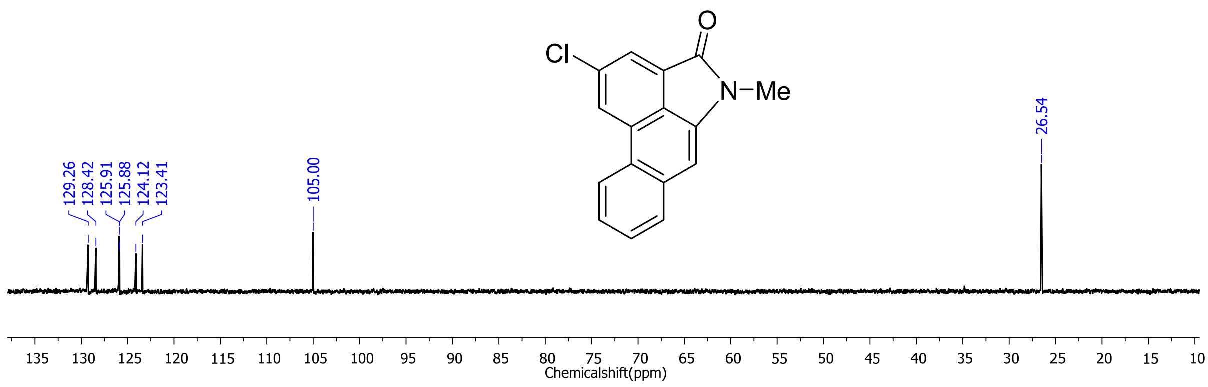

Spectral Data of Compound **9r.**

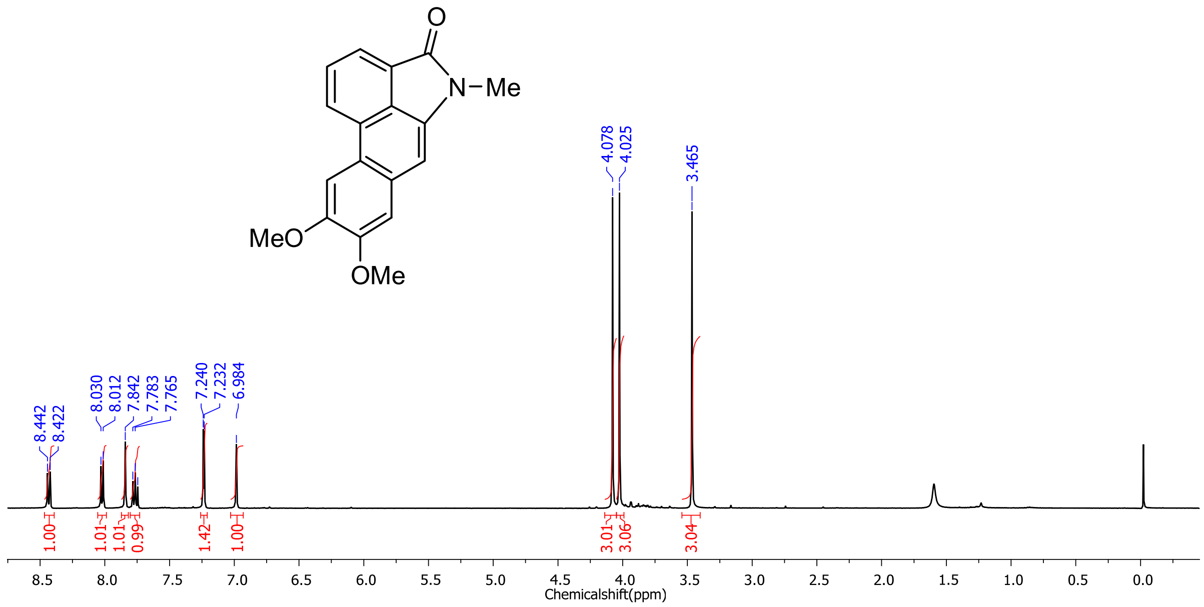

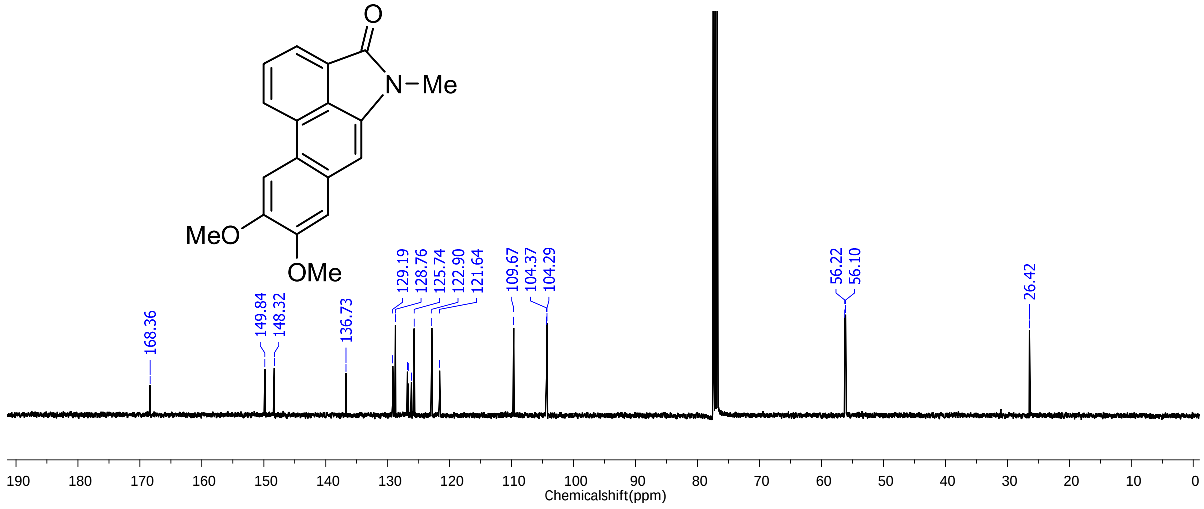

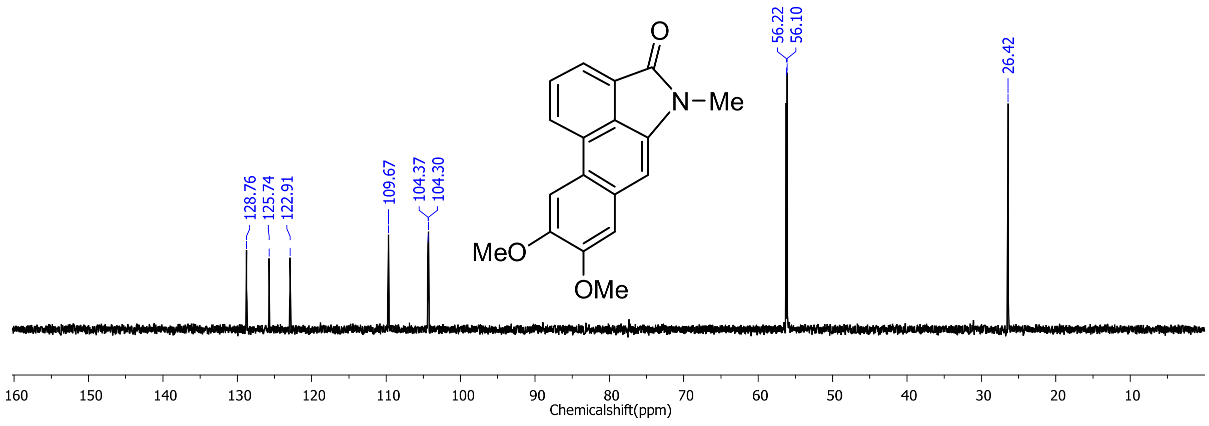

Spectral Data of Compound **9s.**

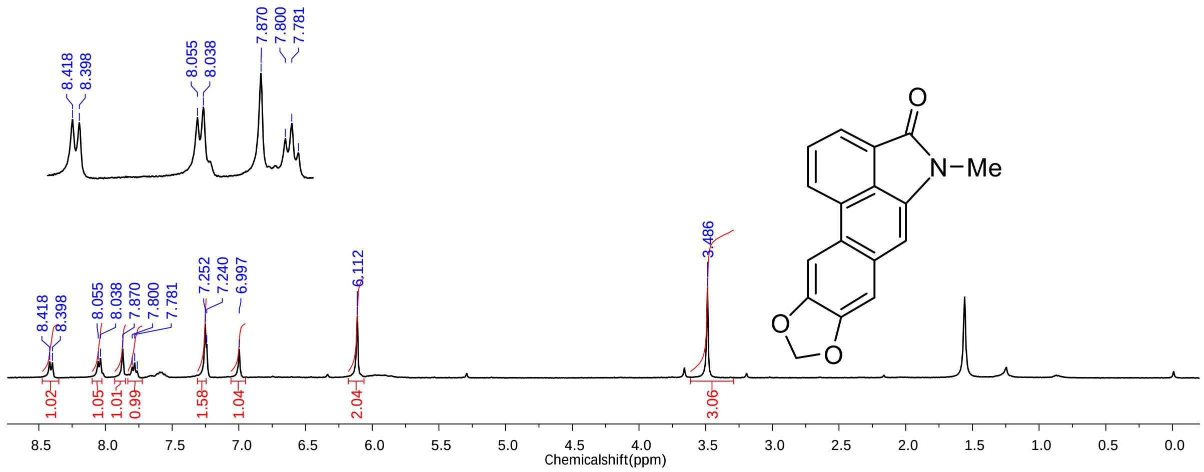

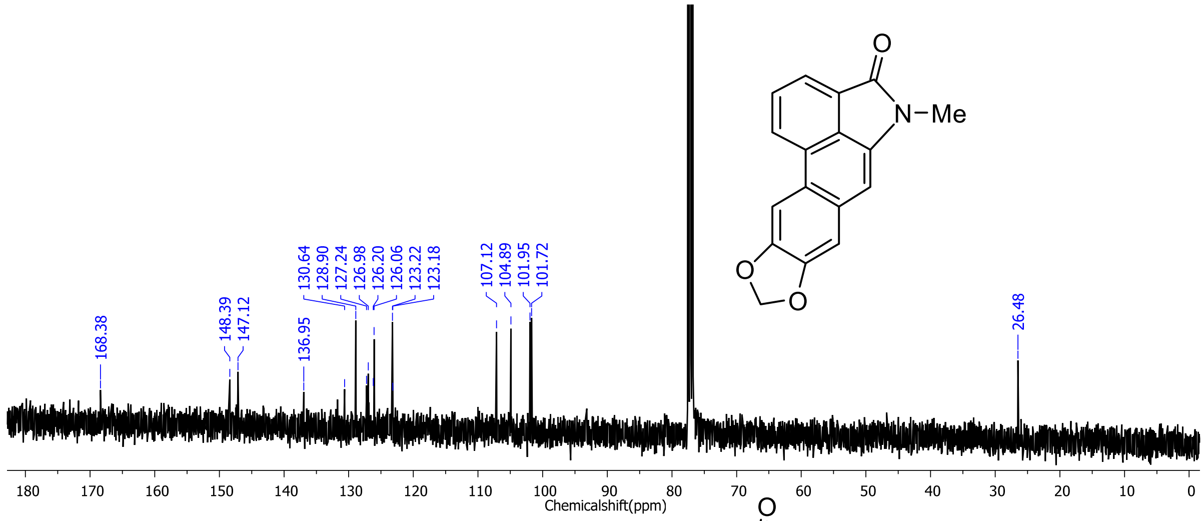

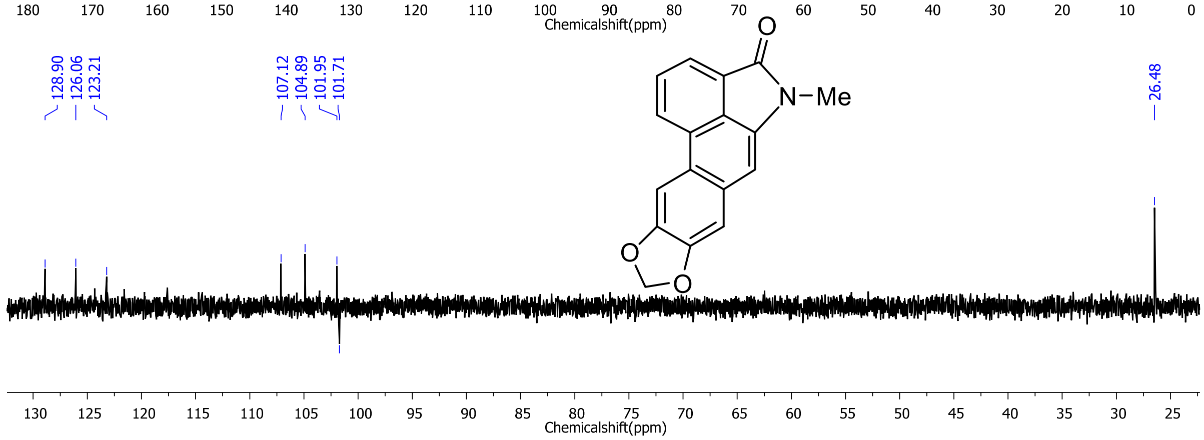

Spectral Data of Compound **9t.**

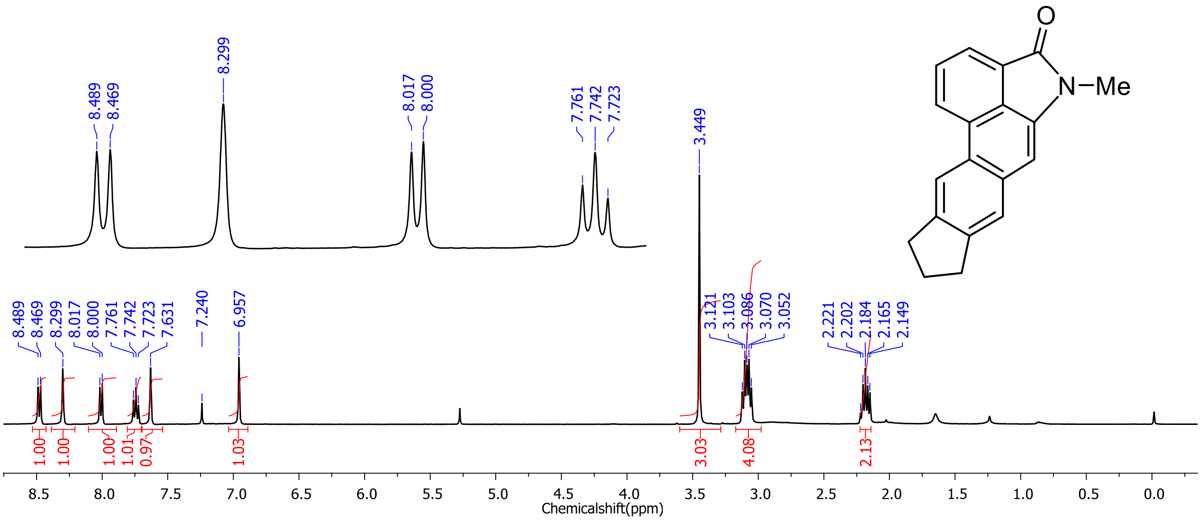

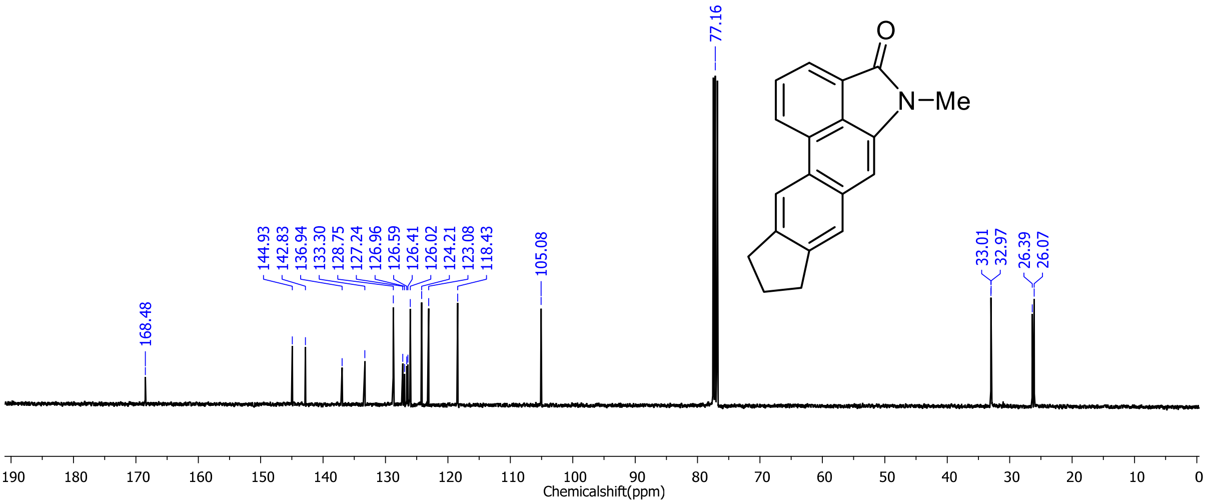

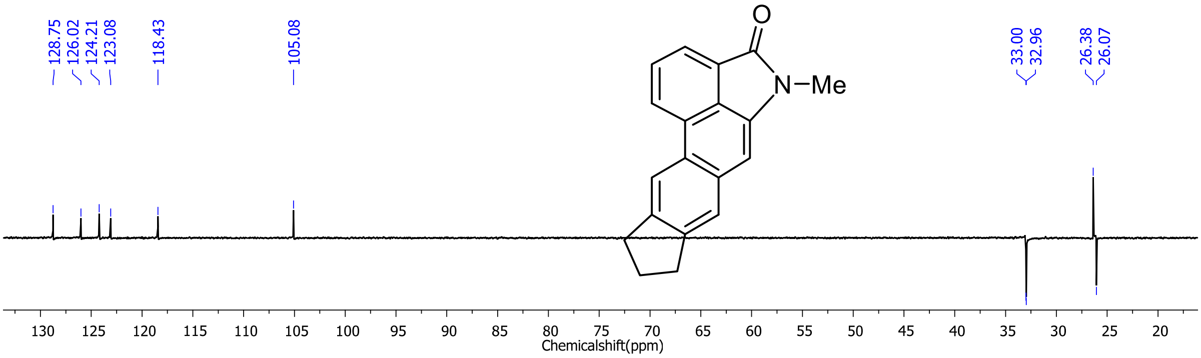

Spectral Data of Compound **9u.**

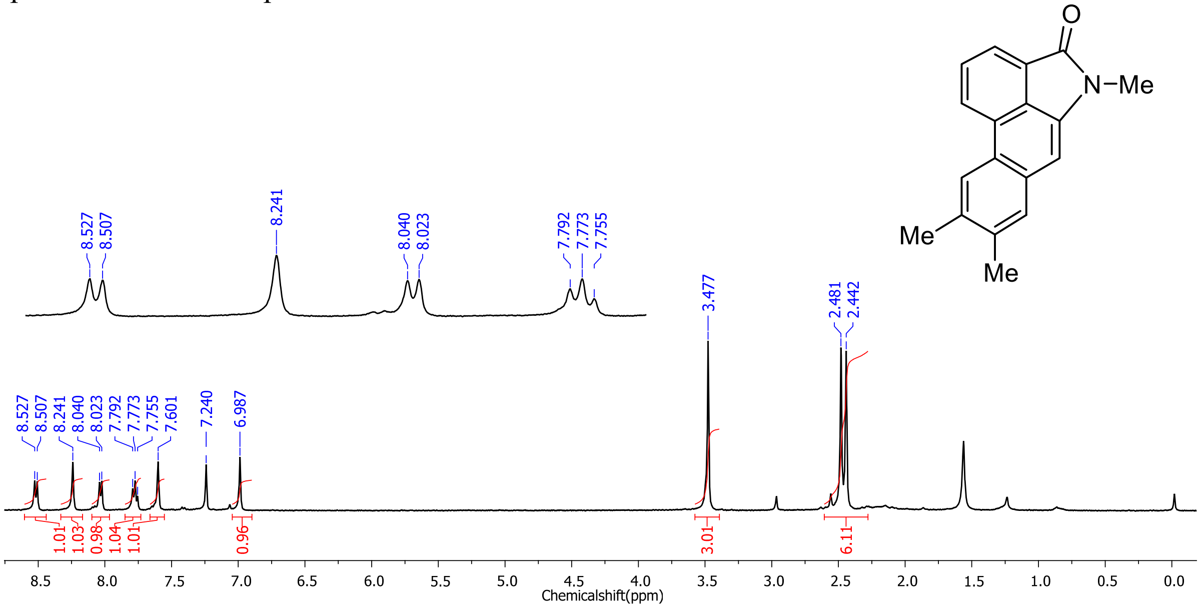

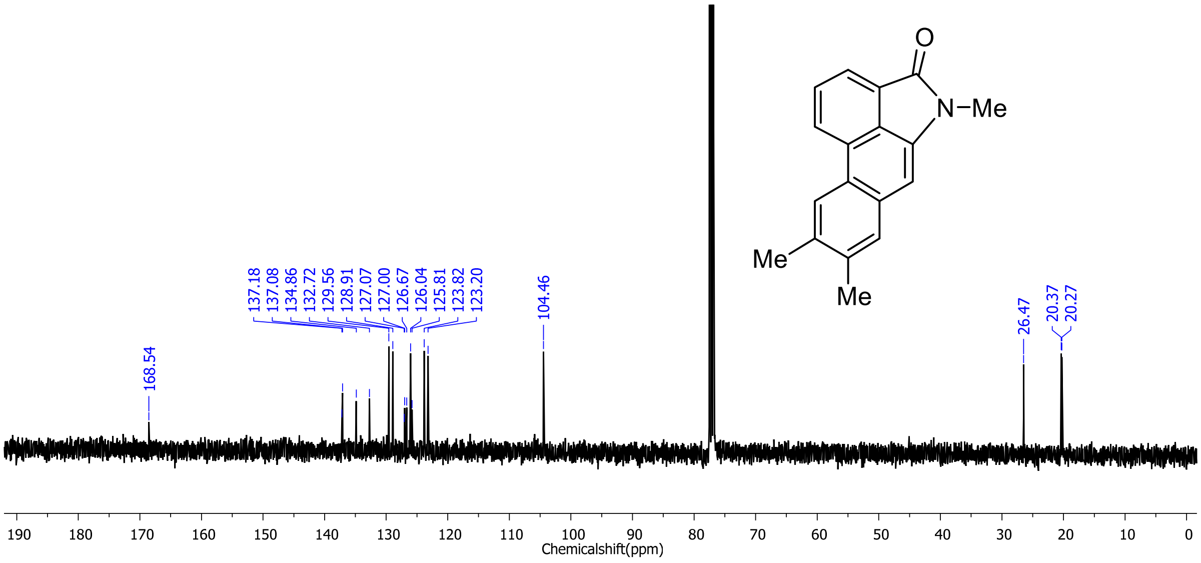

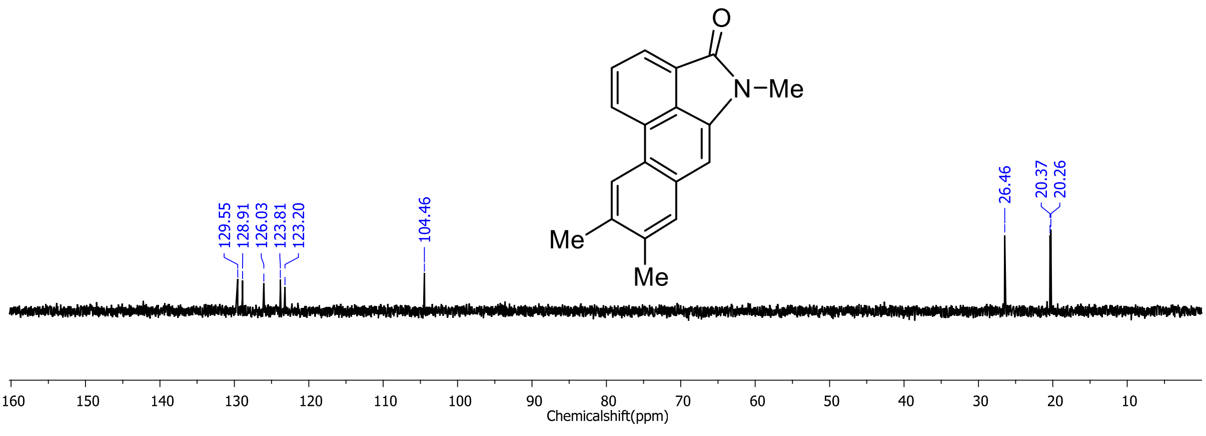

Spectral Data of Compound **9v and 9v'.**

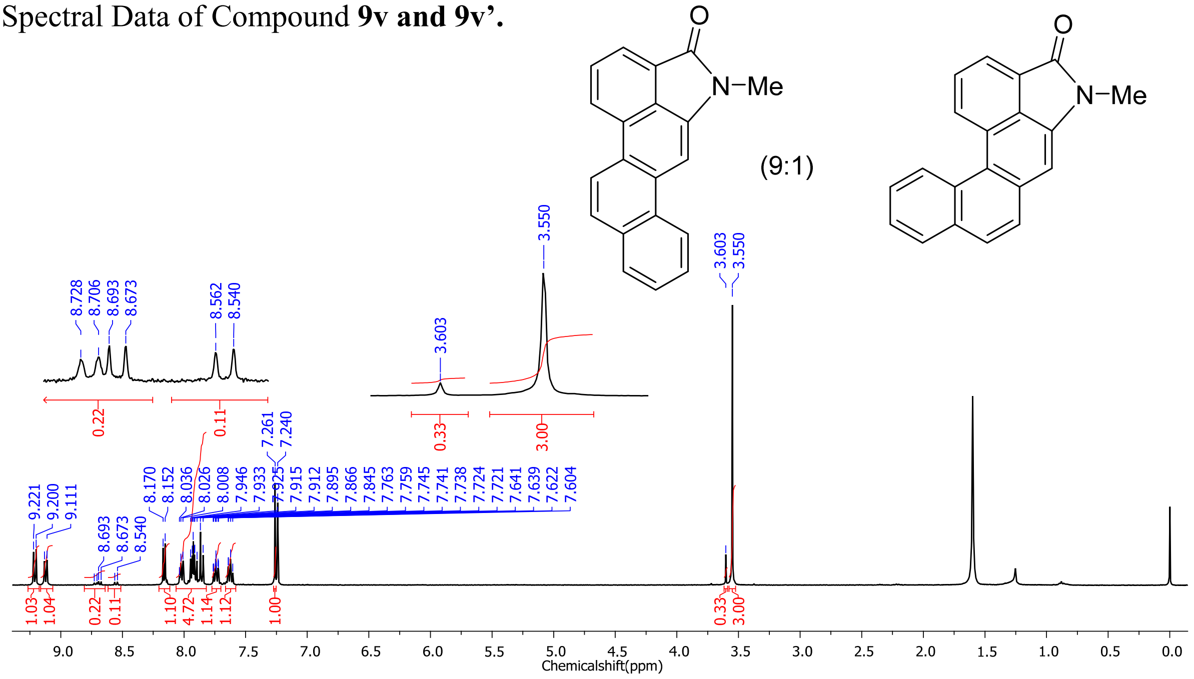

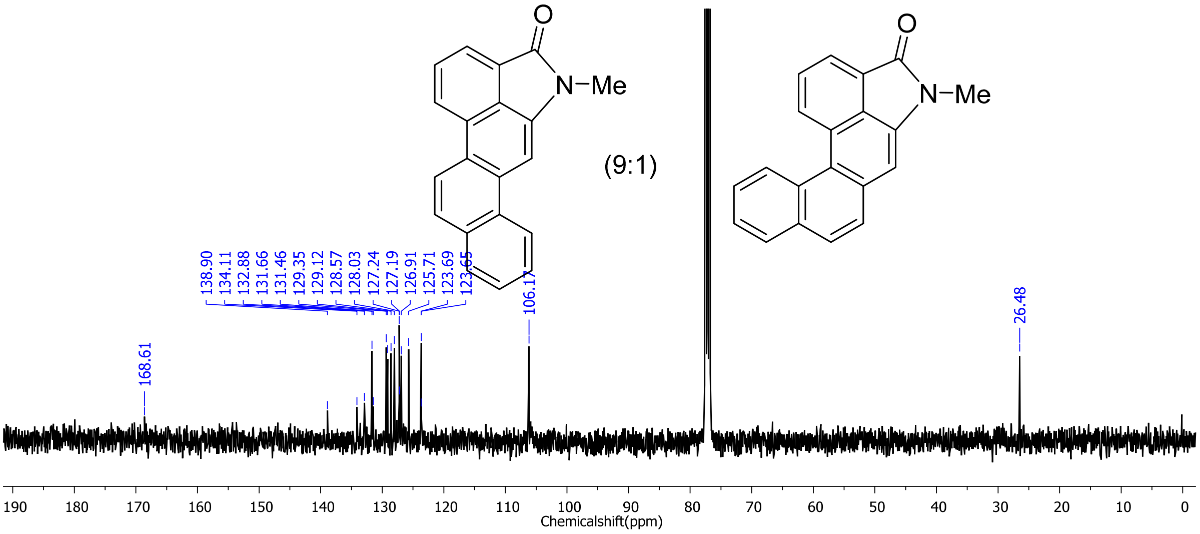

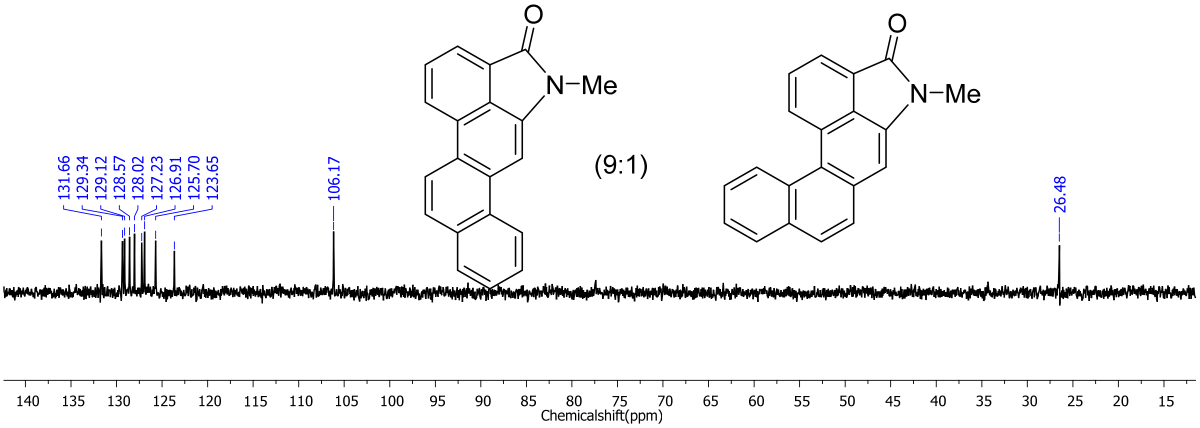

Spectral Data of Compound **9w.**

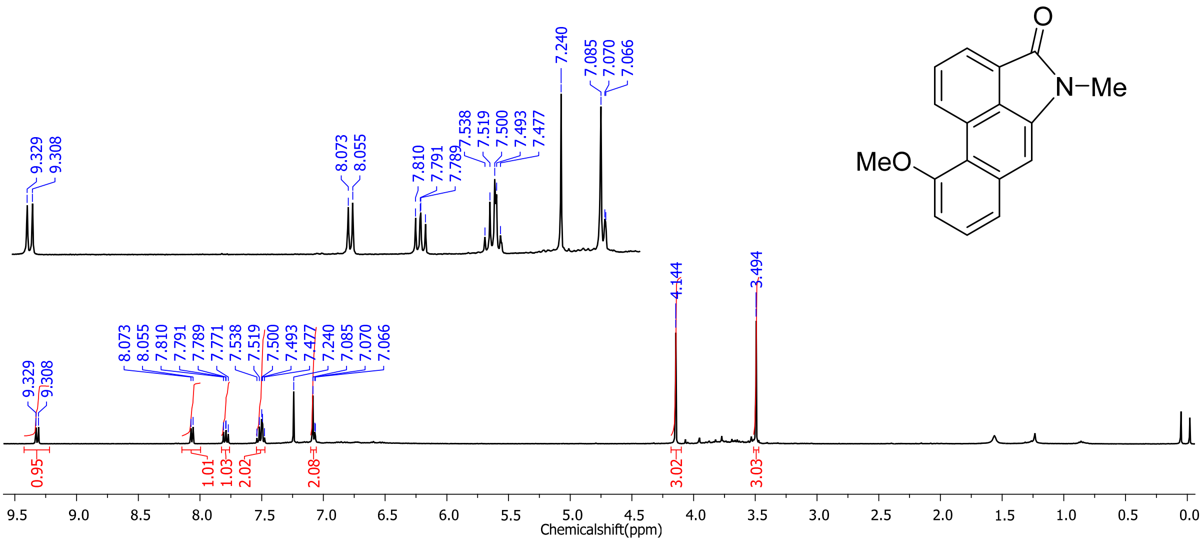

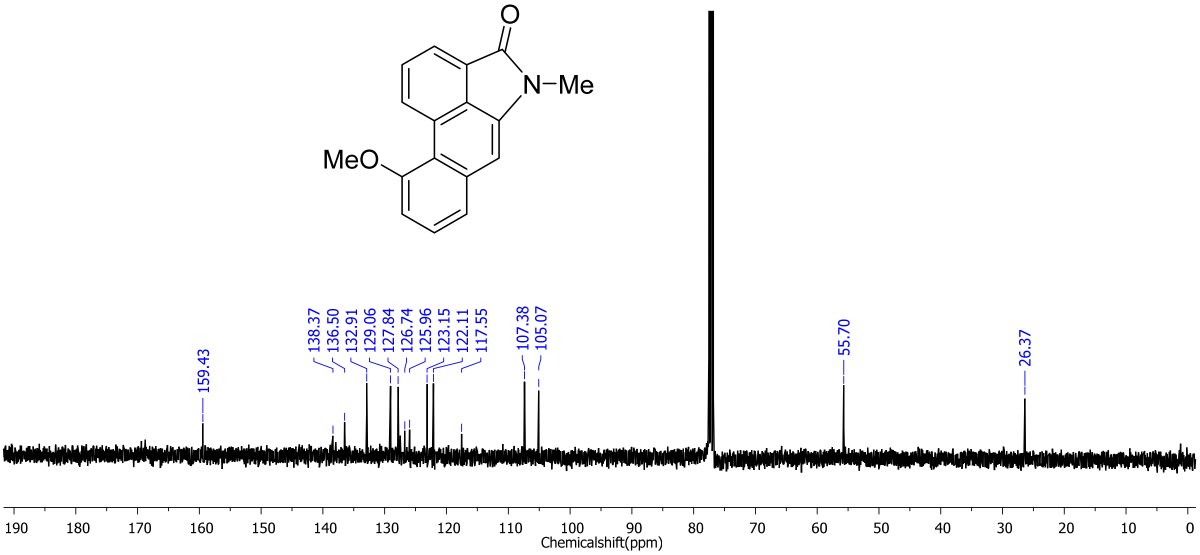

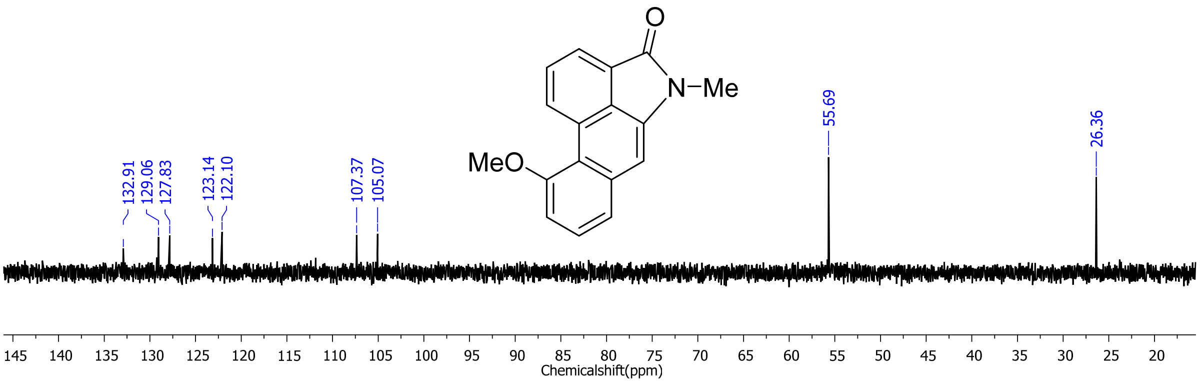

Spectral Data of Compound **10a.**

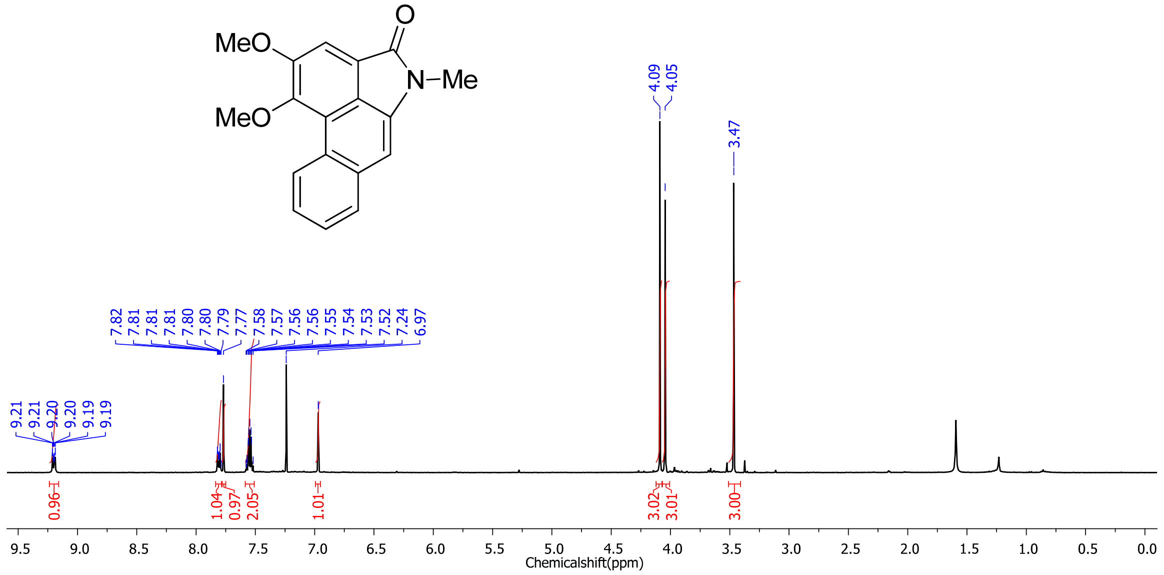

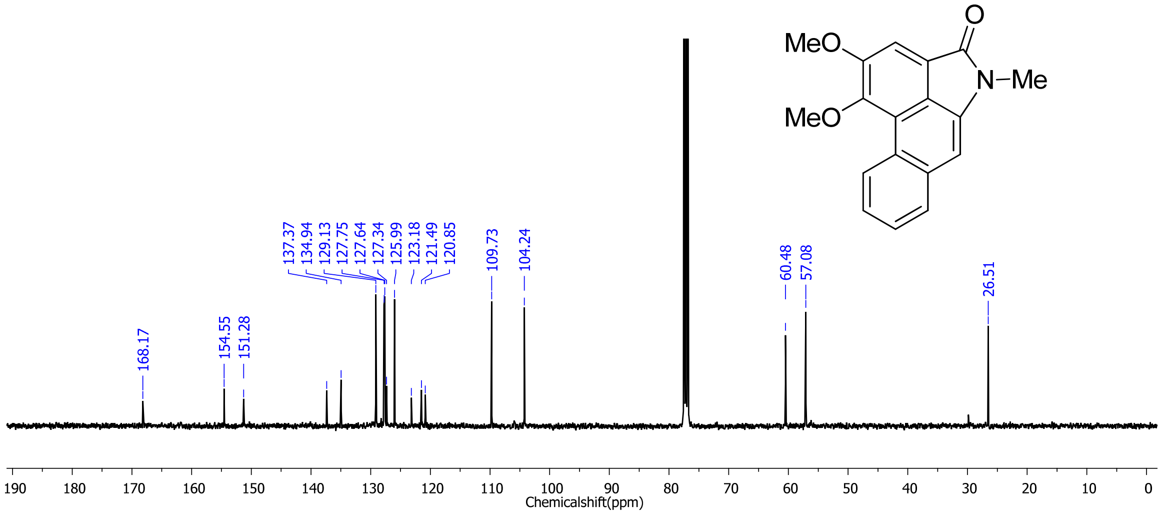

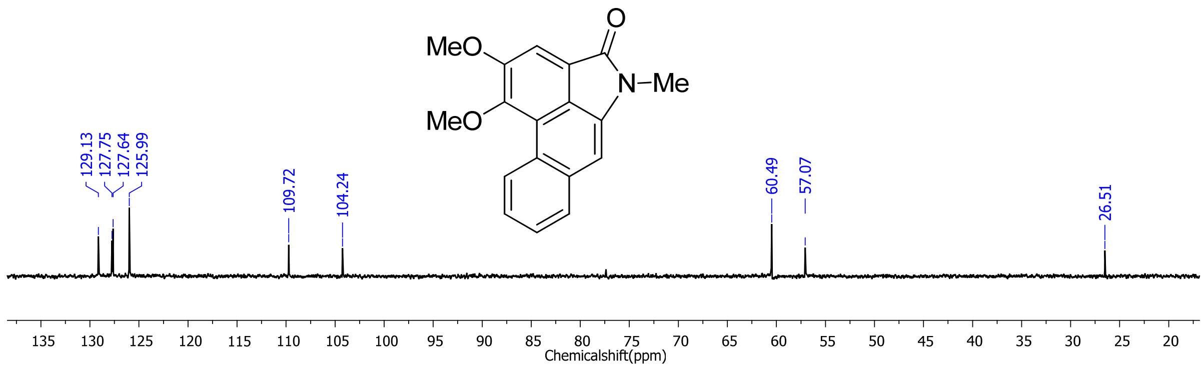

Spectral Data of Compound **10b.**

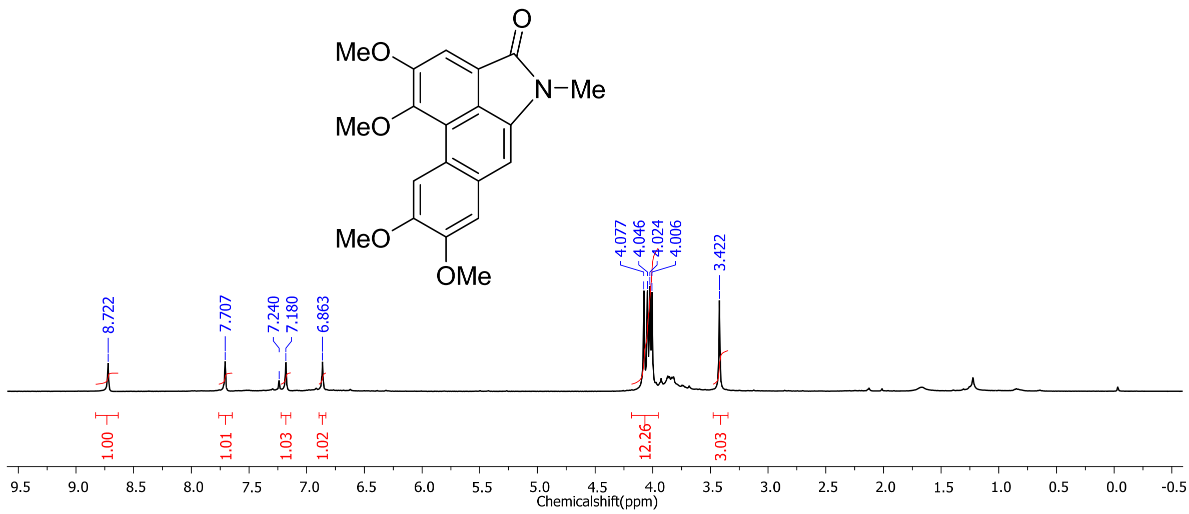

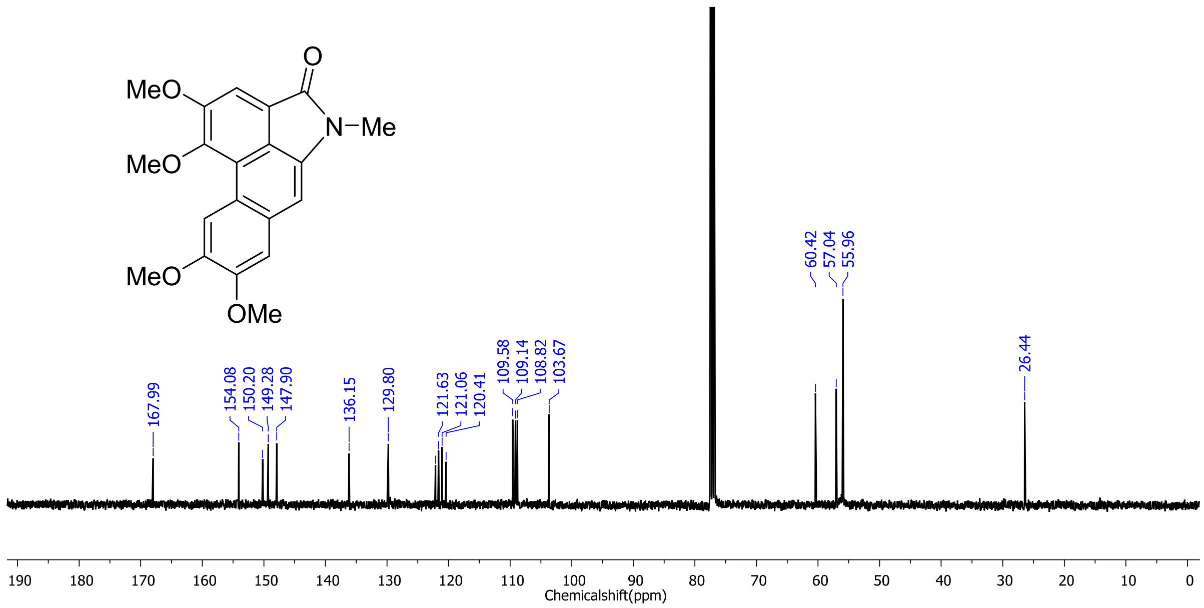

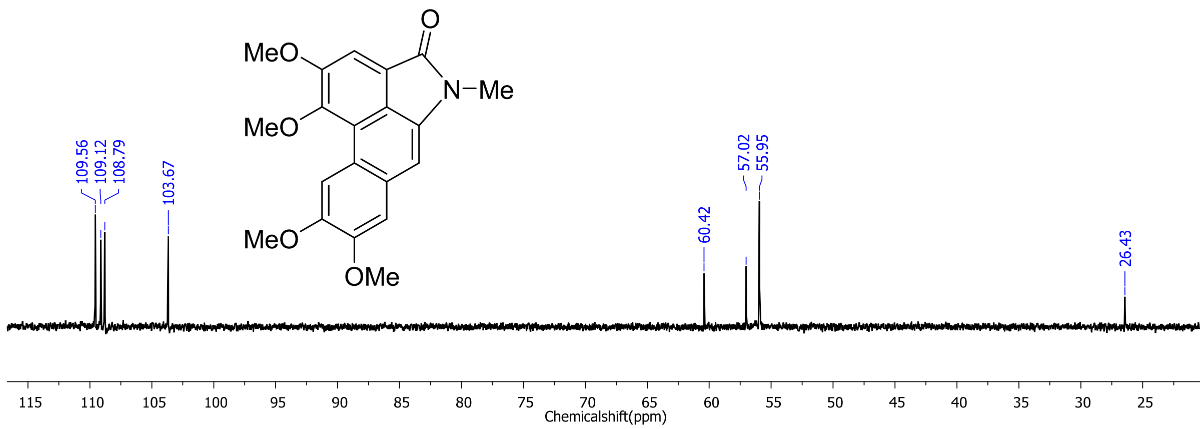

Spectral Data of Compound **10c.**

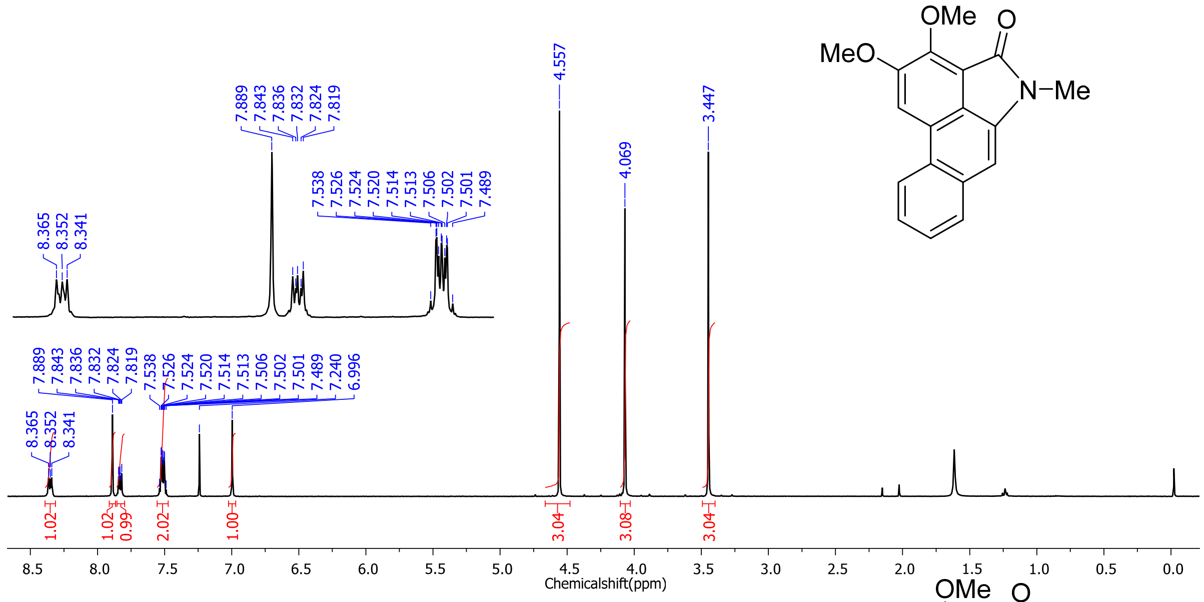

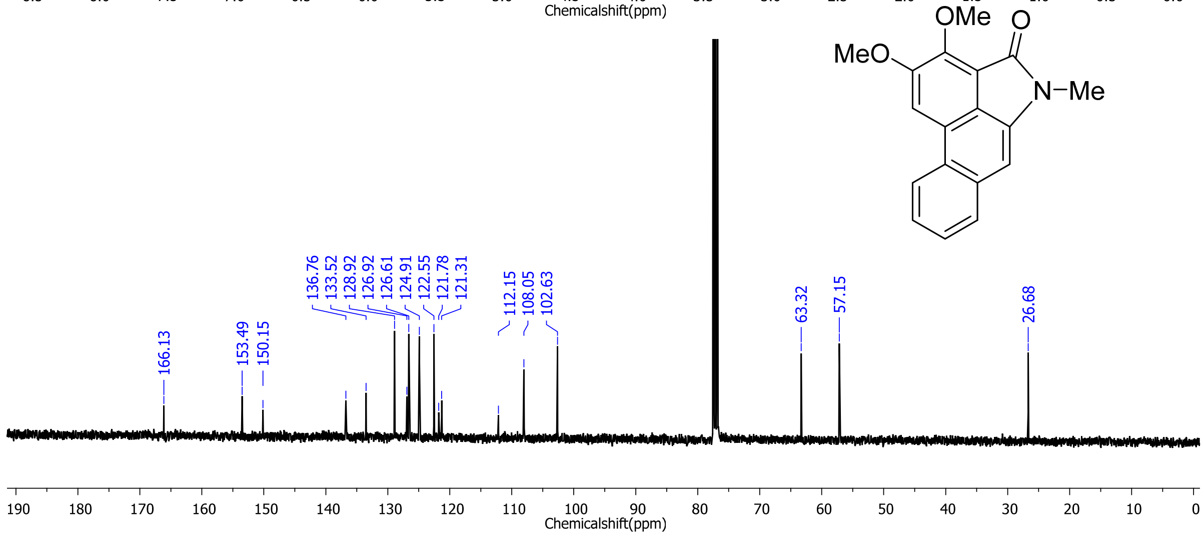

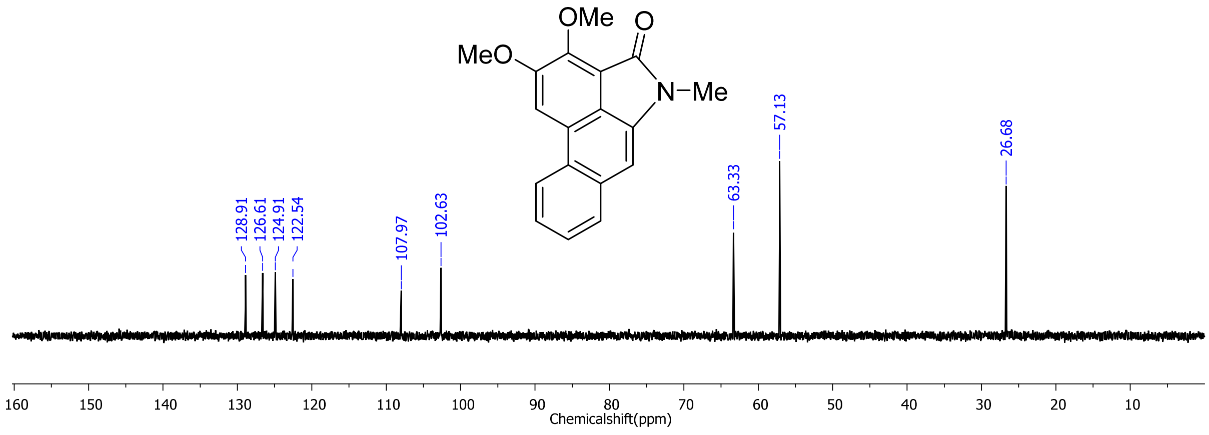

Spectral Data of Compound **10d.**

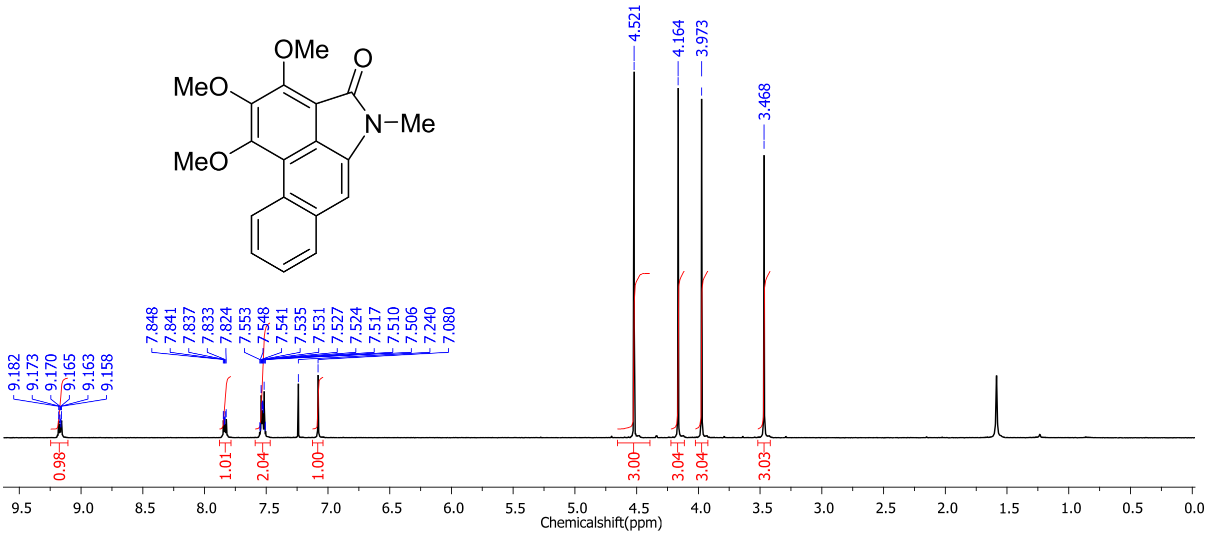

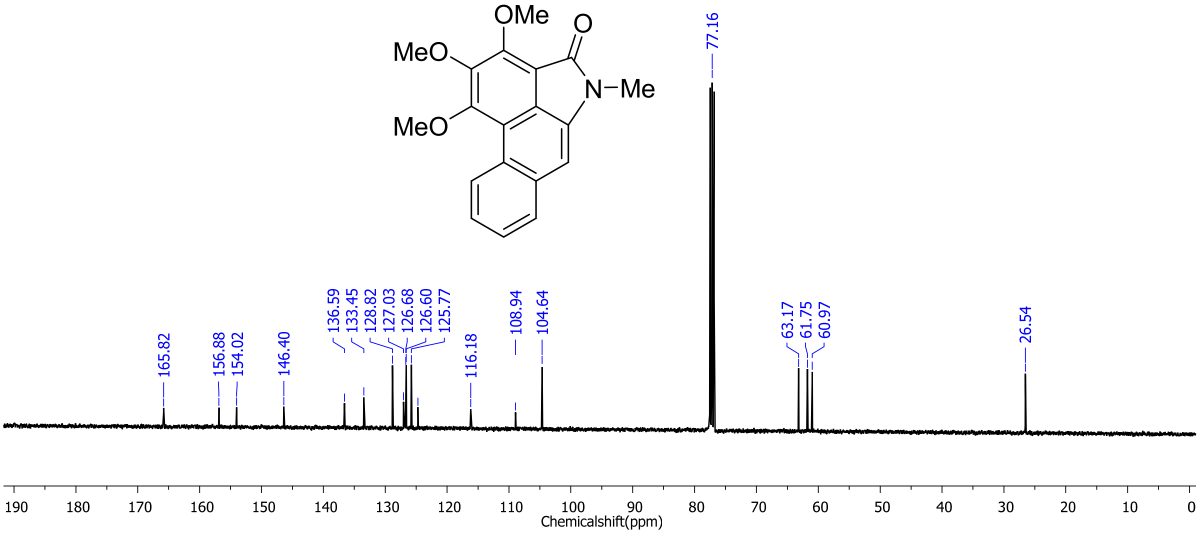

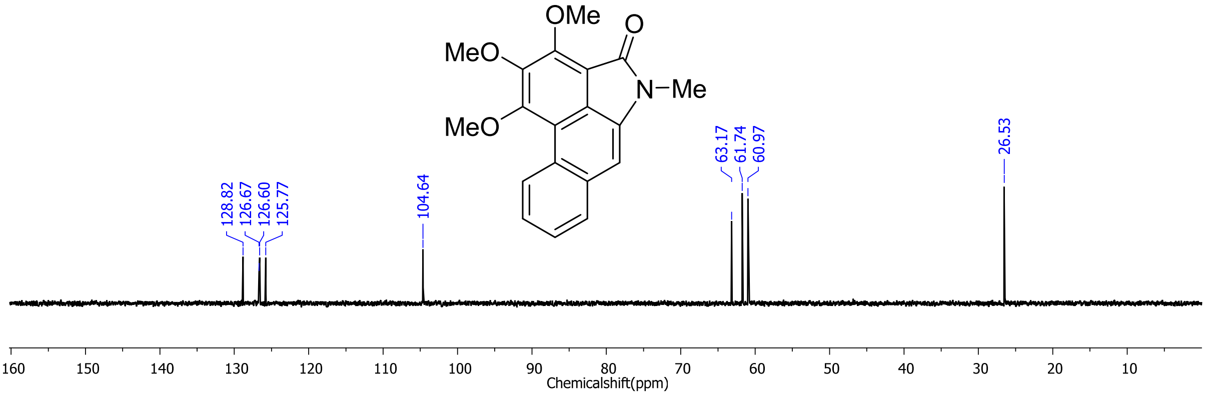

Spectral Data of Compound **11a.**

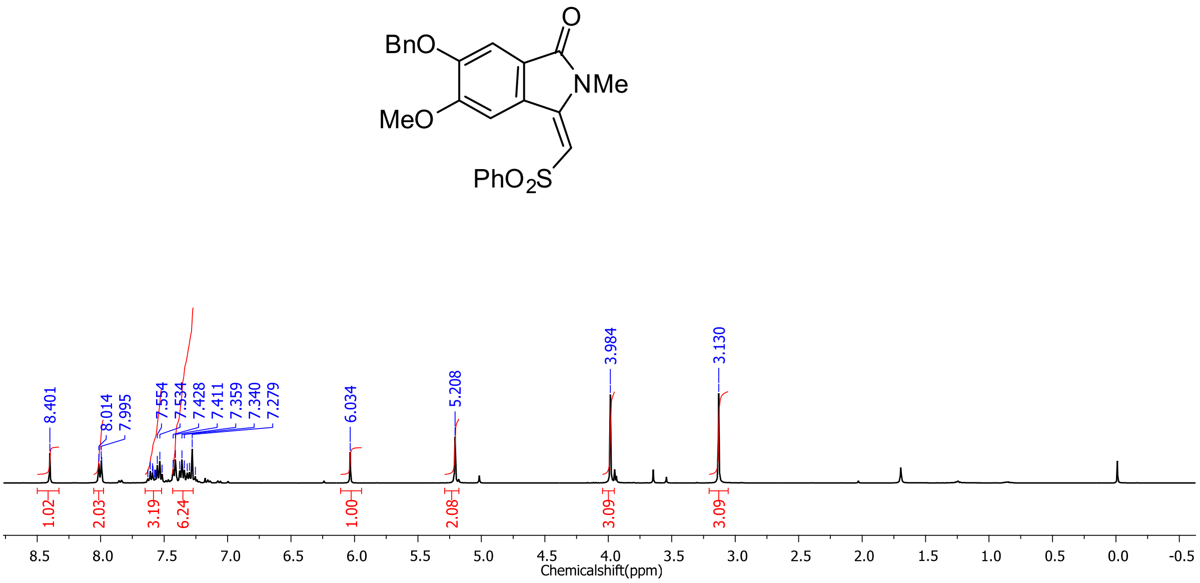

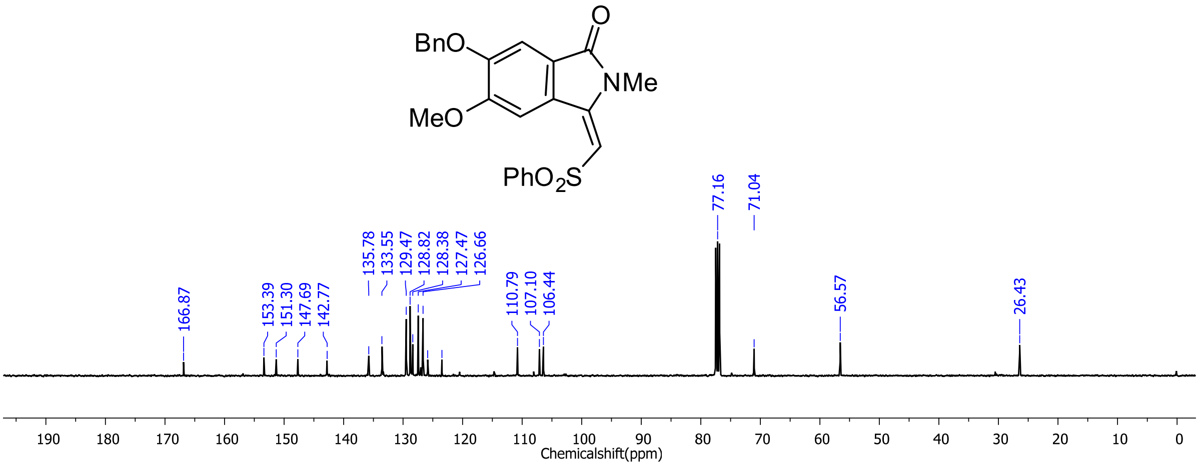

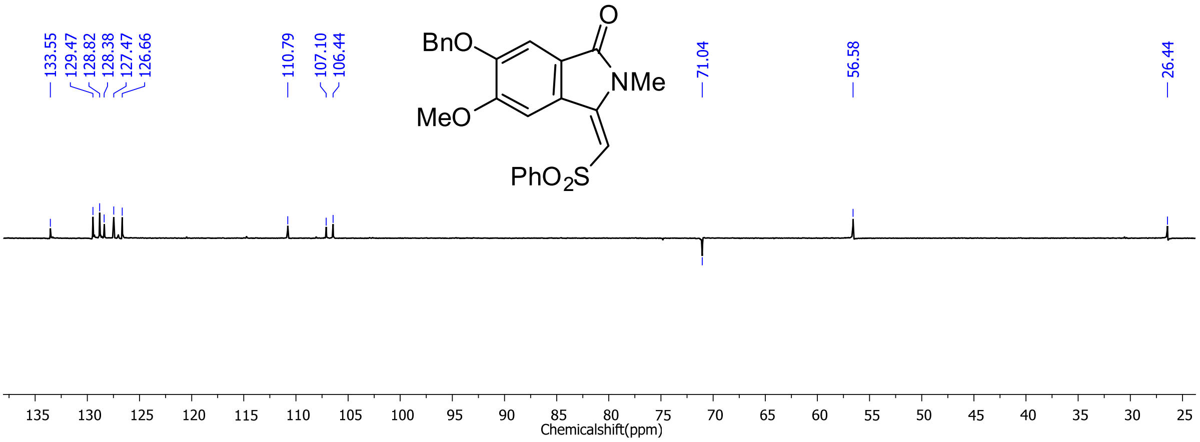

Spectral Data of Compound **12a.**

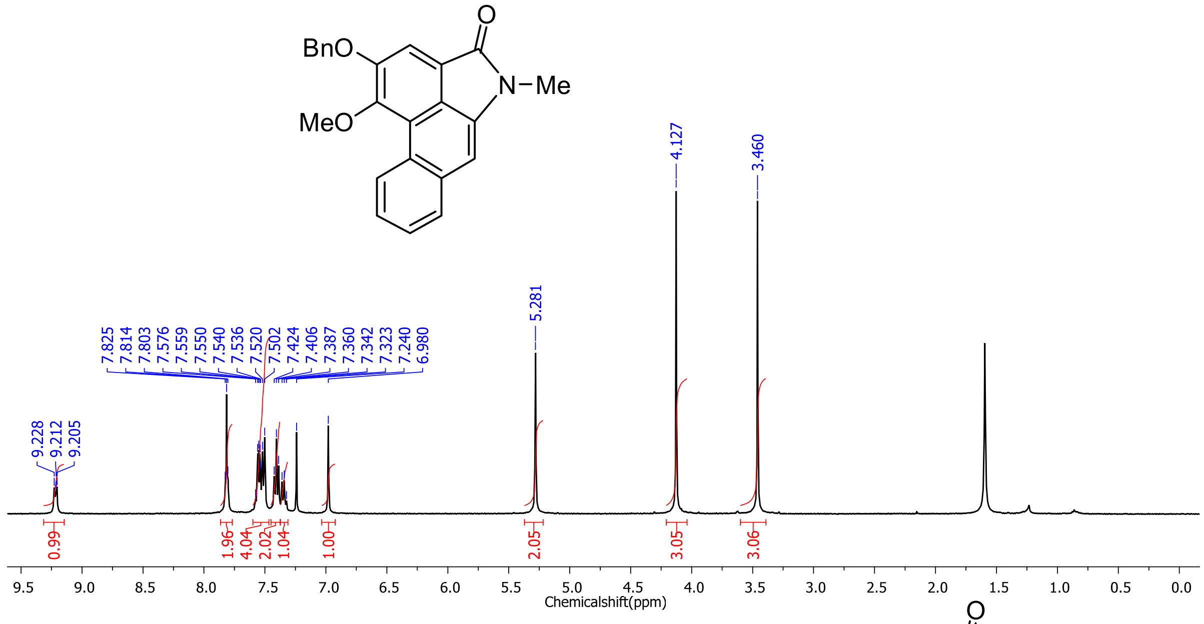

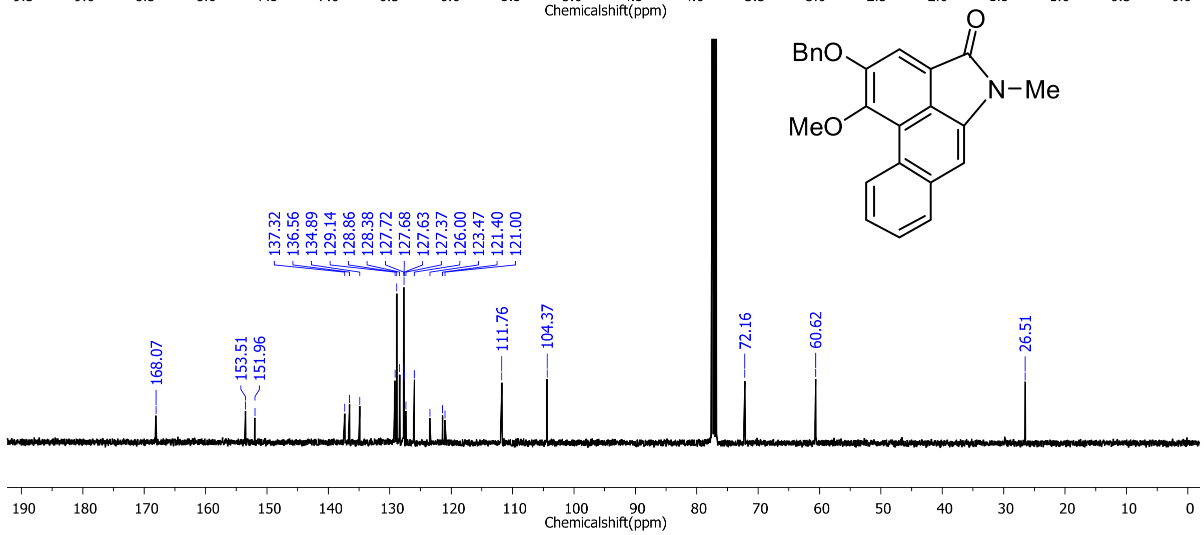

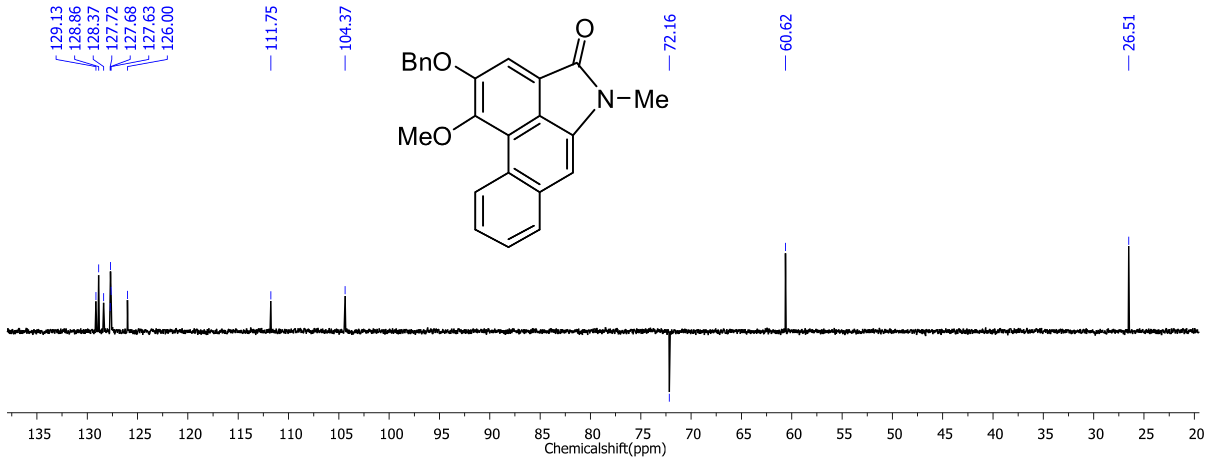

Spectral Data of Compound **10e.**

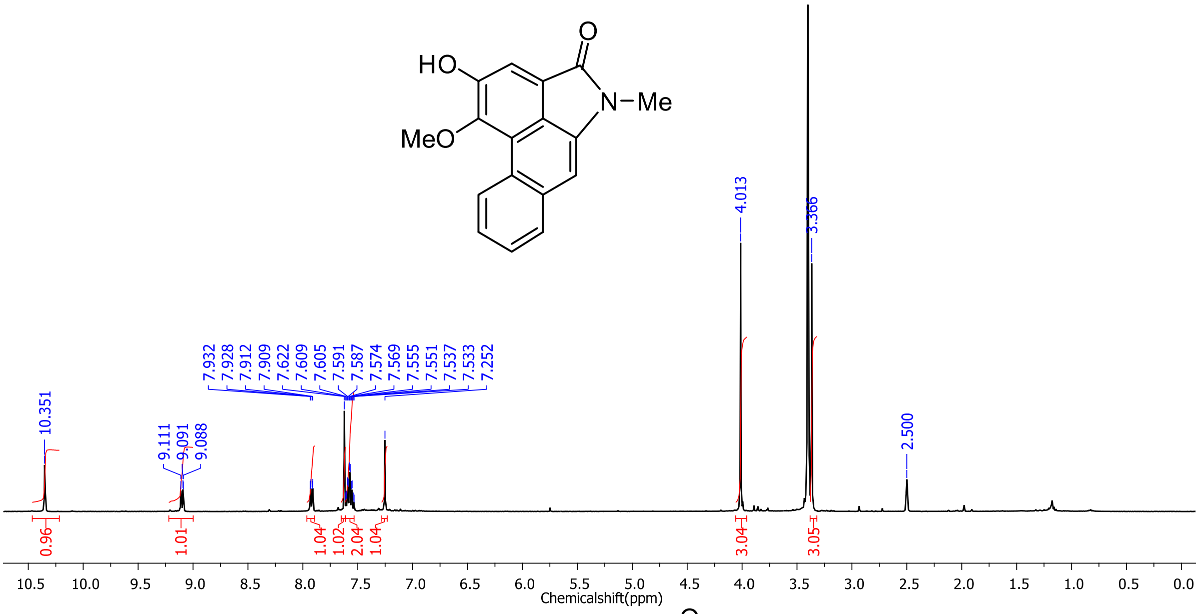

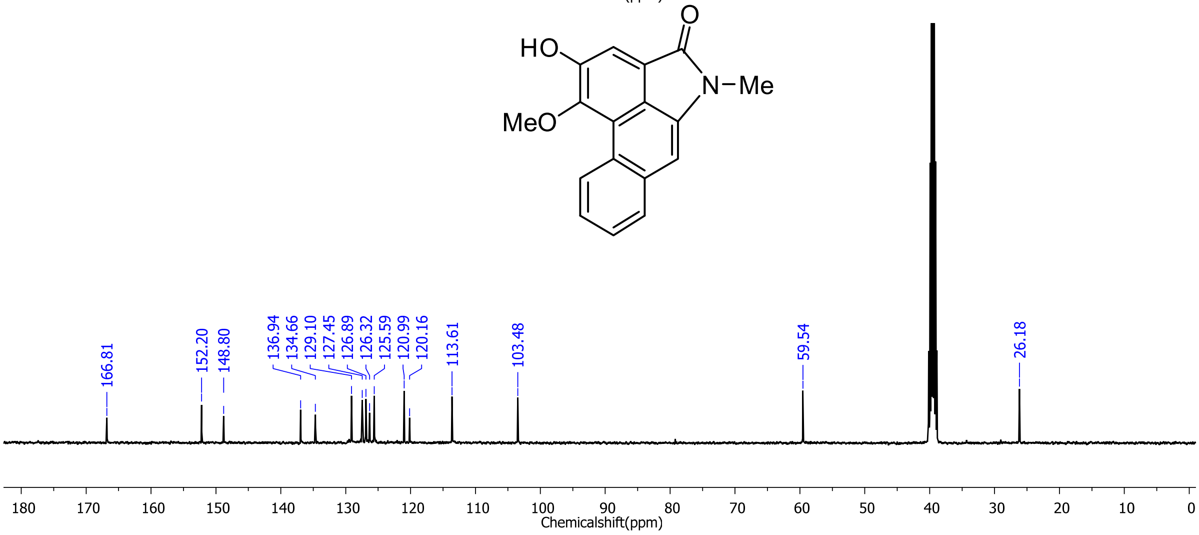

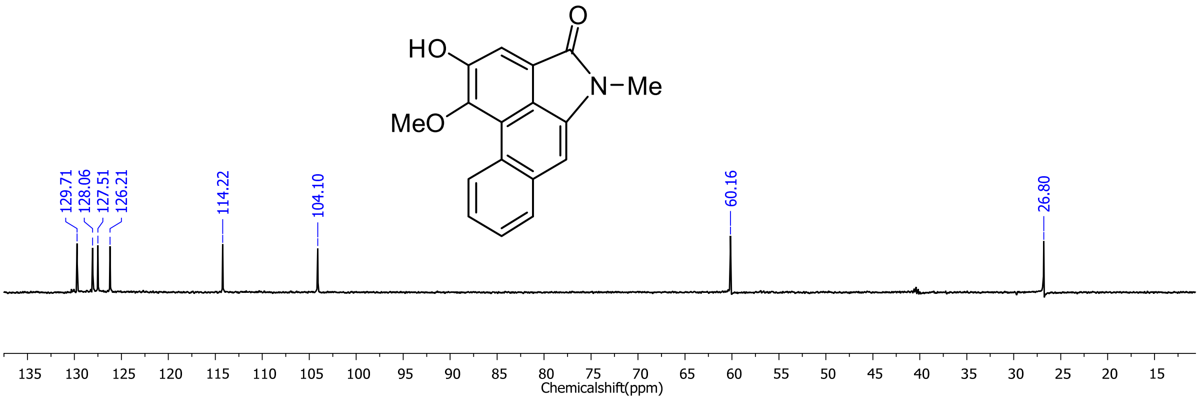

Spectral Data of Compound **11b.**

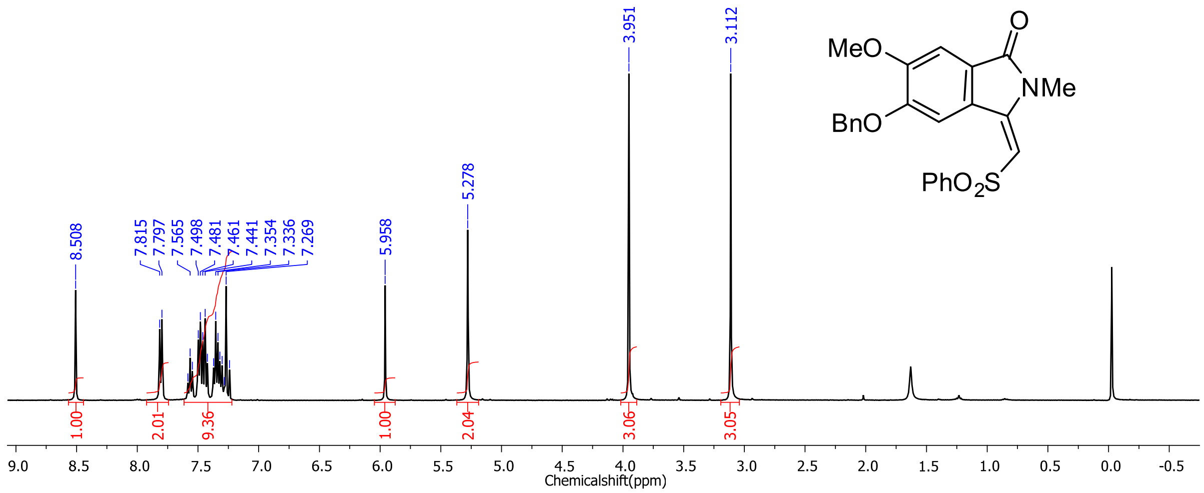

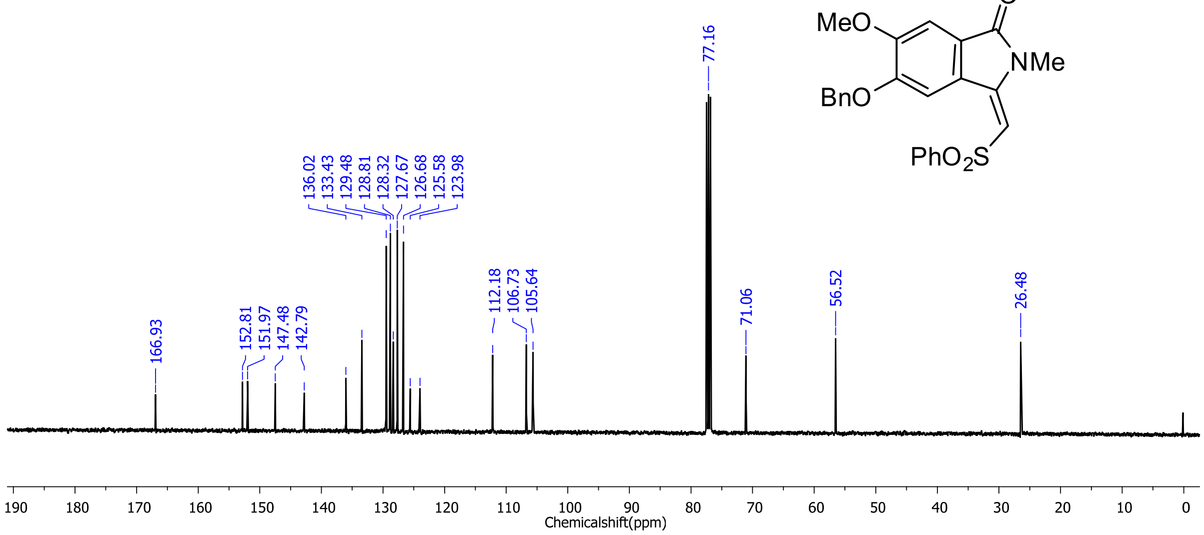

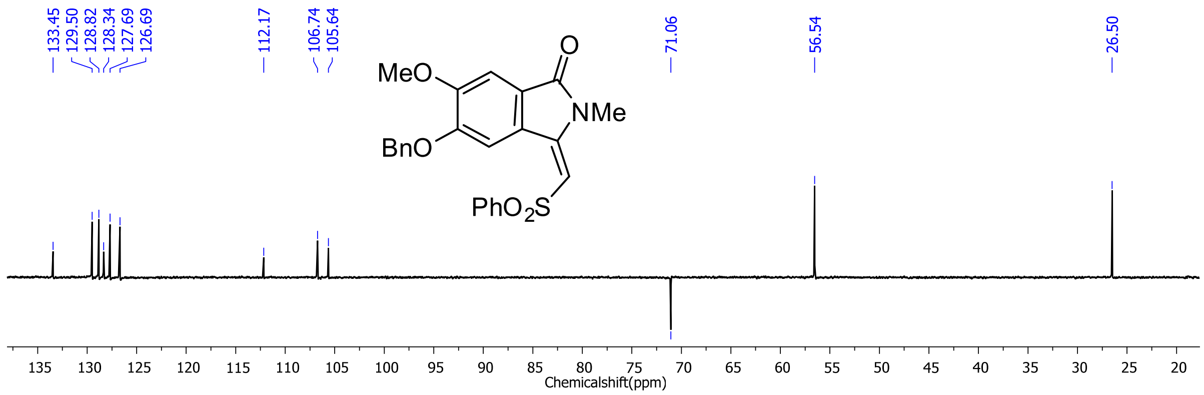

Spectral Data of Compound **12b.**

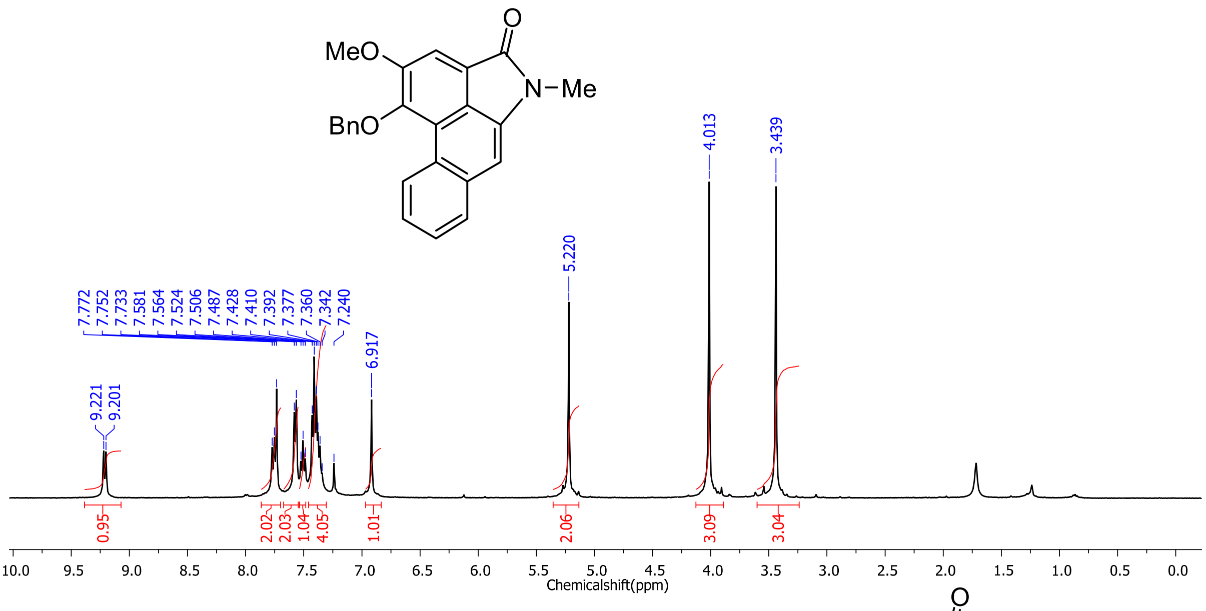

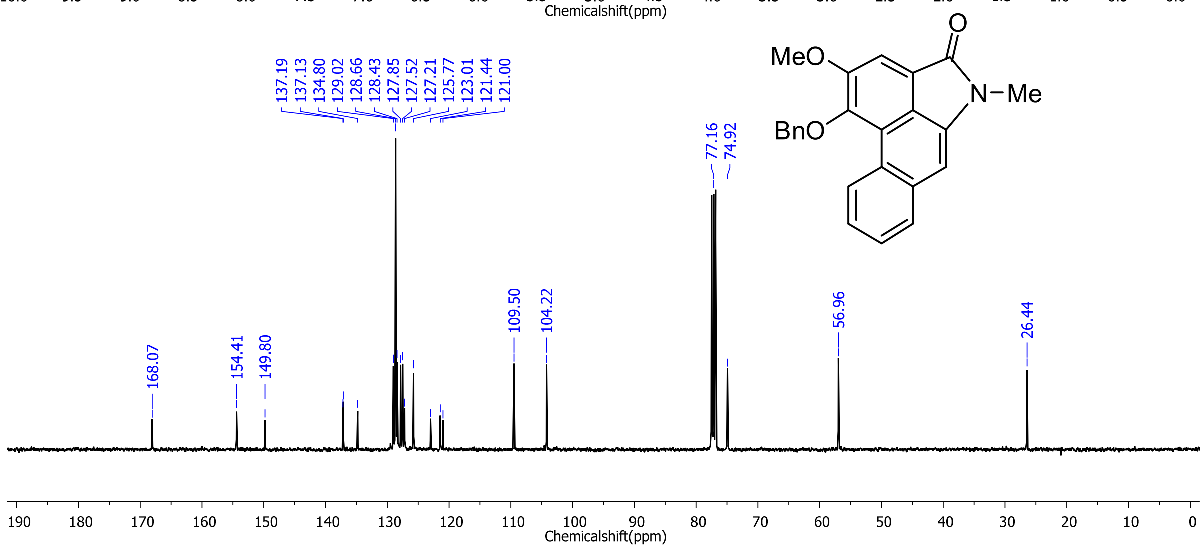

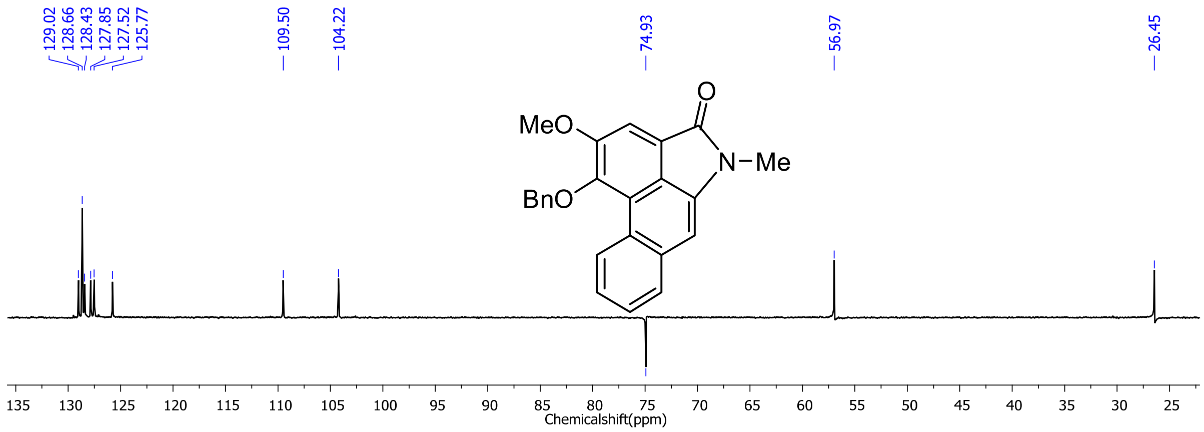

Spectral Data of Compound **10f.**

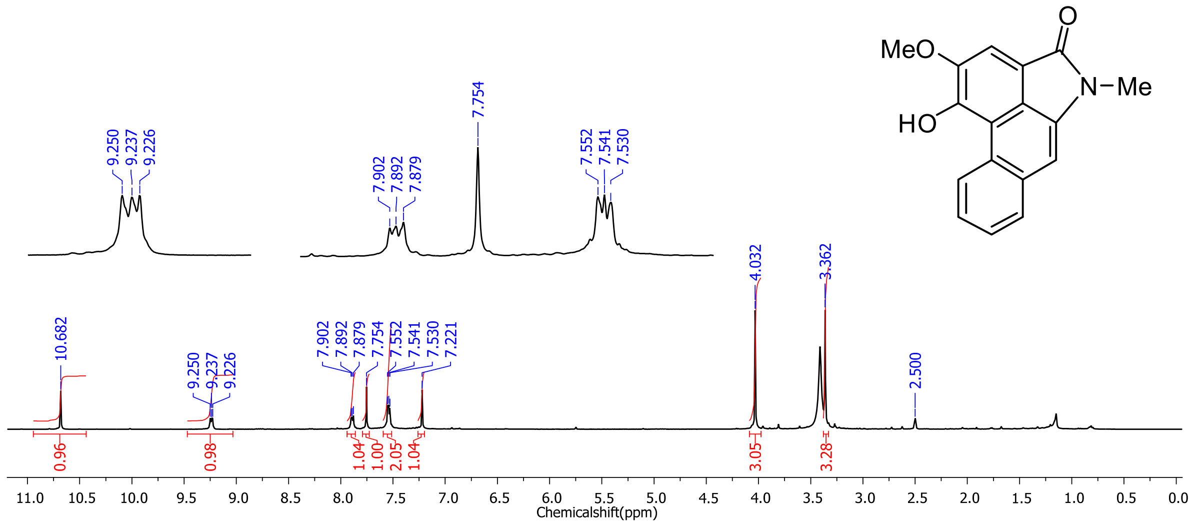

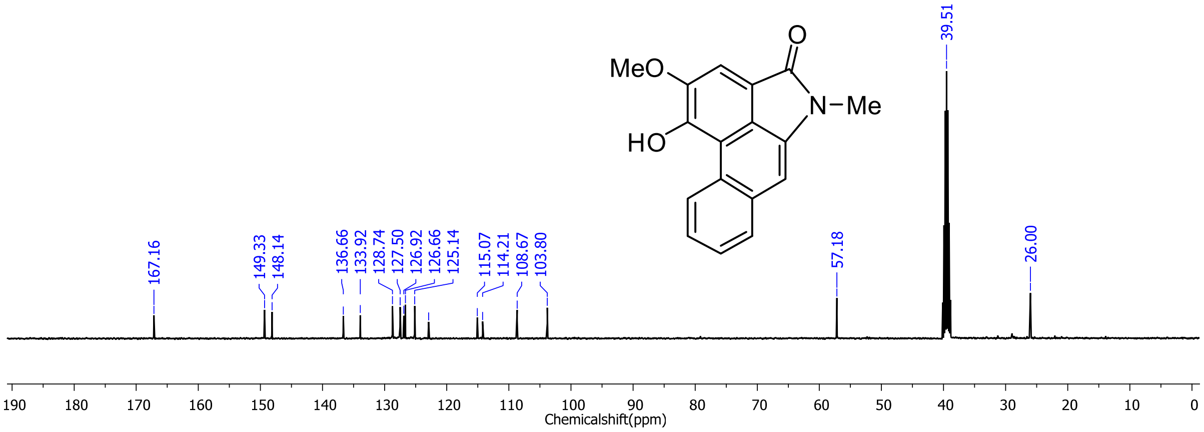

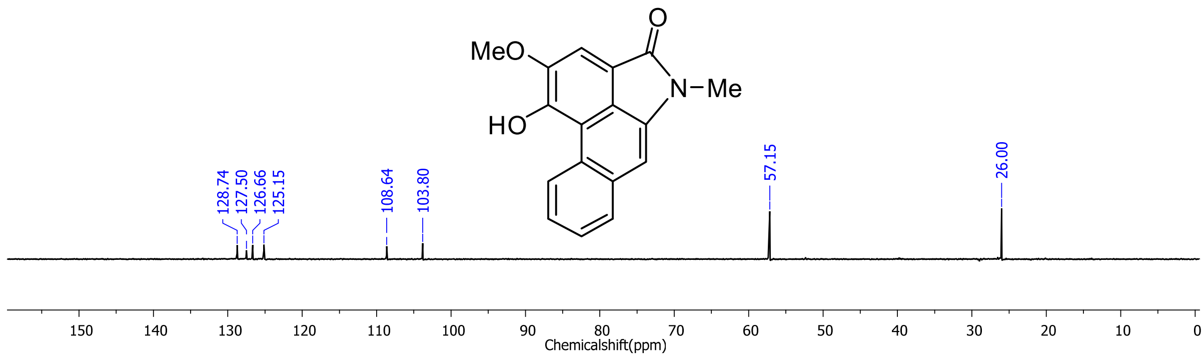

Spectral Data of Compound **11c.**

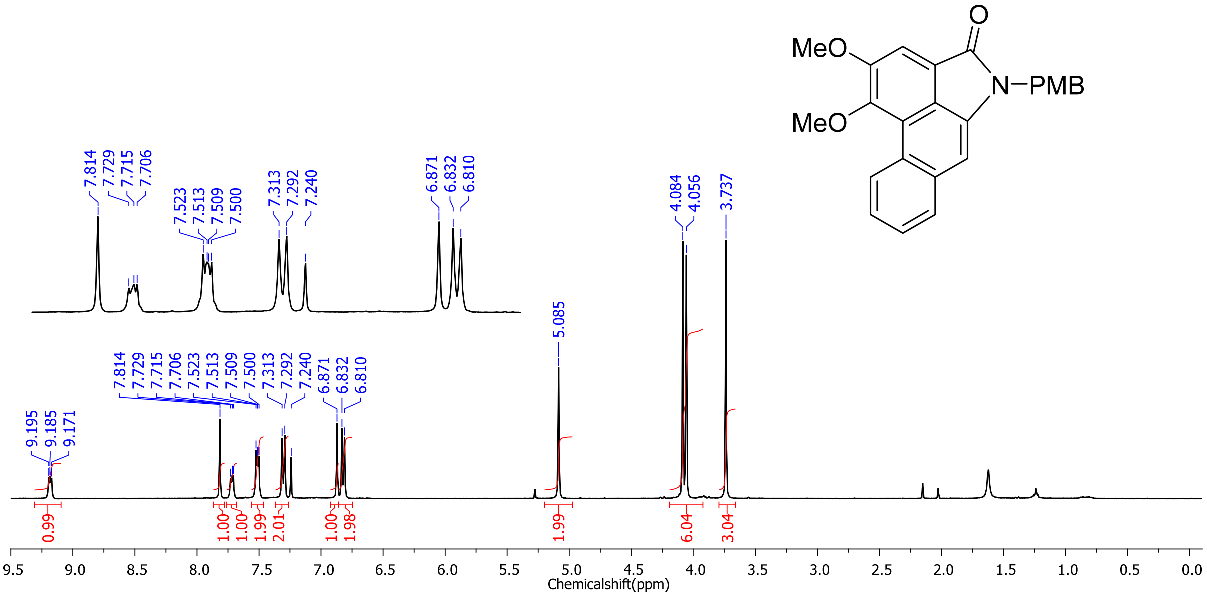

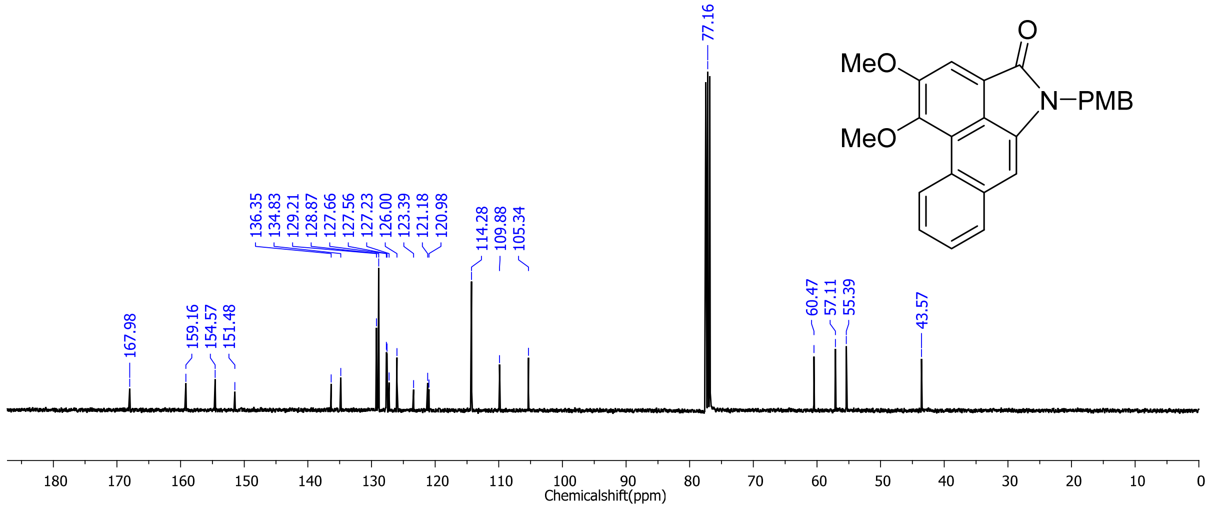

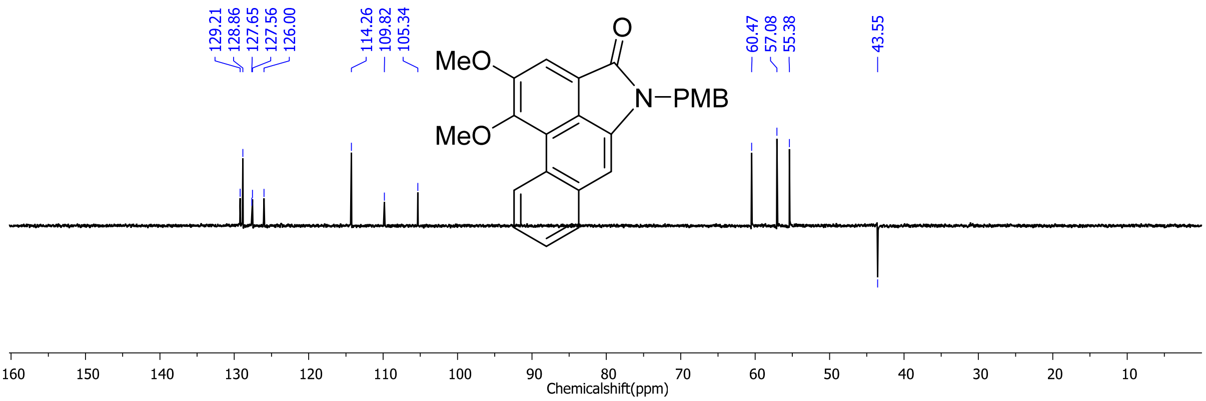

Spectral Data of Compound **10g.**

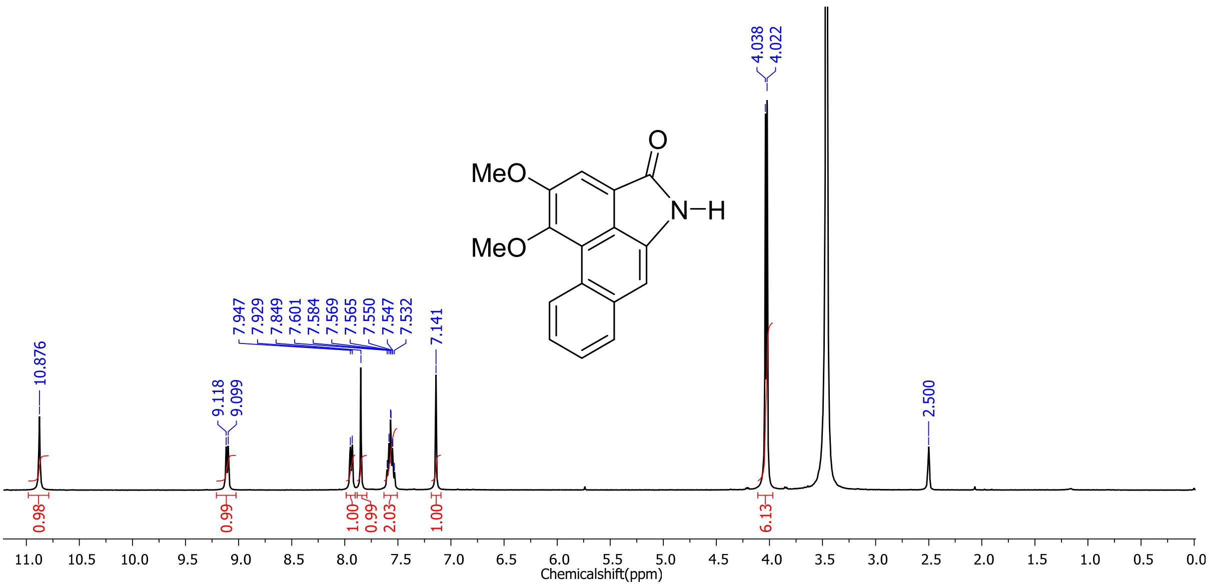

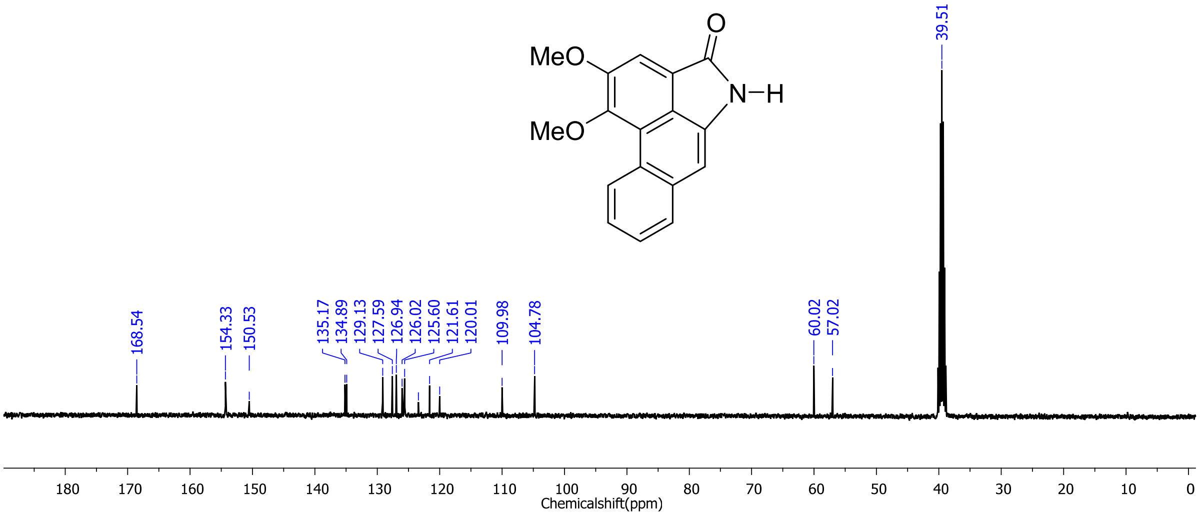

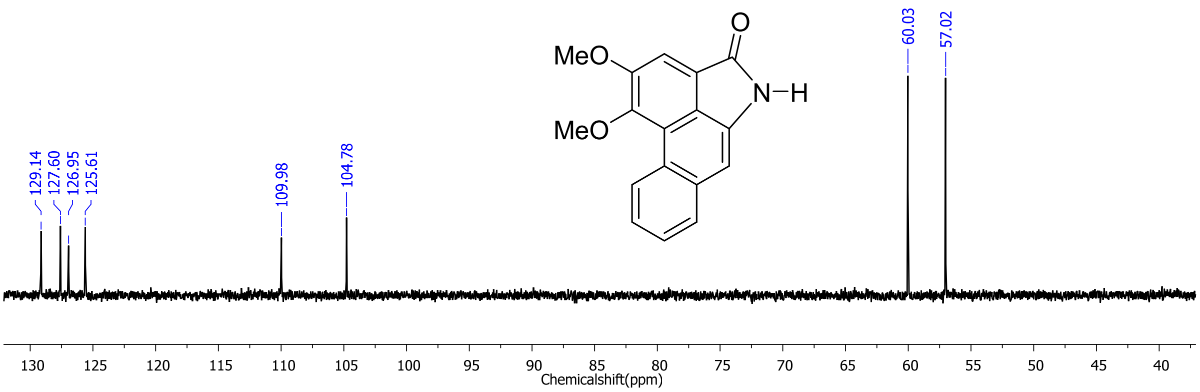

Spectral Data of Compound **11d.**

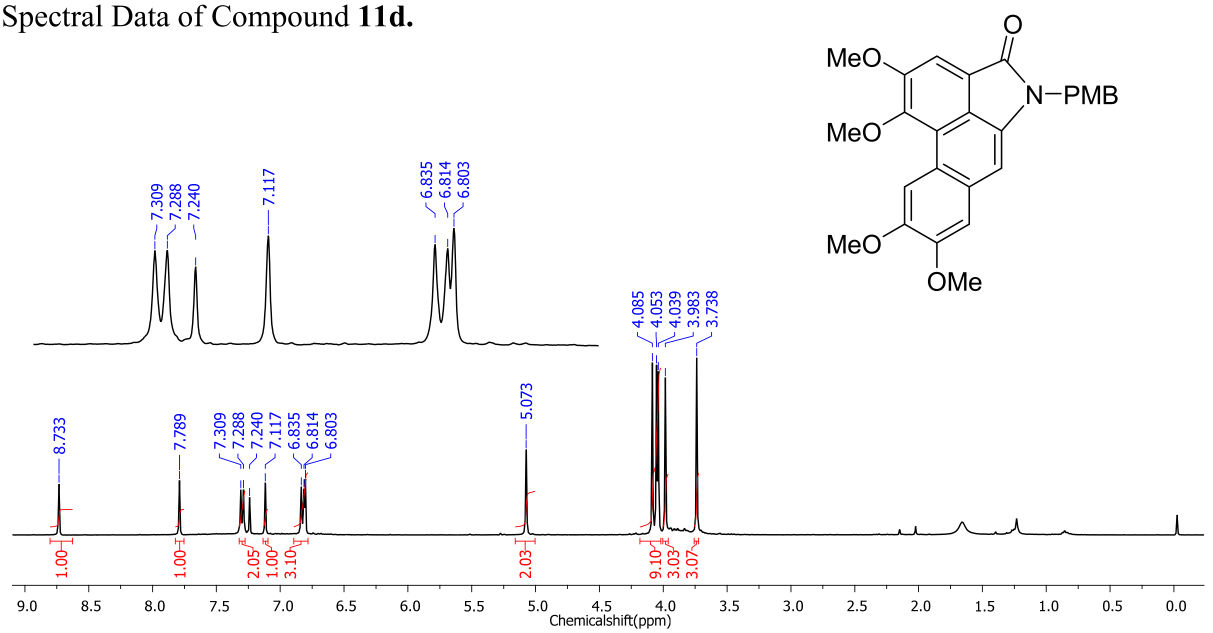

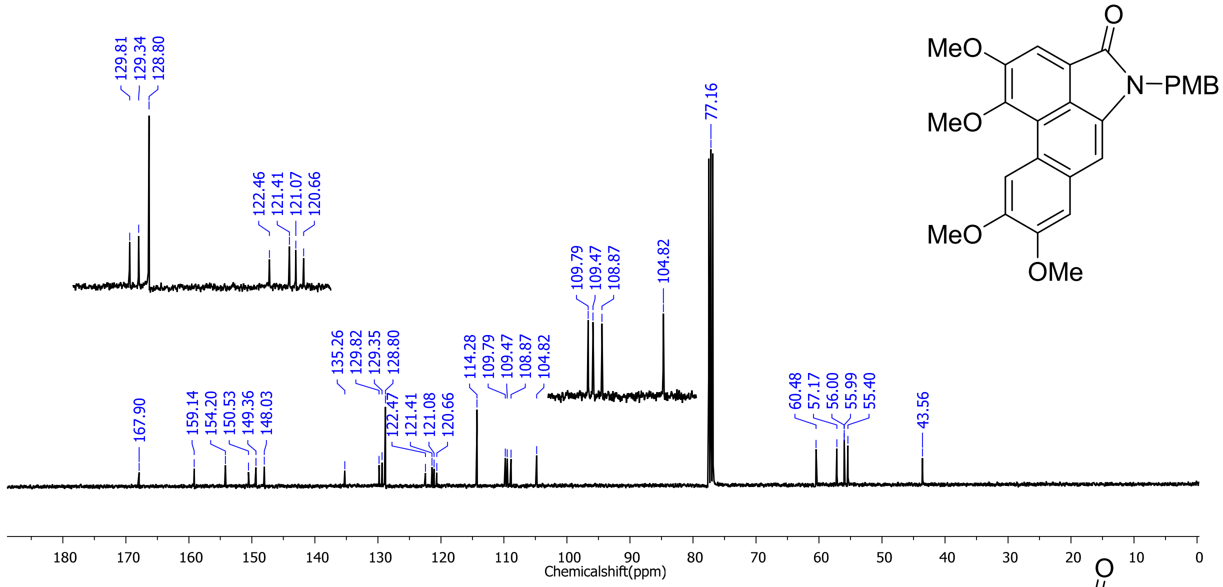

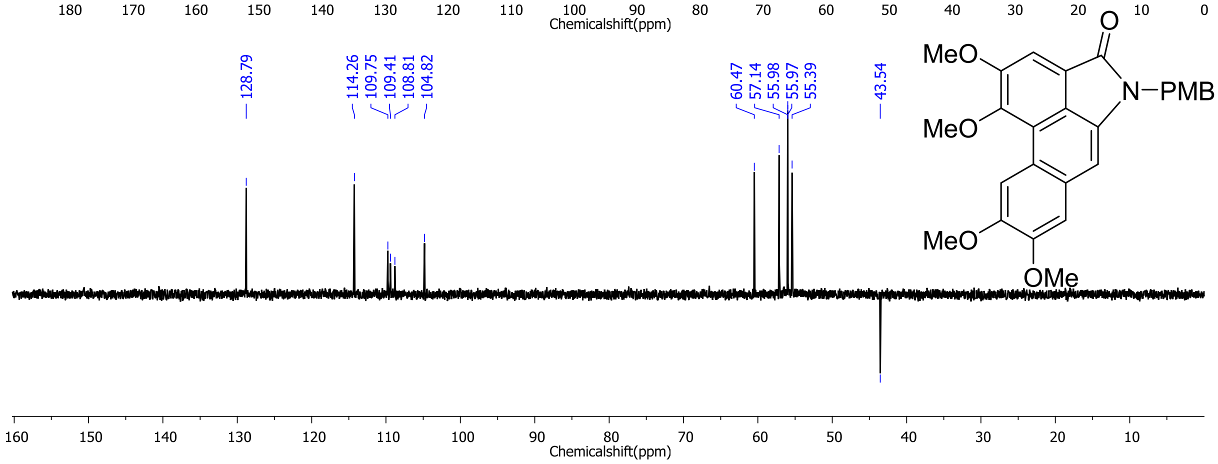

Spectral Data of Compound **10h.**

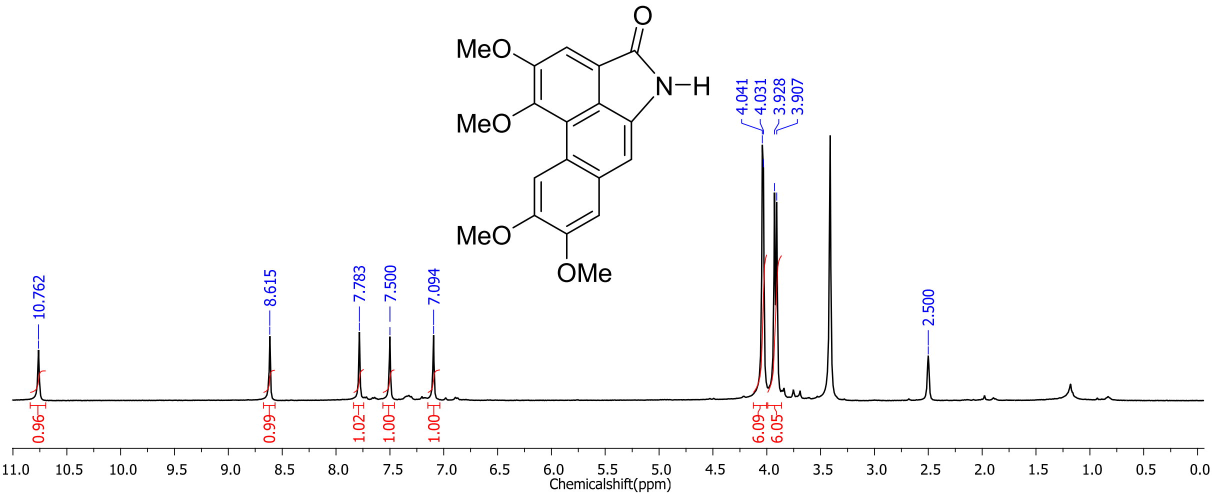

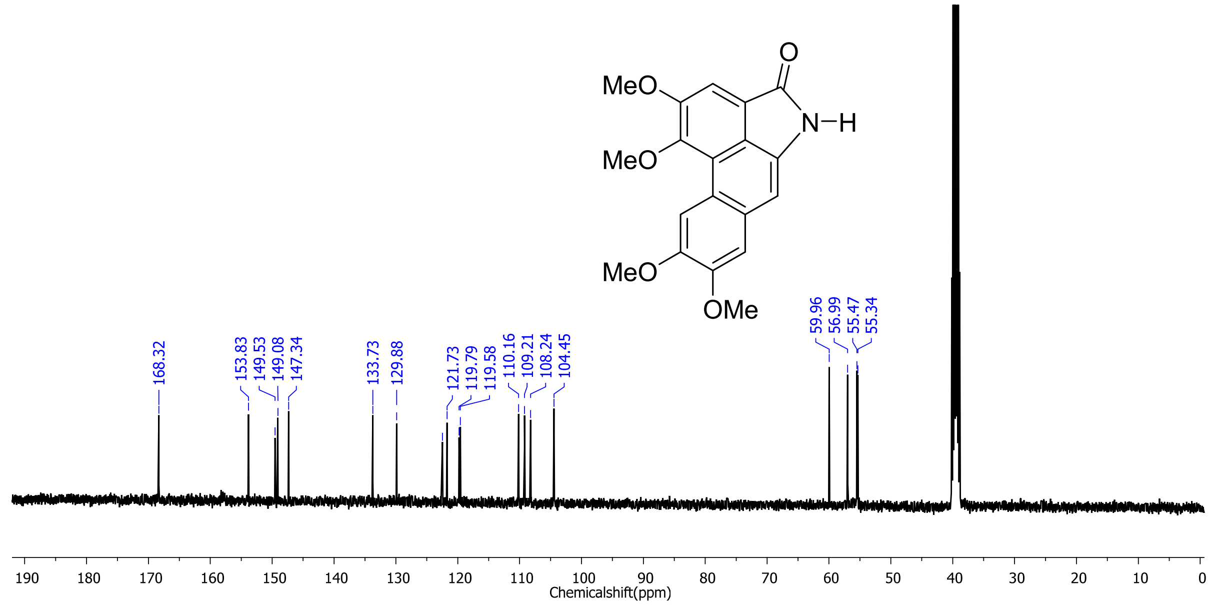

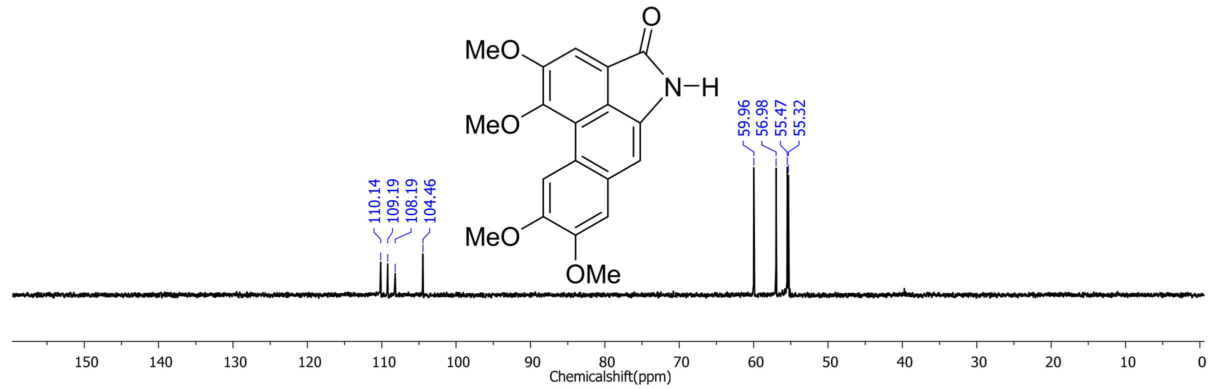

Spectral Data of Compound **11e.**

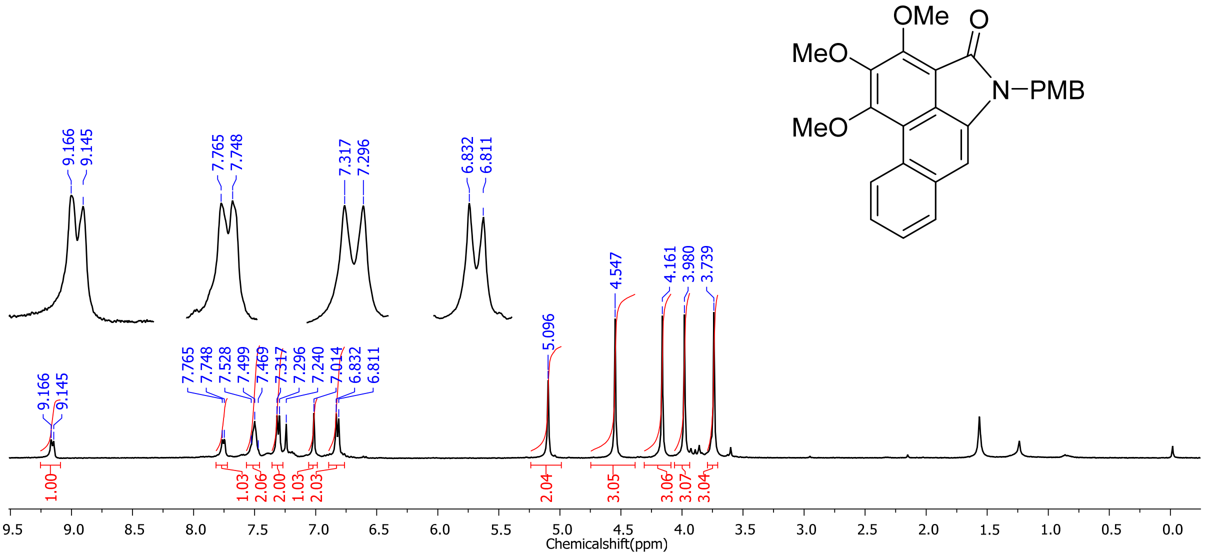

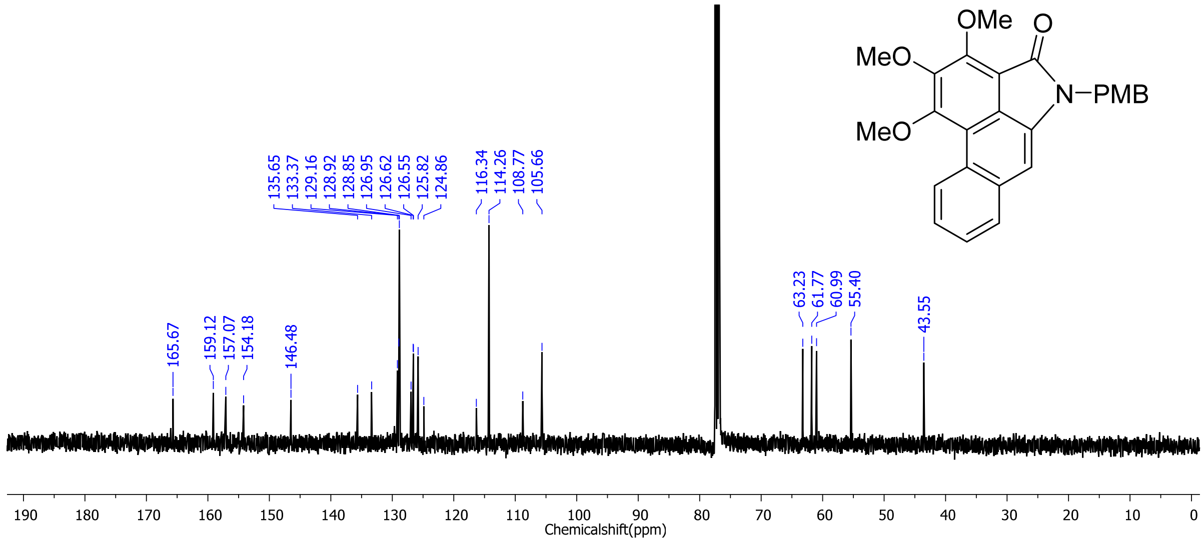

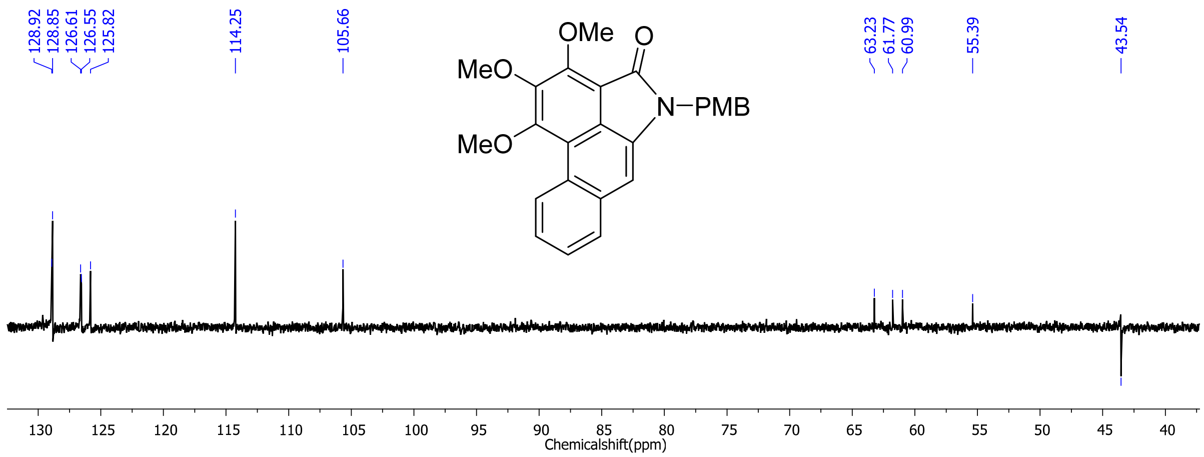

Spectral Data of Compound **10i.**

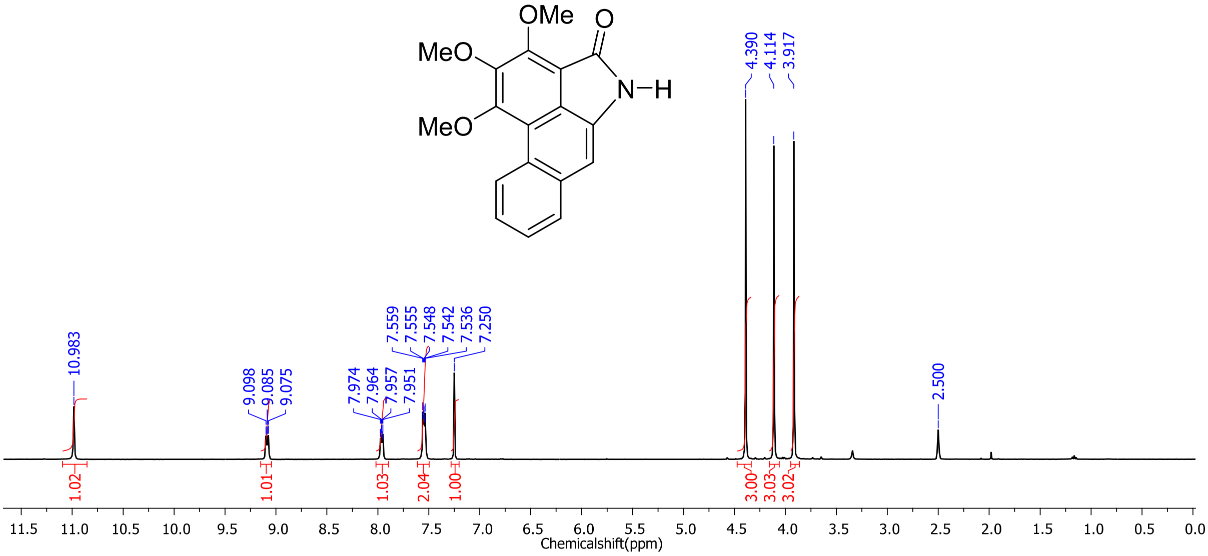

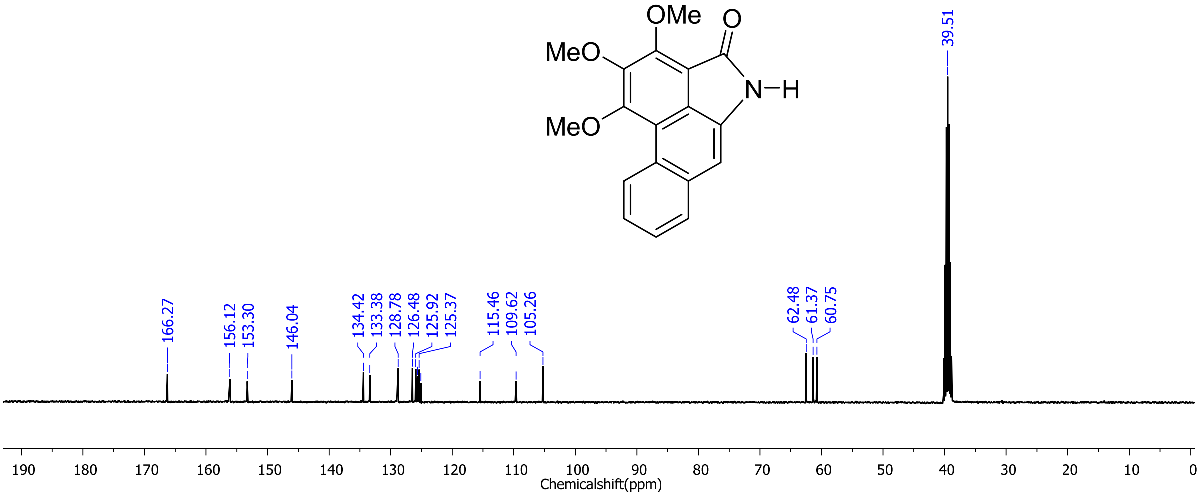

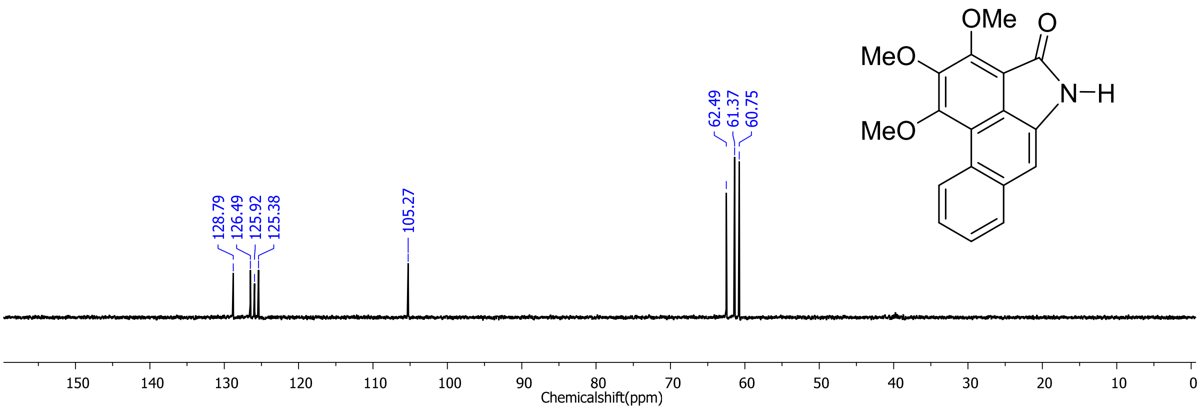

Supplement: Supplementary file 1 [file SC-008-C7SC00161D-s001.pdf]
